# Plasma Proteomic Study in Pulmonary Arterial Hypertension Associated with Congenital Heart Diseases

**DOI:** 10.1038/srep36541

**Published:** 2016-11-25

**Authors:** Xi Zhang, Hai-Tao Hou, Jun Wang, Xiao-Cheng Liu, Qin Yang, Guo-Wei He

**Affiliations:** 1 Department of Surgery & Center for Basic Medical Research, TEDA International Cardiovascular Hospital, Chinese Academy of Medical Sciences (CAMS) & Peking Union Medical College (PUMC), Tianjin & The Affiliated Hospital of Hangzhou Normal University & Zhejiang University, Hangzhou, China; 2 TEDA International Cardiovascular Hospital, CAMS & PUMC, Tianjin, China; 3 The Chinese University of Hong Kong, Shatin, China; 4 Department of Surgery, Oregon Health and Science University, Portland, Oregon, USA

## Abstract

Pulmonary arterial hypertension associated with congenital heart disease (CHD-PAH) has serious consequence and plasma protein profiles in CHD-PAH are unknown. We aimed to reveal the differential plasma proteins in 272 CHD patients with or without PAH. Various types of CHD-PAH were studied. Differential plasma proteins were first detected by iTRAQ proteomic technology and those with significant clinical relevance were selected for further ELISA validation in new cohort of patients. Among the 190 differential plasma proteins detected by iTRAQ, carbamoyl-phosphate synthetase I (CPSI, related to urea cycle and endogenous nitric oxide production) and complement factor H-related protein 2 (CFHR2, related to complement system and coagulant mechanism) were selected for further ELISA validation in new cohort of 152 patients. Both CPSI and CFHR2 were down-regulated with decreased plasma levels (p < 0.01). Thus, we for the first time in CHD-PAH patients identified a large number of differential plasma proteins. The decreased CPSI expression in CHD-PAH patients may reveal a mechanism related to endogenous nitric oxide and the decrease of CFHR2 protein may demonstrate the deficiency of the immune system and coagulation mechanism. The findings may open a new direction for translational medicine in CHD-PAH with regard to the diagnosis and progress of the disease.

Pulmonary arterial hypertension (PAH) is a common complication of congenital heart disease, affecting both disease progression and prognosis. The pathogenesis of PAH is complex, involving multiple modulating genes and environmental factors. The physiological impairment can lead to vasoconstriction, endothelial cell dysfunction and proliferation/fibrosis of vascular smooth muscle cell, inflammation, remodeling and *in-situ* thrombosis^1^,^2^. PAH associated with congenital heart disease (CHD-PAH) is often developed with left-to-right shunt, and characterized by increased pulmonary vascular resistance and remodeling, leading to severe consequence such as right ventricular failure and death. These processes involve a multitude of cellular and molecular elements.

Proliferation of smooth muscle cells in the small peripheral pulmonary arteries is a common characteristic in all forms of PAH and inflammatory mechanism also seems to play an important role in certain forms of PAH^3^. In response to vascular abnormalities, platelets can produce prothrombotic, vasoactive or mitogenic factors, that participate in vasoconstriction and vascular remodeling of idiopathic pulmonary hypertension^4^,^5^. In all forms of PAH, the progressive vasculopathy is a complex with a broad imbalance of vasodilators, such as nitric oxide (NO) and prostacyclin, and vasoconstrictors, such as endothelin-1 (ET-1) and thromboxane A_2_. This condition likely precedes the development of secondary aberrant cellular proliferation. Classic vasodilator systems are dysregulated with decreases in endothelial NO synthase (eNOS) function caused by enzymatic uncoupling, decreases in production of prostacyclin (cyclooxygenase-2 dysfunction), and increased abundance and activity of the vasoconstrictor and mitogenic ET-1 signaling system^6^. However, there are few reports on proteomics of this clinical syndrome.

Plasma serves as an ideal source of disease biomarkers study because it circulates through, or comes in contact with the majority of organs. During the development of the disease, some proteins are secreted or shed by the organs or tissues, which appear to be the potential biomarker for the disease^7^. We recently used proteomic methods to demonstrate the plasma protein changes in CHD patients^8^ that may reveal the possible mechanisms for the prolonged bleeding time in patients with tetralogy of Fallot and the susceptibility to pulmonary infections in patients with CHDs. More recently, we have further reported other protein changes in CHD patients^9^ with potential clinical implications. We also have for the first time identified alterations of 14 differential proteins or polypeptides in the plasma of patients with various valvular heart diseases, which indicate the possible genetic deficiency in these patients^10^.

On the basis of these studies, in the present study, we used iTRAQ proteomic methods to investigate the plasma proteins from completely new group of patients with CHD-PAH and healthy controls in order to identify the differential proteins related to the pathogenesis of CHD-PAH.

## Materials and Methods

### Study population

From January 2013 to May 2014, 266 congenital heart disease patients with or without pulmonary arterial hypertension (PAH) were enrolled at TEDA International Cardiovascular Hospital, Tianjin, China. The study and experimental protocols were approved by the Ethics Committee (Institutional Review Board) of TEDA International Cardiovascular Hospital, Tianjin, China. We confirm that the informed consent was obtained from all subjects - the parents or guardians of the children with congenital heart disease (CHD). In addition to this, we confirm that all methods were performed in accordance with the relevant ethical guidelines and regulations.

Routine clinical assessment of the patients was performed. All patients underwent corrective surgery and the diagnosis was verified by preoperative echocardiography and/or CT angiography, and corrective surgery. The diagnosis of PAH was made on the guidelines by ACCF/AHA^11^ and European guidelines^12^. According to ACCF/AHA 2009 expert consensus document on pulmonary hypertension^11^, PAH was defined as mean PAP > 25 mmHg at rest, >30 mmHg during exercise, or systolic PAP > 40 mmHg. In the present study, PAH was defined as either systolic PAP > 40 mmHg or mean PAP ≥ 25 mmHg at rest either by a transthoracic Doppler echocardiography or direct measurement from the main pulmonary artery during surgery and this is in accordance with European guidelines recently published^12^. The PAH was then confirmed during corrective surgery with direct measurement of pulmonary artery pressure. The enrolled CHD/CHD-PAH phenotypes included ventricular septal defects (VSD) with PAH (60), VSD without PAH (41), atrial septal defects (ASD) with PAH (41), ASD without PAH (41), mixed type of heart defects (two or more defects of VSD, ASD and patent ductus arteriosus [PDA]) with PAH (41) and without PAH (41). The phenotypes of patients in these groups are listed in Data file S1. All patients underwent corrective surgery for the heart defects. The patients were recruited for the iTraq proteomic study and for the further validation (ELISA) of the differential proteins found in the iTraq proteomic study were consecutive and randomized with no bias for selection.

In addition, 57 normal children with same ethnic, gender, and age were recruited in the study as normal control. The control group was chosen from normal body checking or congenital heart disease-screening program at the hospital. All control subjects were confirmed by clinical screening plus echocardiography that confirmed no cardiac diseases. The demographics of the CHD/CHD-PAH patients and controls in the study of iTRAQ and ELISA are shown in Tables 1 and 2.

Due to the fact that patients with ASDs develop PAH at later stages of the disease, in the present study, patients with ASD and PAH inevitably had older age compared to the patients with ASD without PAH and to the control subjects. In fact, those patients with ASD and PAH were 9.3 ± 2.4 year-old vs. 4.1 ± 0.7 year-old in ASD without PAH and 3.8 ± 0.6 year-old in the control (Table 2). The age of the control group was chosen to match most all other groups except this group.

### Plasma samples

The study protocol was approved by the Ethics Committee of the hospital and informed consent was obtained from their parents or guardians. Blood samples were taken from patients before surgery. From each sample, 2 ml blood was harvested 24–48 hours before the day of surgery in collection tubes with EDTA and prepared as described previously^13^ and then centrifuged at 1500 g for 10 min, the plasma was separated from the blood cells. Plasma was then collected, divided into aliquots, and stored frozen at −80 °C until the analysis was carried out.

### Sample preparation

#### Plasma High-Abundance Protein Depletion

Plasma samples from VSD-PAH patients, VSD patients, ASD-PAH patients, ASD patients, Mix-PAH patients, Mix patients and healthy controls (n = 20 for each) were processes to deplete the top-two (albumin, IgG) high abundance proteins using the ProteoExtract^TM^ Albumin/IgG Removal Kit (Calbiochem, La Jolla, CA, USA). Samples were processed according to the manufacturer’s instructions.

#### Solution Digestion and iTRAQ Labeling

The eluted samples were mixed in 0.5 M triethylammonium bicarbonate (TEAB) buffer with 1 mM phenylmethyl sulfonyl fluoride and 0.1% SDS, followed by sonication for 5 min and centrifugation at 20 000 g for 30 min. The supernatant was transferred to another tube, 0.5 M TEAB buffer was added to the pellet to repeat the protein extraction, and the sample was again centrifuged at 20 000 g for 30 min. Proteins in the combined supernatant were reduced (10 mM DTT, 56 °C for 60 min), alkylated (55 mM iodoacetamide, room temperature for 60 min), precipitated by precooled acetone at −20 °C for 30 min, and then centrifuged at 20 000 g for 30 min. The pellet was washed twice with acetone, and the final pellet was dissolved in 0.5 M TEAB buffer with 0.1% SDS, sonicated for 5 min, and centrifuged at 20 000 g for 30 min. The supernatant was used for liquid digestion, and the protein concentration was determined using the Bradford assay.

For the iTRAQ/Shotgun experiment, 3.3 ug of trypsin was added to 100 ug of the protein solution for protein digestion at 37 °C for 24 h. Then, 1 ug of trypsin was added again, and the sample was digested for 12 more hours. The digests were dried in a Speedvac. Each precipitate was dissolved in 30 ul of 0.5 M TEAB and mixed with 70 ul of isopropanol. The protein were labeled with iTRAQ reagent (AB SCIEX, Framingham, MA, USA) 113, 114, 115, 116, 117, 118, 119 and 121, respectively^14^. *iTRAQ Labeling* procedure was followed by validation in individual patients.

### SCX and RP nanoLC-MS/MS Analysis of Labeled Peptide

The peptides were dried in a Speedvac and dissolved in 1 ml of buffer A (10 mM KH_2_PO_4_ in 25% ACN at pH 2.8). After adjusting the pH to 3 with H_3_PO_4_, the sample was fractionated using strong cation-exchange chromatography (SCX) on an HPLC (Shimadzu, Kyoto, Japan) equipped with a silica-based SCX column (250 mm*4.6 mm, Phenomenex, Torrance, CA, USA). A total of 28 fractions were collected at a rate of 1 ml/min with a buffer B (10 mM KH_2_PO_4_ and 2 M KCl in 25% ACN, pH 2.8) gradient as following: 0% for 50 min, 5% for 51 min, 30% for 71 min, 50% for 76 min, 50% for 81 min, 100% for 86 min, and 100% for 96 min. The fractions were desalted with a strata-X 33 mm PolyRevStage SPE (Phenomenex) following the manufacturer’s instructions and dried in a Speedvac. Then, 30 ul of 0.1% FA was added to each dried fraction tube, and 1 ul of the re-dissolved solution was spotted on the target well of an Anchor-chip plate for MALDI-TOF testing. After the MALDI-TOF (Bruker Daltonics, Germany) testing, the peptides in the tubes with few peaks resulted in 16 SCX-separated fractions. Each SCX fraction was loaded on a Prominence Nano HPLC system (Ultimate 3000, Germering, Germany) mounted with a 10 cm reversed phase C18 column (ID 75 mm, 5μm particles, 300 A aperture) and separated over a 40 min acetonitrile gradient from 5 to 35% in 0.1% FA combined with a Q Exactive mass spectrometer (Thermo Fisher Scientific, MA, USA). The data were acquired using a data-dependent data acquisition mode in which, for each cycle, the 20 most abundant multiply charged peptides (2+ to 4+) with an m/z between 350 and 2000 were selected for MS/MS with the 15-s dynamic exclusion setting.

### iTRAQ data analysis and bioinformatic analysis

Protein identification was performed by using Mascot search engine (Matrix Science, London, UK; version 2.3.0) against Uniprot_Human Database containing 216686 sequences. And proteins identified as showing expression changes were tested for conformity to the following conditions: i) a false discovery rate (FDR) <1% (FDR was estimated by ‘decoy database searching’ using the Proteome Discoverer 1.3); and ii) protein confidence >99% (‘unused ProtScore’ >2). Unused ProtScore is defined as −log (1-% confidence/100). Proteins fulfilling these criteria were considered to have ‘statistical significance’. Blast2GO software was used to get the proteins Gene Ontology (GO) annotation. The expect value <0.001 was used to cut off the Blast result. And the GO term with Blast2GO’s score >30 to be consider. The Pathway analysis of Kyoto Encyclopedia of Genes and Genomes (KEGG) was used to get protein that identified KO annotations and pathway identify. The Fisher Exact test was used for the Pathway enrichment.

The identification of candidate proteins for further validation was based on 1) expressed differently in all tested groups between specific CHD with/without PAH; 2) potential functional or pathological significance in PAH; 3) more than 1 peptide was identified by LC-MS/MS; and 4) not been reported before in CHD-PAH patients at the protein level.

### Enzyme-Linked Immunosorbent Assay

In new group of patients, further validation of candidate proteins was performed by using human enzyme-linked immunosorbent assay (ELISA) kits (CUSABIO BIOTECH, Life Sciences Advanced Technologies Inc, USA). By the selection criteria mentioned above, there were two proteins, carbamoyl-phosphate synthetase I and complement factor H-related Protein 2, that were further validated in the plasma. The methods followed the manufacturer’s instructions.

### Statistical Analysis

SPSS 16.0 software (SPSS Inc, Chicago, IL) and GraphPad Prism 5 Demo software (GraphPad Software, San Diego, CA) were used for statistical analysis. Data are expressed as mean ± SEM. When comparison was made among three groups one-way ANOVA and post hoc test were used. Bonferroni test (when equal variances were assumed) or Dunnett’s T3 test (when equal variances were not assumed) were used as the post-hoc tests. When appropriate, unpaired t test was used to compare values between two groups. Statistical significance was defined as P < 0.05.

## Results

### Patient characteristics

The demographics of the cohorts are summarized in Tables 1 and 2. No significant differences were present among the groups except for the age of atrial septal defect associated with pulmonary arterial hypertension (ASD-PAH) and atrial septal defect (ASD) group compared with controls in iTRAQ proteomic study (Table 1). Similar differences of age between ASD-PAH and Mix group also existed in the ELISA validation study (Table 2). The age difference of ASD and other patients is due to the fact that ASD patients are usually operated at older age compared to other patients.

### Proteomic analysis

Using iTRAQ and LC-MS/MS, the differential proteins in the plasma were compared in these patients:

1. VSD-PAH/VSD/controls group:

  (1) VSD-PAH patients.

  (2) VSD patients.

  (3) Normal control.

2. ASD-PAH/ASD/controls group:

  (1) ASD-PAH patients.

  (2) ASD patients.

  (3) Normal control.

3. Mix-PAH/Mix/controls group:

  (1) Mix-PAH patients.

  (2) Mix patients.

  (3) Normal control.

**Figure 1:**
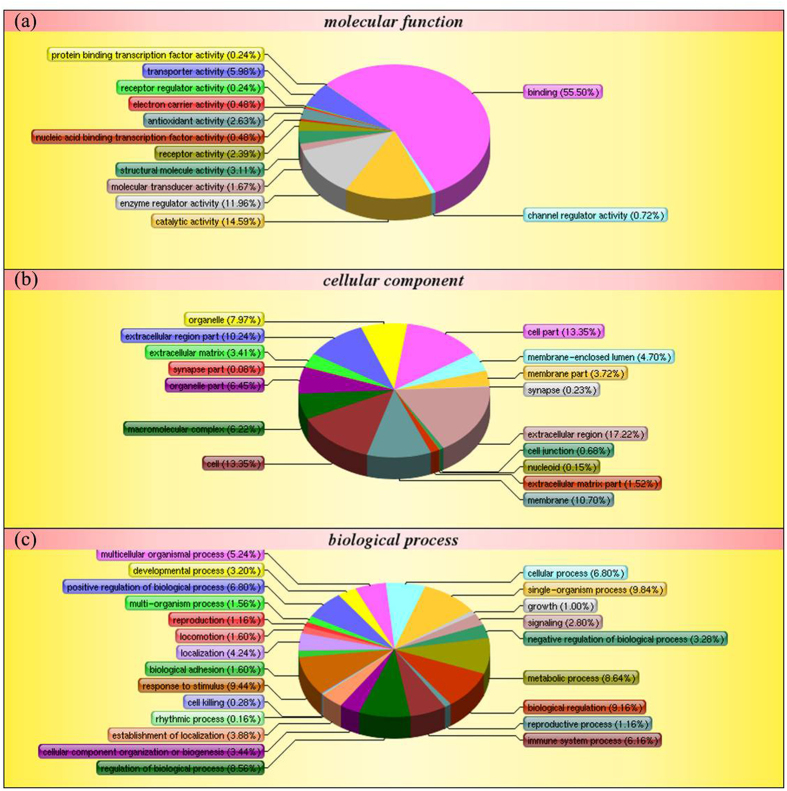
Classification of identified proteins based on their functional annotations using gene ontology (GO) molecular function (**a**), biological processes (**b**), and cellular components (**c**). These analyses were performed with the 1009 proteins identified in both CHD-PAH and CHD compared to the controls. When more than one assignment was available for a given protein, all the functional annotations were considered in the analyses.

Mass spectrometry identified 2371 peptides or proteins, of which 1044 were found to be statistically significant, i.e., the proteins fulfilled both the FDR <1% and protein confidence >99% criteria. Of the 1044 proteins, 190 proteins in VSD-PAH group, 185 proteins in ASD-PAH group and 190 proteins in Mix-PAH group were increased or decreased (>1.2-fold or <0.833-fold) relative to the control samples. The cut-off value is recommended by literature^15^,^16^. Differential proteins expressed in all 6 patient groups with CHD/CHD-PAH compared to the control, identified by iTRAQ were listed in Table 3. In addition, all differentially expressed proteins identified in ***iTRAQ*** study are listed in Data file S2. Specifically, eight proteins were significantly lower and two significantly higher in the disease groups. The peptide mass fingerprint spectra are also provided in Table S1 and Figs S1–10. Of these, two proteins (CPSI and CFHR2) which were associated with PAH were selected for following validation. Compared to CHD, the pathway involved in CHD-PAH with p-value < 0.05 was listed in Table 4. Further, 1009 (i.e., identified in both CHD-PAH and CHD vs. controls) of the identified proteins were used for the ontology analysis (versus 1044). The ontology analysis (Fig. 1) of the identified proteins indicated the relevance and diversity of molecular functions. With regard to gene ontology (GO) molecular function classification (Fig. 1a), protein binding (55.5%), catalytic activity (14.6%), and enzyme regulator activity (11.96%) are the categories in which most of the proteins were identified. With regard to GO biological process classification, many of the identified proteins were involved in biological regulation (9.16%), response to stimulus (9.44%), or regulation of biological process (8.56%) (Fig. 1b). Similarly, in GO cellular component classification (Fig. 1c), the identified proteins are related to extracellular region (17.22%), cell (13.35%), or membrane (10.70%).

### Validation of the candidate protein by ELISA

**Figure 2:**
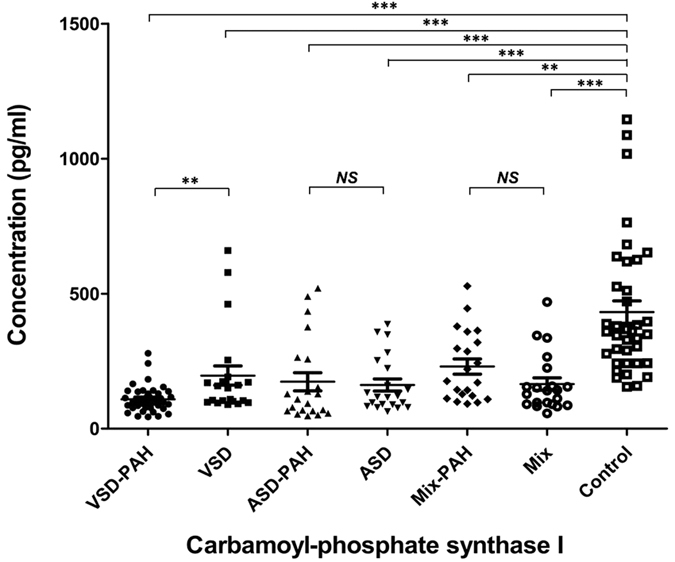
ELISA was used to measure plasma levels of carbamoyl-phosphate synthase I (CPSI) in CHD-PAH/CHD patients (n = 145) and controls (n = 37). Plasma CPSI levels in patients with VSD-PAH (109.2 ± 7.718 pg/ml, n = 40; *P* = 0.000 by ANOVA compared with control group) and VSD (196.5 ± 35.68 pg/ml, n = 21; *P* = 0.000 by ANOVA compared with control group) were lower than that in the healthy controls (431.8 ± 41.42 pg/ml, n = 37). The plasma CPSI level in ASD-PAH patients (173.7 ± 33.58 pg/ml, n = 21; P < 0.0001 by unpaired t test) and ASD patients (161.9 ± 22.51 pg/ml, n = 21; *P* = 0.000 by ANOVA compared with control group) was significantly lower than that in normal controls (431.8 ± 41.42 pg/ml, n = 37). The plasma CPSI level in Mix-PAH patients (230.1 ± 28.18 pg/ml, n = 21; *P* = 0.000 by ANOVA compared with control group) and Mix patients (165.0 ± 23.17 pg/ml, n = 21; *P* = 0.000 by ANOVA test compared with control group) was significantly lower than that in normal controls (431.8 ± 41.42 pg/ml, n = 37). Data are shown as mean ± SEM.

To validate the altered plasma proteins CPSI and CFHR2, ELISA was used to measure plasma levels of these proteins in patients. The demographics of study population in the study of ELISA are shown in Table 2. The plasma CPSI level in VSD-PAH patients (109.2 ± 7.718 pg/ml, n = 40; *P* = 0.000 by ANOVA compared with control group) and VSD patients (196.5 ± 35.68 pg/ml, n = 21; *P* = 0.000 by ANOVA compared with control group) were significantly lower than that in normal controls (431.8 ± 41.42 pg/ml, n = 37; Fig. 2). Importantly, the CPSI protein was also confirmed to be significantly decreased in VSD-PAH patients compared with VSD patients (*P* = 0.012 by ANOVA). The plasma CPSI level in ASD-PAH patients (173.7 ± 33.58 pg/ml, n = 21; *P* = 0.000 by ANOVA compared with control group) and ASD patients (161.9 ± 22.51 pg/ml, n = 21; *P* = 0.000 by ANOVA compared with control group) was significantly lower than that in normal controls (431.8 ± 41.42 pg/ml, n = 37; Fig. 2), although there was no significant difference between ASD-PAH patients and ASD patients (*P* = 0.97 by ANOVA). The plasma CPSI level in Mix-PAH patients (230.1 ± 28.18 pg/ml, n = 21; *P* = 0.000 by ANOVA compared with control group) and Mix patients (165.0 ± 23.17 pg/ml, n = 21; *P* = 0.000 by ANOVA test compared with control group) was significantly lower than that in normal controls (431.8 ± 41.42 pg/ml, n = 37; Fig. 2), although similar to the ASD patients, there was no significant difference between Mix-PAH patients and Mix patients (*P* = 0.15 by ANOVA).

**Figure 3:**
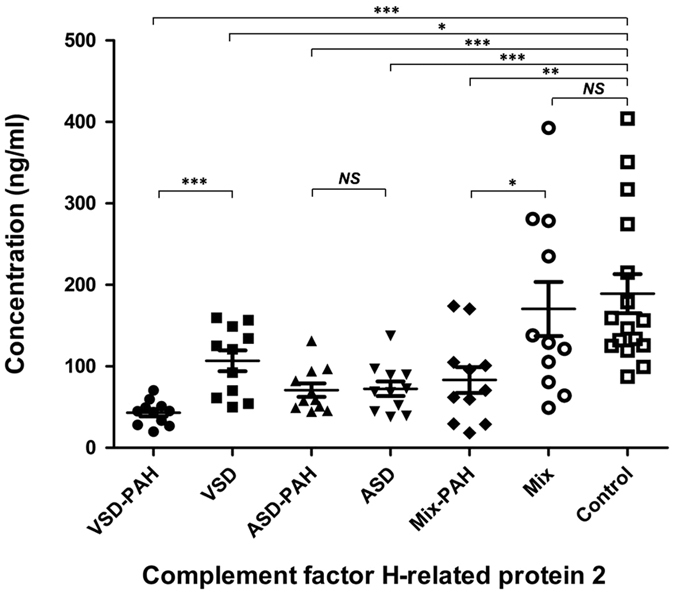
ELISA was used to measure plasma levels of complement Factor H-related Protein 2 (CFHR2) in CHD-PAH/CHD patients (n = 66) and controls (n = 16). The CFHR2 concentration in VSD-PAH patients (42.99 ± 4.534 ng/ml, n = 11; *P* = 0.000 by ANOVA compared with control group) and VSD patients (165.0 ± 23.17 ng/ml, n = 11; *P *=* 0.014 by ANOVA compared with control group*) was significantly lower than that in normal controls (189.1 ± 24.01 ng/ml, n = 16). The plasma CFHR2 level in ASD-PAH patients (70.92 ± 8.267 ng/ml, n = 11; *P *=* 0.000 by ANOVA compared with control group*) and ASD patients (72.48 ± 8.991 ng/ml, n = 11; *P *=* 0.000 by ANOVA compared with control group*) was significantly lower than that in normal controls (189.1 ± 24.01 ng/ml, n = 16). The plasma CFHR2 level in Mix-PAH patients (83.23 ± 15.96 ng/ml, n = 11; *P *=* 0.006 by ANOVA compared with control group*) was significantly lower than that in normal controls (189.1 ± 24.01 ng/ml, n = 16). There was no significant difference between Mix patients (170.4 ± 33.15 ng/ml, n = 11; P = 0.644 by unpaired t test; Fig. 3) and controls. The difference between Mix-PAH and Mix patients was marginal (*P* = 0.028 by unpaired t test; *P* = 0.064 by ANOVA; Fig. 3). Data are shown as mean ± SEM.

The CFHR2 concentration in VSD-PAH patients (42.99 ± 4.534 ng/ml, n = 11; *P* = 0.000 by ANOVA compared with control group) and VSD patients (165.0 ± 23.17 ng/ml, n = 21; *P* = 0.014 by ANOVA compared with control group) were significantly lower than that in normal controls (189.1 ± 24.01 ng/ml, n = 16; Fig. 3). Importantly, the CFHR2 protein was also confirmed to be significantly decreased in VSD-PAH patients compared with VSD patients (*P* = 0.000 by ANOVA). The plasma CFHR2 level in ASD-PAH patients (70.92 ± 8.267 ng/ml, n = 11; *P* = 0.000 by ANOVA compared with control group) and ASD patients (72.48 ± 8.991 ng/ml, n = 11; *P* = 0.000 by ANOVA compared with control group) were significantly lower than that in normal controls (189.1 ± 24.01 ng/ml, n = 16; Fig. 3), although there was no significant difference between ASD-PAH and ASD patients. The plasma CFHR2 level in Mix-PAH patients (83.23 ± 15.96 ng/ml, n = 11; *P* = 0.006 by ANOVA compared with control group) was significantly lower than that in normal controls (189.1 ± 24.01 ng/ml, n = 16). However, the difference between Mix-PAH and Mix patients (170.4 ± 33.15 ng/ml, n = 11) was marginal (*P* = 0.028 by unpaired t test; *P* = 0.064 by ANOVA; Fig. 3).

## Discussion

The present study for the first time, by using the iTRAQ proteomic technology in CHD-PAH patients, has found that (1) a large number of proteins are altered in the plasma of CHD-PAH patients; (2) decreased CPSI expression in CHD-PAH patients may reveal a mechanism that is responsible for decreased endogenous NO that is a critical pathway in the development of PAH; and (3) the decrease of CFHR2 protein, that may possibly activate the complement cascade, in the plasma of CHD-PAH patients may demonstrate the deficiency of the immune system and coagulation mechanism in these patients.

Recently, proteomic methods have been used to investigate the protein changes in hereditary PAH that used 2-dimensional PAGE in combination with liquid chromatography/tandem mass spectrometry analysis^17^. To our knowledge, the present study is the first proteomic study on the plasma of CHD-PAH patients by using iTRAQ technology.

The present study provides potentially important insights into plasma proteome changes in CHD-PAH. We found abundant differentially expressed plasma proteins associated with molecular function, biological processes, and cellular components. The involved various biological processes include extracellular matrix, binding, catalytic activity, and biological regulation (Fig. 1). According to gene ontology (GO) analysis between CHD-PAH and CHD, several genes are involved in blood coagulation, hemostasis, wound healing, cytoskeletal protein binding, and platelet activation (Table S2.). The analysis suggests that pulmonary arterial remodeling in PAH are likely caused by different molecular mechanisms and may require specific therapeutic options.

On the basis of our iTRAQ results, we compared the plasma proteins among CHD patients with or without PAH and the normal controls. In the present study, we have detected ~190 differential proteins in different types of CHD-PAH patients in comparison to the control. Most of them referred to innate immunity, inflammation and tissue injury, and platelet or coagulation proteins.

**Figure 4:**
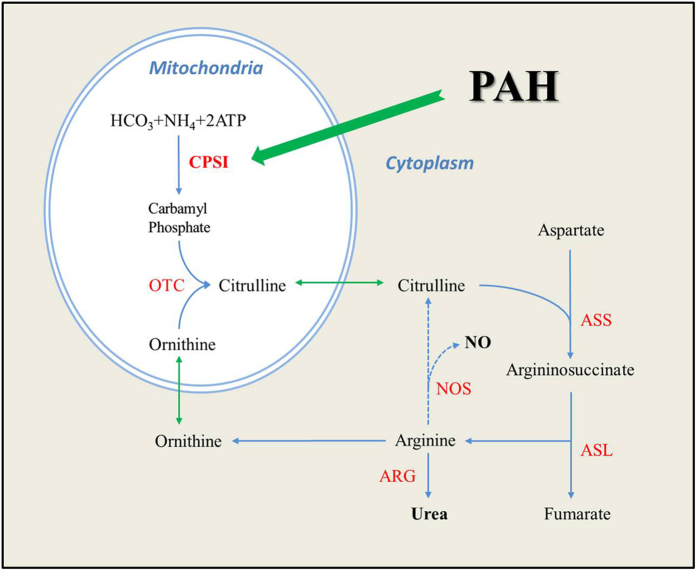
CPSI is related to the urea cycle that regulates NO production. CPSI is the rate limiting step in the urea cycle, and it was found down-regulated in the plasma of CHD-PAH in our study. ASS, argininosuccinate synthase; ASL, argininosuccinate lyase; NOS, nitric oxide synthase; OTC, ornithine transcarbamylase. (The diagram is modified from Pearson DL. *et al*.^22^).

Among the 10 differentially expressed proteins in the plasma of CHD-PAH and CHD patients in comparison to the control, we chose two of them (CPSI and CFHR2) for further validation that were also expressed differently between the specific CHD with/without PAH. In fact, these two proteins also have clear implications in the pathology of PAH. CPSI is related to the urea cycle that regulates NO production (see Fig. 4 for the mechanism). In contrast, CFHR2 is related to the complement system and therefore is related to immune mechanism. The down-regulated expression of CPSI was validated in new cohort of patients with various types of CHD-PAH and the results clearly showed that CPSI was altered in all CHD-PAH patients. In particular, in VSD-PAH and VSD patients compared to the control, CPSI was decreased in VSD and further decreased in VSD-PAH patients, suggesting that CPSI may serve as an important biomarker for CHD-PAH.

CPSI is a mitochondrial enzyme that is related to the urea cycle as mentioned above (Fig. 4). Endogenous NO is critical for the maintenance of normal pulmonary arterial pressure^18^,^19^,^20^ and is derived from arginine supplied by the urea cycle^21^. The rate-limiting step in the urea cycle is catalyzed by the mitochondrial enzyme CPSI^22^. Vascular endothelial cells synthesize endogenous L-arginine by recycling L-citrulline, using argininosuccinic acid synthase and lyase^23^,^24^ and convert L-arginine via nitric oxide synthase (NOS) to L-citrulline and NO. As well known, NO induces vasodilation, inhibits platelet aggregation and leukocyte adhesion, inhibits vascular smooth muscle cell proliferation, and modulates oxidative stress^25^. Therefore, the altered CPSI is possibly related to the alteration of the above biological processes through reduction of the NO production. In fact, polymorphism in the gene encoding CPSI has been suggested to influence NO production as well as vascular smooth muscle reactivity^26^ and the CPSI T1405N genotype appears to be an important new factor in predicting susceptibility to increased pulmonary artery pressure following surgical repair of congenital cardiac defects in children^27^. Our study is the first that reveals the CPSI protein alteration in CHD-PAH patients. Our study also has a particularly important clinical implication because the CPSI protein alteration in CHD-PAH patients was found in the plasma of the patients. The lower plasma concentration of CPSI in CHD-PAH patients seems to reflect the impaired NO production which is important in maintaining PAH, because more subtle changes in carbamoyl-phosphate synthetase affect the availability of arginine and citrulline^22^ that are involved in the production of NO. Indeed, the present study suggests that the plasma CPSI level may reflect the pathology of PAH and the significant decrease of plasma CPSI level may indicate the presence of PAH. Further, the plasma CPSI level may be developed as a biomarker of PAH.

In this study, we also validated another down-regulated protein CFHR2 in the plasma of CHD-PAH patients. Factor H related proteins comprise a group of five plasma proteins: CFHR1, CFHR2, CFHR3, CFHR4 and CFHR5, and each member of this group binds to the complement C3b. CFHR2 is a novel complement regulator that binds to C3b and to the C3 convertase, controls the C3 convertase of complement, and regulates complement activation^28^. The complement system serves as a central component of innate immunity, regulating hemostasis and cooperating with the adaptive immune response^29^,^30^. The present study demonstrates down-regulated CFHR2 in VSD-PAH patients compared with VSD without PAH and healthy controls, suggesting that complement activation plays a pathophysiologic role in the development of PAH. The consumption of CFHR2 protein in the plasma is likely to activate the complement system, which could promote pulmonary vascular remodeling^31^ and contribute to coagulation by augmenting inflammation^32^. Since the physiological impairment of PAH includes *in-situ* thrombosis^1^,^2^, the alteration of the down-regulated CFHR2 in PAH patients may reflect the pathological changes of the coagulant system in PAH patients and the plasma level of this factor is also possibly developed as a biomarker of PAH.

Interestingly, compared with CHD, the pathways involved in CHD-PAH also shed light on the understanding of the factors contributing to the progress of PAH, such as extracellular matrix (ECM)-receptor interaction, focal adhesion, chemokine signaling pathway, and complement and coagulation cascades. Some of the pathways have been previously identified in PAH. For example, chemokines produced from small pulmonary artery of PAH patients appear to contribute to inflammatory cell recruitment and smooth muscle cell proliferation^33^. In addition, changes in ECM protein abundance in PAH have been reported. For example, It has been shown that human plasma levels of TIMP-4, tenascin-C, MMP-2, and NT-proBNP, all ECM interacting proteins, are elevated in PAH patients and are correlated with the severity of the disease^34^. In this study, several altered proteins are involved in the ECM-receptor interaction, such as collagen, thrombospondin (THBS), fibronectin, vitronectin, and von Willebrand Factor (vWF), all of which were increased in the plasma of CHD-PAH patients (Fig. S11). Tissue remodeling is generally characterized by extensive fibrosis and additional changes in the expression of cardiac ECM-associated proteins^35^,^36^,^37^ and the cardiac ECM provides structural support and facilitates mechanical, electrical, and chemical signals during homeostasis and in response to stress or injury^38^,^39^,^40^. Together with the findings previously reported, ECM factors play an important role in the remodeling referred to the process of PAH. This, however, need to be further studied in the future.

### Limitations

Our study has some limitations. Due to the fact that patients with ASDs develop PAH at later stages of the disease, in the present study, patients with ASD and PAH inevitably had older age compared to the patients with ASD without PAH and to the control subjects, although the age of other groups was matched with the control. This could be the reason for no significant differences of the level of candidate proteins between ASD or Mix patients with or without PAH in the validation. In addition, proteomic studies reveal abundant unnamed protein products as appeared in the differential protein list of the present study. Most of them are characterized by highly similar to some particular proteins. For example, a down-regulated protein with accession 158256710 is described as highly similar to thrombospondin. In fact, thrombospondin-1 is hypoxia-responsive mitogens that promote vascular smooth muscle cell proliferation as the critical process in the pathogenesis of pulmonary hypertension^41^. These candidate proteins remain to be further studied. In addition, FVIII and vWF are well known to be involved in endothelial dysfunction that is a key process in the pathogenesis of PAH. Due to our selection criteria mentioned before, these proteins were not of priority for validation in the present study.

In conclusion, the present study for the first time, by using the iTRAQ proteomic technology in CHD-PAH patients, identified a large number of differential plasma proteins. The decreased CPSI expression in CHD-PAH patients may reveal a mechanism that is responsible for decreased endogenous NO in the development of PAH and the decrease of CFHR2 protein may demonstrate the deficiency of the immune system and coagulation mechanism in these patients.

## Additional Information

**How to cite this article**: Zhang, X. *et al*. Plasma Proteomic Study in Pulmonary Arterial Hypertension Associated with Congenital Heart Diseases. *Sci. Rep.* **6**, 36541; doi: 10.1038/srep36541 (2016).

**Publisher’s note:** Springer Nature remains neutral with regard to jurisdictional claims in published maps and institutional affiliations.

## Supplementary Material

Supplementary Information

Supplementary File S1

Supplementary File S2

## Figures and Tables

**Table 1:** Demographics of Study Population in the study of iTRAQ.

Stage	Variables	VSD-PAH^†^	VSD^†^	ASD-PAH^†^	ASD^†^	Mix-PAH^†^	Mix^†^	Controls^†^
iTRAQ^*^	Number	20	20	20	20	20	20	20
	Sex (M/F)^‡^	12/8	10/10	10/10	7/13	12/8	11/9	13/7
	Age (years)	4.0 ± 0.8	3.7 ± 0.5	9.3 ± 2.2^§^	7.1 ± 1.5^||^	2.7 ± 0.7	3.8 ± 0.6	3.5 ± 0.7
	PASP (mmHg)^^¶^^	68.65 ± 3.04	N/A^^¶^^	47.47 ± 2.16	N/A^^¶^^	60.71 ± 4.55	N/A^^¶^^	N/A^^¶^^

^*^iTRAQ = isobaric tags for relative and absolute quantitation;

^†^VSD-PAH = ventricular septal defect with PAH; VSD = ventricular septal defect; ASD-PAH = atrial septal defect with PAH; ASD = atrial septal defect; Mix-PAH = mixed type heart defects (two or more defects of VSD, ASD and PDA) with PAH; Mix = mixed type heart defects (two or more defects of VSD, ASD and PDA) without PAH;

^‡^M = male, F = female;

^§,^*P < 0.05 vs. Controls;

^||,^*P < 0.05 vs. Controls;

^¶^PASP = Pulmonary arterial systolic pressure; N/A = not applicable.

**Table 2:** Demographics of Study Population in the study of ELISA.

Stage	Variables	VSD-PAH^†^	VSD^†^	ASD-PAH^†^	ASD^†^	Mix-PAH^†^	Mix^†^	Controls^†^
ELISA	Number	40	21	21	21	21	21	37
(CPSI)^*^	Sex (M/F)^‡^	21/19	10/11	8/13	9/12	6/15	8/13	20/17
	Age (years)	4.4 ± 0.8	5.0 ± 0.8	9.3 ± 2.4^§^	4.1 ± 0.7	2.2 ± 0.4	6.8 ± 1.5^||^	3.8 ± 0.6
	PASP (mmHg)^¶^	65.05 ± 3.35	N/A^¶^	56.43 ± 3.44	N/A^¶^	78.60 ± 4.59	N/A^¶^	N/A^¶^
ELISA	Number	11	11	11	11	11	11	16
(CFHR2)^*^	Sex (M/F)^‡^	7/4	6/5	5/6	4/7	3/8	5/6	9/7
	Age (years)	3.1 ± 0.7	4.8 ± 1.1	9.5 ± 3.0^#^	4.4 ± 1.1	1.7 ± 0.3	6.5 ± 1.6^**^	3.0 ± 0.5
	PASP (mmHg)^¶^	46.3 ± 1.69	N/A^¶^	62.64 ± 5.75	N/A^¶^	79.32 ± 7.52	N/A^¶^	N/A^¶^

^*^CPSI = Carbamoyl-phosphate Synthetase I; CFHR2 = complement Factor H-related Protein 2;

^†^VSD-PAH = ventricular septal defect with PAH; VSD = ventricular septal defect; ASD-PAH = atrial septal defect with PAH; ASD = atrial septal defect; Mix-PAH = mixed type heart defects (two or more defects of VSD, ASD and PDA) with PAH; Mix = mixed type heart defects (two or more defects of VSD, ASD and PDA) without PAH;

^‡^M = male, F = female;

^§,^*P < 0.05 vs. ASD; **P < 0.01 vs. Controls;

^||,^**P < 0.01 vs. Mix-PAH; *P < 0.05 vs. Controls;

^#,^*P < 0.05 vs. Controls;

**P < 0.01 vs. Mix-PAH;

^¶^PASP = Pulmonary arterial systolic pressure; N/A = not applicable.

**Table 3:** Differential proteins expressed in all 6 patient groups with CHD/CHD-PAH compared to the control, identified by iTRAQ (See Data file S2 for all differential proteins identified by iTraq study).

Accession	Protein	Score	Coverage	Unique Peptides	Peptides	VSD-PAH	VSD	ASD-PAH	ASD	Mix-PAH	Mix	Control
13937839	SAA1 protein	249.56	54.10%	2	5	0.346	0.566	0.47	0.761	0.375	0.306	1
1064908	complement Factor H-related Protein 2*	222.48	31.28%	3	6	0.497	0.612	0.601	0.883	0.641	0.575	1
3152372	anti-FactorVIII scFv	537.51	64.29%	1	10	0.499	0.751	0.5	0.637	0.538	0.726	1
56378229	Carbamoyl-phosphate synthetase I*	22.22	4.09%	1	1	0.503	0.742	0.47	0.681	0.399	0.544	1
47124510	APCS protein	30.38	23.91%	1	1	0.532	0.618	N/A†	N/A†	0.779	0.676	1
1769552	von Willebrand factor	27.48	3.73%	1	1	0.582	0.201	1.223	0.6	0.785	0.606	1
74355107	BRF1 protein	30.09	4.97%	1	1	0.606	N/A†	0.796	0.714	0.815	0.828	1
54304028	glyceraldehyde-3-phosphate dehydrogenase	45.44	17.44%	1	1	0.679	0.675	0.657	0.611	0.603	0.629	1
11122875	glycosylphosphatidylinositol phospholipase D	137.2	8.32%	3	3	1.685	1.28	1.765	1.804	1.674	1.446	1
20377087	intestinal lactoferrin receptor	171.75	29.07%	7	7	1.694	1.393	1.218	1.212	1.508	1.446	1

^*^Candidate proteins for validation.

^†^N/A represents data are not available.

**Table 4:** Pathway involved in the CHD-PAH patients compared to CHD.

#	Pathway	Diff Proteins with pathway annotation*	All Proteins with pathway annotation (1009)	P value	Pathway ID
VSD-PAH/VSD					
**1**	PPAR signaling pathway	4/39 (10.26%)	10 (0.99%)	0.000339653	ko03320
**2**	Focal adhesion	3/39 (7.69%)	14 (1.39%)	0.01447198	ko04510
**3**	ECM-receptor interaction	3/39 (7.69%)	11 (1.09%)	0.007112038	ko04512
**4**	Chemokine signaling pathway	2/39 (5.13%)	4 (0.4%)	0.008320181	ko04062
**5**	Cytokine-cytokine receptor interaction	2/39 (5.13%)	5 (0.5%)	0.01352894	ko04060
**6**	Vitamin digestion and absorption	2/39 (5.13%)	7 (0.69%)	0.02704753	ko04977
**7**	Type II diabetes mellitus	1/39 (2.56%)	1 (0.1%)	0.03865213	ko04930
**8**	Adipocytokine signaling pathway	1/39 (2.56%)	1 (0.1%)	0.03865213	ko04920
ASD-PAH/ASD					
**1**	Complement and coagulation cascades	4/19 (21.05%)	64 (6.34%)	0.02773601	ko04610
**2**	Focal adhesion	2/19 (10.53%)	14 (1.39%)	0.0267276	ko04510
**3**	Alcoholism	1/19 (5.26%)	2 (0.2%)	0.03732479	ko05034
**4**	Neuroactive ligand-receptor interaction	1/19 (5.26%)	3 (0.3%)	0.05548847	ko0408
Mix-PAH/Mix					
**1**	Malaria	4/49 (8.16%)	7 (0.69%)	0.000154843	ko05144
**2**	Aminoacyl-tRNA biosynthesis	2/49 (4.08%)	2 (0.2%)	0.002312521	ko00970
**3**	Osteoclast differentiation	2/49 (4.08%)	3 (0.3%)	0.006721696	ko04380
**4**	Cytokine-cytokine receptor interaction	2/49 (4.08%)	5 (0.5%)	0.02103969	ko04060
**5**	Vitamin digestion and absorption	2/49 (4.08%)	7 (0.69%)	0.04150761	ko04977
**6**	TGF-beta signaling pathway	2/49 (4.08%)	7 (0.69%)	0.04150761	ko04350
**7**	Endocytosis	1/49 (2.04%)	1 (0.1%)	0.04856293	ko04144
**8**	Renal cell carcinoma	1/49 (2.04%)	1 (0.1%)	0.04856293	ko05211
**9**	Mineral absorption	1/49 (2.04%)	1 (0.1%)	0.04856293	ko04978
**10**	Colorectal cancer	1/49 (2.04%)	1 (0.1%)	0.04856293	ko05210
**11**	Glycosaminoglycan degradation	1/49 (2.04%)	1 (0.1%)	0.04856293	ko00531
**12**	HTLV-I infection	1/49 (2.04%)	1 (0.1%)	0.04856293	ko05166
**13**	Toxoplasmosis	1/49 (2.04%)	1 (0.1%)	0.04856293	ko05145
**14**	Valine, leucine and isoleucine degradation	1/49 (2.04%)	1 (0.1%)	0.04856293	ko00280
**15**	Cell cycle	1/49 (2.04%)	1 (0.1%)	0.04856293	ko04110
**16**	Chronic myeloid leukemia	1/49 (2.04%)	1 (0.1%)	0.04856293	ko05220
**17**	Pancreatic cancer	1/49 (2.04%)	1 (0.1%)	0.04856293	ko05212

^*^The numerator is the number of proteins identified from the proteomic study associated to this pathway and the denominator is the total number of proteins known to be associated to this pathway.
