# Supplementary material for: Plasma Proteomic Study in Pulmonary Arterial Hypertension Associated with Congenital Heart Diseases
Source: Sci Rep. 2016 Nov 25;6:36541. doi: 10.1038/srep36541 (PMC5122864; doi:10.1038/srep36541)
Supplement: Supplementary Information [file srep36541-s1.doc]

**Plasma Proteomic Study in Pulmonary Arterial Hypertension Associated with Congenital Heart Diseases**

**Xi Zhang,** *PhD***, Hai-Tao Hou,** *MD,* **Jun Wang,** *MD*, **Xiao-Cheng Liu,** *MD*, **Qin Yang,** *PhD*, **and Guo-Wei He*,** *PhD, DSc*

Table S1**.** **The peptide mass fingerprint spectra of differentially expressed proteins in CHD/CHD-PAH patients identified by iTRAQ.**

| **Confidence** | **Sequence** | **Activation Type** | **Modifications** | **IonScore** | **ΔScore** | **Rank** | **Search Engine** | **Charge** | **Precursor m/z [Da]** | **ΔM**  **[ppm]** | **Annotated Spectrum** |
| --- | --- | --- | --- | --- | --- | --- | --- | --- | --- | --- | --- |
| **54304028 - glyceraldehyde-3-phosphate dehydrogenase [Homo sapiens]** | | | | | | | | | | | |
| High | gALQNIIPASTGAAk | HCD | N-Term(iTRAQ8plex); K15(iTRAQ8plex) | 45.44 | 1 | 1 | Mascot (2) | 3 | 674.06909 | -4.17 | Figure S1 |
| **3152372 - anti-FactorVIII scFv [Homo sapiens]** | | | | | | | | | | | |
| High | aTGIPDR | HCD | N-Term(iTRAQ8plex) | 31.73 | 1 | 1 | Mascot (2) | 2 | 517.29932 | -2.93 | Figure S2A |
| High | TFGQGTk | HCD | K7(iTRAQ8plex) | 27.09 | 1 | 1 | Mascot (2) | 2 | 521.79352 | -3.56 | Figure S2B |
| High | NTLYLQMNSLR | HCD |  | 24.22 |  | 3 | Mascot (2) | 2 | 676.85455 | 1.99 | Figure S2C |
| High | AEDTAVYYcAk | HCD | C9(Carbamidomethyl); K11(iTRAQ8plex) | 38.98 | 1 | 1 | Mascot (2) | 2 | 797.88989 | 0.02 | Figure S2D |
| High | FSGSGSGTDFTLTISR | HCD |  | 109.44 | 1 | 1 | Mascot (2) | 2 | 816.89673 | -0.17 | Figure S2E |
| High | EVQLLESGGGLVQPGGSLR | HCD |  | 51.34 | 0.04 | 1 | Mascot (2) | 2 | 948.51361 | 0.7 | Figure S2F |
| High | aEDTAVYYcAk | HCD | N-Term(iTRAQ8plex); C9(Carbamidomethyl); K11(iTRAQ8plex) | 57.94 | 1 | 1 | Mascot (2) | 2 | 949.99139 | -1.22 | Figure S2G |
| High | fSGSGSGTDFTLTISR | HCD | N-Term(iTRAQ8plex) | 56.94 | 1 | 1 | Mascot (2) | 2 | 968.99945 | -0.1 | Figure S2H |
| High | LScAASGFTFSSYAMSWVR | HCD | C3(Carbamidomethyl) | 49.79 | 1 | 1 | Mascot (2) | 2 | 1064.47864 | -5.99 | Figure S2I |
| High | eVQLLESGGGLVQPGGSLR | HCD | N-Term(iTRAQ8plex) | 82.68 | 1 | 1 | Mascot (2) | 3 | 734.07697 | -3.47 | Figure S2J |
| High | ASQSVSSSYLAWYQQkPGQAPR | HCD | K16(iTRAQ8plex) | 15.25 | 1 | 1 | Mascot (2) | 3 | 915.14319 | 1.62 | Figure S2K |
| High | gLEWVSAISGSGGSTYYADSVk | HCD | N-Term(iTRAQ8plex); K22(iTRAQ8plex) | 59.29 | 1 | 1 | Mascot (2) | 3 | 948.16071 | -1.55 | Figure S2L |
| High | aSQSVSSSYLAWYQQkPGQAPR | HCD | N-Term(iTRAQ8plex); K16(iTRAQ8plex) | 58.11 | 1 | 1 | Mascot (2) | 3 | 1016.54077 | -2.68 | Figure S2M |
| High | lEPEDFAVYYcQQAGLGPRTFGQGTk | HCD | N-Term(iTRAQ8plex); C11(Carbamidomethyl); K26(iTRAQ8plex) | 25.89 | 1 | 1 | Mascot (2) | 4 | 885.94427 | -13.8 | Figure S2N |
| **56378229 - carbamoylphosphate synthetase I [Homo sapiens]** | | | | | | | | | | | |
| High | kEPLFGISTGNLITGLAAGAk | HCD | K1(iTRAQ8plex); N-Term(iTRAQ8plex); K21(iTRAQ8plex) | 22.22 | 1 | 1 | Mascot (2) | 3 | 990.92767 | -2.39 | Figure S3 |
| **11122875 - glycosylphosphatidylinositol phospholipase D [Homo sapiens]** | | | | | | | | | | | |
| High | IADVTSGLIGGEDGR | HCD | N-Term(iTRAQ8plex) | 21.42 | 1 | 1 | Mascot (2) | 2 | 730.37335 | 0.43 | Figure S4A |
| High | aQYVLISPEASSR | HCD | N-Term(iTRAQ8plex) | 55.96 | 1 | 1 | Mascot (2) | 2 | 862.97729 | -0.68 | Figure S4B |
| High | iADVTSGLIGGEDGR | HCD | N-Term(iTRAQ8plex); C6(Carbamidomethyl); K10(iTRAQ8plex) | 57.98 | 1 | 1 | Mascot (2) | 2 | 882.4762 | 0.54 | Figure S4C |
| High | sWITPcPEEk | HCD | N-Term(iTRAQ8plex); K6(iTRAQ8plex) | 23.26 | 1 | 1 | Mascot (2) | 2 | 927.99121 | -6.94 | Figure S4D |
| **47124510 - APCS protein [Homo sapiens]** | | | | | | | | | | | |
| High | gYVIIkPLVWV | HCD | N-Term(iTRAQ8plex); K7(iTRAQ8plex) | 30.38 | 1 | 1 | Mascot (2) | 2 | 948.10077 | -1.91 | Figure S5 |
| **13937839 - SAA1 protein [Homo sapiens]** | | | | | | | | | | | |
| High | gNYDAAk | HCD | N-Term(iTRAQ8plex); K9(iTRAQ8plex) | 31.62 | 1 | 1 | Mascot (2) | 3 | 449.58685 | -4.78 | Figure S6A |
| High | SFFSFLGEAFDGAR | HCD | N-Term(iTRAQ8plex) | 76.89 | 1 | 1 | Mascot (2) | 2 | 775.86578 | -2.07 | Figure S6B |
| High | eANYIGSDk | HCD | N-Term(iTRAQ8plex) | 42.6 | 1 | 1 | Mascot (2) | 2 | 802.93701 | -4.53 | Figure S6C |
| High | sFFSFLGEAFDGAR | HCD |  | 62.41 | 1 | 1 | Mascot (2) | 2 | 927.97229 | 2.39 | Figure S6D |
| High | rGPGGVWAAEAISDAR | HCD | N-Term(iTRAQ8plex) | 26.7 | 1 | 1 | Mascot (2) | 2 | 959.01703 | 1.19 | Figure S6E |
| High | FFGHGAEDSLADQAADEWGR | HCD |  | 69.48 | 1 | 1 | Mascot (2) | 2 | 1089.97485 | -2.4 | Figure S6F |
| High | fFGHGAEDSLADQAADEWGR | HCD | K7(iTRAQ8plex) | 71.75 | 1 | 1 | Mascot (2) | 3 | 828.3858 | -4.09 | Figure S6G |
| **1064908 - complement Factor H-related Protein 2 [Homo sapiens]** | | | | | | | | | | | |
| High | GWSTPPk | HCD | K11(iTRAQ8plex) | 36.44 | 1 | 1 | Mascot (2) | 2 | 538.80371 | -3.78 | Figure S7A |
| High | LVYPScEEk | HCD | N-Term(iTRAQ8plex); C6(Carbamidomethyl); K9(iTRAQ8plex) | 20.22 | 1 | 1 | Mascot (2) | 2 | 714.87067 | -0.41 | Figure S7B |
| High | INHGILYDEEk | HCD | N-Term(iTRAQ8plex); C9(Carbamidomethyl); K10(iTRAQ8plex) | 16 | 1 | 1 | Mascot (2) | 2 | 817.93622 | -2.45 | Figure S7C |
| High | lVYPScEEk | HCD | C3(Carbamidomethyl); K13(iTRAQ8plex) | 31.66 | 1 | 1 | Mascot (2) | 3 | 578.31653 | -2.86 | Figure S7D |
| High | tGDIVEFVck | HCD | N-Term(iTRAQ8plex); K11(iTRAQ8plex) | 28.54 | 1 | 1 | Mascot (2) | 3 | 592.66278 | -4.74 | Figure S7E |
| High | ITcAEEGWSPTPk | HCD | N-Term(iTRAQ8plex); C3(Carbamidomethyl); K13(iTRAQ8plex) | 32.78 | 1 | 1 | Mascot (2) | 2 | 890.44843 | 0.36 | Figure S7F |
| High | iNHGILYDEEk | HCD | N-Term(iTRAQ8plex); Y7(iTRAQ8plex); K11(iTRAQ8plex) | 53.94 | 1 | 1 | Mascot (2) | 2 | 970.03864 | -2.33 | Figure S7G |
| High | iTcAEEGWSPTPk | HCD | K2(iTRAQ8plex); C17(Carbamidomethyl); K26(iTRAQ8plex) | 38.65 | 1 | 1 | Mascot (2) | 3 | 695.36517 | -6.4 | Figure S7H |
| High | iNHGILyDEEk | HCD | N-Term(iTRAQ8plex); K2(iTRAQ8plex); C17(Carbamidomethyl); K26(iTRAQ8plex) | 17.26 | 1 | 1 | Mascot (2) | 2 | 1122.1438 | 0.2 | Figure S7I |
| High | YkPFSQVPTGEVFYYScEYNFVSPSk | HCD |  | 19.22 | 1 | 1 | Mascot (2) | 3 | 1244.62537 | 1.69 | Figure S7K |
| High | ykPFSQVPTGEVFYYScEYNFVSPSk | HCD | Y1(iTRAQ8plex); K9(iTRAQ8plex) | 28.98 | 1 | 1 | Mascot (2) | 3 | 1346.02429 | -0.57 | Figure S7L |
| **1769552 - von Willebrand factor [Homo sapiens]** | | | | | | | | | | | |
| High | yTLFQIFSk | HCD |  | 27.48 | 1 | 1 | Mascot (2) | 3 | 585.67847 | -5.28 | Figure S8 |
| **20377087 - intestinal lactoferrin receptor [Homo sapiens]** | | | | | | | | | | | |
| High | TASYYSPYGQR | HCD | N-Term(iTRAQ8plex); C4(Carbamidomethyl) | 32.35 | 1 | 1 | Mascot (2) | 2 | 646.79926 | 0.49 | Figure S9A |
| High | eFTAGFVQFR | HCD | N-Term(iTRAQ8plex) | 18.83 | 1 | 1 | Mascot (2) | 2 | 753.40411 | -2.99 | Figure S9B |
| High | eWTcSSSPSLPR | HCD | N-Term(iTRAQ8plex); K10(iTRAQ8plex) | 38.81 | 1 | 1 | Mascot (2) | 2 | 855.9201 | -5.48 | Figure S9C |
| High | eITEAAVLLFYR | HCD | K19(iTRAQ8plex) | 24.19 | 1 | 1 | Mascot (2) | 2 | 864.99597 | 0.49 | Figure S9D |
| High | dLGIWHVPNk | HCD | N-Term(iTRAQ8plex); K3(iTRAQ8plex); C6(Carbamidomethyl) | 22.68 | 1 | 1 | Mascot (2) | 2 | 894.02124 | -4.05 | Figure S9E |
| High | TDTGFLQTLGHNLFGIYQk | HCD |  | 11.7 | 1 | 1 | Mascot (2) | 3 | 819.76959 | -5.61 | Figure S9F |
| High | eIkDEcPSAFDGLYFLR | HCD | N-Term(Gln->pyro-Glu); K8(iTRAQ8plex) | 21.16 | 1 | 1 | Mascot (2) | 3 | 890.13135 | -4 | Figure S9G |
| **74355107 - BRF1 protein [Homo sapiens]** | | | | | | | | | | | |
| High | qLEQVLSk | HCD |  | 15.77 | 1 | 1 | Mascot (2) | 2 | 616.36151 | -3.43 | Figure S10A |
| High | qLEQVLSk | HCD |  | 28.66 | 1 | 1 | Mascot (2) | 3 | 518.31842 | -7.19 | Figure S10B |

**Table S2. GO analysis between CHD-PAH and CHD.**

**A. GO analysis** between VSD-PAH and VSD - cellular component

| **Gene Ontology term** | **Cluster frequency** | **Protein frequency of use** | **P-value** |
| --- | --- | --- | --- |
| [vesicle](http://amigo.geneontology.org/amigo/term/GO:0031982) | 7 out of 14 genes, 50.0% | 46 out of 271 genes, 17.0% | 0.003530179 |
| [secretory granule](http://amigo.geneontology.org/amigo/term/GO:0030141) | 6 out of 14 genes, 42.9% | 39 out of 271 genes, 14.4% | 0.007617491 |
| [organelle](http://amigo.geneontology.org/amigo/term/GO:0043226) | 10 out of 14 genes, 71.4% | 105 out of 271 genes, 38.7% | 0.01166471 |
| [cytoplasmic membrane-bounded vesicle](http://amigo.geneontology.org/amigo/term/GO:0016023) | 6 out of 14 genes, 42.9% | 43 out of 271 genes, 15.9% | 0.01268163 |
| [membrane-bounded vesicle](http://amigo.geneontology.org/amigo/term/GO:0031988) | 6 out of 14 genes, 42.9% | 43 out of 271 genes, 15.9% | 0.01268163 |
| [platelet alpha granule lumen](http://amigo.geneontology.org/amigo/term/GO:0031093) | 5 out of 14 genes, 35.7% | 31 out of 271 genes, 11.4% | 0.01347062 |
| [secretory granule lumen](http://amigo.geneontology.org/amigo/term/GO:0034774) | 5 out of 14 genes, 35.7% | 32 out of 271 genes, 11.8% | 0.01548711 |
| [cytoplasmic membrane-bounded vesicle lumen](http://amigo.geneontology.org/amigo/term/GO:0060205) | 5 out of 14 genes, 35.7% | 32 out of 271 genes, 11.8% | 0.01548711 |
| [cytoplasmic vesicle](http://amigo.geneontology.org/amigo/term/GO:0031410) | 6 out of 14 genes, 42.9% | 45 out of 271 genes, 16.6% | 0.01598487 |
| [membrane-bounded organelle](http://amigo.geneontology.org/amigo/term/GO:0043227) | 9 out of 14 genes, 64.3% | 92 out of 271 genes, 33.9% | 0.01716481 |
| [intracellular membrane-bounded organelle](http://amigo.geneontology.org/amigo/term/GO:0043231) | 9 out of 14 genes, 64.3% | 92 out of 271 genes, 33.9% | 0.01716481 |
| [platelet alpha granule](http://amigo.geneontology.org/amigo/term/GO:0031091) | 5 out of 14 genes, 35.7% | 33 out of 271 genes, 12.2% | 0.01770488 |
| [vesicle lumen](http://amigo.geneontology.org/amigo/term/GO:0031983) | 5 out of 14 genes, 35.7% | 34 out of 271 genes, 12.5% | 0.02013311 |
| [membrane-enclosed lumen](http://amigo.geneontology.org/amigo/term/GO:0031974) | 7 out of 14 genes, 50.0% | 62 out of 271 genes, 22.9% | 0.02100324 |
| [organelle lumen](http://amigo.geneontology.org/amigo/term/GO:0043233) | 7 out of 14 genes, 50.0% | 62 out of 271 genes, 22.9% | 0.02100324 |
| [high-density lipoprotein particle](http://amigo.geneontology.org/amigo/term/GO:0034364) | 4 out of 14 genes, 28.6% | 23 out of 271 genes, 8.5% | 0.02241637 |
| [cytoplasmic part](http://amigo.geneontology.org/amigo/term/GO:0044444) | 9 out of 14 genes, 64.3% | 96 out of 271 genes, 35.4% | 0.02328698 |
| [extracellular region part](http://amigo.geneontology.org/amigo/term/GO:0044421) | 11 out of 14 genes, 78.6% | 135 out of 271 genes, 49.8% | 0.02472693 |
| [cytoplasmic vesicle part](http://amigo.geneontology.org/amigo/term/GO:0044433) | 5 out of 14 genes, 35.7% | 36 out of 271 genes, 13.3% | 0.02565572 |
| [endoplasmic reticulum](http://amigo.geneontology.org/amigo/term/GO:0005783) | 4 out of 14 genes, 28.6% | 25 out of 271 genes, 9.2% | 0.03007121 |
| [protein-lipid complex](http://amigo.geneontology.org/amigo/term/GO:0032994) | 4 out of 14 genes, 28.6% | 26 out of 271 genes, 9.6% | 0.03443598 |
| [plasma lipoprotein particle](http://amigo.geneontology.org/amigo/term/GO:0034358) | 4 out of 14 genes, 28.6% | 26 out of 271 genes, 9.6% | 0.03443598 |
| [intracellular organelle](http://amigo.geneontology.org/amigo/term/GO:0043229) | 9 out of 14 genes, 64.3% | 104 out of 271 genes, 38.4% | 0.04057455 |
| [cytoplasm](http://amigo.geneontology.org/amigo/term/GO:0005737) | 9 out of 14 genes, 64.3% | 106 out of 271 genes, 39.1% | 0.04613031 |

B. GO analysis between VSD-PAH and VSD – molecular function

| **Gene Ontology term** | **Cluster frequency** | **Protein frequency of use** | **P-value** |
| --- | --- | --- | --- |
| [cytokine activity](http://amigo.geneontology.org/amigo/term/GO:0005125) | 3 out of 12 genes, 25.0% | 4 out of 258 genes, 1.6% | 0.0003028239 |
| [cholesterol binding](http://amigo.geneontology.org/amigo/term/GO:0015485) | 3 out of 12 genes, 25.0% | 7 out of 258 genes, 2.7% | 0.002444296 |
| [sterol binding](http://amigo.geneontology.org/amigo/term/GO:0032934) | 3 out of 12 genes, 25.0% | 7 out of 258 genes, 2.7% | 0.002444296 |
| [lipoprotein particle binding](http://amigo.geneontology.org/amigo/term/GO:0071813) | 3 out of 12 genes, 25.0% | 7 out of 258 genes, 2.7% | 0.002444296 |
| [protein-lipid complex binding](http://amigo.geneontology.org/amigo/term/GO:0071814) | 3 out of 12 genes, 25.0% | 7 out of 258 genes, 2.7% | 0.002444296 |
| [G-protein coupled receptor binding](http://amigo.geneontology.org/amigo/term/GO:0001664) | 3 out of 12 genes, 25.0% | 8 out of 258 genes, 3.1% | 0.00380682 |
| [chemokine activity](http://amigo.geneontology.org/amigo/term/GO:0008009) | 2 out of 12 genes, 16.7% | 3 out of 258 genes, 1.2% | 0.005816781 |
| [chemokine receptor binding](http://amigo.geneontology.org/amigo/term/GO:0042379) | 2 out of 12 genes, 16.7% | 3 out of 258 genes, 1.2% | 0.005816781 |
| [phospholipid binding](http://amigo.geneontology.org/amigo/term/GO:0005543) | 4 out of 12 genes, 33.3% | 19 out of 258 genes, 7.4% | 0.007211901 |
| [sterol transporter activity](http://amigo.geneontology.org/amigo/term/GO:0015248) | 3 out of 12 genes, 25.0% | 10 out of 258 genes, 3.9% | 0.007728403 |
| [cholesterol transporter activity](http://amigo.geneontology.org/amigo/term/GO:0017127) | 3 out of 12 genes, 25.0% | 10 out of 258 genes, 3.9% | 0.007728403 |
| [steroid binding](http://amigo.geneontology.org/amigo/term/GO:0005496) | 3 out of 12 genes, 25.0% | 11 out of 258 genes, 4.3% | 0.01034281 |
| [substrate-specific transporter activity](http://amigo.geneontology.org/amigo/term/GO:0022892) | 4 out of 12 genes, 33.3% | 21 out of 258 genes, 8.1% | 0.01056354 |
| [high-density lipoprotein particle binding](http://amigo.geneontology.org/amigo/term/GO:0008035) | 2 out of 12 genes, 16.7% | 4 out of 258 genes, 1.6% | 0.01133074 |
| [receptor binding](http://amigo.geneontology.org/amigo/term/GO:0005102) | 7 out of 12 genes, 58.3% | 64 out of 258 genes, 24.8% | 0.01170754 |
| [apolipoprotein receptor binding](http://amigo.geneontology.org/amigo/term/GO:0034190) | 2 out of 12 genes, 16.7% | 5 out of 258 genes, 1.9% | 0.01839324 |
| [high-density lipoprotein particle receptor binding](http://amigo.geneontology.org/amigo/term/GO:0070653) | 2 out of 12 genes, 16.7% | 5 out of 258 genes, 1.9% | 0.01839324 |
| [transporter activity](http://amigo.geneontology.org/amigo/term/GO:0005215) | 4 out of 12 genes, 33.3% | 25 out of 258 genes, 9.7% | 0.0200775 |
| [lipid transporter activity](http://amigo.geneontology.org/amigo/term/GO:0005319) | 3 out of 12 genes, 25.0% | 14 out of 258 genes, 5.4% | 0.02103356 |
| [alcohol binding](http://amigo.geneontology.org/amigo/term/GO:0043178) | 3 out of 12 genes, 25.0% | 15 out of 258 genes, 5.8% | 0.02558678 |
| [identical protein binding](http://amigo.geneontology.org/amigo/term/GO:0042802) | 4 out of 12 genes, 33.3% | 27 out of 258 genes, 10.5% | 0.02640538 |
| [cytokine receptor binding](http://amigo.geneontology.org/amigo/term/GO:0005126) | 2 out of 12 genes, 16.7% | 6 out of 258 genes, 2.3% | 0.02687242 |
| [glycoprotein binding](http://amigo.geneontology.org/amigo/term/GO:0001948) | 2 out of 12 genes, 16.7% | 7 out of 258 genes, 2.7% | 0.03664367 |
| [phosphatidylcholine binding](http://amigo.geneontology.org/amigo/term/GO:0031210) | 2 out of 12 genes, 16.7% | 8 out of 258 genes, 3.1% | 0.04758928 |
| [carboxylic acid binding](http://amigo.geneontology.org/amigo/term/GO:0031406) | 2 out of 12 genes, 16.7% | 8 out of 258 genes, 3.1% | 0.04758928 |
| [quaternary ammonium group binding](http://amigo.geneontology.org/amigo/term/GO:0050997) | 2 out of 12 genes, 16.7% | 8 out of 258 genes, 3.1% | 0.04758928 |
| [phosphatidylcholine-sterol O-acyltransferase activator activity](http://amigo.geneontology.org/amigo/term/GO:0060228) | 2 out of 12 genes, 16.7% | 8 out of 258 genes, 3.1% | 0.04758928 |
| [lipid binding](http://amigo.geneontology.org/amigo/term/GO:0008289) | 4 out of 12 genes, 33.3% | 34 out of 258 genes, 13.2% | 0.05777927 |
| [lipoprotein particle receptor binding](http://amigo.geneontology.org/amigo/term/GO:0070325) | 2 out of 12 genes, 16.7% | 10 out of 258 genes, 3.9% | 0.07256563 |
| [carbohydrate derivative binding](http://amigo.geneontology.org/amigo/term/GO:0097367) | 3 out of 12 genes, 25.0% | 23 out of 258 genes, 8.9% | 0.08004626 |
| [cell surface binding](http://amigo.geneontology.org/amigo/term/GO:0043498) | 3 out of 12 genes, 25.0% | 24 out of 258 genes, 9.3% | 0.08900711 |
| [small molecule binding](http://amigo.geneontology.org/amigo/term/GO:0036094) | 3 out of 12 genes, 25.0% | 30 out of 258 genes, 11.6% | 0.1513926 |
| [eukaryotic cell surface binding](http://amigo.geneontology.org/amigo/term/GO:0043499) | 2 out of 12 genes, 16.7% | 17 out of 258 genes, 6.6% | 0.1825836 |
| [anion binding](http://amigo.geneontology.org/amigo/term/GO:0043168) | 3 out of 12 genes, 25.0% | 35 out of 258 genes, 13.6% | 0.2126149 |
| [enzyme activator activity](http://amigo.geneontology.org/amigo/term/GO:0008047) | 2 out of 12 genes, 16.7% | 19 out of 258 genes, 7.4% | 0.2179158 |
| [heparin binding](http://amigo.geneontology.org/amigo/term/GO:0008201) | 2 out of 12 genes, 16.7% | 20 out of 258 genes, 7.8% | 0.2359136 |
| [protein homodimerization activity](http://amigo.geneontology.org/amigo/term/GO:0042803) | 2 out of 12 genes, 16.7% | 20 out of 258 genes, 7.8% | 0.2359136 |
| [sulfur compound binding](http://amigo.geneontology.org/amigo/term/GO:1901681) | 2 out of 12 genes, 16.7% | 21 out of 258 genes, 8.1% | 0.2540626 |
| [organic cyclic compound binding](http://amigo.geneontology.org/amigo/term/GO:0097159) | 3 out of 12 genes, 25.0% | 39 out of 258 genes, 15.1% | 0.2658135 |
| [glycosaminoglycan binding](http://amigo.geneontology.org/amigo/term/GO:0005539) | 2 out of 12 genes, 16.7% | 22 out of 258 genes, 8.5% | 0.2723149 |
| [protein dimerization activity](http://amigo.geneontology.org/amigo/term/GO:0046983) | 2 out of 12 genes, 16.7% | 22 out of 258 genes, 8.5% | 0.2723149 |
| [protein binding](http://amigo.geneontology.org/amigo/term/GO:0005515) | 9 out of 12 genes, 75.0% | 162 out of 258 genes, 62.8% | 0.2841187 |
| [ion binding](http://amigo.geneontology.org/amigo/term/GO:0043167) | 5 out of 12 genes, 41.7% | 86 out of 258 genes, 33.3% | 0.366552 |
| [enzyme regulator activity](http://amigo.geneontology.org/amigo/term/GO:0030234) | 3 out of 12 genes, 25.0% | 50 out of 258 genes, 19.4% | 0.4205166 |
| [enzyme inhibitor activity](http://amigo.geneontology.org/amigo/term/GO:0004857) | 2 out of 12 genes, 16.7% | 33 out of 258 genes, 12.8% | 0.4693157 |

C. GO analysis between VSD-PAH and VSD – biological process

| **Gene Ontology term** | **Cluster frequency** | **Protein frequency of use** | **P-value** |
| --- | --- | --- | --- |
| [regulation of biological quality](http://amigo.geneontology.org/amigo/term/GO:0065008) | 12 out of 13 genes, 92.3% | 100 out of 273 genes, 36.6% | 3.359442e-05 |
| [localization](http://amigo.geneontology.org/amigo/term/GO:0051179) | 11 out of 13 genes, 84.6% | 94 out of 273 genes, 34.4% | 0.0002114234 |
| [regulation of cytokine production](http://amigo.geneontology.org/amigo/term/GO:0001817) | 6 out of 13 genes, 46.2% | 23 out of 273 genes, 8.4% | 0.0002144431 |
| [regulation of lipid transport](http://amigo.geneontology.org/amigo/term/GO:0032368) | 5 out of 13 genes, 38.5% | 15 out of 273 genes, 5.5% | 0.0002459594 |
| [single-multicellular organism process](http://amigo.geneontology.org/amigo/term/GO:0044707) | 12 out of 13 genes, 92.3% | 118 out of 273 genes, 43.2% | 0.0002476415 |
| [regulation of body fluid levels](http://amigo.geneontology.org/amigo/term/GO:0050878) | 9 out of 13 genes, 69.2% | 63 out of 273 genes, 23.1% | 0.0003686381 |
| [multicellular organismal process](http://amigo.geneontology.org/amigo/term/GO:0032501) | 12 out of 13 genes, 92.3% | 123 out of 273 genes, 45.1% | 0.0004056856 |
| [platelet activation](http://amigo.geneontology.org/amigo/term/GO:0030168) | 7 out of 13 genes, 53.8% | 40 out of 273 genes, 14.7% | 0.0007719108 |
| [response to hexose stimulus](http://amigo.geneontology.org/amigo/term/GO:0009746) | 3 out of 13 genes, 23.1% | 5 out of 273 genes, 1.8% | 0.0008059992 |
| [response to glucose stimulus](http://amigo.geneontology.org/amigo/term/GO:0009749) | 3 out of 13 genes, 23.1% | 5 out of 273 genes, 1.8% | 0.0008059992 |
| [response to monosaccharide stimulus](http://amigo.geneontology.org/amigo/term/GO:0034284) | 3 out of 13 genes, 23.1% | 5 out of 273 genes, 1.8% | 0.0008059992 |
| [negative regulation of secretion](http://amigo.geneontology.org/amigo/term/GO:0051048) | 3 out of 13 genes, 23.1% | 5 out of 273 genes, 1.8% | 0.0008059992 |
| [regulation of cell adhesion](http://amigo.geneontology.org/amigo/term/GO:0030155) | 5 out of 13 genes, 38.5% | 19 out of 273 genes, 7.0% | 0.0008585887 |
| [secretion](http://amigo.geneontology.org/amigo/term/GO:0046903) | 7 out of 13 genes, 53.8% | 43 out of 273 genes, 15.8% | 0.001248252 |
| [negative regulation of cell adhesion](http://amigo.geneontology.org/amigo/term/GO:0007162) | 4 out of 13 genes, 30.8% | 12 out of 273 genes, 4.4% | 0.001256468 |
| [negative regulation of transport](http://amigo.geneontology.org/amigo/term/GO:0051051) | 4 out of 13 genes, 30.8% | 12 out of 273 genes, 4.4% | 0.001256468 |
| [regulation of tumor necrosis factor production](http://amigo.geneontology.org/amigo/term/GO:0032680) | 3 out of 13 genes, 23.1% | 6 out of 273 genes, 2.2% | 0.001567128 |
| [cellular localization](http://amigo.geneontology.org/amigo/term/GO:0051641) | 7 out of 13 genes, 53.8% | 45 out of 273 genes, 16.5% | 0.001680915 |
| [positive regulation of cytokine production](http://amigo.geneontology.org/amigo/term/GO:0001819) | 4 out of 13 genes, 30.8% | 13 out of 273 genes, 4.8% | 0.001765574 |
| [blood coagulation](http://amigo.geneontology.org/amigo/term/GO:0007596) | 8 out of 13 genes, 61.5% | 60 out of 273 genes, 22.0% | 0.001828259 |
| [hemostasis](http://amigo.geneontology.org/amigo/term/GO:0007599) | 8 out of 13 genes, 61.5% | 60 out of 273 genes, 22.0% | 0.001828259 |
| [coagulation](http://amigo.geneontology.org/amigo/term/GO:0050817) | 8 out of 13 genes, 61.5% | 60 out of 273 genes, 22.0% | 0.001828259 |
| [negative regulation of macrophage derived foam cell differentiation](http://amigo.geneontology.org/amigo/term/GO:0010745) | 2 out of 13 genes, 15.4% | 2 out of 273 genes, 0.7% | 0.002100840 |
| [regulation of eukaryotic cell surface binding](http://amigo.geneontology.org/amigo/term/GO:2000460) | 2 out of 13 genes, 15.4% | 2 out of 273 genes, 0.7% | 0.002100840 |
| [cell activation](http://amigo.geneontology.org/amigo/term/GO:0001775) | 7 out of 13 genes, 53.8% | 47 out of 273 genes, 17.2% | 0.002226958 |
| [regulation of sterol transport](http://amigo.geneontology.org/amigo/term/GO:0032371) | 4 out of 13 genes, 30.8% | 14 out of 273 genes, 5.1% | 0.002404503 |
| [regulation of cholesterol transport](http://amigo.geneontology.org/amigo/term/GO:0032374) | 4 out of 13 genes, 30.8% | 14 out of 273 genes, 5.1% | 0.002404503 |
| [regulation of secretion](http://amigo.geneontology.org/amigo/term/GO:0051046) | 4 out of 13 genes, 30.8% | 14 out of 273 genes, 5.1% | 0.002404503 |
| [response to wounding](http://amigo.geneontology.org/amigo/term/GO:0009611) | 9 out of 13 genes, 69.2% | 79 out of 273 genes, 28.9% | 0.002433792 |
| [single-organism transport](http://amigo.geneontology.org/amigo/term/GO:0044765) | 9 out of 13 genes, 69.2% | 79 out of 273 genes, 28.9% | 0.002433792 |
| [vesicle-mediated transport](http://amigo.geneontology.org/amigo/term/GO:0016192) | 7 out of 13 genes, 53.8% | 48 out of 273 genes, 17.6% | 0.002548817 |
| [response to carbohydrate stimulus](http://amigo.geneontology.org/amigo/term/GO:0009743) | 3 out of 13 genes, 23.1% | 7 out of 273 genes, 2.6% | 0.002666062 |
| [negative regulation of cell-cell adhesion](http://amigo.geneontology.org/amigo/term/GO:0022408) | 3 out of 13 genes, 23.1% | 7 out of 273 genes, 2.6% | 0.002666062 |
| [regulation of cytokine secretion](http://amigo.geneontology.org/amigo/term/GO:0050707) | 3 out of 13 genes, 23.1% | 7 out of 273 genes, 2.6% | 0.002666062 |
| [negative regulation of protein transport](http://amigo.geneontology.org/amigo/term/GO:0051224) | 3 out of 13 genes, 23.1% | 7 out of 273 genes, 2.6% | 0.002666062 |
| [wound healing](http://amigo.geneontology.org/amigo/term/GO:0042060) | 8 out of 13 genes, 61.5% | 64 out of 273 genes, 23.4% | 0.002915508 |
| [regulation of protein localization](http://amigo.geneontology.org/amigo/term/GO:0032880) | 4 out of 13 genes, 30.8% | 15 out of 273 genes, 5.5% | 0.003189429 |
| [negative regulation of cellular component organization](http://amigo.geneontology.org/amigo/term/GO:0051129) | 4 out of 13 genes, 30.8% | 15 out of 273 genes, 5.5% | 0.003189429 |
| [regulation of protein transport](http://amigo.geneontology.org/amigo/term/GO:0051223) | 4 out of 13 genes, 30.8% | 15 out of 273 genes, 5.5% | 0.003189429 |
| [regulation of establishment of protein localization](http://amigo.geneontology.org/amigo/term/GO:0070201) | 4 out of 13 genes, 30.8% | 15 out of 273 genes, 5.5% | 0.003189429 |
| [platelet degranulation](http://amigo.geneontology.org/amigo/term/GO:0002576) | 6 out of 13 genes, 46.2% | 37 out of 273 genes, 13.6% | 0.003513904 |
| [exocytosis](http://amigo.geneontology.org/amigo/term/GO:0006887) | 6 out of 13 genes, 46.2% | 37 out of 273 genes, 13.6% | 0.003513904 |
| [transport](http://amigo.geneontology.org/amigo/term/GO:0006810) | 9 out of 13 genes, 69.2% | 83 out of 273 genes, 30.4% | 0.0036178 |
| [establishment of localization](http://amigo.geneontology.org/amigo/term/GO:0051234) | 9 out of 13 genes, 69.2% | 83 out of 273 genes, 30.4% | 0.0036178 |
| [secretion by cell](http://amigo.geneontology.org/amigo/term/GO:0032940) | 6 out of 13 genes, 46.2% | 38 out of 273 genes, 13.9% | 0.004070378 |
| [regulation of plasma lipoprotein particle levels](http://amigo.geneontology.org/amigo/term/GO:0097006) | 4 out of 13 genes, 30.8% | 16 out of 273 genes, 5.9% | 0.004136359 |
| [regulation of macrophage derived foam cell differentiation](http://amigo.geneontology.org/amigo/term/GO:0010743) | 3 out of 13 genes, 23.1% | 8 out of 273 genes, 2.9% | 0.004146731 |
| [regulation of cell-cell adhesion](http://amigo.geneontology.org/amigo/term/GO:0022407) | 3 out of 13 genes, 23.1% | 8 out of 273 genes, 2.9% | 0.004146731 |
| [regulation of transport](http://amigo.geneontology.org/amigo/term/GO:0051049) | 6 out of 13 genes, 46.2% | 39 out of 273 genes, 14.3% | 0.004692079 |
| [regulation of protein secretion](http://amigo.geneontology.org/amigo/term/GO:0050708) | 3 out of 13 genes, 23.1% | 9 out of 273 genes, 3.3% | 0.006046457 |
| [positive regulation of transferase activity](http://amigo.geneontology.org/amigo/term/GO:0051347) | 3 out of 13 genes, 23.1% | 9 out of 273 genes, 3.3% | 0.006046457 |
| [regulation of gastrulation](http://amigo.geneontology.org/amigo/term/GO:0010470) | 2 out of 13 genes, 15.4% | 3 out of 273 genes, 1.1% | 0.006131973 |
| [cytokine-mediated signaling pathway](http://amigo.geneontology.org/amigo/term/GO:0019221) | 2 out of 13 genes, 15.4% | 3 out of 273 genes, 1.1% | 0.006131973 |
| [regulation of heterotypic cell-cell adhesion](http://amigo.geneontology.org/amigo/term/GO:0034114) | 2 out of 13 genes, 15.4% | 3 out of 273 genes, 1.1% | 0.006131973 |
| [negative regulation of heterotypic cell-cell adhesion](http://amigo.geneontology.org/amigo/term/GO:0034115) | 2 out of 13 genes, 15.4% | 3 out of 273 genes, 1.1% | 0.006131973 |
| [regulation of embryonic development](http://amigo.geneontology.org/amigo/term/GO:0045995) | 2 out of 13 genes, 15.4% | 3 out of 273 genes, 1.1% | 0.006131973 |
| [phospholipid homeostasis](http://amigo.geneontology.org/amigo/term/GO:0055091) | 2 out of 13 genes, 15.4% | 3 out of 273 genes, 1.1% | 0.006131973 |
| [regulation of cell-cell adhesion involved in gastrulation](http://amigo.geneontology.org/amigo/term/GO:0070587) | 2 out of 13 genes, 15.4% | 3 out of 273 genes, 1.1% | 0.006131973 |
| [positive regulation of multicellular organismal process](http://amigo.geneontology.org/amigo/term/GO:0051240) | 5 out of 13 genes, 38.5% | 30 out of 273 genes, 11.0% | 0.007870158 |
| [establishment of localization in cell](http://amigo.geneontology.org/amigo/term/GO:0051649) | 6 out of 13 genes, 46.2% | 43 out of 273 genes, 15.8% | 0.007930124 |
| [cellular biogenic amine metabolic process](http://amigo.geneontology.org/amigo/term/GO:0006576) | 3 out of 13 genes, 23.1% | 10 out of 273 genes, 3.7% | 0.008396443 |
| [glycerophospholipid metabolic process](http://amigo.geneontology.org/amigo/term/GO:0006650) | 3 out of 13 genes, 23.1% | 10 out of 273 genes, 3.7% | 0.008396443 |
| [amine metabolic process](http://amigo.geneontology.org/amigo/term/GO:0009308) | 3 out of 13 genes, 23.1% | 10 out of 273 genes, 3.7% | 0.008396443 |
| [high-density lipoprotein particle remodeling](http://amigo.geneontology.org/amigo/term/GO:0034375) | 3 out of 13 genes, 23.1% | 10 out of 273 genes, 3.7% | 0.008396443 |
| [ethanolamine-containing compound metabolic process](http://amigo.geneontology.org/amigo/term/GO:0042439) | 3 out of 13 genes, 23.1% | 10 out of 273 genes, 3.7% | 0.008396443 |
| [cellular amine metabolic process](http://amigo.geneontology.org/amigo/term/GO:0044106) | 3 out of 13 genes, 23.1% | 10 out of 273 genes, 3.7% | 0.008396443 |
| [phosphatidylcholine metabolic process](http://amigo.geneontology.org/amigo/term/GO:0046470) | 3 out of 13 genes, 23.1% | 10 out of 273 genes, 3.7% | 0.008396443 |
| [negative regulation of developmental process](http://amigo.geneontology.org/amigo/term/GO:0051093) | 4 out of 13 genes, 30.8% | 20 out of 273 genes, 7.3% | 0.009851123 |
| [regulation of cellular localization](http://amigo.geneontology.org/amigo/term/GO:0060341) | 4 out of 13 genes, 30.8% | 20 out of 273 genes, 7.3% | 0.009851123 |
| [posttranscriptional regulation of gene expression](http://amigo.geneontology.org/amigo/term/GO:0010608) | 3 out of 13 genes, 23.1% | 11 out of 273 genes, 4.0% | 0.01122223 |
| [organophosphate ester transport](http://amigo.geneontology.org/amigo/term/GO:0015748) | 3 out of 13 genes, 23.1% | 11 out of 273 genes, 4.0% | 0.01122223 |
| [phospholipid transport](http://amigo.geneontology.org/amigo/term/GO:0015914) | 3 out of 13 genes, 23.1% | 11 out of 273 genes, 4.0% | 0.01122223 |
| [plasma lipoprotein particle clearance](http://amigo.geneontology.org/amigo/term/GO:0034381) | 3 out of 13 genes, 23.1% | 11 out of 273 genes, 4.0% | 0.01122223 |
| [negative regulation of cell differentiation](http://amigo.geneontology.org/amigo/term/GO:0045596) | 3 out of 13 genes, 23.1% | 11 out of 273 genes, 4.0% | 0.01122223 |
| [regulation of cytokine secretion involved in immune response](http://amigo.geneontology.org/amigo/term/GO:0002739) | 2 out of 13 genes, 15.4% | 4 out of 273 genes, 1.5% | 0.01193233 |
| [negative regulation of cytokine secretion involved in immune response](http://amigo.geneontology.org/amigo/term/GO:0002740) | 2 out of 13 genes, 15.4% | 4 out of 273 genes, 1.5% | 0.01193233 |
| [regulation of myeloid leukocyte differentiation](http://amigo.geneontology.org/amigo/term/GO:0002761) | 2 out of 13 genes, 15.4% | 4 out of 273 genes, 1.5% | 0.01193233 |
| [phosphatidylcholine biosynthetic process](http://amigo.geneontology.org/amigo/term/GO:0006656) | 2 out of 13 genes, 15.4% | 4 out of 273 genes, 1.5% | 0.01193233 |
| [phospholipid biosynthetic process](http://amigo.geneontology.org/amigo/term/GO:0008654) | 2 out of 13 genes, 15.4% | 4 out of 273 genes, 1.5% | 0.01193233 |
| [regulation of tumor necrosis factor-mediated signaling pathway](http://amigo.geneontology.org/amigo/term/GO:0010803) | 2 out of 13 genes, 15.4% | 4 out of 273 genes, 1.5% | 0.01193233 |
| [negative regulation of tumor necrosis factor-mediated signaling pathway](http://amigo.geneontology.org/amigo/term/GO:0010804) | 2 out of 13 genes, 15.4% | 4 out of 273 genes, 1.5% | 0.01193233 |
| [negative regulation of cell-substrate adhesion](http://amigo.geneontology.org/amigo/term/GO:0010812) | 2 out of 13 genes, 15.4% | 4 out of 273 genes, 1.5% | 0.01193233 |
| [protein oxidation](http://amigo.geneontology.org/amigo/term/GO:0018158) | 2 out of 13 genes, 15.4% | 4 out of 273 genes, 1.5% | 0.01193233 |
| [peptidyl-methionine modification](http://amigo.geneontology.org/amigo/term/GO:0018206) | 2 out of 13 genes, 15.4% | 4 out of 273 genes, 1.5% | 0.01193233 |
| [regulation of interleukin-8 production](http://amigo.geneontology.org/amigo/term/GO:0032677) | 2 out of 13 genes, 15.4% | 4 out of 273 genes, 1.5% | 0.01193233 |
| [regulation of transmembrane transport](http://amigo.geneontology.org/amigo/term/GO:0034762) | 2 out of 13 genes, 15.4% | 4 out of 273 genes, 1.5% | 0.01193233 |
| [negative regulation of transmembrane transport](http://amigo.geneontology.org/amigo/term/GO:0034763) | 2 out of 13 genes, 15.4% | 4 out of 273 genes, 1.5% | 0.01193233 |
| [regulation of cytokine biosynthetic process](http://amigo.geneontology.org/amigo/term/GO:0042035) | 2 out of 13 genes, 15.4% | 4 out of 273 genes, 1.5% | 0.01193233 |
| [positive regulation of cytokine biosynthetic process](http://amigo.geneontology.org/amigo/term/GO:0042108) | 2 out of 13 genes, 15.4% | 4 out of 273 genes, 1.5% | 0.01193233 |
| [regulation of myeloid cell differentiation](http://amigo.geneontology.org/amigo/term/GO:0045637) | 2 out of 13 genes, 15.4% | 4 out of 273 genes, 1.5% | 0.01193233 |
| [negative regulation of myeloid cell differentiation](http://amigo.geneontology.org/amigo/term/GO:0045638) | 2 out of 13 genes, 15.4% | 4 out of 273 genes, 1.5% | 0.01193233 |
| [regulation of macrophage differentiation](http://amigo.geneontology.org/amigo/term/GO:0045649) | 2 out of 13 genes, 15.4% | 4 out of 273 genes, 1.5% | 0.01193233 |
| [glycerophospholipid biosynthetic process](http://amigo.geneontology.org/amigo/term/GO:0046474) | 2 out of 13 genes, 15.4% | 4 out of 273 genes, 1.5% | 0.01193233 |
| [negative regulation of protein secretion](http://amigo.geneontology.org/amigo/term/GO:0050709) | 2 out of 13 genes, 15.4% | 4 out of 273 genes, 1.5% | 0.01193233 |
| [negative regulation of cytokine secretion](http://amigo.geneontology.org/amigo/term/GO:0050710) | 2 out of 13 genes, 15.4% | 4 out of 273 genes, 1.5% | 0.01193233 |
| [chemical homeostasis](http://amigo.geneontology.org/amigo/term/GO:0048878) | 5 out of 13 genes, 38.5% | 33 out of 273 genes, 12.1% | 0.01209279 |
| [negative regulation of cellular process](http://amigo.geneontology.org/amigo/term/GO:0048523) | 7 out of 13 genes, 53.8% | 63 out of 273 genes, 23.1% | 0.01351353 |
| [regulation of multicellular organismal process](http://amigo.geneontology.org/amigo/term/GO:0051239) | 7 out of 13 genes, 53.8% | 63 out of 273 genes, 23.1% | 0.01351353 |
| [regulation of anatomical structure morphogenesis](http://amigo.geneontology.org/amigo/term/GO:0022603) | 4 out of 13 genes, 30.8% | 22 out of 273 genes, 8.1% | 0.01406365 |
| [negative regulation of cytokine production](http://amigo.geneontology.org/amigo/term/GO:0001818) | 3 out of 13 genes, 23.1% | 12 out of 273 genes, 4.4% | 0.01454416 |
| [reverse cholesterol transport](http://amigo.geneontology.org/amigo/term/GO:0043691) | 3 out of 13 genes, 23.1% | 12 out of 273 genes, 4.4% | 0.01454416 |
| [regulation of system process](http://amigo.geneontology.org/amigo/term/GO:0044057) | 3 out of 13 genes, 23.1% | 12 out of 273 genes, 4.4% | 0.01454416 |
| [negative regulation of inflammatory response](http://amigo.geneontology.org/amigo/term/GO:0050728) | 3 out of 13 genes, 23.1% | 12 out of 273 genes, 4.4% | 0.01454416 |
| [negative regulation of biological process](http://amigo.geneontology.org/amigo/term/GO:0048519) | 8 out of 13 genes, 61.5% | 82 out of 273 genes, 30.0% | 0.01602548 |
| [regulation of localization](http://amigo.geneontology.org/amigo/term/GO:0032879) | 6 out of 13 genes, 46.2% | 50 out of 273 genes, 18.3% | 0.01729121 |
| [negative regulation of multicellular organismal process](http://amigo.geneontology.org/amigo/term/GO:0051241) | 5 out of 13 genes, 38.5% | 36 out of 273 genes, 13.2% | 0.01771150 |
| [cholesterol metabolic process](http://amigo.geneontology.org/amigo/term/GO:0008203) | 3 out of 13 genes, 23.1% | 13 out of 273 genes, 4.8% | 0.01837773 |
| [sterol metabolic process](http://amigo.geneontology.org/amigo/term/GO:0016125) | 3 out of 13 genes, 23.1% | 13 out of 273 genes, 4.8% | 0.01837773 |
| [negative regulation of response to external stimulus](http://amigo.geneontology.org/amigo/term/GO:0032102) | 3 out of 13 genes, 23.1% | 13 out of 273 genes, 4.8% | 0.01837773 |
| [oxidation-reduction process](http://amigo.geneontology.org/amigo/term/GO:0055114) | 3 out of 13 genes, 23.1% | 13 out of 273 genes, 4.8% | 0.01837773 |
| [negative regulation of cytokine-mediated signaling pathway](http://amigo.geneontology.org/amigo/term/GO:0001960) | 2 out of 13 genes, 15.4% | 5 out of 273 genes, 1.8% | 0.01934988 |
| [regulation of cytokine production involved in immune response](http://amigo.geneontology.org/amigo/term/GO:0002718) | 2 out of 13 genes, 15.4% | 5 out of 273 genes, 1.8% | 0.01934988 |
| [regulation of cholesterol efflux](http://amigo.geneontology.org/amigo/term/GO:0010874) | 2 out of 13 genes, 15.4% | 5 out of 273 genes, 1.8% | 0.01934988 |
| [negative regulation of very-low-density lipoprotein particle remodeling](http://amigo.geneontology.org/amigo/term/GO:0010903) | 2 out of 13 genes, 15.4% | 5 out of 273 genes, 1.8% | 0.01934988 |
| [high-density lipoprotein particle assembly](http://amigo.geneontology.org/amigo/term/GO:0034380) | 2 out of 13 genes, 15.4% | 5 out of 273 genes, 1.8% | 0.01934988 |
| [glycerolipid biosynthetic process](http://amigo.geneontology.org/amigo/term/GO:0045017) | 2 out of 13 genes, 15.4% | 5 out of 273 genes, 1.8% | 0.01934988 |
| [regulation of interleukin-1 secretion](http://amigo.geneontology.org/amigo/term/GO:0050704) | 2 out of 13 genes, 15.4% | 5 out of 273 genes, 1.8% | 0.01934988 |
| [negative regulation of response to cytokine stimulus](http://amigo.geneontology.org/amigo/term/GO:0060761) | 2 out of 13 genes, 15.4% | 5 out of 273 genes, 1.8% | 0.01934988 |
| [homeostatic process](http://amigo.geneontology.org/amigo/term/GO:0042592) | 5 out of 13 genes, 38.5% | 37 out of 273 genes, 13.6% | 0.01992988 |
| [regulation of developmental process](http://amigo.geneontology.org/amigo/term/GO:0050793) | 5 out of 13 genes, 38.5% | 37 out of 273 genes, 13.6% | 0.01992988 |
| [cellular lipid metabolic process](http://amigo.geneontology.org/amigo/term/GO:0044255) | 4 out of 13 genes, 30.8% | 25 out of 273 genes, 9.2% | 0.02235379 |
| [chemotaxis](http://amigo.geneontology.org/amigo/term/GO:0006935) | 3 out of 13 genes, 23.1% | 14 out of 273 genes, 5.1% | 0.02273406 |
| [taxis](http://amigo.geneontology.org/amigo/term/GO:0042330) | 3 out of 13 genes, 23.1% | 14 out of 273 genes, 5.1% | 0.02273406 |
| [response to drug](http://amigo.geneontology.org/amigo/term/GO:0042493) | 3 out of 13 genes, 23.1% | 14 out of 273 genes, 5.1% | 0.02273406 |
| [cholesterol homeostasis](http://amigo.geneontology.org/amigo/term/GO:0042632) | 3 out of 13 genes, 23.1% | 14 out of 273 genes, 5.1% | 0.02273406 |
| [regulation of transferase activity](http://amigo.geneontology.org/amigo/term/GO:0051338) | 3 out of 13 genes, 23.1% | 14 out of 273 genes, 5.1% | 0.02273406 |
| [sterol homeostasis](http://amigo.geneontology.org/amigo/term/GO:0055092) | 3 out of 13 genes, 23.1% | 14 out of 273 genes, 5.1% | 0.02273406 |
| [positive regulation of cellular process](http://amigo.geneontology.org/amigo/term/GO:0048522) | 7 out of 13 genes, 53.8% | 69 out of 273 genes, 25.3% | 0.02280627 |
| [phospholipid metabolic process](http://amigo.geneontology.org/amigo/term/GO:0006644) | 3 out of 13 genes, 23.1% | 15 out of 273 genes, 5.5% | 0.02762022 |
| [organic hydroxy compound transport](http://amigo.geneontology.org/amigo/term/GO:0015850) | 3 out of 13 genes, 23.1% | 15 out of 273 genes, 5.5% | 0.02762022 |
| [sterol transport](http://amigo.geneontology.org/amigo/term/GO:0015918) | 3 out of 13 genes, 23.1% | 15 out of 273 genes, 5.5% | 0.02762022 |
| [cholesterol transport](http://amigo.geneontology.org/amigo/term/GO:0030301) | 3 out of 13 genes, 23.1% | 15 out of 273 genes, 5.5% | 0.02762022 |
| [negative regulation of defense response](http://amigo.geneontology.org/amigo/term/GO:0031348) | 3 out of 13 genes, 23.1% | 15 out of 273 genes, 5.5% | 0.02762022 |
| [macromolecular complex remodeling](http://amigo.geneontology.org/amigo/term/GO:0034367) | 3 out of 13 genes, 23.1% | 15 out of 273 genes, 5.5% | 0.02762022 |
| [protein-lipid complex remodeling](http://amigo.geneontology.org/amigo/term/GO:0034368) | 3 out of 13 genes, 23.1% | 15 out of 273 genes, 5.5% | 0.02762022 |
| [plasma lipoprotein particle remodeling](http://amigo.geneontology.org/amigo/term/GO:0034369) | 3 out of 13 genes, 23.1% | 15 out of 273 genes, 5.5% | 0.02762022 |
| [positive regulation of lipid metabolic process](http://amigo.geneontology.org/amigo/term/GO:0045834) | 3 out of 13 genes, 23.1% | 15 out of 273 genes, 5.5% | 0.02762022 |
| [response to steroid hormone stimulus](http://amigo.geneontology.org/amigo/term/GO:0048545) | 3 out of 13 genes, 23.1% | 15 out of 273 genes, 5.5% | 0.02762022 |
| [positive regulation of developmental process](http://amigo.geneontology.org/amigo/term/GO:0051094) | 3 out of 13 genes, 23.1% | 15 out of 273 genes, 5.5% | 0.02762022 |
| [protein-lipid complex subunit organization](http://amigo.geneontology.org/amigo/term/GO:0071825) | 3 out of 13 genes, 23.1% | 15 out of 273 genes, 5.5% | 0.02762022 |
| [plasma lipoprotein particle organization](http://amigo.geneontology.org/amigo/term/GO:0071827) | 3 out of 13 genes, 23.1% | 15 out of 273 genes, 5.5% | 0.02762022 |
| [regulation of cell-substrate adhesion](http://amigo.geneontology.org/amigo/term/GO:0010810) | 2 out of 13 genes, 15.4% | 6 out of 273 genes, 2.2% | 0.02824125 |
| [cellular membrane organization](http://amigo.geneontology.org/amigo/term/GO:0016044) | 2 out of 13 genes, 15.4% | 6 out of 273 genes, 2.2% | 0.02824125 |
| [negative regulation of angiogenesis](http://amigo.geneontology.org/amigo/term/GO:0016525) | 2 out of 13 genes, 15.4% | 6 out of 273 genes, 2.2% | 0.02824125 |
| [regulation of intestinal cholesterol absorption](http://amigo.geneontology.org/amigo/term/GO:0030300) | 2 out of 13 genes, 15.4% | 6 out of 273 genes, 2.2% | 0.02824125 |
| [leukocyte chemotaxis](http://amigo.geneontology.org/amigo/term/GO:0030595) | 2 out of 13 genes, 15.4% | 6 out of 273 genes, 2.2% | 0.02824125 |
| [negative regulation of lipid transport](http://amigo.geneontology.org/amigo/term/GO:0032369) | 2 out of 13 genes, 15.4% | 6 out of 273 genes, 2.2% | 0.02824125 |
| [regulation of interleukin-1 production](http://amigo.geneontology.org/amigo/term/GO:0032652) | 2 out of 13 genes, 15.4% | 6 out of 273 genes, 2.2% | 0.02824125 |
| [high-density lipoprotein particle clearance](http://amigo.geneontology.org/amigo/term/GO:0034384) | 2 out of 13 genes, 15.4% | 6 out of 273 genes, 2.2% | 0.02824125 |
| [regulation of digestive system process](http://amigo.geneontology.org/amigo/term/GO:0044058) | 2 out of 13 genes, 15.4% | 6 out of 273 genes, 2.2% | 0.02824125 |
| [cell chemotaxis](http://amigo.geneontology.org/amigo/term/GO:0060326) | 2 out of 13 genes, 15.4% | 6 out of 273 genes, 2.2% | 0.02824125 |
| [membrane organization](http://amigo.geneontology.org/amigo/term/GO:0061024) | 2 out of 13 genes, 15.4% | 6 out of 273 genes, 2.2% | 0.02824125 |
| [lipid homeostasis](http://amigo.geneontology.org/amigo/term/GO:0055088) | 3 out of 13 genes, 23.1% | 16 out of 273 genes, 5.9% | 0.03303958 |
| [phosphorus metabolic process](http://amigo.geneontology.org/amigo/term/GO:0006793) | 4 out of 13 genes, 30.8% | 28 out of 273 genes, 10.3% | 0.03324088 |
| [phosphate-containing compound metabolic process](http://amigo.geneontology.org/amigo/term/GO:0006796) | 4 out of 13 genes, 30.8% | 28 out of 273 genes, 10.3% | 0.03324088 |
| [regulation of macromolecule biosynthetic process](http://amigo.geneontology.org/amigo/term/GO:0010556) | 4 out of 13 genes, 30.8% | 29 out of 273 genes, 10.6% | 0.03748165 |
| [locomotion](http://amigo.geneontology.org/amigo/term/GO:0040011) | 4 out of 13 genes, 30.8% | 29 out of 273 genes, 10.6% | 0.03748165 |
| [response to hypoxia](http://amigo.geneontology.org/amigo/term/GO:0001666) | 2 out of 13 genes, 15.4% | 7 out of 273 genes, 2.6% | 0.03847132 |
| [regulation of cytokine-mediated signaling pathway](http://amigo.geneontology.org/amigo/term/GO:0001959) | 2 out of 13 genes, 15.4% | 7 out of 273 genes, 2.6% | 0.03847132 |
| [regulation of production of molecular mediator of immune response](http://amigo.geneontology.org/amigo/term/GO:0002700) | 2 out of 13 genes, 15.4% | 7 out of 273 genes, 2.6% | 0.03847132 |
| [negative regulation of macromolecule biosynthetic process](http://amigo.geneontology.org/amigo/term/GO:0010558) | 2 out of 13 genes, 15.4% | 7 out of 273 genes, 2.6% | 0.03847132 |
| [regulation of very-low-density lipoprotein particle remodeling](http://amigo.geneontology.org/amigo/term/GO:0010901) | 2 out of 13 genes, 15.4% | 7 out of 273 genes, 2.6% | 0.03847132 |
| [positive regulation of kinase activity](http://amigo.geneontology.org/amigo/term/GO:0033674) | 2 out of 13 genes, 15.4% | 7 out of 273 genes, 2.6% | 0.03847132 |
| [low-density lipoprotein particle remodeling](http://amigo.geneontology.org/amigo/term/GO:0034374) | 2 out of 13 genes, 15.4% | 7 out of 273 genes, 2.6% | 0.03847132 |
| [response to decreased oxygen levels](http://amigo.geneontology.org/amigo/term/GO:0036293) | 2 out of 13 genes, 15.4% | 7 out of 273 genes, 2.6% | 0.03847132 |
| [positive regulation of protein kinase activity](http://amigo.geneontology.org/amigo/term/GO:0045860) | 2 out of 13 genes, 15.4% | 7 out of 273 genes, 2.6% | 0.03847132 |
| [alcohol biosynthetic process](http://amigo.geneontology.org/amigo/term/GO:0046165) | 2 out of 13 genes, 15.4% | 7 out of 273 genes, 2.6% | 0.03847132 |
| [regulation of binding](http://amigo.geneontology.org/amigo/term/GO:0051098) | 2 out of 13 genes, 15.4% | 7 out of 273 genes, 2.6% | 0.03847132 |
| [regulation of response to cytokine stimulus](http://amigo.geneontology.org/amigo/term/GO:0060759) | 2 out of 13 genes, 15.4% | 7 out of 273 genes, 2.6% | 0.03847132 |
| [response to oxygen levels](http://amigo.geneontology.org/amigo/term/GO:0070482) | 2 out of 13 genes, 15.4% | 7 out of 273 genes, 2.6% | 0.03847132 |
| [positive regulation of protein serine/threonine kinase activity](http://amigo.geneontology.org/amigo/term/GO:0071902) | 2 out of 13 genes, 15.4% | 7 out of 273 genes, 2.6% | 0.03847132 |
| [organic hydroxy compound biosynthetic process](http://amigo.geneontology.org/amigo/term/GO:1901617) | 2 out of 13 genes, 15.4% | 7 out of 273 genes, 2.6% | 0.03847132 |
| [cell surface receptor signaling pathway](http://amigo.geneontology.org/amigo/term/GO:0007166) | 3 out of 13 genes, 23.1% | 17 out of 273 genes, 6.2% | 0.03899213 |
| [negative regulation of cell proliferation](http://amigo.geneontology.org/amigo/term/GO:0008285) | 3 out of 13 genes, 23.1% | 17 out of 273 genes, 6.2% | 0.03899213 |
| [lipoprotein metabolic process](http://amigo.geneontology.org/amigo/term/GO:0042157) | 3 out of 13 genes, 23.1% | 17 out of 273 genes, 6.2% | 0.03899213 |
| [regulation of multicellular organismal development](http://amigo.geneontology.org/amigo/term/GO:2000026) | 4 out of 13 genes, 30.8% | 30 out of 273 genes, 11.0% | 0.04203726 |
| [regulation of cellular process](http://amigo.geneontology.org/amigo/term/GO:0050794) | 9 out of 13 genes, 69.2% | 116 out of 273 genes, 42.5% | 0.04413444 |
| [positive regulation of metabolic process](http://amigo.geneontology.org/amigo/term/GO:0009893) | 5 out of 13 genes, 38.5% | 45 out of 273 genes, 16.5% | 0.04484172 |
| [steroid metabolic process](http://amigo.geneontology.org/amigo/term/GO:0008202) | 3 out of 13 genes, 23.1% | 18 out of 273 genes, 6.6% | 0.04547483 |
| [cellular process](http://amigo.geneontology.org/amigo/term/GO:0009987) | 11 out of 13 genes, 84.6% | 161 out of 273 genes, 59.0% | 0.04594542 |
| [negative regulation of response to stimulus](http://amigo.geneontology.org/amigo/term/GO:0048585) | 4 out of 13 genes, 30.8% | 31 out of 273 genes, 11.4% | 0.04691158 |
| [lipid biosynthetic process](http://amigo.geneontology.org/amigo/term/GO:0008610) | 2 out of 13 genes, 15.4% | 8 out of 273 genes, 2.9% | 0.04991286 |
| [negative regulation of cell migration](http://amigo.geneontology.org/amigo/term/GO:0030336) | 2 out of 13 genes, 15.4% | 8 out of 273 genes, 2.9% | 0.04991286 |
| [negative regulation of locomotion](http://amigo.geneontology.org/amigo/term/GO:0040013) | 2 out of 13 genes, 15.4% | 8 out of 273 genes, 2.9% | 0.04991286 |
| [positive regulation of cell adhesion](http://amigo.geneontology.org/amigo/term/GO:0045785) | 2 out of 13 genes, 15.4% | 8 out of 273 genes, 2.9% | 0.04991286 |
| [negative regulation of cellular component movement](http://amigo.geneontology.org/amigo/term/GO:0051271) | 2 out of 13 genes, 15.4% | 8 out of 273 genes, 2.9% | 0.04991286 |
| [negative regulation of fibrinolysis](http://amigo.geneontology.org/amigo/term/GO:0051918) | 2 out of 13 genes, 15.4% | 8 out of 273 genes, 2.9% | 0.04991286 |
| [acylglycerol homeostasis](http://amigo.geneontology.org/amigo/term/GO:0055090) | 2 out of 13 genes, 15.4% | 8 out of 273 genes, 2.9% | 0.04991286 |
| [triglyceride homeostasis](http://amigo.geneontology.org/amigo/term/GO:0070328) | 2 out of 13 genes, 15.4% | 8 out of 273 genes, 2.9% | 0.04991286 |
| [organophosphate biosynthetic process](http://amigo.geneontology.org/amigo/term/GO:0090407) | 2 out of 13 genes, 15.4% | 8 out of 273 genes, 2.9% | 0.04991286 |
| [negative regulation of cell motility](http://amigo.geneontology.org/amigo/term/GO:2000146) | 2 out of 13 genes, 15.4% | 8 out of 273 genes, 2.9% | 0.04991286 |

D. GO analysis between ASD-PAH and ASD - cellular component

| **Gene Ontology term** | **Cluster frequency** | **Protein frequency of use** | **P-value** |
| --- | --- | --- | --- |
| [lysosomal membrane](http://amigo.geneontology.org/amigo/term/GO:0005765) | 2 out of 11 genes, 18.2% | 4 out of 271 genes, 1.5% | 0.008622211 |
| [vacuolar membrane](http://amigo.geneontology.org/amigo/term/GO:0005774) | 2 out of 11 genes, 18.2% | 4 out of 271 genes, 1.5% | 0.008622211 |
| [axon](http://amigo.geneontology.org/amigo/term/GO:0030424) | 2 out of 11 genes, 18.2% | 5 out of 271 genes, 1.8% | 0.01404989 |
| [lysosomal lumen](http://amigo.geneontology.org/amigo/term/GO:0043202) | 2 out of 11 genes, 18.2% | 5 out of 271 genes, 1.8% | 0.01404989 |
| [vacuolar lumen](http://amigo.geneontology.org/amigo/term/GO:0005775) | 2 out of 11 genes, 18.2% | 6 out of 271 genes, 2.2% | 0.02060493 |
| [endoplasmic reticulum part](http://amigo.geneontology.org/amigo/term/GO:0044432) | 3 out of 11 genes, 27.3% | 17 out of 271 genes, 6.3% | 0.02483872 |
| [cytosol](http://amigo.geneontology.org/amigo/term/GO:0005829) | 4 out of 11 genes, 36.4% | 31 out of 271 genes, 11.4% | 0.02621268 |
| [endoplasmic reticulum membrane](http://amigo.geneontology.org/amigo/term/GO:0005789) | 2 out of 11 genes, 18.2% | 7 out of 271 genes, 2.6% | 0.02820384 |
| [nuclear outer membrane-endoplasmic reticulum membrane network](http://amigo.geneontology.org/amigo/term/GO:0042175) | 2 out of 11 genes, 18.2% | 7 out of 271 genes, 2.6% | 0.02820384 |
| [vacuolar part](http://amigo.geneontology.org/amigo/term/GO:0044437) | 2 out of 11 genes, 18.2% | 7 out of 271 genes, 2.6% | 0.02820384 |
| [extracellular region part](http://amigo.geneontology.org/amigo/term/GO:0044421) | 9 out of 11 genes, 81.8% | 135 out of 271 genes, 49.8% | 0.02921171 |
| [integral to membrane](http://amigo.geneontology.org/amigo/term/GO:0016021) | 3 out of 11 genes, 27.3% | 20 out of 271 genes, 7.4% | 0.03885273 |
| [lytic vacuole](http://amigo.geneontology.org/amigo/term/GO:0000323) | 2 out of 11 genes, 18.2% | 9 out of 271 genes, 3.3% | 0.04621856 |
| [lysosome](http://amigo.geneontology.org/amigo/term/GO:0005764) | 2 out of 11 genes, 18.2% | 9 out of 271 genes, 3.3% | 0.04621856 |
| [vacuole](http://amigo.geneontology.org/amigo/term/GO:0005773) | 2 out of 11 genes, 18.2% | 9 out of 271 genes, 3.3% | 0.04621856 |

E. GO analysis between ASD-PAH and ASD - molecular function

| **Gene Ontology term** | **Cluster frequency** | **Protein frequency of use** | **P-value** |
| --- | --- | --- | --- |
| [actin binding](http://amigo.geneontology.org/amigo/term/GO:0003779) | 3 out of 9 genes, 33.3% | 7 out of 258 genes, 2.7% | 0.0009675771 |
| [receptor binding](http://amigo.geneontology.org/amigo/term/GO:0005102) | 7 out of 9 genes, 77.8% | 64 out of 258 genes, 24.8% | 0.001032588 |
| [G-protein coupled receptor binding](http://amigo.geneontology.org/amigo/term/GO:0001664) | 3 out of 9 genes, 33.3% | 8 out of 258 genes, 3.1% | 0.001520593 |
| [cytoskeletal protein binding](http://amigo.geneontology.org/amigo/term/GO:0008092) | 3 out of 9 genes, 33.3% | 10 out of 258 genes, 3.9% | 0.003143229 |
| [glucosylceramidase activity](http://amigo.geneontology.org/amigo/term/GO:0004348) | 2 out of 9 genes, 22.2% | 3 out of 258 genes, 1.2% | 0.00319824 |
| [vitamin D binding](http://amigo.geneontology.org/amigo/term/GO:0005499) | 2 out of 9 genes, 22.2% | 3 out of 258 genes, 1.2% | 0.00319824 |
| [gamma-glutamyl carboxylase activity](http://amigo.geneontology.org/amigo/term/GO:0008488) | 2 out of 9 genes, 22.2% | 3 out of 258 genes, 1.2% | 0.00319824 |
| [carbon-carbon lyase activity](http://amigo.geneontology.org/amigo/term/GO:0016830) | 2 out of 9 genes, 22.2% | 3 out of 258 genes, 1.2% | 0.00319824 |
| [carboxy-lyase activity](http://amigo.geneontology.org/amigo/term/GO:0016831) | 2 out of 9 genes, 22.2% | 3 out of 258 genes, 1.2% | 0.00319824 |
| [hydrolase activity, hydrolyzing O-glycosyl compounds](http://amigo.geneontology.org/amigo/term/GO:0004553) | 2 out of 9 genes, 22.2% | 4 out of 258 genes, 1.6% | 0.006279808 |
| [hydrolase activity, acting on glycosyl bonds](http://amigo.geneontology.org/amigo/term/GO:0016798) | 2 out of 9 genes, 22.2% | 4 out of 258 genes, 1.6% | 0.006279808 |
| [vitamin binding](http://amigo.geneontology.org/amigo/term/GO:0019842) | 2 out of 9 genes, 22.2% | 4 out of 258 genes, 1.6% | 0.006279808 |
| [vitamin transporter activity](http://amigo.geneontology.org/amigo/term/GO:0051183) | 2 out of 9 genes, 22.2% | 4 out of 258 genes, 1.6% | 0.006279808 |
| [lyase activity](http://amigo.geneontology.org/amigo/term/GO:0016829) | 2 out of 9 genes, 22.2% | 5 out of 258 genes, 1.9% | 0.01027533 |
| [signal transducer activity](http://amigo.geneontology.org/amigo/term/GO:0004871) | 2 out of 9 genes, 22.2% | 7 out of 258 genes, 2.7% | 0.02079714 |
| [molecular transducer activity](http://amigo.geneontology.org/amigo/term/GO:0060089) | 2 out of 9 genes, 22.2% | 7 out of 258 genes, 2.7% | 0.02079714 |
| [transporter activity](http://amigo.geneontology.org/amigo/term/GO:0005215) | 3 out of 9 genes, 33.3% | 25 out of 258 genes, 9.7% | 0.04579541 |
| [steroid binding](http://amigo.geneontology.org/amigo/term/GO:0005496) | 2 out of 9 genes, 22.2% | 11 out of 258 genes, 4.3% | 0.05059151 |
| [alcohol binding](http://amigo.geneontology.org/amigo/term/GO:0043178) | 2 out of 9 genes, 22.2% | 15 out of 258 genes, 5.8% | 0.08969826 |
| [protein binding](http://amigo.geneontology.org/amigo/term/GO:0005515) | 8 out of 9 genes, 88.9% | 162 out of 258 genes, 62.8% | 0.09211224 |
| [organic cyclic compound binding](http://amigo.geneontology.org/amigo/term/GO:0097159) | 3 out of 9 genes, 33.3% | 39 out of 258 genes, 15.1% | 0.1399504 |
| [hydrolase activity](http://amigo.geneontology.org/amigo/term/GO:0016787) | 3 out of 9 genes, 33.3% | 42 out of 258 genes, 16.3% | 0.1660538 |
| [cation binding](http://amigo.geneontology.org/amigo/term/GO:0043169) | 4 out of 9 genes, 44.4% | 67 out of 258 genes, 26.0% | 0.1806943 |
| [enzyme binding](http://amigo.geneontology.org/amigo/term/GO:0019899) | 2 out of 9 genes, 22.2% | 30 out of 258 genes, 11.6% | 0.2814709 |
| [small molecule binding](http://amigo.geneontology.org/amigo/term/GO:0036094) | 2 out of 9 genes, 22.2% | 30 out of 258 genes, 11.6% | 0.2814709 |
| [lipid binding](http://amigo.geneontology.org/amigo/term/GO:0008289) | 2 out of 9 genes, 22.2% | 34 out of 258 genes, 13.2% | 0.3370967 |
| [ion binding](http://amigo.geneontology.org/amigo/term/GO:0043167) | 4 out of 9 genes, 44.4% | 86 out of 258 genes, 33.3% | 0.3480550 |
| [catalytic activity](http://amigo.geneontology.org/amigo/term/GO:0003824) | 3 out of 9 genes, 33.3% | 61 out of 258 genes, 23.6% | 0.3607275 |
| [binding](http://amigo.geneontology.org/amigo/term/GO:0005488) | 9 out of 9 genes, 100.0% | 232 out of 258 genes, 89.9% | 0.3783219 |

F. GO analysis between ASD-PAH and ASD - biological process

| **Gene Ontology term** | **Cluster frequency** | **Protein frequency of use** | **P-value** |
| --- | --- | --- | --- |
| [blood coagulation](http://amigo.geneontology.org/amigo/term/GO:0007596) | 10 out of 11 genes, 90.9% | 60 out of 273 genes, 22.0% | 1.279065e-06 |
| [hemostasis](http://amigo.geneontology.org/amigo/term/GO:0007599) | 10 out of 11 genes, 90.9% | 60 out of 273 genes, 22.0% | 1.279065e-06 |
| [coagulation](http://amigo.geneontology.org/amigo/term/GO:0050817) | 10 out of 11 genes, 90.9% | 60 out of 273 genes, 22.0% | 1.279065e-06 |
| [regulation of body fluid levels](http://amigo.geneontology.org/amigo/term/GO:0050878) | 10 out of 11 genes, 90.9% | 63 out of 273 genes, 23.1% | 2.141047e-06 |
| [wound healing](http://amigo.geneontology.org/amigo/term/GO:0042060) | 10 out of 11 genes, 90.9% | 64 out of 273 genes, 23.4% | 2.526799e-06 |
| [response to wounding](http://amigo.geneontology.org/amigo/term/GO:0009611) | 10 out of 11 genes, 90.9% | 79 out of 273 genes, 28.9% | 2.250065e-05 |
| [negative regulation of inflammatory response](http://amigo.geneontology.org/amigo/term/GO:0050728) | 5 out of 11 genes, 45.5% | 12 out of 273 genes, 4.4% | 2.630359e-05 |
| [negative regulation of response to external stimulus](http://amigo.geneontology.org/amigo/term/GO:0032102) | 5 out of 11 genes, 45.5% | 13 out of 273 genes, 4.8% | 4.193091e-05 |
| [neutrophil chemotaxis](http://amigo.geneontology.org/amigo/term/GO:0030593) | 3 out of 11 genes, 27.3% | 3 out of 273 genes, 1.1% | 4.919652e-05 |
| [positive regulation of interleukin-1 production](http://amigo.geneontology.org/amigo/term/GO:0032732) | 3 out of 11 genes, 27.3% | 3 out of 273 genes, 1.1% | 4.919652e-05 |
| [macrophage chemotaxis](http://amigo.geneontology.org/amigo/term/GO:0048246) | 3 out of 11 genes, 27.3% | 3 out of 273 genes, 1.1% | 4.919652e-05 |
| [lymphocyte chemotaxis](http://amigo.geneontology.org/amigo/term/GO:0048247) | 3 out of 11 genes, 27.3% | 3 out of 273 genes, 1.1% | 4.919652e-05 |
| [positive regulation of cytokine secretion](http://amigo.geneontology.org/amigo/term/GO:0050715) | 3 out of 11 genes, 27.3% | 3 out of 273 genes, 1.1% | 4.919652e-05 |
| [positive regulation of interleukin-1 secretion](http://amigo.geneontology.org/amigo/term/GO:0050716) | 3 out of 11 genes, 27.3% | 3 out of 273 genes, 1.1% | 4.919652e-05 |
| [lymphocyte migration](http://amigo.geneontology.org/amigo/term/GO:0072676) | 3 out of 11 genes, 27.3% | 3 out of 273 genes, 1.1% | 4.919652e-05 |
| [negative regulation of defense response](http://amigo.geneontology.org/amigo/term/GO:0031348) | 5 out of 11 genes, 45.5% | 15 out of 273 genes, 5.5% | 9.414022e-05 |
| [acute-phase response](http://amigo.geneontology.org/amigo/term/GO:0006953) | 5 out of 11 genes, 45.5% | 16 out of 273 genes, 5.9% | 0.0001343076 |
| [positive regulation of protein transport](http://amigo.geneontology.org/amigo/term/GO:0051222) | 4 out of 11 genes, 36.4% | 9 out of 273 genes, 3.3% | 0.0001652512 |
| [elevation of cytosolic calcium ion concentration](http://amigo.geneontology.org/amigo/term/GO:0007204) | 3 out of 11 genes, 27.3% | 4 out of 273 genes, 1.5% | 0.0001924130 |
| [cytosolic calcium ion homeostasis](http://amigo.geneontology.org/amigo/term/GO:0051480) | 3 out of 11 genes, 27.3% | 4 out of 273 genes, 1.5% | 0.0001924130 |
| [regulation of biological quality](http://amigo.geneontology.org/amigo/term/GO:0065008) | 10 out of 11 genes, 90.9% | 100 out of 273 genes, 36.6% | 0.0002445823 |
| [acute inflammatory response](http://amigo.geneontology.org/amigo/term/GO:0002526) | 5 out of 11 genes, 45.5% | 20 out of 273 genes, 7.3% | 0.0004409878 |
| [regulation of cellular localization](http://amigo.geneontology.org/amigo/term/GO:0060341) | 5 out of 11 genes, 45.5% | 20 out of 273 genes, 7.3% | 0.0004409878 |
| [cellular calcium ion homeostasis](http://amigo.geneontology.org/amigo/term/GO:0006874) | 3 out of 11 genes, 27.3% | 5 out of 273 genes, 1.8% | 0.0004703276 |
| [regulation of interleukin-1 secretion](http://amigo.geneontology.org/amigo/term/GO:0050704) | 3 out of 11 genes, 27.3% | 5 out of 273 genes, 1.8% | 0.0004703276 |
| [positive regulation of protein secretion](http://amigo.geneontology.org/amigo/term/GO:0050714) | 3 out of 11 genes, 27.3% | 5 out of 273 genes, 1.8% | 0.0004703276 |
| [calcium ion homeostasis](http://amigo.geneontology.org/amigo/term/GO:0055074) | 3 out of 11 genes, 27.3% | 5 out of 273 genes, 1.8% | 0.0004703276 |
| [cellular divalent inorganic cation homeostasis](http://amigo.geneontology.org/amigo/term/GO:0072503) | 3 out of 11 genes, 27.3% | 5 out of 273 genes, 1.8% | 0.0004703276 |
| [divalent inorganic cation homeostasis](http://amigo.geneontology.org/amigo/term/GO:0072507) | 3 out of 11 genes, 27.3% | 5 out of 273 genes, 1.8% | 0.0004703276 |
| [leukocyte migration](http://amigo.geneontology.org/amigo/term/GO:0050900) | 4 out of 11 genes, 36.4% | 13 out of 273 genes, 4.8% | 0.0008610007 |
| [regulation of cytokine production](http://amigo.geneontology.org/amigo/term/GO:0001817) | 5 out of 11 genes, 45.5% | 23 out of 273 genes, 8.4% | 0.0009022713 |
| [leukocyte chemotaxis](http://amigo.geneontology.org/amigo/term/GO:0030595) | 3 out of 11 genes, 27.3% | 6 out of 273 genes, 2.2% | 0.000919692 |
| [regulation of interleukin-1 production](http://amigo.geneontology.org/amigo/term/GO:0032652) | 3 out of 11 genes, 27.3% | 6 out of 273 genes, 2.2% | 0.000919692 |
| [cell chemotaxis](http://amigo.geneontology.org/amigo/term/GO:0060326) | 3 out of 11 genes, 27.3% | 6 out of 273 genes, 2.2% | 0.000919692 |
| [single-multicellular organism process](http://amigo.geneontology.org/amigo/term/GO:0044707) | 10 out of 11 genes, 90.9% | 118 out of 273 genes, 43.2% | 0.001252607 |
| [localization](http://amigo.geneontology.org/amigo/term/GO:0051179) | 9 out of 11 genes, 81.8% | 94 out of 273 genes, 34.4% | 0.001453066 |
| [regulation of cytokine secretion](http://amigo.geneontology.org/amigo/term/GO:0050707) | 3 out of 11 genes, 27.3% | 7 out of 273 genes, 2.6% | 0.001573542 |
| [regulation of protein localization](http://amigo.geneontology.org/amigo/term/GO:0032880) | 4 out of 11 genes, 36.4% | 15 out of 273 genes, 5.5% | 0.001574596 |
| [regulation of protein transport](http://amigo.geneontology.org/amigo/term/GO:0051223) | 4 out of 11 genes, 36.4% | 15 out of 273 genes, 5.5% | 0.001574596 |
| [regulation of establishment of protein localization](http://amigo.geneontology.org/amigo/term/GO:0070201) | 4 out of 11 genes, 36.4% | 15 out of 273 genes, 5.5% | 0.001574596 |
| [positive regulation of transport](http://amigo.geneontology.org/amigo/term/GO:0051050) | 5 out of 11 genes, 45.5% | 26 out of 273 genes, 9.5% | 0.001662003 |
| [multicellular organismal process](http://amigo.geneontology.org/amigo/term/GO:0032501) | 10 out of 11 genes, 90.9% | 123 out of 273 genes, 45.1% | 0.001875034 |
| [inflammatory response](http://amigo.geneontology.org/amigo/term/GO:0006954) | 5 out of 11 genes, 45.5% | 27 out of 273 genes, 9.9% | 0.00199948 |
| [peptidyl-glutamic acid carboxylation](http://amigo.geneontology.org/amigo/term/GO:0017187) | 3 out of 11 genes, 27.3% | 8 out of 273 genes, 2.9% | 0.002461403 |
| [peptidyl-glutamic acid modification](http://amigo.geneontology.org/amigo/term/GO:0018200) | 3 out of 11 genes, 27.3% | 8 out of 273 genes, 2.9% | 0.002461403 |
| [protein carboxylation](http://amigo.geneontology.org/amigo/term/GO:0018214) | 3 out of 11 genes, 27.3% | 8 out of 273 genes, 2.9% | 0.002461403 |
| [response to estradiol stimulus](http://amigo.geneontology.org/amigo/term/GO:0032355) | 3 out of 11 genes, 27.3% | 8 out of 273 genes, 2.9% | 0.002461403 |
| [post-translational protein modification](http://amigo.geneontology.org/amigo/term/GO:0043687) | 3 out of 11 genes, 27.3% | 8 out of 273 genes, 2.9% | 0.002461403 |
| [positive regulation of cell adhesion](http://amigo.geneontology.org/amigo/term/GO:0045785) | 3 out of 11 genes, 27.3% | 8 out of 273 genes, 2.9% | 0.002461403 |
| [positive regulation of secretion](http://amigo.geneontology.org/amigo/term/GO:0051047) | 3 out of 11 genes, 27.3% | 8 out of 273 genes, 2.9% | 0.002461403 |
| [regulation of protein secretion](http://amigo.geneontology.org/amigo/term/GO:0050708) | 3 out of 11 genes, 27.3% | 9 out of 273 genes, 3.3% | 0.003609478 |
| [negative regulation of response to stimulus](http://amigo.geneontology.org/amigo/term/GO:0048585) | 5 out of 11 genes, 45.5% | 31 out of 273 genes, 11.4% | 0.003883685 |
| [sphingolipid metabolic process](http://amigo.geneontology.org/amigo/term/GO:0006665) | 2 out of 11 genes, 18.2% | 3 out of 273 genes, 1.1% | 0.004345692 |
| [sphingosine metabolic process](http://amigo.geneontology.org/amigo/term/GO:0006670) | 2 out of 11 genes, 18.2% | 3 out of 273 genes, 1.1% | 0.004345692 |
| [ceramide metabolic process](http://amigo.geneontology.org/amigo/term/GO:0006672) | 2 out of 11 genes, 18.2% | 3 out of 273 genes, 1.1% | 0.004345692 |
| [glycosylceramide metabolic process](http://amigo.geneontology.org/amigo/term/GO:0006677) | 2 out of 11 genes, 18.2% | 3 out of 273 genes, 1.1% | 0.004345692 |
| [glucosylceramide metabolic process](http://amigo.geneontology.org/amigo/term/GO:0006678) | 2 out of 11 genes, 18.2% | 3 out of 273 genes, 1.1% | 0.004345692 |
| [glucosylceramide catabolic process](http://amigo.geneontology.org/amigo/term/GO:0006680) | 2 out of 11 genes, 18.2% | 3 out of 273 genes, 1.1% | 0.004345692 |
| [glycosphingolipid metabolic process](http://amigo.geneontology.org/amigo/term/GO:0006687) | 2 out of 11 genes, 18.2% | 3 out of 273 genes, 1.1% | 0.004345692 |
| [vacuole organization](http://amigo.geneontology.org/amigo/term/GO:0007033) | 2 out of 11 genes, 18.2% | 3 out of 273 genes, 1.1% | 0.004345692 |
| [lysosome organization](http://amigo.geneontology.org/amigo/term/GO:0007040) | 2 out of 11 genes, 18.2% | 3 out of 273 genes, 1.1% | 0.004345692 |
| [glycolipid catabolic process](http://amigo.geneontology.org/amigo/term/GO:0019377) | 2 out of 11 genes, 18.2% | 3 out of 273 genes, 1.1% | 0.004345692 |
| [termination of signal transduction](http://amigo.geneontology.org/amigo/term/GO:0023021) | 2 out of 11 genes, 18.2% | 3 out of 273 genes, 1.1% | 0.004345692 |
| [sphingolipid biosynthetic process](http://amigo.geneontology.org/amigo/term/GO:0030148) | 2 out of 11 genes, 18.2% | 3 out of 273 genes, 1.1% | 0.004345692 |
| [sphingolipid catabolic process](http://amigo.geneontology.org/amigo/term/GO:0030149) | 2 out of 11 genes, 18.2% | 3 out of 273 genes, 1.1% | 0.004345692 |
| [response to vitamin K](http://amigo.geneontology.org/amigo/term/GO:0032571) | 2 out of 11 genes, 18.2% | 3 out of 273 genes, 1.1% | 0.004345692 |
| [negative regulation of interleukin-6 production](http://amigo.geneontology.org/amigo/term/GO:0032715) | 2 out of 11 genes, 18.2% | 3 out of 273 genes, 1.1% | 0.004345692 |
| [diol metabolic process](http://amigo.geneontology.org/amigo/term/GO:0034311) | 2 out of 11 genes, 18.2% | 3 out of 273 genes, 1.1% | 0.004345692 |
| [diol biosynthetic process](http://amigo.geneontology.org/amigo/term/GO:0034312) | 2 out of 11 genes, 18.2% | 3 out of 273 genes, 1.1% | 0.004345692 |
| [vitamin D metabolic process](http://amigo.geneontology.org/amigo/term/GO:0042359) | 2 out of 11 genes, 18.2% | 3 out of 273 genes, 1.1% | 0.004345692 |
| [membrane lipid catabolic process](http://amigo.geneontology.org/amigo/term/GO:0046466) | 2 out of 11 genes, 18.2% | 3 out of 273 genes, 1.1% | 0.004345692 |
| [membrane lipid biosynthetic process](http://amigo.geneontology.org/amigo/term/GO:0046467) | 2 out of 11 genes, 18.2% | 3 out of 273 genes, 1.1% | 0.004345692 |
| [glycosylceramide catabolic process](http://amigo.geneontology.org/amigo/term/GO:0046477) | 2 out of 11 genes, 18.2% | 3 out of 273 genes, 1.1% | 0.004345692 |
| [glycosphingolipid catabolic process](http://amigo.geneontology.org/amigo/term/GO:0046479) | 2 out of 11 genes, 18.2% | 3 out of 273 genes, 1.1% | 0.004345692 |
| [sphingosine biosynthetic process](http://amigo.geneontology.org/amigo/term/GO:0046512) | 2 out of 11 genes, 18.2% | 3 out of 273 genes, 1.1% | 0.004345692 |
| [ceramide biosynthetic process](http://amigo.geneontology.org/amigo/term/GO:0046513) | 2 out of 11 genes, 18.2% | 3 out of 273 genes, 1.1% | 0.004345692 |
| [ceramide catabolic process](http://amigo.geneontology.org/amigo/term/GO:0046514) | 2 out of 11 genes, 18.2% | 3 out of 273 genes, 1.1% | 0.004345692 |
| [sphingoid metabolic process](http://amigo.geneontology.org/amigo/term/GO:0046519) | 2 out of 11 genes, 18.2% | 3 out of 273 genes, 1.1% | 0.004345692 |
| [sphingoid biosynthetic process](http://amigo.geneontology.org/amigo/term/GO:0046520) | 2 out of 11 genes, 18.2% | 3 out of 273 genes, 1.1% | 0.004345692 |
| [vitamin transport](http://amigo.geneontology.org/amigo/term/GO:0051180) | 2 out of 11 genes, 18.2% | 3 out of 273 genes, 1.1% | 0.004345692 |
| [response to estrogen stimulus](http://amigo.geneontology.org/amigo/term/GO:0043627) | 3 out of 11 genes, 27.3% | 10 out of 273 genes, 3.7% | 0.005040845 |
| [response to external stimulus](http://amigo.geneontology.org/amigo/term/GO:0009605) | 5 out of 11 genes, 45.5% | 33 out of 273 genes, 12.1% | 0.005209712 |
| [cellular metal ion homeostasis](http://amigo.geneontology.org/amigo/term/GO:0006875) | 3 out of 11 genes, 27.3% | 11 out of 273 genes, 4.0% | 0.00677563 |
| [response to cytokine stimulus](http://amigo.geneontology.org/amigo/term/GO:0034097) | 3 out of 11 genes, 27.3% | 11 out of 273 genes, 4.0% | 0.00677563 |
| [metal ion homeostasis](http://amigo.geneontology.org/amigo/term/GO:0055065) | 3 out of 11 genes, 27.3% | 11 out of 273 genes, 4.0% | 0.00677563 |
| [positive regulation of cellular process](http://amigo.geneontology.org/amigo/term/GO:0048522) | 7 out of 11 genes, 63.6% | 69 out of 273 genes, 25.3% | 0.006900681 |
| [single-organism cellular process](http://amigo.geneontology.org/amigo/term/GO:0044763) | 10 out of 11 genes, 90.9% | 141 out of 273 genes, 51.6% | 0.006929397 |
| [cell migration](http://amigo.geneontology.org/amigo/term/GO:0016477) | 4 out of 11 genes, 36.4% | 22 out of 273 genes, 8.1% | 0.00724927 |
| [membrane lipid metabolic process](http://amigo.geneontology.org/amigo/term/GO:0006643) | 2 out of 11 genes, 18.2% | 4 out of 273 genes, 1.5% | 0.008498972 |
| [glycolipid metabolic process](http://amigo.geneontology.org/amigo/term/GO:0006664) | 2 out of 11 genes, 18.2% | 4 out of 273 genes, 1.5% | 0.008498972 |
| [vitamin metabolic process](http://amigo.geneontology.org/amigo/term/GO:0006766) | 2 out of 11 genes, 18.2% | 4 out of 273 genes, 1.5% | 0.008498972 |
| [fat-soluble vitamin metabolic process](http://amigo.geneontology.org/amigo/term/GO:0006775) | 2 out of 11 genes, 18.2% | 4 out of 273 genes, 1.5% | 0.008498972 |
| [lactation](http://amigo.geneontology.org/amigo/term/GO:0007595) | 2 out of 11 genes, 18.2% | 4 out of 273 genes, 1.5% | 0.008498972 |
| [regulation of protein dephosphorylation](http://amigo.geneontology.org/amigo/term/GO:0035304) | 2 out of 11 genes, 18.2% | 4 out of 273 genes, 1.5% | 0.008498972 |
| [positive regulation of dephosphorylation](http://amigo.geneontology.org/amigo/term/GO:0035306) | 2 out of 11 genes, 18.2% | 4 out of 273 genes, 1.5% | 0.008498972 |
| [positive regulation of protein dephosphorylation](http://amigo.geneontology.org/amigo/term/GO:0035307) | 2 out of 11 genes, 18.2% | 4 out of 273 genes, 1.5% | 0.008498972 |
| [cell motility](http://amigo.geneontology.org/amigo/term/GO:0048870) | 4 out of 11 genes, 36.4% | 23 out of 273 genes, 8.4% | 0.00858548 |
| [localization of cell](http://amigo.geneontology.org/amigo/term/GO:0051674) | 4 out of 11 genes, 36.4% | 23 out of 273 genes, 8.4% | 0.00858548 |
| [cellular cation homeostasis](http://amigo.geneontology.org/amigo/term/GO:0030003) | 3 out of 11 genes, 27.3% | 12 out of 273 genes, 4.4% | 0.00883118 |
| [cation homeostasis](http://amigo.geneontology.org/amigo/term/GO:0055080) | 3 out of 11 genes, 27.3% | 12 out of 273 genes, 4.4% | 0.00883118 |
| [regulation of response to external stimulus](http://amigo.geneontology.org/amigo/term/GO:0032101) | 6 out of 11 genes, 54.5% | 54 out of 273 genes, 19.8% | 0.009513364 |
| [cellular response to chemical stimulus](http://amigo.geneontology.org/amigo/term/GO:0070887) | 5 out of 11 genes, 45.5% | 38 out of 273 genes, 13.9% | 0.009948375 |
| [regulation of inflammatory response](http://amigo.geneontology.org/amigo/term/GO:0050727) | 5 out of 11 genes, 45.5% | 39 out of 273 genes, 14.3% | 0.01117879 |
| [regulation of transport](http://amigo.geneontology.org/amigo/term/GO:0051049) | 5 out of 11 genes, 45.5% | 39 out of 273 genes, 14.3% | 0.01117879 |
| [positive regulation of cytokine production](http://amigo.geneontology.org/amigo/term/GO:0001819) | 3 out of 11 genes, 27.3% | 13 out of 273 genes, 4.8% | 0.01122223 |
| [negative regulation of cellular protein metabolic process](http://amigo.geneontology.org/amigo/term/GO:0032269) | 3 out of 11 genes, 27.3% | 13 out of 273 genes, 4.8% | 0.01122223 |
| [platelet activation](http://amigo.geneontology.org/amigo/term/GO:0030168) | 5 out of 11 genes, 45.5% | 40 out of 273 genes, 14.7% | 0.01251468 |
| [negative regulation of protein metabolic process](http://amigo.geneontology.org/amigo/term/GO:0051248) | 4 out of 11 genes, 36.4% | 26 out of 273 genes, 9.5% | 0.01357011 |
| [body fluid secretion](http://amigo.geneontology.org/amigo/term/GO:0007589) | 2 out of 11 genes, 18.2% | 5 out of 273 genes, 1.8% | 0.0138514 |
| [mammary gland development](http://amigo.geneontology.org/amigo/term/GO:0030879) | 2 out of 11 genes, 18.2% | 5 out of 273 genes, 1.8% | 0.0138514 |
| [regulation of interleukin-6 production](http://amigo.geneontology.org/amigo/term/GO:0032675) | 2 out of 11 genes, 18.2% | 5 out of 273 genes, 1.8% | 0.0138514 |
| [secretion by tissue](http://amigo.geneontology.org/amigo/term/GO:0032941) | 2 out of 11 genes, 18.2% | 5 out of 273 genes, 1.8% | 0.0138514 |
| [regulation of dephosphorylation](http://amigo.geneontology.org/amigo/term/GO:0035303) | 2 out of 11 genes, 18.2% | 5 out of 273 genes, 1.8% | 0.0138514 |
| [cellular ion homeostasis](http://amigo.geneontology.org/amigo/term/GO:0006873) | 3 out of 11 genes, 27.3% | 14 out of 273 genes, 5.1% | 0.01396108 |
| [chemotaxis](http://amigo.geneontology.org/amigo/term/GO:0006935) | 3 out of 11 genes, 27.3% | 14 out of 273 genes, 5.1% | 0.01396108 |
| [peptidyl-amino acid modification](http://amigo.geneontology.org/amigo/term/GO:0018193) | 3 out of 11 genes, 27.3% | 14 out of 273 genes, 5.1% | 0.01396108 |
| [taxis](http://amigo.geneontology.org/amigo/term/GO:0042330) | 3 out of 11 genes, 27.3% | 14 out of 273 genes, 5.1% | 0.01396108 |
| [regulation of secretion](http://amigo.geneontology.org/amigo/term/GO:0051046) | 3 out of 11 genes, 27.3% | 14 out of 273 genes, 5.1% | 0.01396108 |
| [cellular chemical homeostasis](http://amigo.geneontology.org/amigo/term/GO:0055082) | 3 out of 11 genes, 27.3% | 14 out of 273 genes, 5.1% | 0.01396108 |
| [cellular component movement](http://amigo.geneontology.org/amigo/term/GO:0006928) | 4 out of 11 genes, 36.4% | 27 out of 273 genes, 9.9% | 0.0155824 |
| [regulation of response to stress](http://amigo.geneontology.org/amigo/term/GO:0080134) | 6 out of 11 genes, 54.5% | 60 out of 273 genes, 22.0% | 0.0164605 |
| [response to steroid hormone stimulus](http://amigo.geneontology.org/amigo/term/GO:0048545) | 3 out of 11 genes, 27.3% | 15 out of 273 genes, 5.5% | 0.01705770 |
| [ion homeostasis](http://amigo.geneontology.org/amigo/term/GO:0050801) | 3 out of 11 genes, 27.3% | 15 out of 273 genes, 5.5% | 0.01705770 |
| [regulation of defense response](http://amigo.geneontology.org/amigo/term/GO:0031347) | 5 out of 11 genes, 45.5% | 43 out of 273 genes, 15.8% | 0.01720224 |
| [response to stress](http://amigo.geneontology.org/amigo/term/GO:0006950) | 11 out of 11 genes, 100.0% | 192 out of 273 genes, 70.3% | 0.01907323 |
| [negative regulation of biological process](http://amigo.geneontology.org/amigo/term/GO:0048519) | 7 out of 11 genes, 63.6% | 82 out of 273 genes, 30.0% | 0.01955382 |
| [locomotion](http://amigo.geneontology.org/amigo/term/GO:0040011) | 4 out of 11 genes, 36.4% | 29 out of 273 genes, 10.6% | 0.02017475 |
| [negative regulation of protein kinase activity](http://amigo.geneontology.org/amigo/term/GO:0006469) | 2 out of 11 genes, 18.2% | 6 out of 273 genes, 2.2% | 0.02031726 |
| [response to vitamin](http://amigo.geneontology.org/amigo/term/GO:0033273) | 2 out of 11 genes, 18.2% | 6 out of 273 genes, 2.2% | 0.02031726 |
| [negative regulation of kinase activity](http://amigo.geneontology.org/amigo/term/GO:0033673) | 2 out of 11 genes, 18.2% | 6 out of 273 genes, 2.2% | 0.02031726 |
| [negative regulation of MAP kinase activity](http://amigo.geneontology.org/amigo/term/GO:0043407) | 2 out of 11 genes, 18.2% | 6 out of 273 genes, 2.2% | 0.02031726 |
| [negative regulation of transferase activity](http://amigo.geneontology.org/amigo/term/GO:0051348) | 2 out of 11 genes, 18.2% | 6 out of 273 genes, 2.2% | 0.02031726 |
| [cellular response to tumor necrosis factor](http://amigo.geneontology.org/amigo/term/GO:0071356) | 2 out of 11 genes, 18.2% | 6 out of 273 genes, 2.2% | 0.02031726 |
| [negative regulation of protein serine/threonine kinase activity](http://amigo.geneontology.org/amigo/term/GO:0071901) | 2 out of 11 genes, 18.2% | 6 out of 273 genes, 2.2% | 0.02031726 |
| [organelle organization](http://amigo.geneontology.org/amigo/term/GO:0006996) | 3 out of 11 genes, 27.3% | 16 out of 273 genes, 5.9% | 0.02051993 |
| [cellular homeostasis](http://amigo.geneontology.org/amigo/term/GO:0019725) | 3 out of 11 genes, 27.3% | 16 out of 273 genes, 5.9% | 0.02051993 |
| [response to alcohol](http://amigo.geneontology.org/amigo/term/GO:0097305) | 3 out of 11 genes, 27.3% | 16 out of 273 genes, 5.9% | 0.02051993 |
| [regulation of multicellular organismal process](http://amigo.geneontology.org/amigo/term/GO:0051239) | 6 out of 11 genes, 54.5% | 63 out of 273 genes, 23.1% | 0.0210885 |
| [positive regulation of multicellular organismal process](http://amigo.geneontology.org/amigo/term/GO:0051240) | 4 out of 11 genes, 36.4% | 30 out of 273 genes, 11.0% | 0.02276724 |
| [cellular process](http://amigo.geneontology.org/amigo/term/GO:0009987) | 10 out of 11 genes, 90.9% | 161 out of 273 genes, 59.0% | 0.02377299 |
| [cell death](http://amigo.geneontology.org/amigo/term/GO:0008219) | 3 out of 11 genes, 27.3% | 17 out of 273 genes, 6.2% | 0.02435359 |
| [death](http://amigo.geneontology.org/amigo/term/GO:0016265) | 3 out of 11 genes, 27.3% | 17 out of 273 genes, 6.2% | 0.02435359 |
| [cell activation](http://amigo.geneontology.org/amigo/term/GO:0001775) | 5 out of 11 genes, 45.5% | 47 out of 273 genes, 17.2% | 0.02520803 |
| [negative regulation of macromolecule metabolic process](http://amigo.geneontology.org/amigo/term/GO:0010605) | 4 out of 11 genes, 36.4% | 31 out of 273 genes, 11.4% | 0.0255648 |
| [negative regulation of intracellular protein kinase cascade](http://amigo.geneontology.org/amigo/term/GO:0010741) | 2 out of 11 genes, 18.2% | 7 out of 273 genes, 2.6% | 0.02781474 |
| [positive regulation of intracellular transport](http://amigo.geneontology.org/amigo/term/GO:0032388) | 2 out of 11 genes, 18.2% | 7 out of 273 genes, 2.6% | 0.02781474 |
| [response to tumor necrosis factor](http://amigo.geneontology.org/amigo/term/GO:0034612) | 2 out of 11 genes, 18.2% | 7 out of 273 genes, 2.6% | 0.02781474 |
| [negative regulation of MAPK cascade](http://amigo.geneontology.org/amigo/term/GO:0043409) | 2 out of 11 genes, 18.2% | 7 out of 273 genes, 2.6% | 0.02781474 |
| [alcohol biosynthetic process](http://amigo.geneontology.org/amigo/term/GO:0046165) | 2 out of 11 genes, 18.2% | 7 out of 273 genes, 2.6% | 0.02781474 |
| [organic hydroxy compound biosynthetic process](http://amigo.geneontology.org/amigo/term/GO:1901617) | 2 out of 11 genes, 18.2% | 7 out of 273 genes, 2.6% | 0.02781474 |
| [regulation of localization](http://amigo.geneontology.org/amigo/term/GO:0032879) | 5 out of 11 genes, 45.5% | 50 out of 273 genes, 18.3% | 0.03267724 |
| [positive regulation of phosphorus metabolic process](http://amigo.geneontology.org/amigo/term/GO:0010562) | 3 out of 11 genes, 27.3% | 19 out of 273 genes, 7.0% | 0.03314926 |
| [regulation of intracellular protein kinase cascade](http://amigo.geneontology.org/amigo/term/GO:0010627) | 3 out of 11 genes, 27.3% | 19 out of 273 genes, 7.0% | 0.03314926 |
| [regulation of cell adhesion](http://amigo.geneontology.org/amigo/term/GO:0030155) | 3 out of 11 genes, 27.3% | 19 out of 273 genes, 7.0% | 0.03314926 |
| [positive regulation of phosphate metabolic process](http://amigo.geneontology.org/amigo/term/GO:0045937) | 3 out of 11 genes, 27.3% | 19 out of 273 genes, 7.0% | 0.03314926 |
| [negative regulation of protein phosphorylation](http://amigo.geneontology.org/amigo/term/GO:0001933) | 2 out of 11 genes, 18.2% | 8 out of 273 genes, 2.9% | 0.03626585 |
| [lipid biosynthetic process](http://amigo.geneontology.org/amigo/term/GO:0008610) | 2 out of 11 genes, 18.2% | 8 out of 273 genes, 2.9% | 0.03626585 |
| [response to ketone](http://amigo.geneontology.org/amigo/term/GO:1901654) | 2 out of 11 genes, 18.2% | 8 out of 273 genes, 2.9% | 0.03626585 |
| [positive regulation of protein modification process](http://amigo.geneontology.org/amigo/term/GO:0031401) | 3 out of 11 genes, 27.3% | 20 out of 273 genes, 7.3% | 0.03811403 |
| [response to lipid](http://amigo.geneontology.org/amigo/term/GO:0033993) | 3 out of 11 genes, 27.3% | 21 out of 273 genes, 7.7% | 0.04345601 |
| [female pregnancy](http://amigo.geneontology.org/amigo/term/GO:0007565) | 2 out of 11 genes, 18.2% | 9 out of 273 genes, 3.3% | 0.04559625 |
| [embryo development](http://amigo.geneontology.org/amigo/term/GO:0009790) | 2 out of 11 genes, 18.2% | 9 out of 273 genes, 3.3% | 0.04559625 |
| [negative regulation of protein modification process](http://amigo.geneontology.org/amigo/term/GO:0031400) | 2 out of 11 genes, 18.2% | 9 out of 273 genes, 3.3% | 0.04559625 |
| [regulation of intracellular transport](http://amigo.geneontology.org/amigo/term/GO:0032386) | 2 out of 11 genes, 18.2% | 9 out of 273 genes, 3.3% | 0.04559625 |
| [regulation of MAP kinase activity](http://amigo.geneontology.org/amigo/term/GO:0043405) | 2 out of 11 genes, 18.2% | 9 out of 273 genes, 3.3% | 0.04559625 |
| [cellular nitrogen compound biosynthetic process](http://amigo.geneontology.org/amigo/term/GO:0044271) | 2 out of 11 genes, 18.2% | 9 out of 273 genes, 3.3% | 0.04559625 |
| [multi-multicellular organism process](http://amigo.geneontology.org/amigo/term/GO:0044706) | 2 out of 11 genes, 18.2% | 9 out of 273 genes, 3.3% | 0.04559625 |
| [gland development](http://amigo.geneontology.org/amigo/term/GO:0048732) | 2 out of 11 genes, 18.2% | 9 out of 273 genes, 3.3% | 0.04559625 |
| [cellular response to cytokine stimulus](http://amigo.geneontology.org/amigo/term/GO:0071345) | 2 out of 11 genes, 18.2% | 9 out of 273 genes, 3.3% | 0.04559625 |
| [response to hormone stimulus](http://amigo.geneontology.org/amigo/term/GO:0009725) | 3 out of 11 genes, 27.3% | 22 out of 273 genes, 8.1% | 0.04917286 |
| [positive regulation of cellular protein metabolic process](http://amigo.geneontology.org/amigo/term/GO:0032270) | 3 out of 11 genes, 27.3% | 22 out of 273 genes, 8.1% | 0.04917286 |
| [cellular response to stimulus](http://amigo.geneontology.org/amigo/term/GO:0051716) | 6 out of 11 genes, 54.5% | 75 out of 273 genes, 27.5% | 0.04934145 |

**G. GO analysis between Mix-PAH and Mix - cellular component**

| **Gene Ontology term** | **Cluster frequency** | **Protein frequency of use** | **P-value** |
| --- | --- | --- | --- |
| [hemoglobin complex](http://amigo.geneontology.org/amigo/term/GO:0005833) | 3 out of 26 genes, 11.5% | 3 out of 271 genes, 1.1% | 0.000792573 |
| [intracellular part](http://amigo.geneontology.org/amigo/term/GO:0044424) | 18 out of 26 genes, 69.2% | 113 out of 271 genes, 41.7% | 0.002786632 |
| [organelle part](http://amigo.geneontology.org/amigo/term/GO:0044422) | 15 out of 26 genes, 57.7% | 85 out of 271 genes, 31.4% | 0.003193746 |
| [intracellular](http://amigo.geneontology.org/amigo/term/GO:0005622) | 18 out of 26 genes, 69.2% | 115 out of 271 genes, 42.4% | 0.003573397 |
| [organelle](http://amigo.geneontology.org/amigo/term/GO:0043226) | 17 out of 26 genes, 65.4% | 105 out of 271 genes, 38.7% | 0.003621549 |
| [cytoplasmic part](http://amigo.geneontology.org/amigo/term/GO:0044444) | 16 out of 26 genes, 61.5% | 96 out of 271 genes, 35.4% | 0.00397878 |
| [cytoplasm](http://amigo.geneontology.org/amigo/term/GO:0005737) | 17 out of 26 genes, 65.4% | 106 out of 271 genes, 39.1% | 0.004103763 |
| [vesicle](http://amigo.geneontology.org/amigo/term/GO:0031982) | 10 out of 26 genes, 38.5% | 46 out of 271 genes, 17.0% | 0.004915721 |
| [cell junction](http://amigo.geneontology.org/amigo/term/GO:0030054) | 4 out of 26 genes, 15.4% | 9 out of 271 genes, 3.3% | 0.006094099 |
| [cytosolic part](http://amigo.geneontology.org/amigo/term/GO:0044445) | 3 out of 26 genes, 11.5% | 5 out of 271 genes, 1.8% | 0.006939067 |
| [intracellular organelle part](http://amigo.geneontology.org/amigo/term/GO:0044446) | 14 out of 26 genes, 53.8% | 82 out of 271 genes, 30.3% | 0.007220111 |
| [cell projection](http://amigo.geneontology.org/amigo/term/GO:0042995) | 5 out of 26 genes, 19.2% | 15 out of 271 genes, 5.5% | 0.008493643 |
| [spindle pole](http://amigo.geneontology.org/amigo/term/GO:0000922) | 2 out of 26 genes, 7.7% | 2 out of 271 genes, 0.7% | 0.008883422 |
| [centriole](http://amigo.geneontology.org/amigo/term/GO:0005814) | 2 out of 26 genes, 7.7% | 2 out of 271 genes, 0.7% | 0.008883422 |
| [microtubule organizing center part](http://amigo.geneontology.org/amigo/term/GO:0044450) | 2 out of 26 genes, 7.7% | 2 out of 271 genes, 0.7% | 0.008883422 |
| [neuron projection](http://amigo.geneontology.org/amigo/term/GO:0043005) | 4 out of 26 genes, 15.4% | 10 out of 271 genes, 3.7% | 0.009487363 |
| [neuron part](http://amigo.geneontology.org/amigo/term/GO:0097458) | 4 out of 26 genes, 15.4% | 10 out of 271 genes, 3.7% | 0.009487363 |
| [intracellular organelle](http://amigo.geneontology.org/amigo/term/GO:0043229) | 16 out of 26 genes, 61.5% | 104 out of 271 genes, 38.4% | 0.01040654 |
| [centrosome](http://amigo.geneontology.org/amigo/term/GO:0005813) | 3 out of 26 genes, 11.5% | 6 out of 271 genes, 2.2% | 0.01298793 |
| [microtubule organizing center](http://amigo.geneontology.org/amigo/term/GO:0005815) | 3 out of 26 genes, 11.5% | 6 out of 271 genes, 2.2% | 0.01298793 |
| [cytoplasmic vesicle](http://amigo.geneontology.org/amigo/term/GO:0031410) | 9 out of 26 genes, 34.6% | 45 out of 271 genes, 16.6% | 0.01493368 |
| [cell projection part](http://amigo.geneontology.org/amigo/term/GO:0044463) | 3 out of 26 genes, 11.5% | 7 out of 271 genes, 2.6% | 0.02127373 |
| [membrane-bounded organelle](http://amigo.geneontology.org/amigo/term/GO:0043227) | 14 out of 26 genes, 53.8% | 92 out of 271 genes, 33.9% | 0.02294396 |
| [intracellular membrane-bounded organelle](http://amigo.geneontology.org/amigo/term/GO:0043231) | 14 out of 26 genes, 53.8% | 92 out of 271 genes, 33.9% | 0.02294396 |
| [spindle](http://amigo.geneontology.org/amigo/term/GO:0005819) | 2 out of 26 genes, 7.7% | 3 out of 271 genes, 1.1% | 0.02506512 |
| [basolateral plasma membrane](http://amigo.geneontology.org/amigo/term/GO:0016323) | 2 out of 26 genes, 7.7% | 3 out of 271 genes, 1.1% | 0.02506512 |
| [synapse](http://amigo.geneontology.org/amigo/term/GO:0045202) | 2 out of 26 genes, 7.7% | 3 out of 271 genes, 1.1% | 0.02506512 |
| cytoplasmic membrane-bounded vesicle | 8 out of 26 genes, 30.8% | 43 out of 271 genes, 15.9% | 0.03512211 |
| [membrane-bounded vesicle](http://amigo.geneontology.org/amigo/term/GO:0031988) | 8 out of 26 genes, 30.8% | 43 out of 271 genes, 15.9% | 0.03512211 |
| [cytoskeleton](http://amigo.geneontology.org/amigo/term/GO:0005856) | 5 out of 26 genes, 19.2% | 21 out of 271 genes, 7.7% | 0.03808097 |
| [microtubule cytoskeleton](http://amigo.geneontology.org/amigo/term/GO:0015630) | 3 out of 26 genes, 11.5% | 9 out of 271 genes, 3.3% | 0.04474781 |
| [membrane-enclosed lumen](http://amigo.geneontology.org/amigo/term/GO:0031974) | 10 out of 26 genes, 38.5% | 62 out of 271 genes, 22.9% | 0.04545834 |
| organelle lumen | 10 out of 26 genes, 38.5% | 62 out of 271 genes, 22.9% | 0.04545834 |
| [haptoglobin-hemoglobin complex](http://amigo.geneontology.org/amigo/term/GO:0031838) | 2 out of 26 genes, 7.7% | 4 out of 271 genes, 1.5% | 0.04716401 |
| [apical junction complex](http://amigo.geneontology.org/amigo/term/GO:0043296) | 2 out of 26 genes, 7.7% | 4 out of 271 genes, 1.5% | 0.04716401 |

**H. GO analysis between Mix-PAH and Mix – molecular function**

| **Gene Ontology term** | **Cluster frequency** | **Protein frequency of use** | **P-value** |
| --- | --- | --- | --- |
| [oxygen binding](http://amigo.geneontology.org/amigo/term/GO:0019825) | 5 out of 23 genes, 21.7% | 5 out of 258 genes, 1.9% | 3.672711e-06 |
| [transition metal ion binding](http://amigo.geneontology.org/amigo/term/GO:0046914) | 8 out of 23 genes, 34.8% | 21 out of 258 genes, 8.1% | 0.0001107129 |
| [iron ion binding](http://amigo.geneontology.org/amigo/term/GO:0005506) | 4 out of 23 genes, 17.4% | 5 out of 258 genes, 1.9% | 0.0002308009 |
| [organic cyclic compound binding](http://amigo.geneontology.org/amigo/term/GO:0097159) | 10 out of 23 genes, 43.5% | 39 out of 258 genes, 15.1% | 0.0005455992 |
| [oxygen transporter activity](http://amigo.geneontology.org/amigo/term/GO:0005344) | 3 out of 23 genes, 13.0% | 3 out of 258 genes, 1.2% | 0.0006260039 |
| [heterocyclic compound binding](http://amigo.geneontology.org/amigo/term/GO:1901363) | 8 out of 23 genes, 34.8% | 28 out of 258 genes, 10.9% | 0.001131201 |
| [antioxidant activity](http://amigo.geneontology.org/amigo/term/GO:0016209) | 5 out of 23 genes, 21.7% | 11 out of 258 genes, 4.3% | 0.001174211 |
| [transporter activity](http://amigo.geneontology.org/amigo/term/GO:0005215) | 7 out of 23 genes, 30.4% | 25 out of 258 genes, 9.7% | 0.00294678 |
| [ion binding](http://amigo.geneontology.org/amigo/term/GO:0043167) | 14 out of 23 genes, 60.9% | 86 out of 258 genes, 33.3% | 0.004293848 |
| [substrate-specific transporter activity](http://amigo.geneontology.org/amigo/term/GO:0022892) | 6 out of 23 genes, 26.1% | 21 out of 258 genes, 8.1% | 0.005724936 |
| [drug binding](http://amigo.geneontology.org/amigo/term/GO:0008144) | 2 out of 23 genes, 8.7% | 2 out of 258 genes, 0.8% | 0.007631285 |
| [toxic substance binding](http://amigo.geneontology.org/amigo/term/GO:0015643) | 2 out of 23 genes, 8.7% | 2 out of 258 genes, 0.8% | 0.007631285 |
| [pyridoxal phosphate binding](http://amigo.geneontology.org/amigo/term/GO:0030170) | 2 out of 23 genes, 8.7% | 2 out of 258 genes, 0.8% | 0.007631285 |
| [haptoglobin binding](http://amigo.geneontology.org/amigo/term/GO:0031720) | 2 out of 23 genes, 8.7% | 2 out of 258 genes, 0.8% | 0.007631285 |
| [cofactor binding](http://amigo.geneontology.org/amigo/term/GO:0048037) | 3 out of 23 genes, 13.0% | 6 out of 258 genes, 2.3% | 0.01044026 |
| [cation binding](http://amigo.geneontology.org/amigo/term/GO:0043169) | 11 out of 23 genes, 47.8% | 67 out of 258 genes, 26.0% | 0.01518981 |
| [copper ion binding](http://amigo.geneontology.org/amigo/term/GO:0005507) | 3 out of 23 genes, 13.0% | 7 out of 258 genes, 2.7% | 0.01719954 |
| [fatty acid binding](http://amigo.geneontology.org/amigo/term/GO:0005504) | 2 out of 23 genes, 8.7% | 3 out of 258 genes, 1.2% | 0.02164185 |
| [DNA binding](http://amigo.geneontology.org/amigo/term/GO:0003677) | 3 out of 23 genes, 13.0% | 8 out of 258 genes, 3.1% | 0.02590852 |
| [carboxylic acid binding](http://amigo.geneontology.org/amigo/term/GO:0031406) | 3 out of 23 genes, 13.0% | 8 out of 258 genes, 3.1% | 0.02590852 |
| [enzyme binding](http://amigo.geneontology.org/amigo/term/GO:0019899) | 6 out of 23 genes, 26.1% | 30 out of 258 genes, 11.6% | 0.03589501 |
| [heme binding](http://amigo.geneontology.org/amigo/term/GO:0020037) | 3 out of 23 genes, 13.0% | 9 out of 258 genes, 3.5% | 0.03659154 |
| [tetrapyrrole binding](http://amigo.geneontology.org/amigo/term/GO:0046906) | 3 out of 23 genes, 13.0% | 9 out of 258 genes, 3.5% | 0.03659154 |
| [hydrolase activity, hydrolyzing O-glycosyl compounds](http://amigo.geneontology.org/amigo/term/GO:0004553) | 2 out of 23 genes, 8.7% | 4 out of 258 genes, 1.6% | 0.04092698 |
| [hydrolase activity, acting on glycosyl bonds](http://amigo.geneontology.org/amigo/term/GO:0016798) | 2 out of 23 genes, 8.7% | 4 out of 258 genes, 1.6% | 0.04092698 |
| [monocarboxylic acid binding](http://amigo.geneontology.org/amigo/term/GO:0033293) | 2 out of 23 genes, 8.7% | 4 out of 258 genes, 1.6% | 0.04092698 |
| [metal ion binding](http://amigo.geneontology.org/amigo/term/GO:0046872) | 9 out of 23 genes, 39.1% | 57 out of 258 genes, 22.1% | 0.04127032 |
| [cell surface binding](http://amigo.geneontology.org/amigo/term/GO:0043498) | 5 out of 23 genes, 21.7% | 24 out of 258 genes, 9.3% | 0.04817428 |
| [nucleic acid binding](http://amigo.geneontology.org/amigo/term/GO:0003676) | 3 out of 23 genes, 13.0% | 10 out of 258 genes, 3.9% | 0.0492236 |

**I.** GO analysis between Mix-PAH and Mix – biological process

| **Gene Ontology term** | **Cluster frequency** | **Protein frequency of use** | **P-value** |
| --- | --- | --- | --- |
| [organic anion transport](http://amigo.geneontology.org/amigo/term/GO:0015711) | 4 out of 24 genes, 16.7% | 4 out of 273 genes, 1.5% | 4.693712e-05 |
| [ion transport](http://amigo.geneontology.org/amigo/term/GO:0006811) | 5 out of 24 genes, 20.8% | 8 out of 273 genes, 2.9% | 0.0001627531 |
| [anion transport](http://amigo.geneontology.org/amigo/term/GO:0006820) | 4 out of 24 genes, 16.7% | 5 out of 273 genes, 1.8% | 0.0002207266 |
| [regulation of biological quality](http://amigo.geneontology.org/amigo/term/GO:0065008) | 17 out of 24 genes, 70.8% | 100 out of 273 genes, 36.6% | 0.000397383 |
| [gas transport](http://amigo.geneontology.org/amigo/term/GO:0015669) | 3 out of 24 genes, 12.5% | 3 out of 273 genes, 1.1% | 0.0006034773 |
| [oxygen transport](http://amigo.geneontology.org/amigo/term/GO:0015671) | 3 out of 24 genes, 12.5% | 3 out of 273 genes, 1.1% | 0.0006034773 |
| [positive regulation of behavior](http://amigo.geneontology.org/amigo/term/GO:0048520) | 4 out of 24 genes, 16.7% | 6 out of 273 genes, 2.2% | 0.0006227769 |
| [regulation of behavior](http://amigo.geneontology.org/amigo/term/GO:0050795) | 4 out of 24 genes, 16.7% | 7 out of 273 genes, 2.6% | 0.001366645 |
| [regulation of cell death](http://amigo.geneontology.org/amigo/term/GO:0010941) | 8 out of 24 genes, 33.3% | 29 out of 273 genes, 10.6% | 0.001391296 |
| [small molecule metabolic process](http://amigo.geneontology.org/amigo/term/GO:0044281) | 10 out of 24 genes, 41.7% | 44 out of 273 genes, 16.1% | 0.001533809 |
| [modification by symbiont of host morphology or physiology](http://amigo.geneontology.org/amigo/term/GO:0044003) | 3 out of 24 genes, 12.5% | 4 out of 273 genes, 1.5% | 0.002273098 |
| [cellular response to stress](http://amigo.geneontology.org/amigo/term/GO:0033554) | 6 out of 24 genes, 25.0% | 19 out of 273 genes, 7.0% | 0.003068129 |
| [single-organism metabolic process](http://amigo.geneontology.org/amigo/term/GO:0044710) | 11 out of 24 genes, 45.8% | 57 out of 273 genes, 20.9% | 0.00356289 |
| [single-multicellular organism process](http://amigo.geneontology.org/amigo/term/GO:0044707) | 17 out of 24 genes, 70.8% | 118 out of 273 genes, 43.2% | 0.004132237 |
| [regulation of cell cycle](http://amigo.geneontology.org/amigo/term/GO:0051726) | 3 out of 24 genes, 12.5% | 5 out of 273 genes, 1.8% | 0.005351654 |
| [modification of morphology or physiology of other organism involved in symbiotic interaction](http://amigo.geneontology.org/amigo/term/GO:0051817) | 3 out of 24 genes, 12.5% | 5 out of 273 genes, 1.8% | 0.005351654 |
| [single-organism transport](http://amigo.geneontology.org/amigo/term/GO:0044765) | 13 out of 24 genes, 54.2% | 79 out of 273 genes, 28.9% | 0.005919075 |
| [response to nutrient](http://amigo.geneontology.org/amigo/term/GO:0007584) | 4 out of 24 genes, 16.7% | 10 out of 273 genes, 3.7% | 0.006820298 |
| [multicellular organismal process](http://amigo.geneontology.org/amigo/term/GO:0032501) | 17 out of 24 genes, 70.8% | 123 out of 273 genes, 45.1% | 0.007158077 |
| [cellular component organization](http://amigo.geneontology.org/amigo/term/GO:0016043) | 12 out of 24 genes, 50.0% | 71 out of 273 genes, 26.0% | 0.007241628 |
| [cellular component organization or biogenesis](http://amigo.geneontology.org/amigo/term/GO:0071840) | 12 out of 24 genes, 50.0% | 71 out of 273 genes, 26.0% | 0.007241628 |
| [rhythmic behavior](http://amigo.geneontology.org/amigo/term/GO:0007622) | 2 out of 24 genes, 8.3% | 2 out of 273 genes, 0.7% | 0.007433743 |
| [circadian rhythm](http://amigo.geneontology.org/amigo/term/GO:0007623) | 2 out of 24 genes, 8.3% | 2 out of 273 genes, 0.7% | 0.007433743 |
| [bile acid metabolic process](http://amigo.geneontology.org/amigo/term/GO:0008206) | 2 out of 24 genes, 8.3% | 2 out of 273 genes, 0.7% | 0.007433743 |
| [regulation of fibroblast migration](http://amigo.geneontology.org/amigo/term/GO:0010762) | 2 out of 24 genes, 8.3% | 2 out of 273 genes, 0.7% | 0.007433743 |
| [positive regulation of fibroblast migration](http://amigo.geneontology.org/amigo/term/GO:0010763) | 2 out of 24 genes, 8.3% | 2 out of 273 genes, 0.7% | 0.007433743 |
| [bicarbonate transport](http://amigo.geneontology.org/amigo/term/GO:0015701) | 2 out of 24 genes, 8.3% | 2 out of 273 genes, 0.7% | 0.007433743 |
| [monocarboxylic acid transport](http://amigo.geneontology.org/amigo/term/GO:0015718) | 2 out of 24 genes, 8.3% | 2 out of 273 genes, 0.7% | 0.007433743 |
| [bile acid and bile salt transport](http://amigo.geneontology.org/amigo/term/GO:0015721) | 2 out of 24 genes, 8.3% | 2 out of 273 genes, 0.7% | 0.007433743 |
| [organic acid transport](http://amigo.geneontology.org/amigo/term/GO:0015849) | 2 out of 24 genes, 8.3% | 2 out of 273 genes, 0.7% | 0.007433743 |
| [regulation of transforming growth factor beta receptor signaling pathway](http://amigo.geneontology.org/amigo/term/GO:0017015) | 2 out of 24 genes, 8.3% | 2 out of 273 genes, 0.7% | 0.007433743 |
| [circadian sleep/wake cycle process](http://amigo.geneontology.org/amigo/term/GO:0022410) | 2 out of 24 genes, 8.3% | 2 out of 273 genes, 0.7% | 0.007433743 |
| [hyaluronan catabolic process](http://amigo.geneontology.org/amigo/term/GO:0030214) | 2 out of 24 genes, 8.3% | 2 out of 273 genes, 0.7% | 0.007433743 |
| [circadian sleep/wake cycle](http://amigo.geneontology.org/amigo/term/GO:0042745) | 2 out of 24 genes, 8.3% | 2 out of 273 genes, 0.7% | 0.007433743 |
| [regulation of circadian sleep/wake cycle](http://amigo.geneontology.org/amigo/term/GO:0042749) | 2 out of 24 genes, 8.3% | 2 out of 273 genes, 0.7% | 0.007433743 |
| [regulation of circadian rhythm](http://amigo.geneontology.org/amigo/term/GO:0042752) | 2 out of 24 genes, 8.3% | 2 out of 273 genes, 0.7% | 0.007433743 |
| [positive regulation of circadian rhythm](http://amigo.geneontology.org/amigo/term/GO:0042753) | 2 out of 24 genes, 8.3% | 2 out of 273 genes, 0.7% | 0.007433743 |
| [receptor metabolic process](http://amigo.geneontology.org/amigo/term/GO:0043112) | 2 out of 24 genes, 8.3% | 2 out of 273 genes, 0.7% | 0.007433743 |
| [sodium-independent organic anion transport](http://amigo.geneontology.org/amigo/term/GO:0043252) | 2 out of 24 genes, 8.3% | 2 out of 273 genes, 0.7% | 0.007433743 |
| [negative regulation of ion transport](http://amigo.geneontology.org/amigo/term/GO:0043271) | 2 out of 24 genes, 8.3% | 2 out of 273 genes, 0.7% | 0.007433743 |
| [positive regulation of blood vessel endothelial cell migration](http://amigo.geneontology.org/amigo/term/GO:0043536) | 2 out of 24 genes, 8.3% | 2 out of 273 genes, 0.7% | 0.007433743 |
| [regulation of circadian sleep/wake cycle, sleep](http://amigo.geneontology.org/amigo/term/GO:0045187) | 2 out of 24 genes, 8.3% | 2 out of 273 genes, 0.7% | 0.007433743 |
| [regulation of circadian sleep/wake cycle, non-REM sleep](http://amigo.geneontology.org/amigo/term/GO:0045188) | 2 out of 24 genes, 8.3% | 2 out of 273 genes, 0.7% | 0.007433743 |
| [negative regulation of cell cycle](http://amigo.geneontology.org/amigo/term/GO:0045786) | 2 out of 24 genes, 8.3% | 2 out of 273 genes, 0.7% | 0.007433743 |
| [positive regulation of circadian sleep/wake cycle, sleep](http://amigo.geneontology.org/amigo/term/GO:0045938) | 2 out of 24 genes, 8.3% | 2 out of 273 genes, 0.7% | 0.007433743 |
| [positive regulation of circadian sleep/wake cycle, non-REM sleep](http://amigo.geneontology.org/amigo/term/GO:0046010) | 2 out of 24 genes, 8.3% | 2 out of 273 genes, 0.7% | 0.007433743 |
| [response to mercury ion](http://amigo.geneontology.org/amigo/term/GO:0046689) | 2 out of 24 genes, 8.3% | 2 out of 273 genes, 0.7% | 0.007433743 |
| [carboxylic acid transport](http://amigo.geneontology.org/amigo/term/GO:0046942) | 2 out of 24 genes, 8.3% | 2 out of 273 genes, 0.7% | 0.007433743 |
| [circadian behavior](http://amigo.geneontology.org/amigo/term/GO:0048512) | 2 out of 24 genes, 8.3% | 2 out of 273 genes, 0.7% | 0.007433743 |
| [organelle localization](http://amigo.geneontology.org/amigo/term/GO:0051640) | 2 out of 24 genes, 8.3% | 2 out of 273 genes, 0.7% | 0.007433743 |
| [mitochondrion localization](http://amigo.geneontology.org/amigo/term/GO:0051646) | 2 out of 24 genes, 8.3% | 2 out of 273 genes, 0.7% | 0.007433743 |
| [maintenance of organelle location](http://amigo.geneontology.org/amigo/term/GO:0051657) | 2 out of 24 genes, 8.3% | 2 out of 273 genes, 0.7% | 0.007433743 |
| [maintenance of mitochondrion location](http://amigo.geneontology.org/amigo/term/GO:0051659) | 2 out of 24 genes, 8.3% | 2 out of 273 genes, 0.7% | 0.007433743 |
| [response to platinum ion](http://amigo.geneontology.org/amigo/term/GO:0070541) | 2 out of 24 genes, 8.3% | 2 out of 273 genes, 0.7% | 0.007433743 |
| [regulation of transmembrane receptor protein serine/threonine kinase signaling pathway](http://amigo.geneontology.org/amigo/term/GO:0090092) | 2 out of 24 genes, 8.3% | 2 out of 273 genes, 0.7% | 0.007433743 |
| [positive regulation of transmembrane receptor protein serine/threonine kinase signaling pathway](http://amigo.geneontology.org/amigo/term/GO:0090100) | 2 out of 24 genes, 8.3% | 2 out of 273 genes, 0.7% | 0.007433743 |
| [establishment of epithelial cell polarity](http://amigo.geneontology.org/amigo/term/GO:0090162) | 2 out of 24 genes, 8.3% | 2 out of 273 genes, 0.7% | 0.007433743 |
| [positive regulation of reproductive process](http://amigo.geneontology.org/amigo/term/GO:2000243) | 2 out of 24 genes, 8.3% | 2 out of 273 genes, 0.7% | 0.007433743 |
| [organic substance transport](http://amigo.geneontology.org/amigo/term/GO:0071702) | 8 out of 24 genes, 33.3% | 37 out of 273 genes, 13.6% | 0.007813079 |
| [cellular metabolic process](http://amigo.geneontology.org/amigo/term/GO:0044237) | 13 out of 24 genes, 54.2% | 82 out of 273 genes, 30.0% | 0.008571683 |
| [cellular response to chemical stimulus](http://amigo.geneontology.org/amigo/term/GO:0070887) | 8 out of 24 genes, 33.3% | 38 out of 273 genes, 13.9% | 0.009324346 |
| [transport](http://amigo.geneontology.org/amigo/term/GO:0006810) | 13 out of 24 genes, 54.2% | 83 out of 273 genes, 30.4% | 0.009649038 |
| [establishment of localization](http://amigo.geneontology.org/amigo/term/GO:0051234) | 13 out of 24 genes, 54.2% | 83 out of 273 genes, 30.4% | 0.009649038 |
| [cartilage development](http://amigo.geneontology.org/amigo/term/GO:0051216) | 3 out of 24 genes, 12.5% | 6 out of 273 genes, 2.2% | 0.01008053 |
| [connective tissue development](http://amigo.geneontology.org/amigo/term/GO:0061448) | 3 out of 24 genes, 12.5% | 6 out of 273 genes, 2.2% | 0.01008053 |
| [regulation of apoptotic process](http://amigo.geneontology.org/amigo/term/GO:0042981) | 6 out of 24 genes, 25.0% | 24 out of 273 genes, 8.8% | 0.01120738 |
| [regulation of programmed cell death](http://amigo.geneontology.org/amigo/term/GO:0043067) | 6 out of 24 genes, 25.0% | 24 out of 273 genes, 8.8% | 0.01120738 |
| [cellular response to stimulus](http://amigo.geneontology.org/amigo/term/GO:0051716) | 12 out of 24 genes, 50.0% | 75 out of 273 genes, 27.5% | 0.01191394 |
| [steroid metabolic process](http://amigo.geneontology.org/amigo/term/GO:0008202) | 5 out of 24 genes, 20.8% | 18 out of 273 genes, 6.6% | 0.01346533 |
| [response to reactive oxygen species](http://amigo.geneontology.org/amigo/term/GO:0000302) | 4 out of 24 genes, 16.7% | 12 out of 273 genes, 4.4% | 0.01421789 |
| [regulation of kinase activity](http://amigo.geneontology.org/amigo/term/GO:0043549) | 4 out of 24 genes, 16.7% | 12 out of 273 genes, 4.4% | 0.01421789 |
| [regulation of protein kinase activity](http://amigo.geneontology.org/amigo/term/GO:0045859) | 4 out of 24 genes, 16.7% | 12 out of 273 genes, 4.4% | 0.01421789 |
| [regulation of protein serine/threonine kinase activity](http://amigo.geneontology.org/amigo/term/GO:0071900) | 4 out of 24 genes, 16.7% | 12 out of 273 genes, 4.4% | 0.01421789 |
| [cellular catabolic process](http://amigo.geneontology.org/amigo/term/GO:0044248) | 7 out of 24 genes, 29.2% | 33 out of 273 genes, 12.1% | 0.01526217 |
| [response to organic substance](http://amigo.geneontology.org/amigo/term/GO:0010033) | 9 out of 24 genes, 37.5% | 50 out of 273 genes, 18.3% | 0.01642944 |
| [positive regulation of kinase activity](http://amigo.geneontology.org/amigo/term/GO:0033674) | 3 out of 24 genes, 12.5% | 7 out of 273 genes, 2.6% | 0.01661595 |
| [modification of morphology or physiology of other organism](http://amigo.geneontology.org/amigo/term/GO:0035821) | 3 out of 24 genes, 12.5% | 7 out of 273 genes, 2.6% | 0.01661595 |
| [response to hydrogen peroxide](http://amigo.geneontology.org/amigo/term/GO:0042542) | 3 out of 24 genes, 12.5% | 7 out of 273 genes, 2.6% | 0.01661595 |
| [hydrogen peroxide metabolic process](http://amigo.geneontology.org/amigo/term/GO:0042743) | 3 out of 24 genes, 12.5% | 7 out of 273 genes, 2.6% | 0.01661595 |
| [hydrogen peroxide catabolic process](http://amigo.geneontology.org/amigo/term/GO:0042744) | 3 out of 24 genes, 12.5% | 7 out of 273 genes, 2.6% | 0.01661595 |
| [cell-cell junction organization](http://amigo.geneontology.org/amigo/term/GO:0045216) | 3 out of 24 genes, 12.5% | 7 out of 273 genes, 2.6% | 0.01661595 |
| [positive regulation of protein kinase activity](http://amigo.geneontology.org/amigo/term/GO:0045860) | 3 out of 24 genes, 12.5% | 7 out of 273 genes, 2.6% | 0.01661595 |
| [regulation of epithelial cell proliferation](http://amigo.geneontology.org/amigo/term/GO:0050678) | 3 out of 24 genes, 12.5% | 7 out of 273 genes, 2.6% | 0.01661595 |
| [regulation of binding](http://amigo.geneontology.org/amigo/term/GO:0051098) | 3 out of 24 genes, 12.5% | 7 out of 273 genes, 2.6% | 0.01661595 |
| [cellular response to hydrogen peroxide](http://amigo.geneontology.org/amigo/term/GO:0070301) | 3 out of 24 genes, 12.5% | 7 out of 273 genes, 2.6% | 0.01661595 |
| [positive regulation of protein serine/threonine kinase activity](http://amigo.geneontology.org/amigo/term/GO:0071902) | 3 out of 24 genes, 12.5% | 7 out of 273 genes, 2.6% | 0.01661595 |
| [response to wounding](http://amigo.geneontology.org/amigo/term/GO:0009611) | 12 out of 24 genes, 50.0% | 79 out of 273 genes, 28.9% | 0.01876946 |
| [blood coagulation](http://amigo.geneontology.org/amigo/term/GO:0007596) | 10 out of 24 genes, 41.7% | 60 out of 273 genes, 22.0% | 0.01885270 |
| [hemostasis](http://amigo.geneontology.org/amigo/term/GO:0007599) | 10 out of 24 genes, 41.7% | 60 out of 273 genes, 22.0% | 0.01885270 |
| [coagulation](http://amigo.geneontology.org/amigo/term/GO:0050817) | 10 out of 24 genes, 41.7% | 60 out of 273 genes, 22.0% | 0.01885270 |
| [response to extracellular stimulus](http://amigo.geneontology.org/amigo/term/GO:0009991) | 4 out of 24 genes, 16.7% | 13 out of 273 genes, 4.8% | 0.01931343 |
| [response to nutrient levels](http://amigo.geneontology.org/amigo/term/GO:0031667) | 4 out of 24 genes, 16.7% | 13 out of 273 genes, 4.8% | 0.01931343 |
| [negative regulation of apoptotic process](http://amigo.geneontology.org/amigo/term/GO:0043066) | 4 out of 24 genes, 16.7% | 13 out of 273 genes, 4.8% | 0.01931343 |
| [negative regulation of programmed cell death](http://amigo.geneontology.org/amigo/term/GO:0043069) | 4 out of 24 genes, 16.7% | 13 out of 273 genes, 4.8% | 0.01931343 |
| [secretion](http://amigo.geneontology.org/amigo/term/GO:0046903) | 8 out of 24 genes, 33.3% | 43 out of 273 genes, 15.8% | 0.02048283 |
| [cytolysis by symbiont of host cells](http://amigo.geneontology.org/amigo/term/GO:0001897) | 2 out of 24 genes, 8.3% | 3 out of 273 genes, 1.1% | 0.02109427 |
| [killing by symbiont of host cells](http://amigo.geneontology.org/amigo/term/GO:0001907) | 2 out of 24 genes, 8.3% | 3 out of 273 genes, 1.1% | 0.02109427 |
| [cell-cell junction assembly](http://amigo.geneontology.org/amigo/term/GO:0007043) | 2 out of 24 genes, 8.3% | 3 out of 273 genes, 1.1% | 0.02109427 |
| [establishment or maintenance of cell polarity](http://amigo.geneontology.org/amigo/term/GO:0007163) | 2 out of 24 genes, 8.3% | 3 out of 273 genes, 1.1% | 0.02109427 |
| [regulation of mitotic cell cycle](http://amigo.geneontology.org/amigo/term/GO:0007346) | 2 out of 24 genes, 8.3% | 3 out of 273 genes, 1.1% | 0.02109427 |
| [cellular response to starvation](http://amigo.geneontology.org/amigo/term/GO:0009267) | 2 out of 24 genes, 8.3% | 3 out of 273 genes, 1.1% | 0.02109427 |
| [regulation of cell morphogenesis involved in differentiation](http://amigo.geneontology.org/amigo/term/GO:0010769) | 2 out of 24 genes, 8.3% | 3 out of 273 genes, 1.1% | 0.02109427 |
| [transmission of nerve impulse](http://amigo.geneontology.org/amigo/term/GO:0019226) | 2 out of 24 genes, 8.3% | 3 out of 273 genes, 1.1% | 0.02109427 |
| [hemolysis by symbiont of host erythrocytes](http://amigo.geneontology.org/amigo/term/GO:0019836) | 2 out of 24 genes, 8.3% | 3 out of 273 genes, 1.1% | 0.02109427 |
| [establishment of cell polarity](http://amigo.geneontology.org/amigo/term/GO:0030010) | 2 out of 24 genes, 8.3% | 3 out of 273 genes, 1.1% | 0.02109427 |
| [cellular response to extracellular stimulus](http://amigo.geneontology.org/amigo/term/GO:0031668) | 2 out of 24 genes, 8.3% | 3 out of 273 genes, 1.1% | 0.02109427 |
| [cellular response to nutrient levels](http://amigo.geneontology.org/amigo/term/GO:0031669) | 2 out of 24 genes, 8.3% | 3 out of 273 genes, 1.1% | 0.02109427 |
| [response to progesterone stimulus](http://amigo.geneontology.org/amigo/term/GO:0032570) | 2 out of 24 genes, 8.3% | 3 out of 273 genes, 1.1% | 0.02109427 |
| [multicellular organismal signaling](http://amigo.geneontology.org/amigo/term/GO:0035637) | 2 out of 24 genes, 8.3% | 3 out of 273 genes, 1.1% | 0.02109427 |
| [apical junction assembly](http://amigo.geneontology.org/amigo/term/GO:0043297) | 2 out of 24 genes, 8.3% | 3 out of 273 genes, 1.1% | 0.02109427 |
| [positive regulation of MAP kinase activity](http://amigo.geneontology.org/amigo/term/GO:0043406) | 2 out of 24 genes, 8.3% | 3 out of 273 genes, 1.1% | 0.02109427 |
| [disruption by symbiont of host cell](http://amigo.geneontology.org/amigo/term/GO:0044004) | 2 out of 24 genes, 8.3% | 3 out of 273 genes, 1.1% | 0.02109427 |
| [hemolysis in other organism](http://amigo.geneontology.org/amigo/term/GO:0044179) | 2 out of 24 genes, 8.3% | 3 out of 273 genes, 1.1% | 0.02109427 |
| [single-organism behavior](http://amigo.geneontology.org/amigo/term/GO:0044708) | 2 out of 24 genes, 8.3% | 3 out of 273 genes, 1.1% | 0.02109427 |
| [regulation of nitric oxide biosynthetic process](http://amigo.geneontology.org/amigo/term/GO:0045428) | 2 out of 24 genes, 8.3% | 3 out of 273 genes, 1.1% | 0.02109427 |
| [positive regulation of nitric oxide biosynthetic process](http://amigo.geneontology.org/amigo/term/GO:0045429) | 2 out of 24 genes, 8.3% | 3 out of 273 genes, 1.1% | 0.02109427 |
| [protein heterooligomerization](http://amigo.geneontology.org/amigo/term/GO:0051291) | 2 out of 24 genes, 8.3% | 3 out of 273 genes, 1.1% | 0.02109427 |
| [cytolysis in other organism](http://amigo.geneontology.org/amigo/term/GO:0051715) | 2 out of 24 genes, 8.3% | 3 out of 273 genes, 1.1% | 0.02109427 |
| [cytolysis in other organism involved in symbiotic interaction](http://amigo.geneontology.org/amigo/term/GO:0051801) | 2 out of 24 genes, 8.3% | 3 out of 273 genes, 1.1% | 0.02109427 |
| [regulation of protein kinase B signaling cascade](http://amigo.geneontology.org/amigo/term/GO:0051896) | 2 out of 24 genes, 8.3% | 3 out of 273 genes, 1.1% | 0.02109427 |
| [positive regulation of protein kinase B signaling cascade](http://amigo.geneontology.org/amigo/term/GO:0051897) | 2 out of 24 genes, 8.3% | 3 out of 273 genes, 1.1% | 0.02109427 |
| [hemolysis in other organism involved in symbiotic interaction](http://amigo.geneontology.org/amigo/term/GO:0052331) | 2 out of 24 genes, 8.3% | 3 out of 273 genes, 1.1% | 0.02109427 |
| [anatomical structure homeostasis](http://amigo.geneontology.org/amigo/term/GO:0060249) | 2 out of 24 genes, 8.3% | 3 out of 273 genes, 1.1% | 0.02109427 |
| [bone development](http://amigo.geneontology.org/amigo/term/GO:0060348) | 2 out of 24 genes, 8.3% | 3 out of 273 genes, 1.1% | 0.02109427 |
| [bone morphogenesis](http://amigo.geneontology.org/amigo/term/GO:0060349) | 2 out of 24 genes, 8.3% | 3 out of 273 genes, 1.1% | 0.02109427 |
| [developmental growth involved in morphogenesis](http://amigo.geneontology.org/amigo/term/GO:0060560) | 2 out of 24 genes, 8.3% | 3 out of 273 genes, 1.1% | 0.02109427 |
| [positive regulation of cell cycle process](http://amigo.geneontology.org/amigo/term/GO:0090068) | 2 out of 24 genes, 8.3% | 3 out of 273 genes, 1.1% | 0.02109427 |
| [positive regulation of cellular biosynthetic process](http://amigo.geneontology.org/amigo/term/GO:0031328) | 5 out of 24 genes, 20.8% | 20 out of 273 genes, 7.3% | 0.02153028 |
| [positive regulation of cell migration](http://amigo.geneontology.org/amigo/term/GO:0030335) | 3 out of 24 genes, 12.5% | 8 out of 273 genes, 2.9% | 0.02504319 |
| [cellular response to oxidative stress](http://amigo.geneontology.org/amigo/term/GO:0034599) | 3 out of 24 genes, 12.5% | 8 out of 273 genes, 2.9% | 0.02504319 |
| [cellular response to reactive oxygen species](http://amigo.geneontology.org/amigo/term/GO:0034614) | 3 out of 24 genes, 12.5% | 8 out of 273 genes, 2.9% | 0.02504319 |
| [positive regulation of locomotion](http://amigo.geneontology.org/amigo/term/GO:0040017) | 3 out of 24 genes, 12.5% | 8 out of 273 genes, 2.9% | 0.02504319 |
| [positive regulation of cellular component movement](http://amigo.geneontology.org/amigo/term/GO:0051272) | 3 out of 24 genes, 12.5% | 8 out of 273 genes, 2.9% | 0.02504319 |
| [reactive oxygen species metabolic process](http://amigo.geneontology.org/amigo/term/GO:0072593) | 3 out of 24 genes, 12.5% | 8 out of 273 genes, 2.9% | 0.02504319 |
| [response to ketone](http://amigo.geneontology.org/amigo/term/GO:1901654) | 3 out of 24 genes, 12.5% | 8 out of 273 genes, 2.9% | 0.02504319 |
| [positive regulation of cell motility](http://amigo.geneontology.org/amigo/term/GO:2000147) | 3 out of 24 genes, 12.5% | 8 out of 273 genes, 2.9% | 0.02504319 |
| [regulation of transferase activity](http://amigo.geneontology.org/amigo/term/GO:0051338) | 4 out of 24 genes, 16.7% | 14 out of 273 genes, 5.1% | 0.02542806 |
| [negative regulation of cell death](http://amigo.geneontology.org/amigo/term/GO:0060548) | 4 out of 24 genes, 16.7% | 14 out of 273 genes, 5.1% | 0.02542806 |
| [organonitrogen compound biosynthetic process](http://amigo.geneontology.org/amigo/term/GO:1901566) | 4 out of 24 genes, 16.7% | 14 out of 273 genes, 5.1% | 0.02542806 |
| [negative regulation of cellular process](http://amigo.geneontology.org/amigo/term/GO:0048523) | 10 out of 24 genes, 41.7% | 63 out of 273 genes, 23.1% | 0.02683416 |
| [regulation of body fluid levels](http://amigo.geneontology.org/amigo/term/GO:0050878) | 10 out of 24 genes, 41.7% | 63 out of 273 genes, 23.1% | 0.02683416 |
| [platelet degranulation](http://amigo.geneontology.org/amigo/term/GO:0002576) | 7 out of 24 genes, 29.2% | 37 out of 273 genes, 13.6% | 0.02879302 |
| [exocytosis](http://amigo.geneontology.org/amigo/term/GO:0006887) | 7 out of 24 genes, 29.2% | 37 out of 273 genes, 13.6% | 0.02879302 |
| [homeostatic process](http://amigo.geneontology.org/amigo/term/GO:0042592) | 7 out of 24 genes, 29.2% | 37 out of 273 genes, 13.6% | 0.02879302 |
| [organonitrogen compound metabolic process](http://amigo.geneontology.org/amigo/term/GO:1901564) | 7 out of 24 genes, 29.2% | 37 out of 273 genes, 13.6% | 0.02879302 |
| [response to oxygen-containing compound](http://amigo.geneontology.org/amigo/term/GO:1901700) | 7 out of 24 genes, 29.2% | 37 out of 273 genes, 13.6% | 0.02879302 |
| [wound healing](http://amigo.geneontology.org/amigo/term/GO:0042060) | 10 out of 24 genes, 41.7% | 64 out of 273 genes, 23.4% | 0.02999303 |
| [localization](http://amigo.geneontology.org/amigo/term/GO:0051179) | 13 out of 24 genes, 54.2% | 94 out of 273 genes, 34.4% | 0.03059452 |
| [positive regulation of biosynthetic process](http://amigo.geneontology.org/amigo/term/GO:0009891) | 5 out of 24 genes, 20.8% | 22 out of 273 genes, 8.1% | 0.03230763 |
| [regulation of anatomical structure morphogenesis](http://amigo.geneontology.org/amigo/term/GO:0022603) | 5 out of 24 genes, 20.8% | 22 out of 273 genes, 8.1% | 0.03230763 |
| [organic acid metabolic process](http://amigo.geneontology.org/amigo/term/GO:0006082) | 4 out of 24 genes, 16.7% | 15 out of 273 genes, 5.5% | 0.03260938 |
| [response to abiotic stimulus](http://amigo.geneontology.org/amigo/term/GO:0009628) | 4 out of 24 genes, 16.7% | 15 out of 273 genes, 5.5% | 0.03260938 |
| [oxoacid metabolic process](http://amigo.geneontology.org/amigo/term/GO:0043436) | 4 out of 24 genes, 16.7% | 15 out of 273 genes, 5.5% | 0.03260938 |
| [positive regulation of developmental process](http://amigo.geneontology.org/amigo/term/GO:0051094) | 4 out of 24 genes, 16.7% | 15 out of 273 genes, 5.5% | 0.03260938 |
| [secretion by cell](http://amigo.geneontology.org/amigo/term/GO:0032940) | 7 out of 24 genes, 29.2% | 38 out of 273 genes, 13.9% | 0.03318917 |
| [response to inorganic substance](http://amigo.geneontology.org/amigo/term/GO:0010035) | 6 out of 24 genes, 25.0% | 30 out of 273 genes, 11.0% | 0.03413638 |
| [cell junction organization](http://amigo.geneontology.org/amigo/term/GO:0034330) | 3 out of 24 genes, 12.5% | 9 out of 273 genes, 3.3% | 0.03538909 |
| [regulation of MAP kinase activity](http://amigo.geneontology.org/amigo/term/GO:0043405) | 3 out of 24 genes, 12.5% | 9 out of 273 genes, 3.3% | 0.03538909 |
| [cellular nitrogen compound biosynthetic process](http://amigo.geneontology.org/amigo/term/GO:0044271) | 3 out of 24 genes, 12.5% | 9 out of 273 genes, 3.3% | 0.03538909 |
| [positive regulation of transferase activity](http://amigo.geneontology.org/amigo/term/GO:0051347) | 3 out of 24 genes, 12.5% | 9 out of 273 genes, 3.3% | 0.03538909 |
| [transmembrane transport](http://amigo.geneontology.org/amigo/term/GO:0055085) | 3 out of 24 genes, 12.5% | 9 out of 273 genes, 3.3% | 0.03538909 |
| [cellular response to cytokine stimulus](http://amigo.geneontology.org/amigo/term/GO:0071345) | 3 out of 24 genes, 12.5% | 9 out of 273 genes, 3.3% | 0.03538909 |
| [catabolic process](http://amigo.geneontology.org/amigo/term/GO:0009056) | 7 out of 24 genes, 29.2% | 39 out of 273 genes, 14.3% | 0.03803196 |
| [vesicle-mediated transport](http://amigo.geneontology.org/amigo/term/GO:0016192) | 8 out of 24 genes, 33.3% | 48 out of 273 genes, 17.6% | 0.0393466 |
| [negative regulation of macromolecule metabolic process](http://amigo.geneontology.org/amigo/term/GO:0010605) | 6 out of 24 genes, 25.0% | 31 out of 273 genes, 11.4% | 0.03978269 |
| [aminoglycan catabolic process](http://amigo.geneontology.org/amigo/term/GO:0006026) | 2 out of 24 genes, 8.3% | 4 out of 273 genes, 1.5% | 0.03991545 |
| [glycosaminoglycan catabolic process](http://amigo.geneontology.org/amigo/term/GO:0006027) | 2 out of 24 genes, 8.3% | 4 out of 273 genes, 1.5% | 0.03991545 |
| [digestion](http://amigo.geneontology.org/amigo/term/GO:0007586) | 2 out of 24 genes, 8.3% | 4 out of 273 genes, 1.5% | 0.03991545 |
| [behavior](http://amigo.geneontology.org/amigo/term/GO:0007610) | 2 out of 24 genes, 8.3% | 4 out of 273 genes, 1.5% | 0.03991545 |
| [regulation of cell cycle process](http://amigo.geneontology.org/amigo/term/GO:0010564) | 2 out of 24 genes, 8.3% | 4 out of 273 genes, 1.5% | 0.03991545 |
| [positive regulation of endothelial cell migration](http://amigo.geneontology.org/amigo/term/GO:0010595) | 2 out of 24 genes, 8.3% | 4 out of 273 genes, 1.5% | 0.03991545 |
| [regulation of protein dephosphorylation](http://amigo.geneontology.org/amigo/term/GO:0035304) | 2 out of 24 genes, 8.3% | 4 out of 273 genes, 1.5% | 0.03991545 |
| [positive regulation of dephosphorylation](http://amigo.geneontology.org/amigo/term/GO:0035306) | 2 out of 24 genes, 8.3% | 4 out of 273 genes, 1.5% | 0.03991545 |
| [positive regulation of protein dephosphorylation](http://amigo.geneontology.org/amigo/term/GO:0035307) | 2 out of 24 genes, 8.3% | 4 out of 273 genes, 1.5% | 0.03991545 |
| [response to starvation](http://amigo.geneontology.org/amigo/term/GO:0042594) | 2 out of 24 genes, 8.3% | 4 out of 273 genes, 1.5% | 0.03991545 |
| [regulation of ion transport](http://amigo.geneontology.org/amigo/term/GO:0043269) | 2 out of 24 genes, 8.3% | 4 out of 273 genes, 1.5% | 0.03991545 |
| [regulation of blood vessel endothelial cell migration](http://amigo.geneontology.org/amigo/term/GO:0043535) | 2 out of 24 genes, 8.3% | 4 out of 273 genes, 1.5% | 0.03991545 |
| [negative regulation of blood vessel endothelial cell migration](http://amigo.geneontology.org/amigo/term/GO:0043537) | 2 out of 24 genes, 8.3% | 4 out of 273 genes, 1.5% | 0.03991545 |
| [positive regulation of angiogenesis](http://amigo.geneontology.org/amigo/term/GO:0045766) | 2 out of 24 genes, 8.3% | 4 out of 273 genes, 1.5% | 0.03991545 |
| [rhythmic process](http://amigo.geneontology.org/amigo/term/GO:0048511) | 2 out of 24 genes, 8.3% | 4 out of 273 genes, 1.5% | 0.03991545 |
| [skeletal system morphogenesis](http://amigo.geneontology.org/amigo/term/GO:0048705) | 2 out of 24 genes, 8.3% | 4 out of 273 genes, 1.5% | 0.03991545 |
| [positive regulation of epithelial cell proliferation](http://amigo.geneontology.org/amigo/term/GO:0050679) | 2 out of 24 genes, 8.3% | 4 out of 273 genes, 1.5% | 0.03991545 |
| [negative regulation of epithelial cell proliferation](http://amigo.geneontology.org/amigo/term/GO:0050680) | 2 out of 24 genes, 8.3% | 4 out of 273 genes, 1.5% | 0.03991545 |
| [positive regulation of chemotaxis](http://amigo.geneontology.org/amigo/term/GO:0050921) | 2 out of 24 genes, 8.3% | 4 out of 273 genes, 1.5% | 0.03991545 |
| [maintenance of location](http://amigo.geneontology.org/amigo/term/GO:0051235) | 2 out of 24 genes, 8.3% | 4 out of 273 genes, 1.5% | 0.03991545 |
| [maintenance of location in cell](http://amigo.geneontology.org/amigo/term/GO:0051651) | 2 out of 24 genes, 8.3% | 4 out of 273 genes, 1.5% | 0.03991545 |
| [disruption of cells of other organism involved in symbiotic interaction](http://amigo.geneontology.org/amigo/term/GO:0051818) | 2 out of 24 genes, 8.3% | 4 out of 273 genes, 1.5% | 0.03991545 |
| [killing of cells in other organism involved in symbiotic interaction](http://amigo.geneontology.org/amigo/term/GO:0051883) | 2 out of 24 genes, 8.3% | 4 out of 273 genes, 1.5% | 0.03991545 |
| [cellular response to external stimulus](http://amigo.geneontology.org/amigo/term/GO:0071496) | 2 out of 24 genes, 8.3% | 4 out of 273 genes, 1.5% | 0.03991545 |
| [negative regulation of cellular response to growth factor stimulus](http://amigo.geneontology.org/amigo/term/GO:0090288) | 2 out of 24 genes, 8.3% | 4 out of 273 genes, 1.5% | 0.03991545 |
| [regulation of reproductive process](http://amigo.geneontology.org/amigo/term/GO:2000241) | 2 out of 24 genes, 8.3% | 4 out of 273 genes, 1.5% | 0.03991545 |
| [system process](http://amigo.geneontology.org/amigo/term/GO:0003008) | 4 out of 24 genes, 16.7% | 16 out of 273 genes, 5.9% | 0.04089017 |
| [response to oxidative stress](http://amigo.geneontology.org/amigo/term/GO:0006979) | 4 out of 24 genes, 16.7% | 16 out of 273 genes, 5.9% | 0.04089017 |
| [generation of neurons](http://amigo.geneontology.org/amigo/term/GO:0048699) | 4 out of 24 genes, 16.7% | 16 out of 273 genes, 5.9% | 0.04089017 |
| [response to chemical stimulus](http://amigo.geneontology.org/amigo/term/GO:0042221) | 11 out of 24 genes, 45.8% | 77 out of 273 genes, 28.2% | 0.04196771 |
| [platelet activation](http://amigo.geneontology.org/amigo/term/GO:0030168) | 7 out of 24 genes, 29.2% | 40 out of 273 genes, 14.7% | 0.04333945 |
| [positive regulation of catalytic activity](http://amigo.geneontology.org/amigo/term/GO:0043085) | 5 out of 24 genes, 20.8% | 24 out of 273 genes, 8.8% | 0.04606022 |
| [regulation of cellular component biogenesis](http://amigo.geneontology.org/amigo/term/GO:0044087) | 3 out of 24 genes, 12.5% | 10 out of 273 genes, 3.7% | 0.04763286 |
| [regulation of angiogenesis](http://amigo.geneontology.org/amigo/term/GO:0045765) | 3 out of 24 genes, 12.5% | 10 out of 273 genes, 3.7% | 0.04763286 |
| [interaction with host](http://amigo.geneontology.org/amigo/term/GO:0051701) | 3 out of 24 genes, 12.5% | 10 out of 273 genes, 3.7% | 0.04763286 |

**Fig S1. Annotated spectrum for 54304028 - glyceraldehyde-3-phosphate dehydrogenase**

**
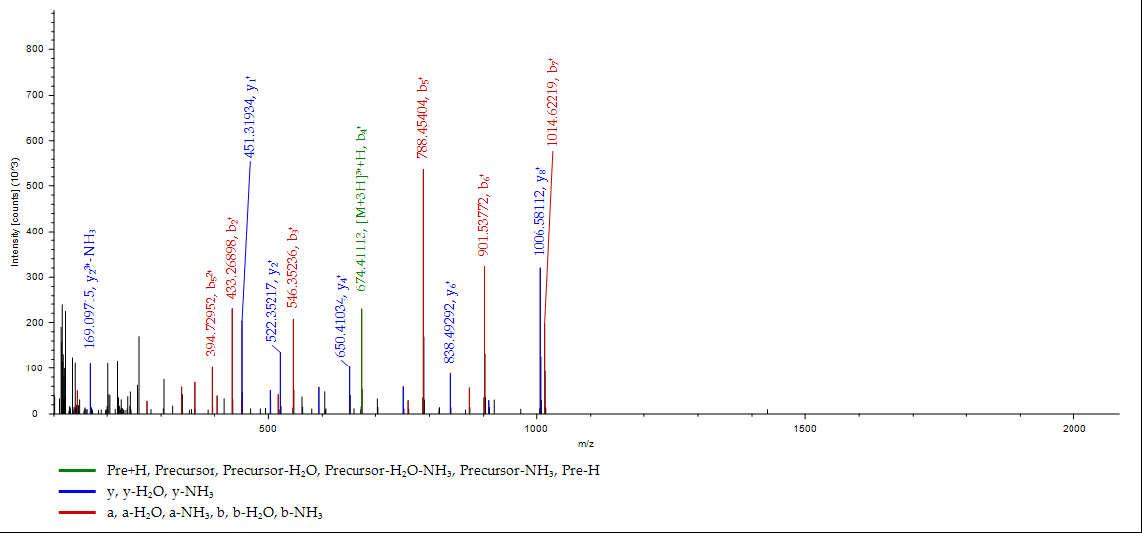
**

**Fig S2A. Annotated spectrum for 3152372 - anti-FactorVIII scFv [Homo sapiens]**

**
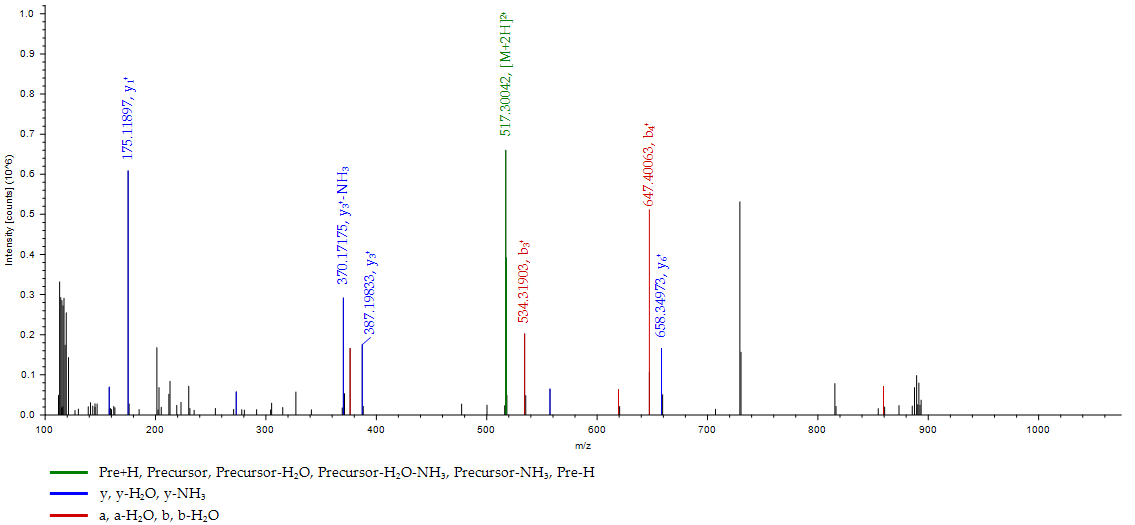
**

**Fig S2B. Annotated spectrum for 3152372 - anti-FactorVIII scFv [Homo sapiens]**

**
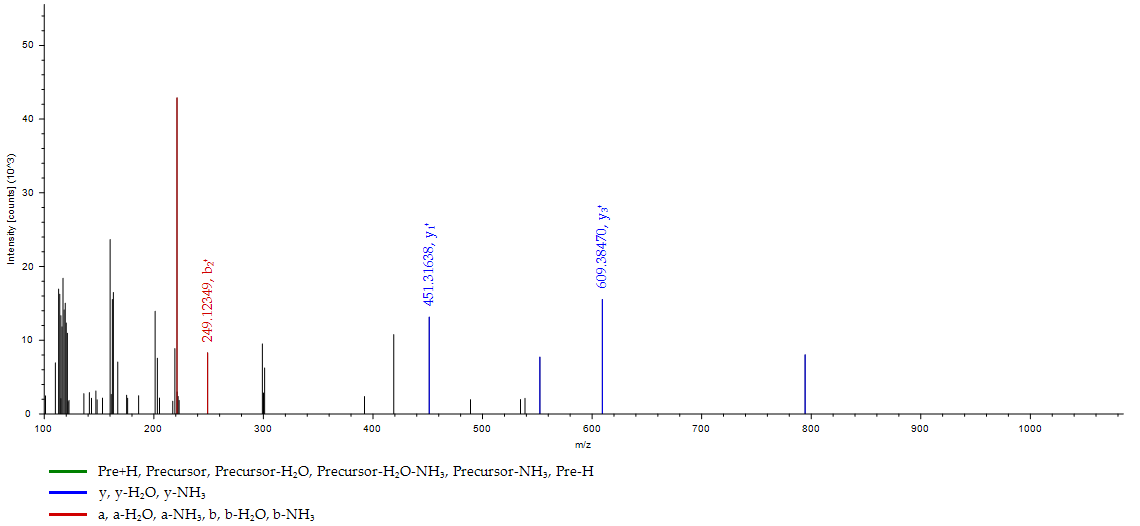
**

**Fig S2C. Annotated spectrum for 3152372 - anti-FactorVIII scFv [Homo sapiens]**

**
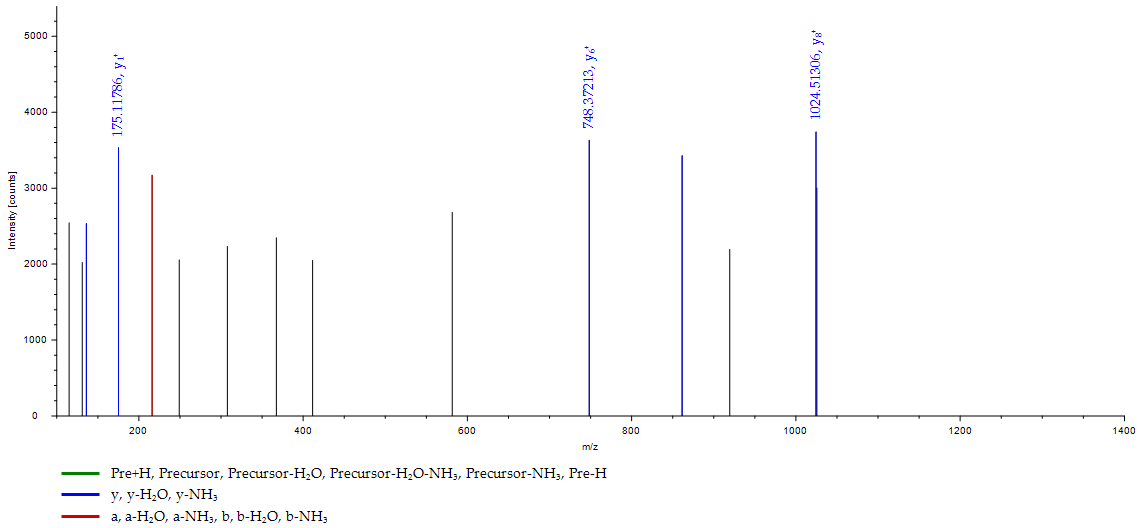
**

**Fig S2D. Annotated spectrum for 3152372 - anti-FactorVIII scFv [Homo sapiens]**

**
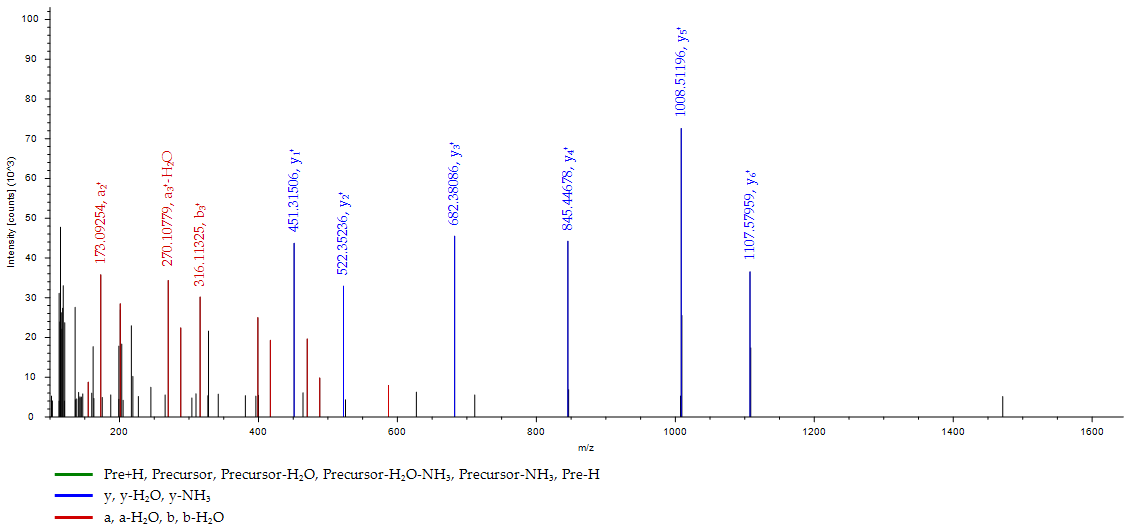
**

**Fig S2E. Annotated spectrum for 3152372 - anti-FactorVIII scFv [Homo sapiens]**

**
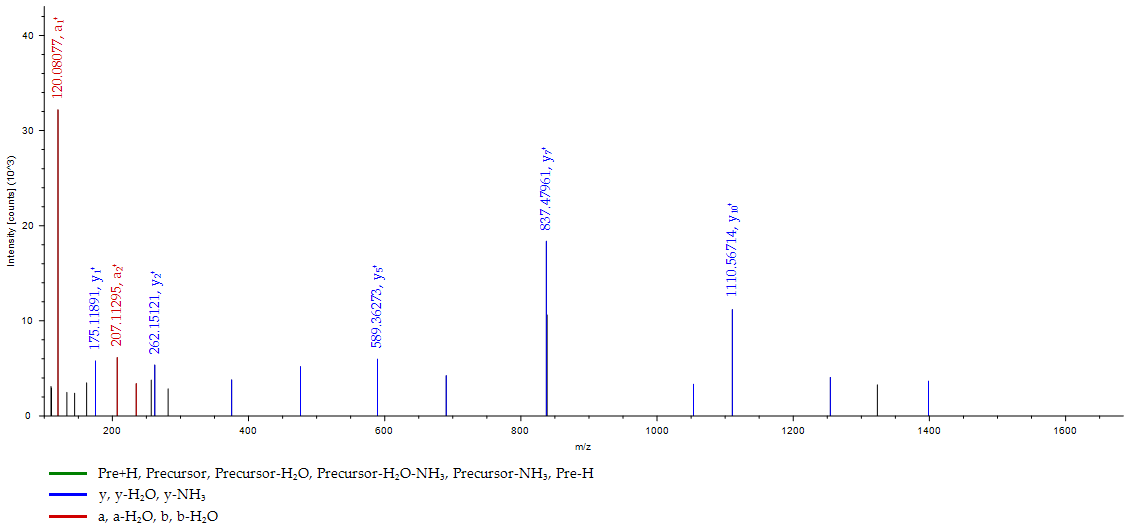
**

**Fig S2F. Annotated spectrum for 3152372 - anti-FactorVIII scFv [Homo sapiens]**

**
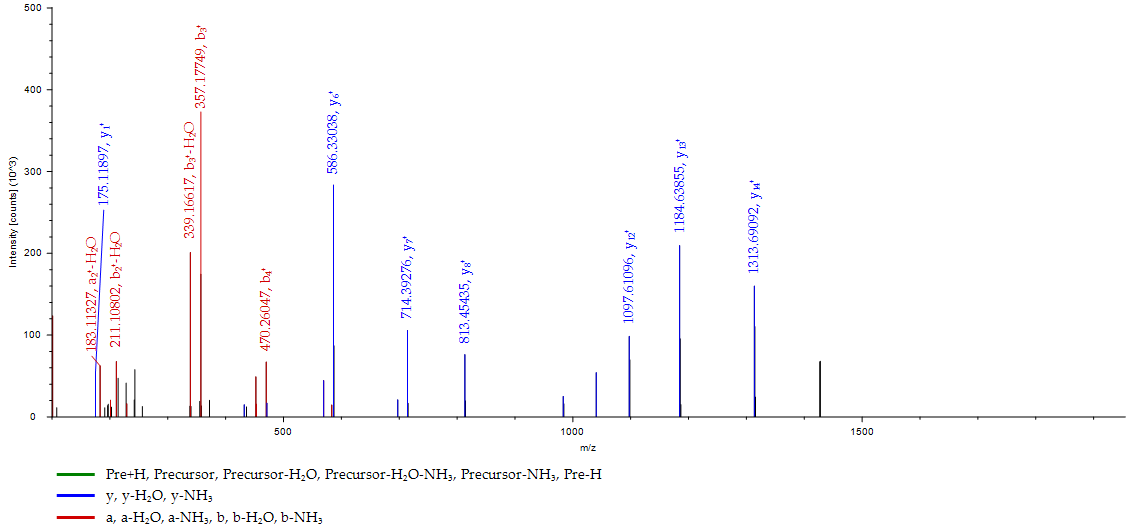
**

**Fig S2G. Annotated spectrum for 3152372 - anti-FactorVIII scFv [Homo sapiens]**

**
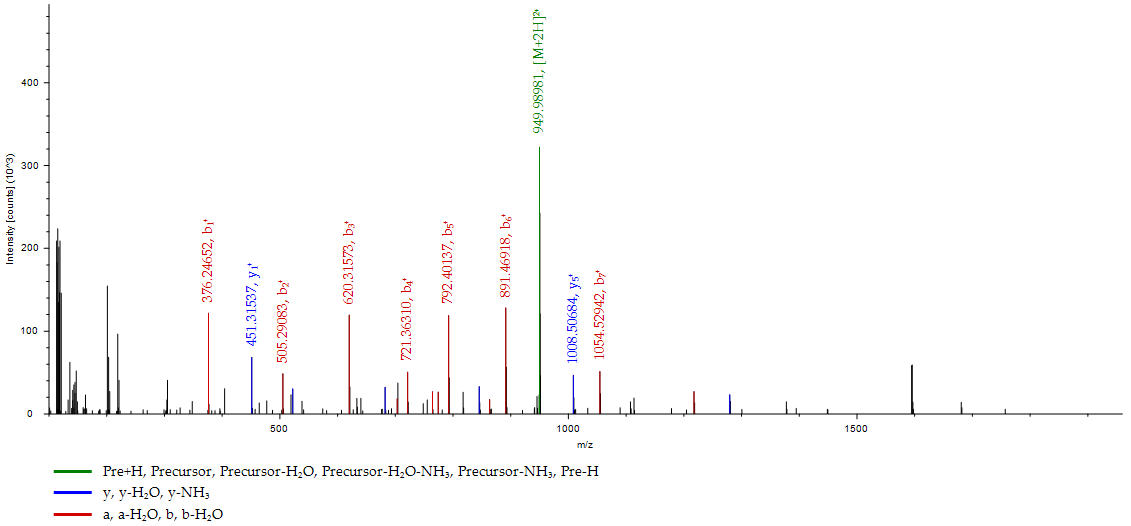
**

**Fig S2H. Annotated spectrum for 3152372 - anti-FactorVIII scFv [Homo sapiens]**

**
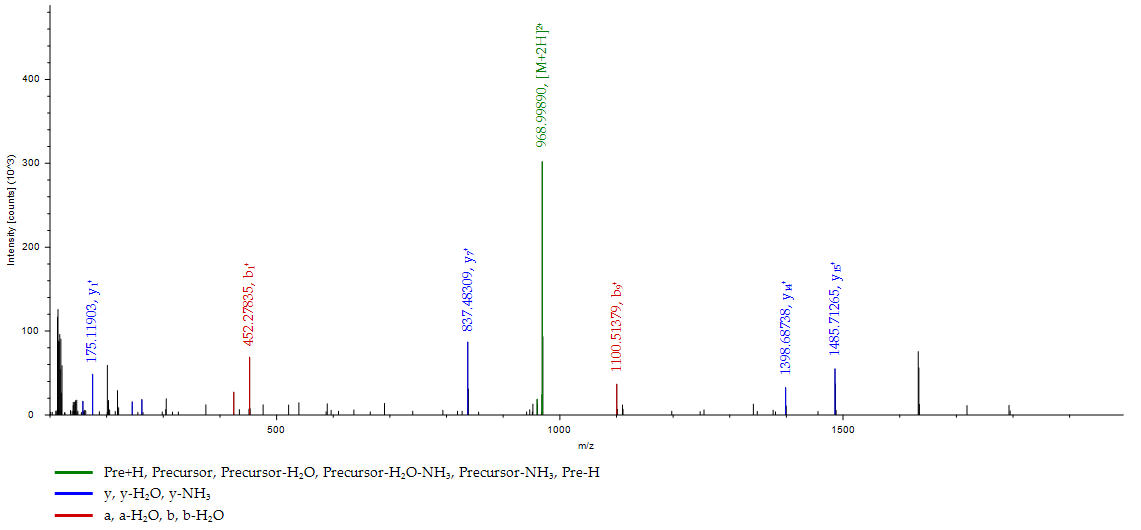
**

**Fig S2I. Annotated spectrum for 3152372 - anti-FactorVIII scFv [Homo sapiens]**

**
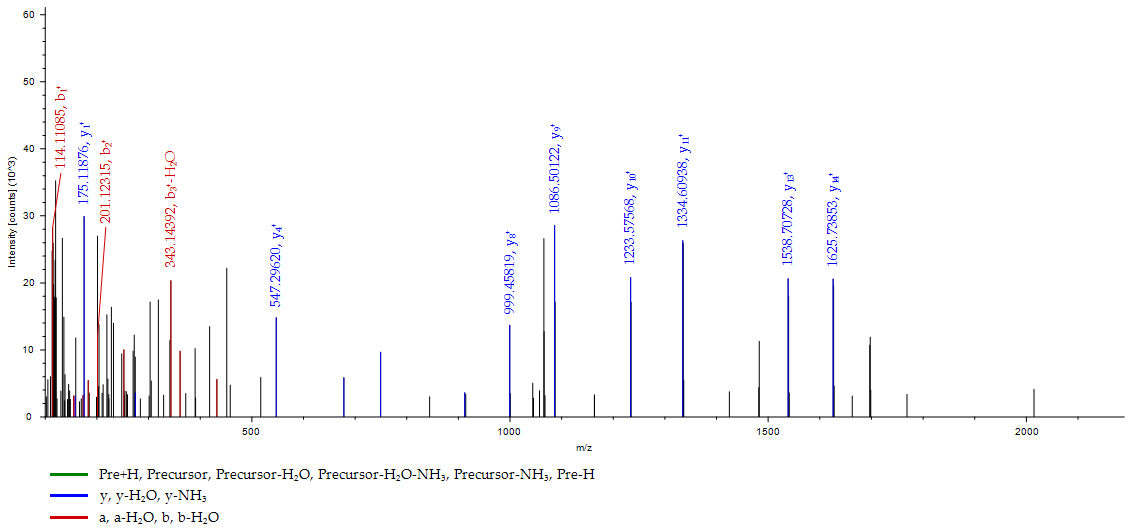
**

**Fig S2J. Annotated spectrum for 3152372 - anti-FactorVIII scFv [Homo sapiens]**

**
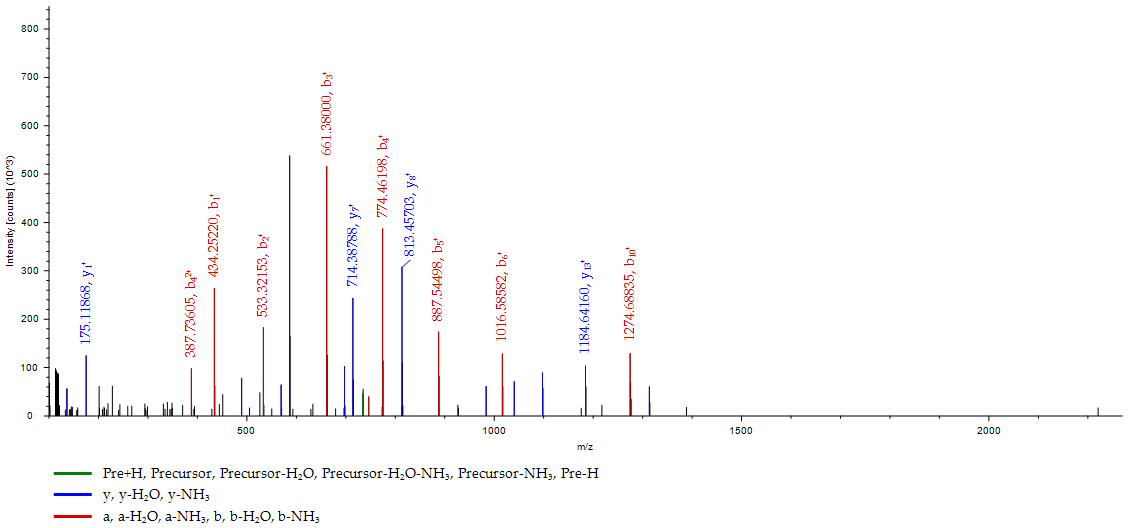
**

**Fig S2K. Annotated spectrum for 3152372 - anti-FactorVIII scFv [Homo sapiens]**

**
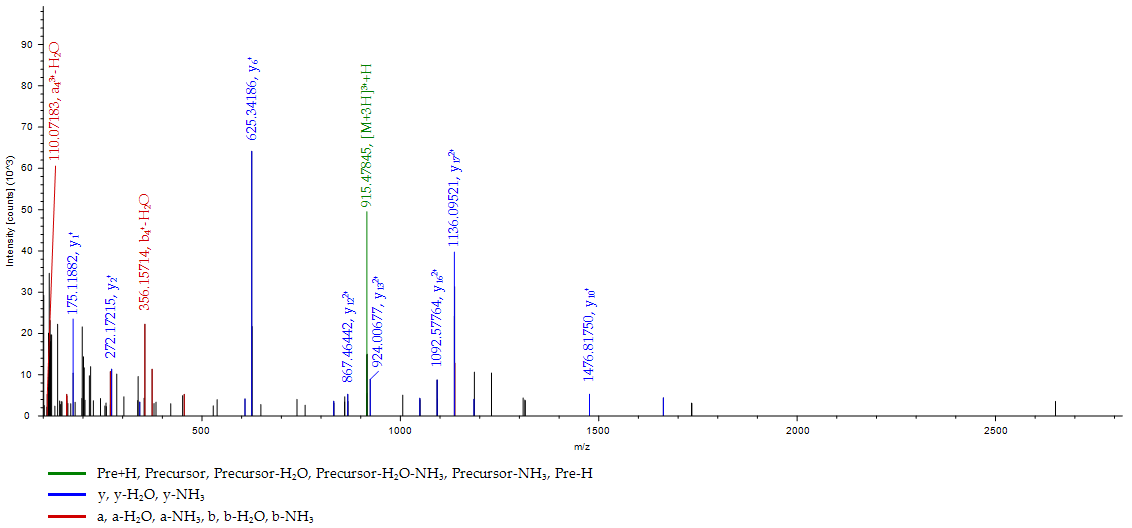
**

**Fig S2L. Annotated spectrum for 3152372 - anti-FactorVIII scFv [Homo sapiens]**

**
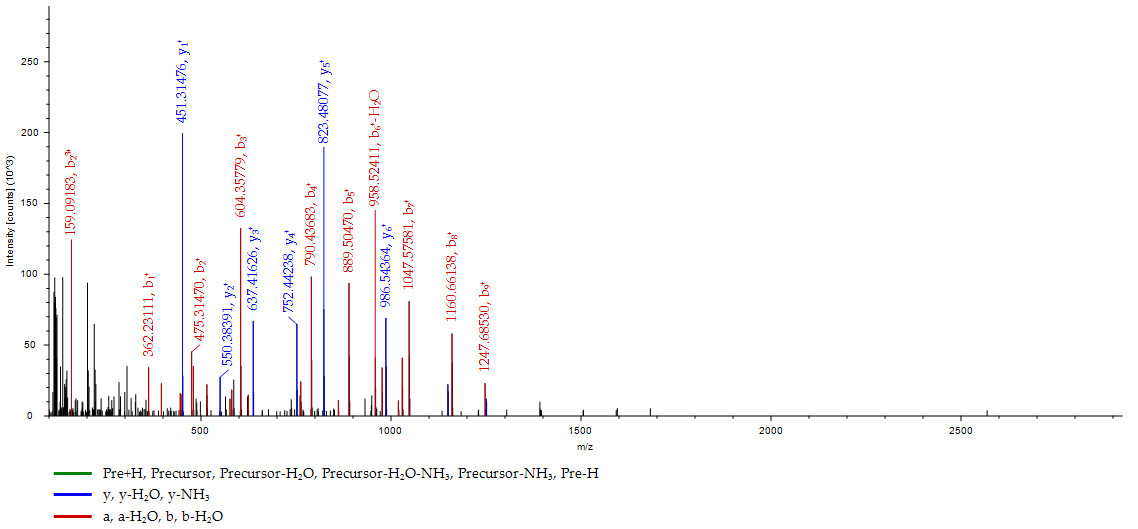
**

**Fig S2M. Annotated spectrum for 3152372 - anti-FactorVIII scFv [Homo sapiens]**

**
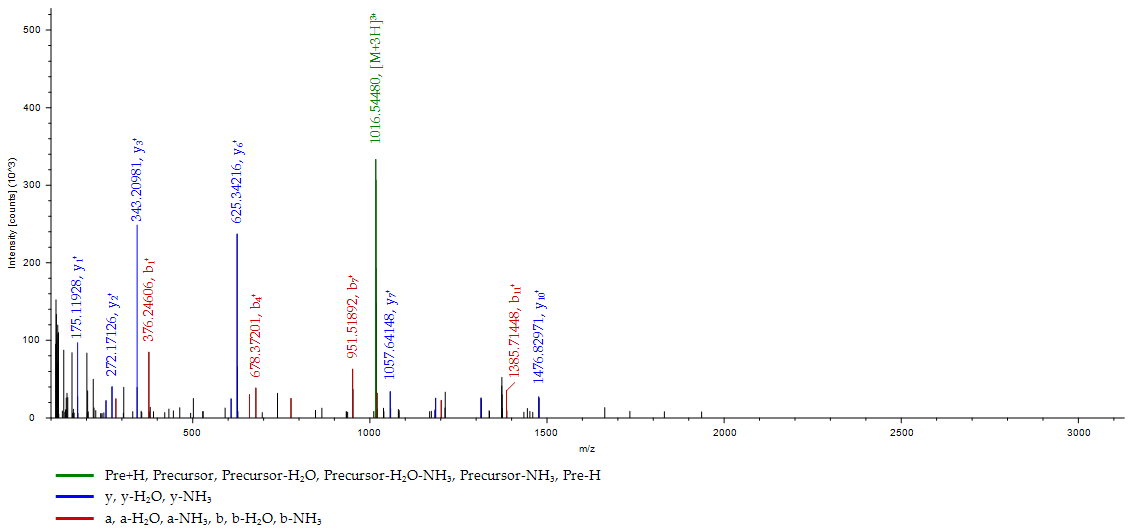
**

**Fig S2N. Annotated spectrum for 3152372 - anti-FactorVIII scFv [Homo sapiens]**

**
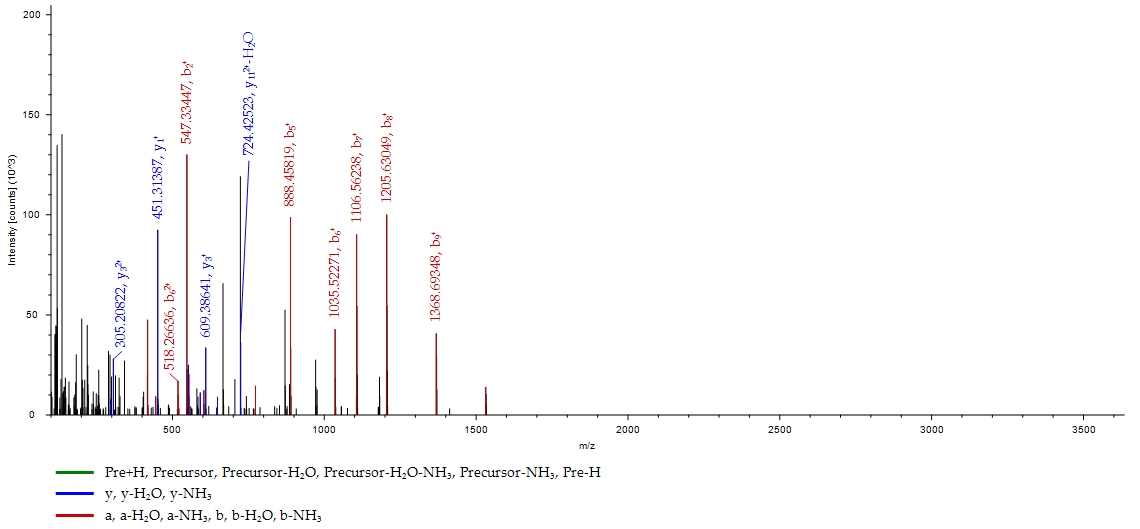
**

**Fig S3. Annotated spectrum for 56378229 - carbamoylphosphate synthetase I [Homo sapiens]**

**
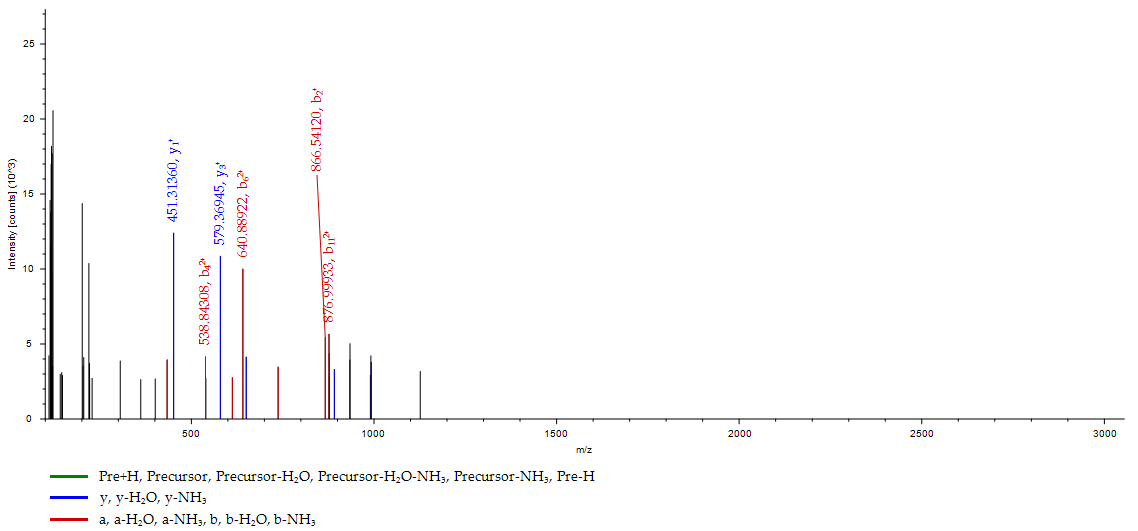
**

**Fig S4A. Annotated spectrum for 11122875 - glycosylphosphatidylinositol phospholipase D [Homo sapiens]**

**
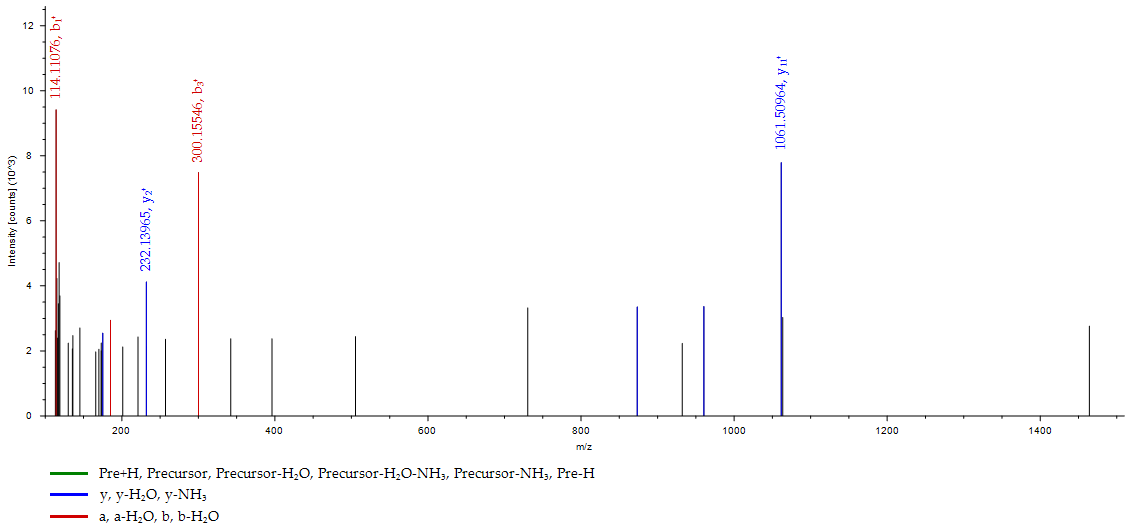
**

**Fig S4B. Annotated spectrum for 11122875 - glycosylphosphatidylinositol phospholipase D [Homo sapiens]**

**
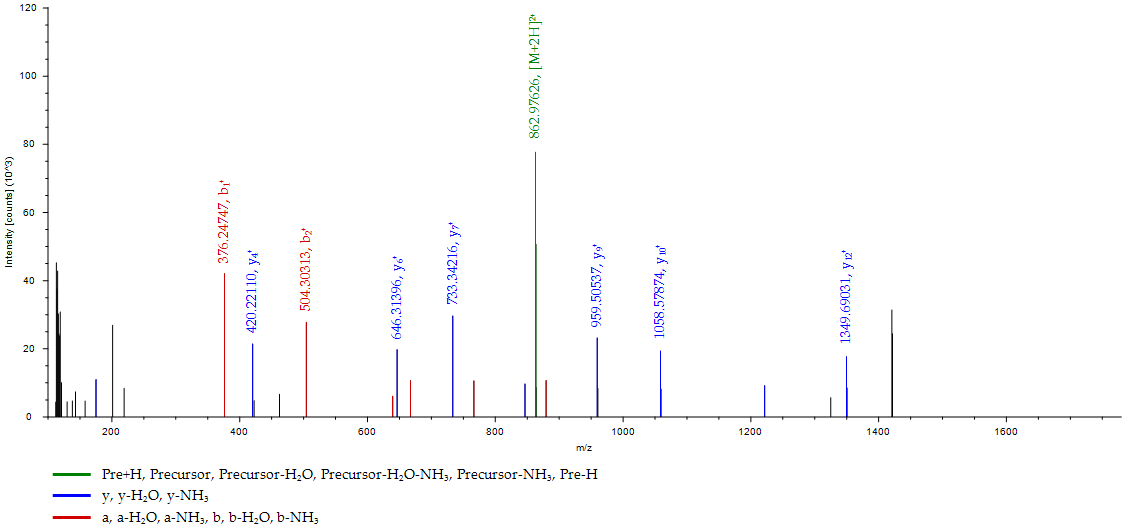
**

**Fig S4C. Annotated spectrum for 11122875 - glycosylphosphatidylinositol phospholipase D [Homo sapiens]**

**
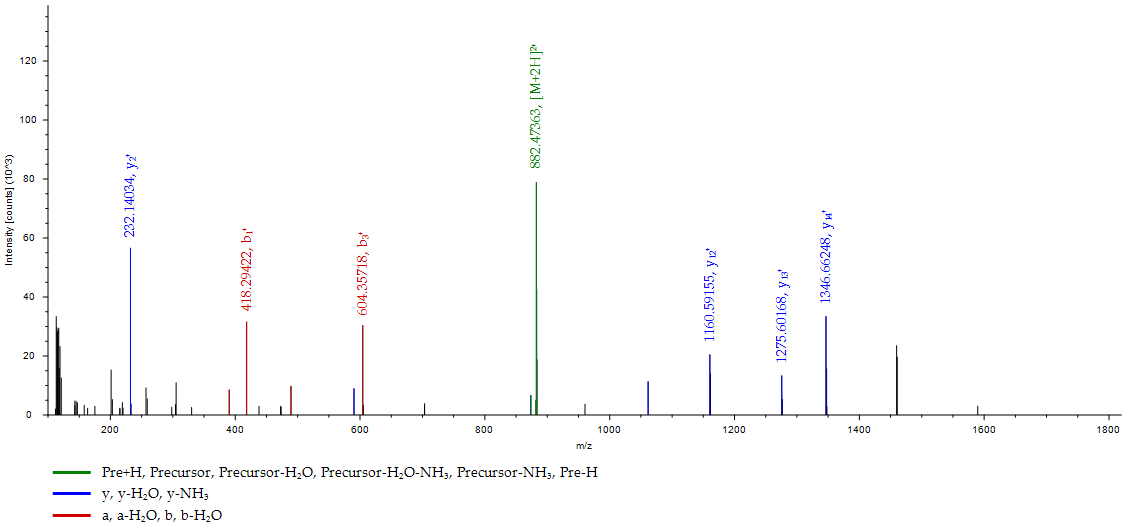
**

**Fig S4D. Annotated spectrum for 11122875 - glycosylphosphatidylinositol phospholipase D [Homo sapiens]**

**
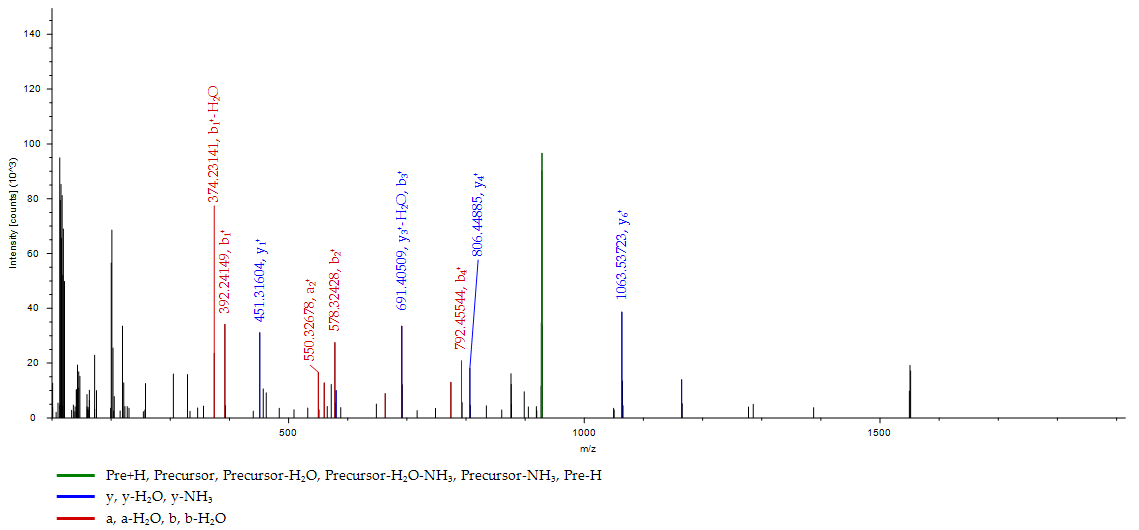
**

**Fig S5. Annotated spectrum for 47124510 - APCS protein [Homo sapiens]**

**
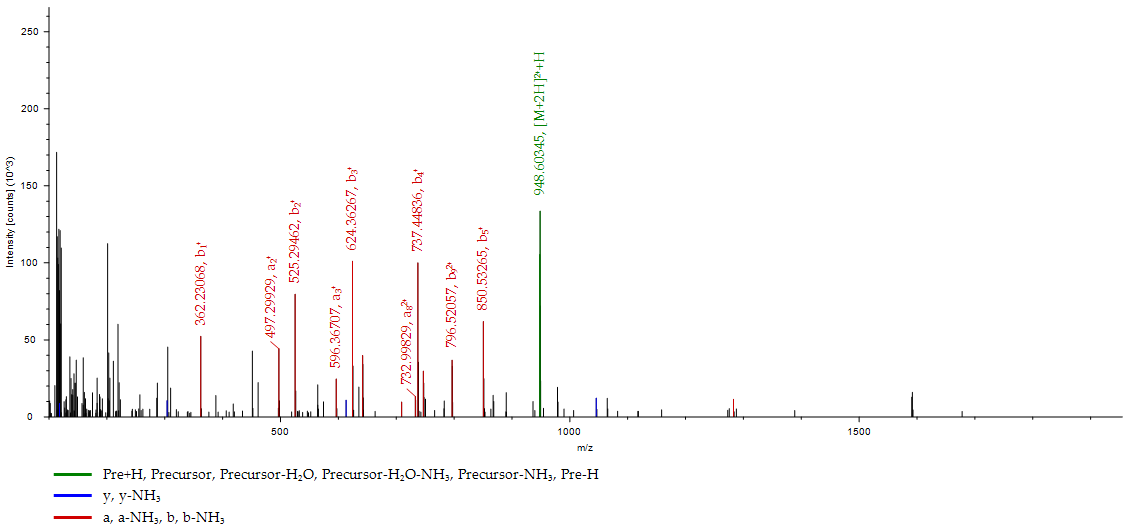
**

**Fig S6A. Annotated spectrum for 13937839 - SAA1 protein [Homo sapiens]**

**
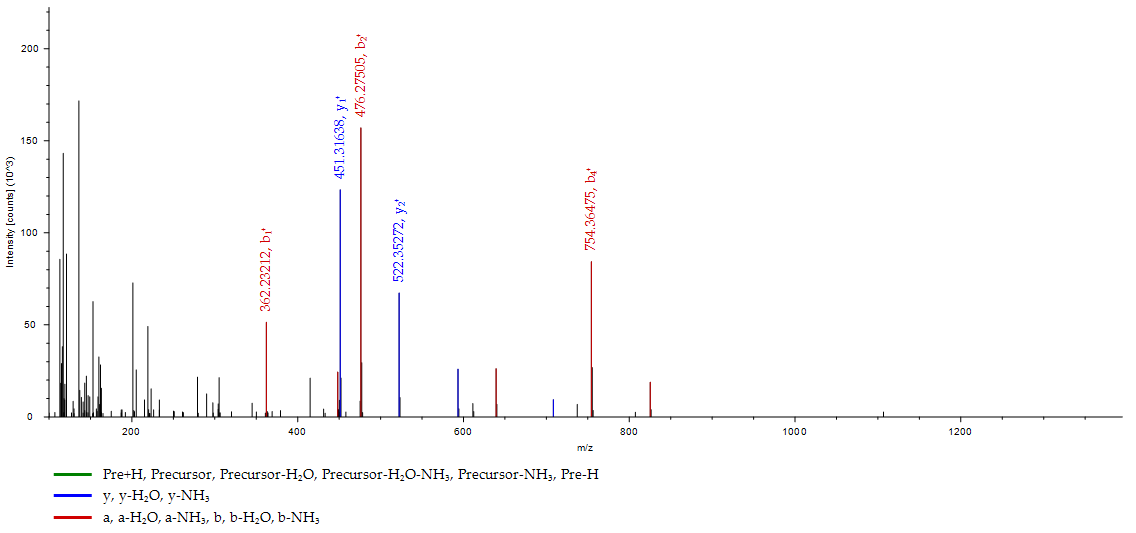
**

**Fig S6B. Annotated spectrum for 13937839 - SAA1 protein [Homo sapiens]**

**
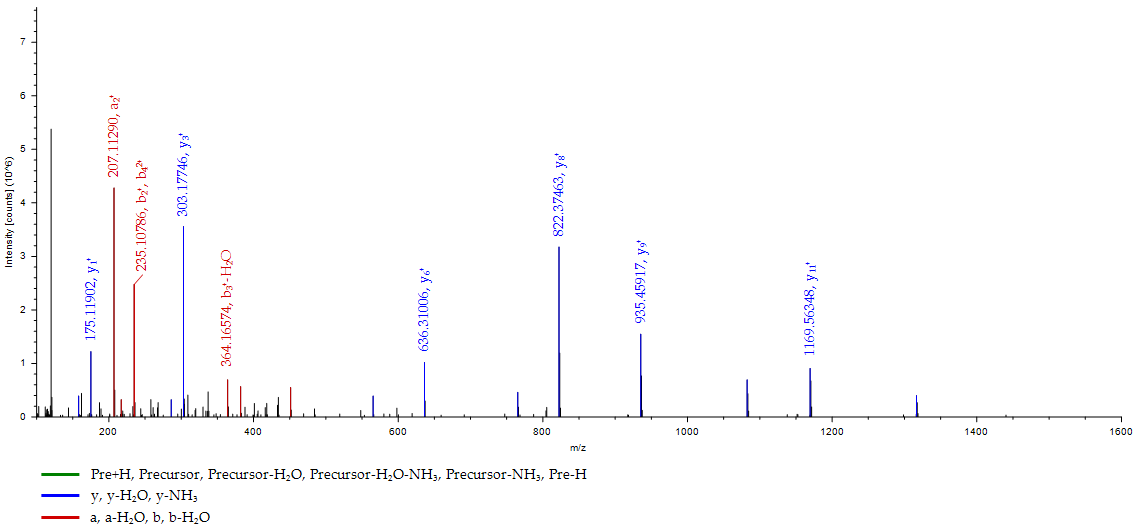
**

**Fig S6C. Annotated spectrum for 13937839 - SAA1 protein [Homo sapiens]**

**
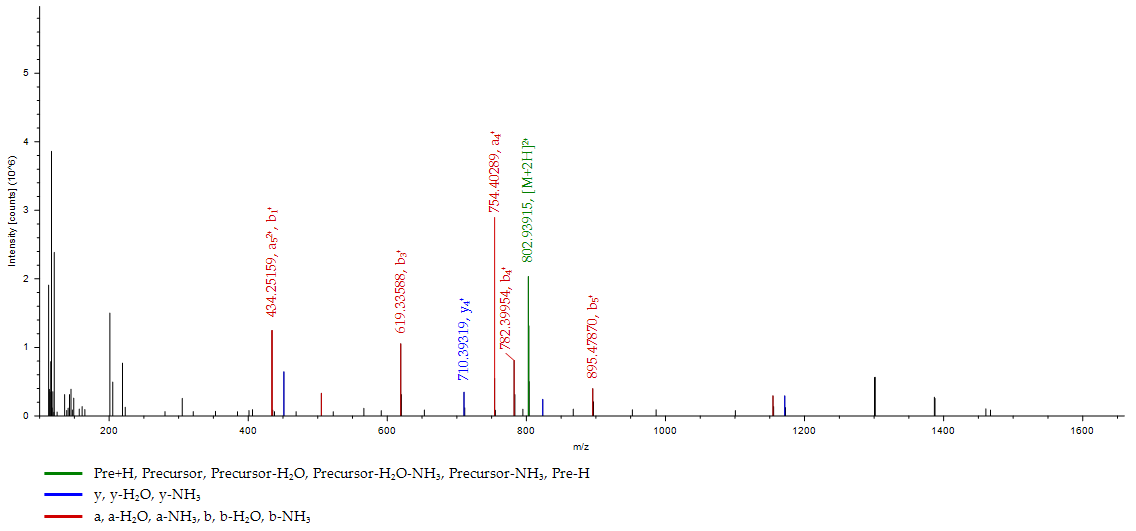
**

**Fig S6D. Annotated spectrum for 13937839 - SAA1 protein [Homo sapiens]**

**
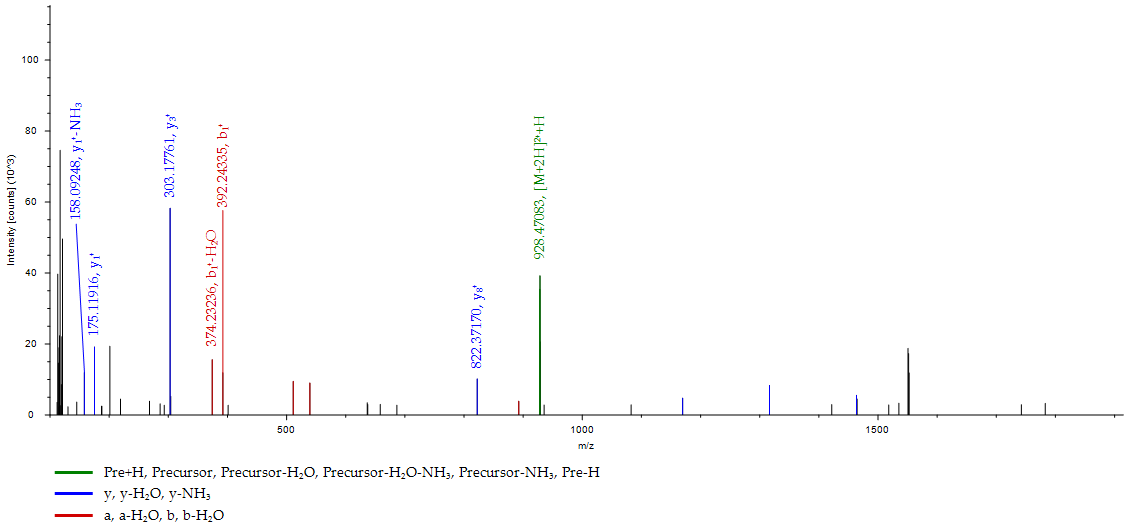
**

**Fig S6E. Annotated spectrum for 13937839 - SAA1 protein [Homo sapiens]**

**
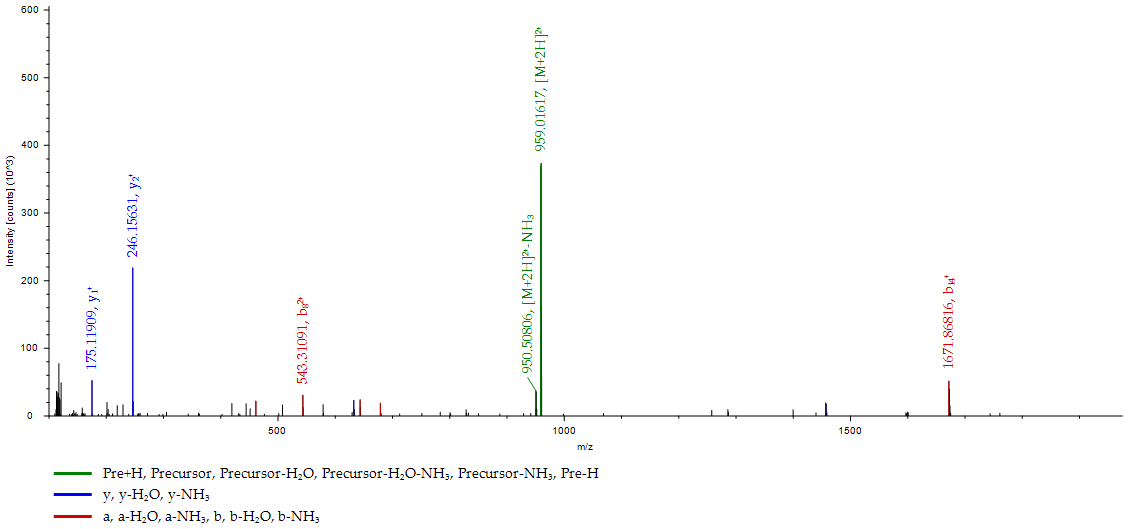
**

**Fig S6F. Annotated spectrum for 13937839 - SAA1 protein [Homo sapiens]**

**
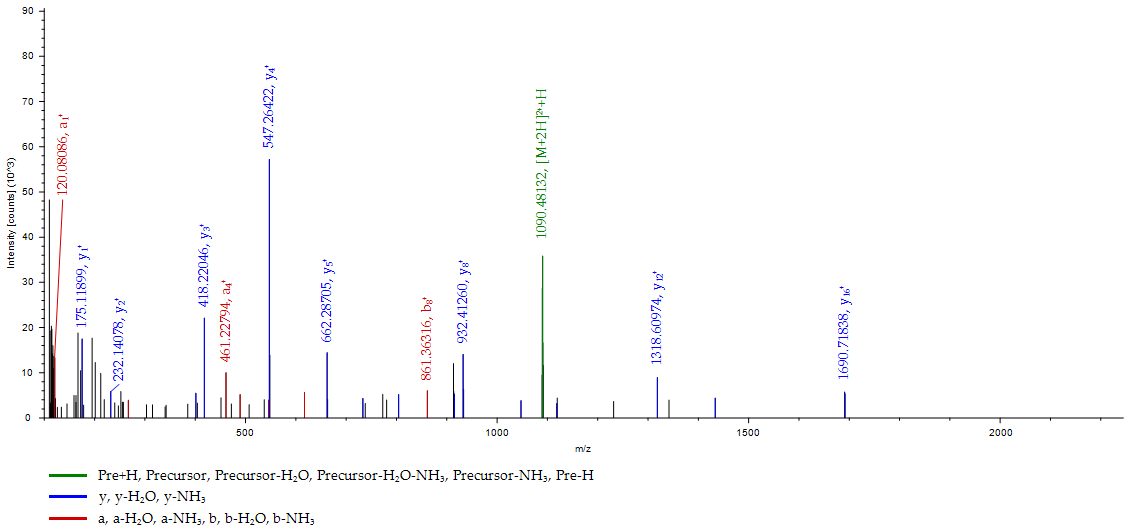
**

**Fig S6G. Annotated spectrum for 13937839 - SAA1 protein [Homo sapiens]**

**
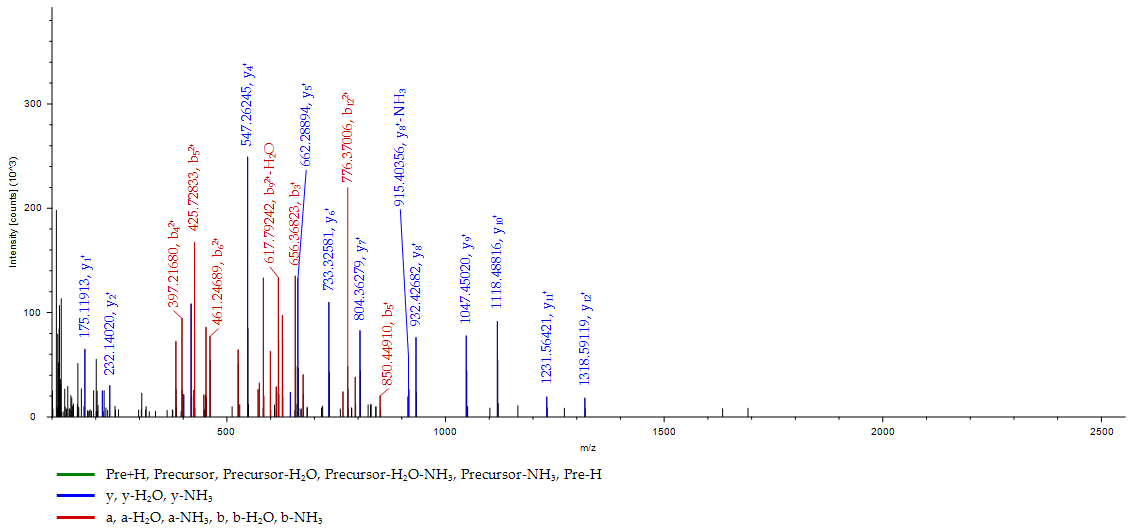
**

**Fig S7A. Annotated spectrum for 1064908 - complement Factor H-related Protein 2 [Homo sapiens]**

**
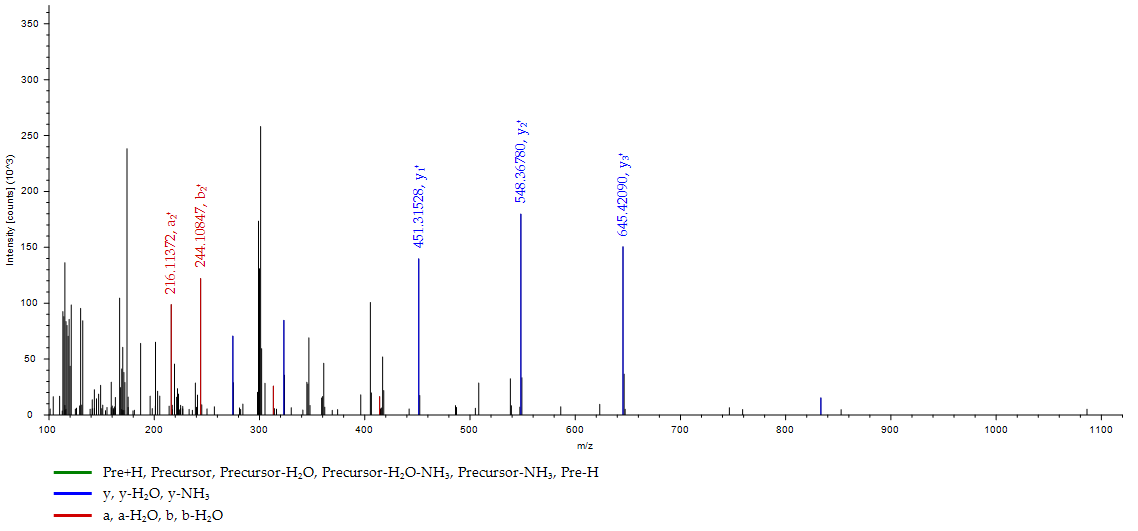
**

**Fig S7B. Annotated spectrum for 1064908 - complement Factor H-related Protein 2 [Homo sapiens]**

**
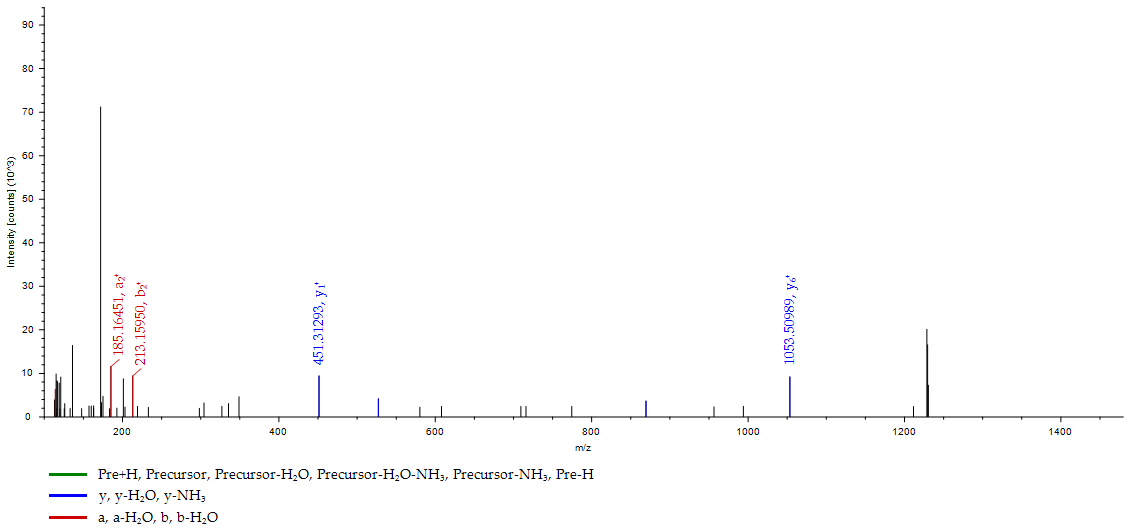
**

**Fig S7C. Annotated spectrum for 1064908 - complement Factor H-related Protein 2 [Homo sapiens]**

**
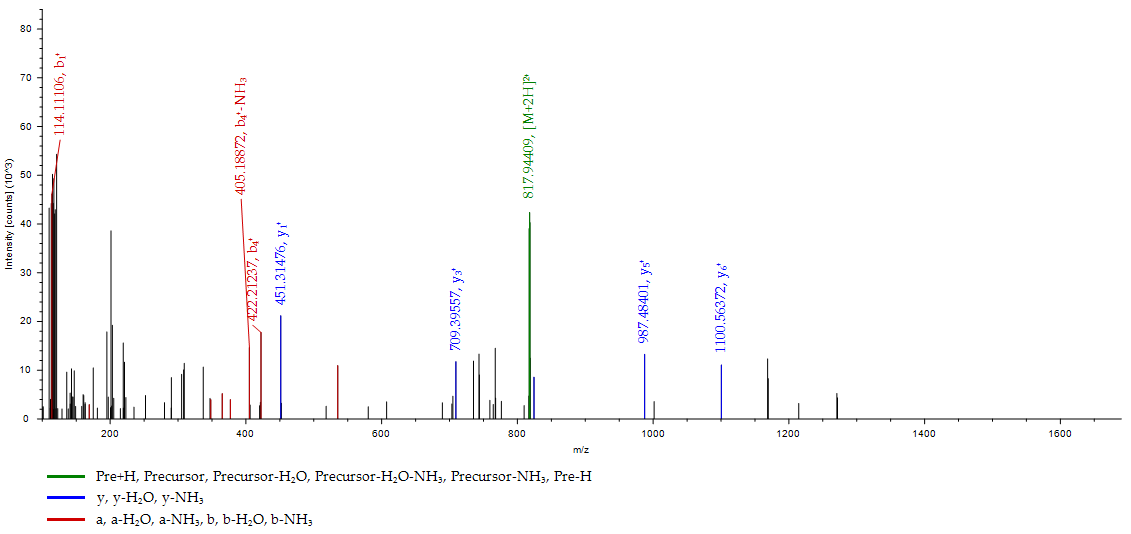
**

**Fig S7D. Annotated spectrum for 1064908 - complement Factor H-related Protein 2 [Homo sapiens]**

**
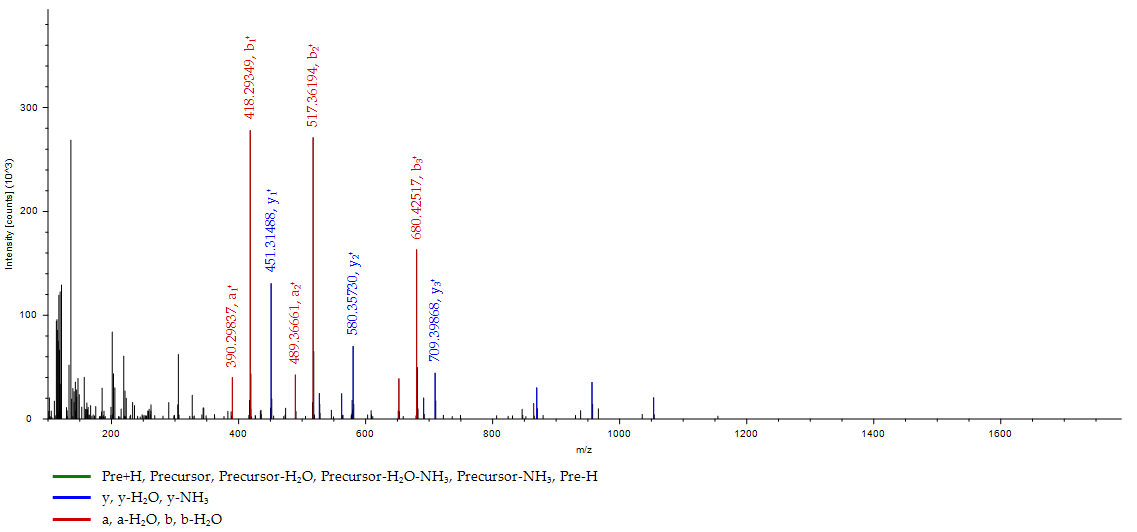
**

**Fig S7E. Annotated spectrum for 1064908 - complement Factor H-related Protein 2 [Homo sapiens]**

**
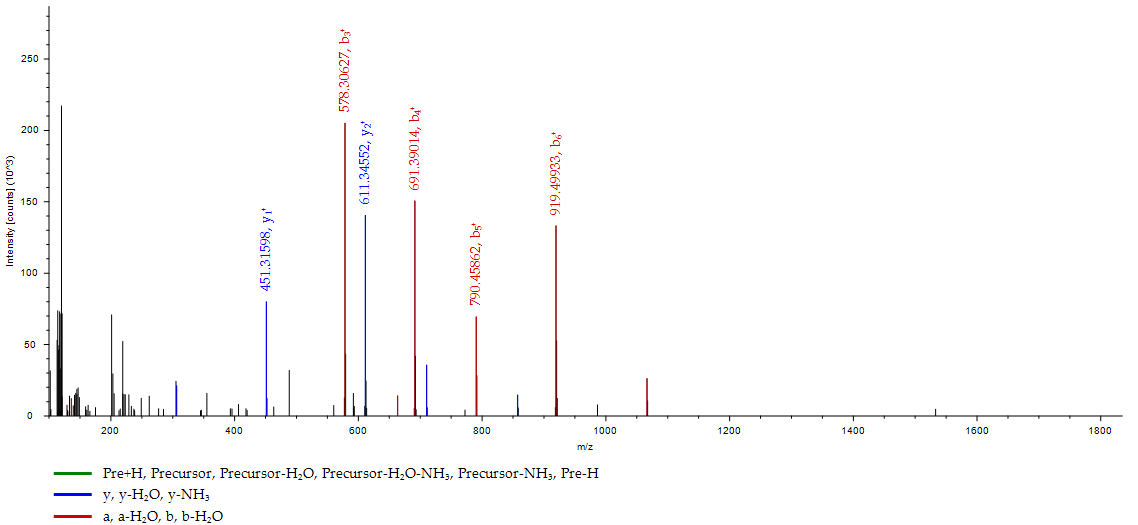
**

**Fig S7F. Annotated spectrum for 1064908 - complement Factor H-related Protein 2 [Homo sapiens]**

**
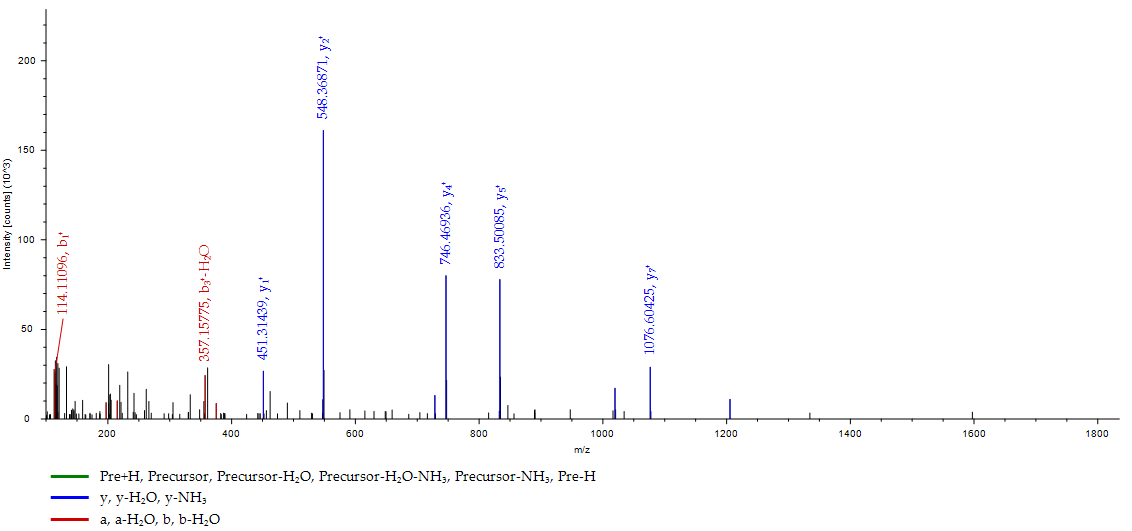
**

**Fig S7G. Annotated spectrum for 1064908 - complement Factor H-related Protein 2 [Homo sapiens]**

**
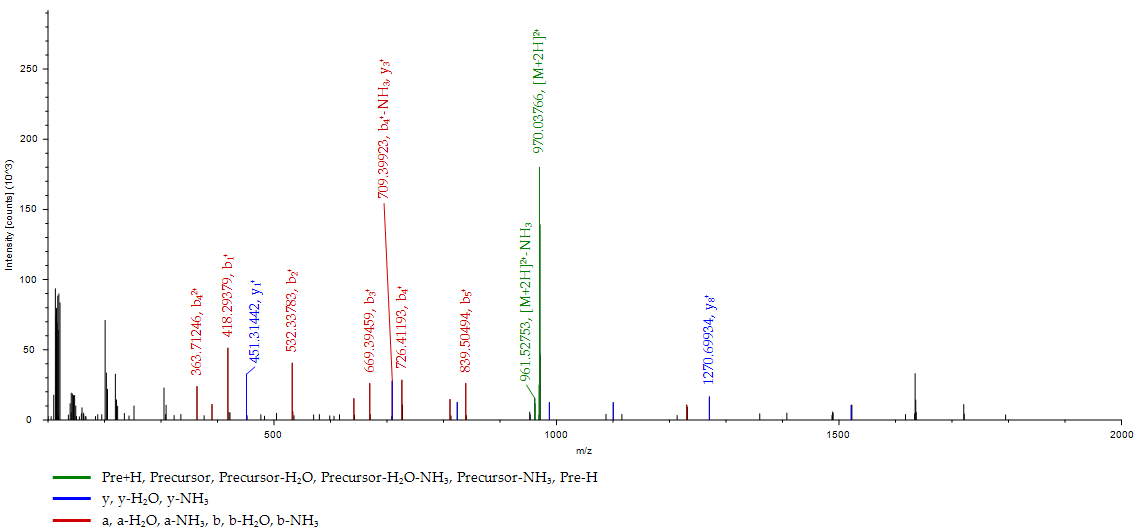
**

**Fig S7H. Annotated spectrum for 1064908 - complement Factor H-related Protein 2 [Homo sapiens]**

**
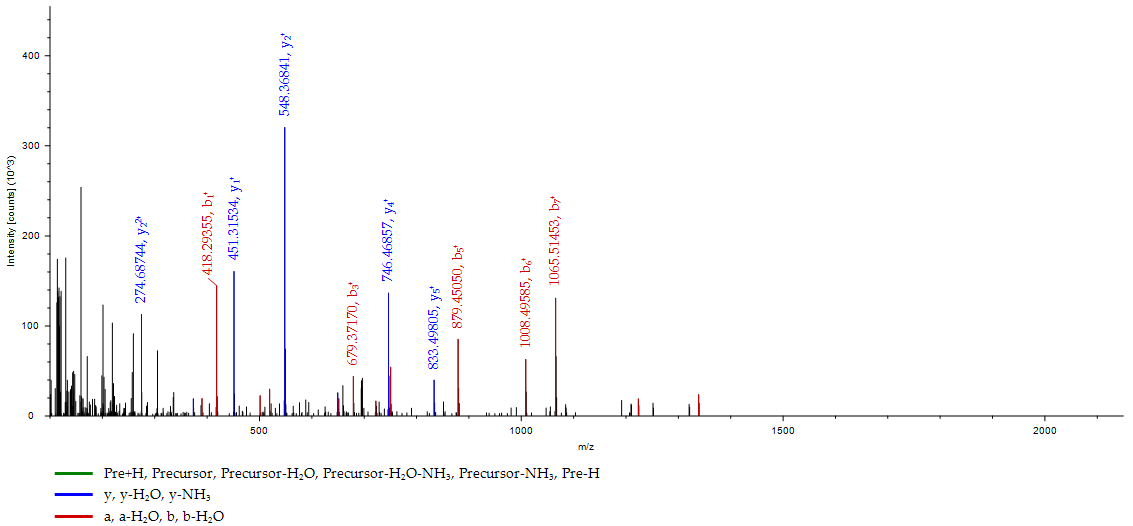
**

**Fig S7I. Annotated spectrum for 1064908 - complement Factor H-related Protein 2 [Homo sapiens]**

**
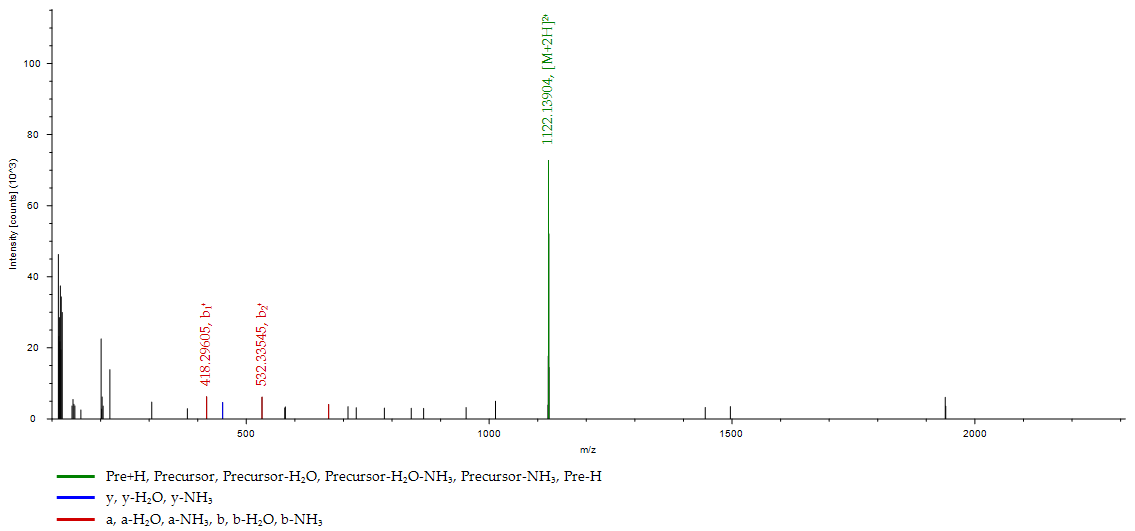
**

**Fig S7K. Annotated spectrum for 1064908 - complement Factor H-related Protein 2 [Homo sapiens]**

**
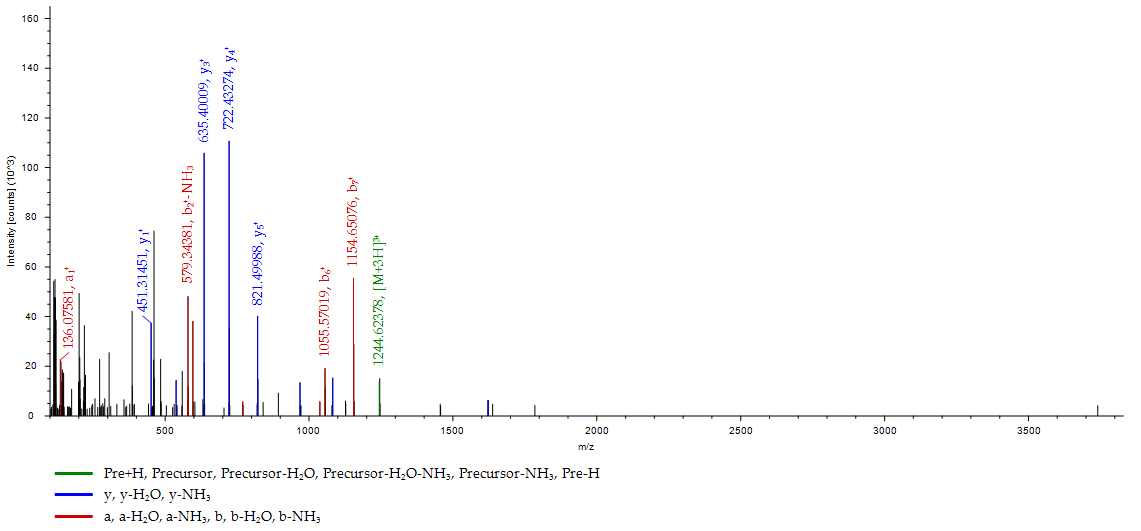
**

**Fig S7L. Annotated spectrum for 1064908 - complement Factor H-related Protein 2 [Homo sapiens]**

**
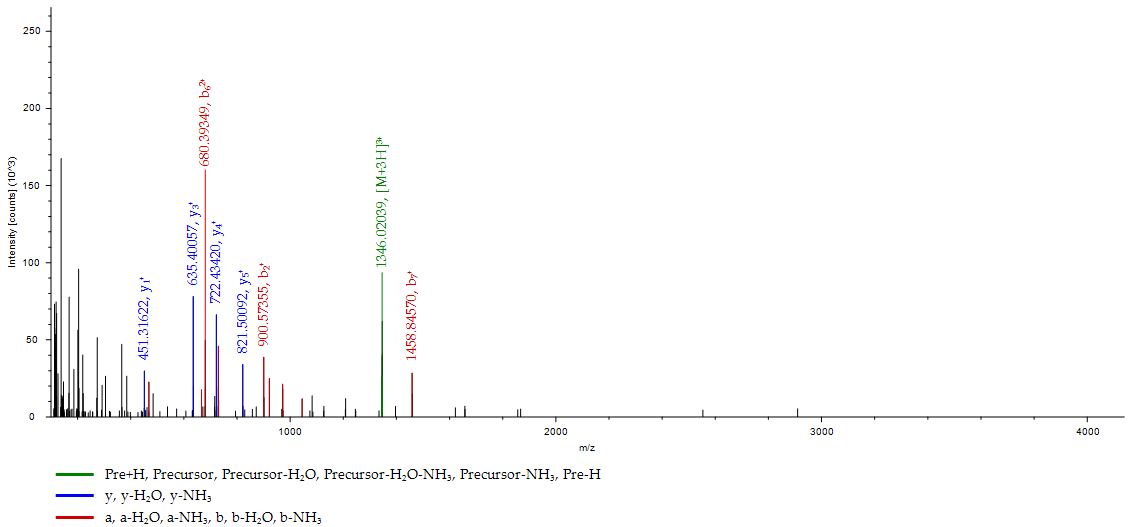
**

**Fig S8. Annotated spectrum for 1769552 - von Willebrand factor [Homo sapiens]**

**
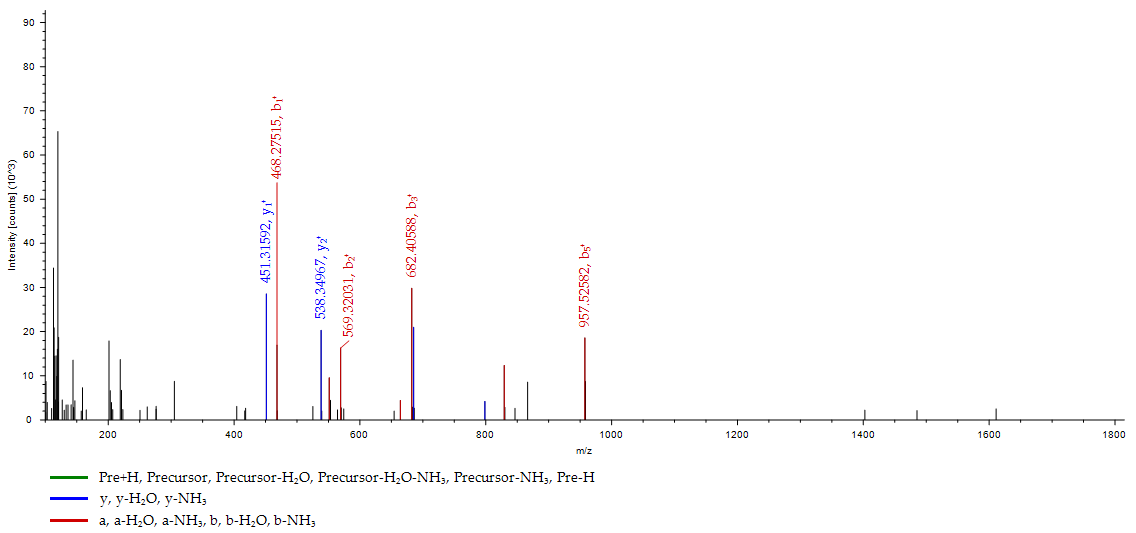
**

**Fig S9A. Annotated spectrum for 20377087 - intestinal lactoferrin receptor [Homo sapiens]**

**
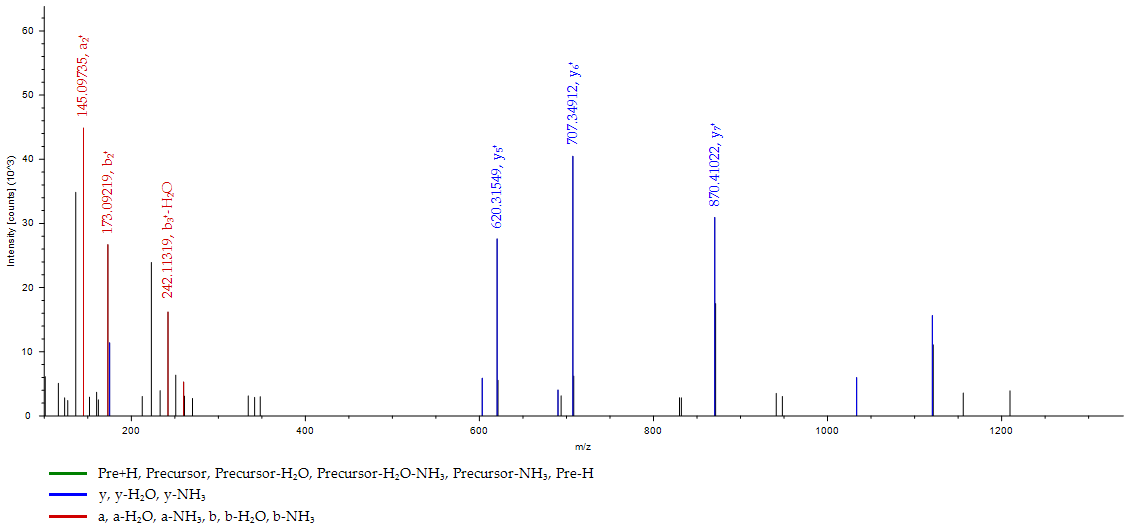
**

**Fig S9B. Annotated spectrum for 20377087 - intestinal lactoferrin receptor [Homo sapiens]**

**
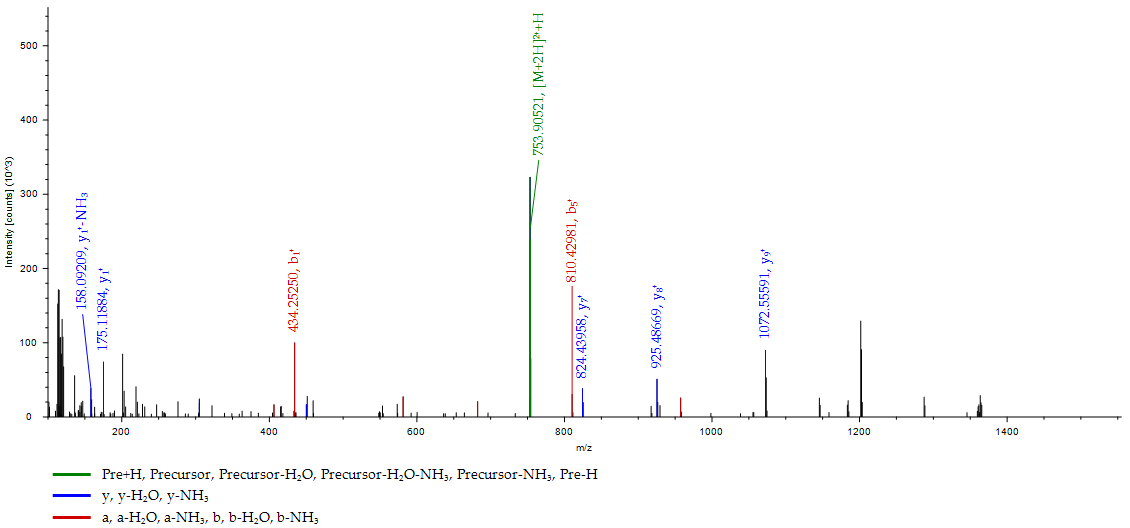
**

**Fig S9C. Annotated spectrum for 20377087 - intestinal lactoferrin receptor [Homo sapiens]**

**
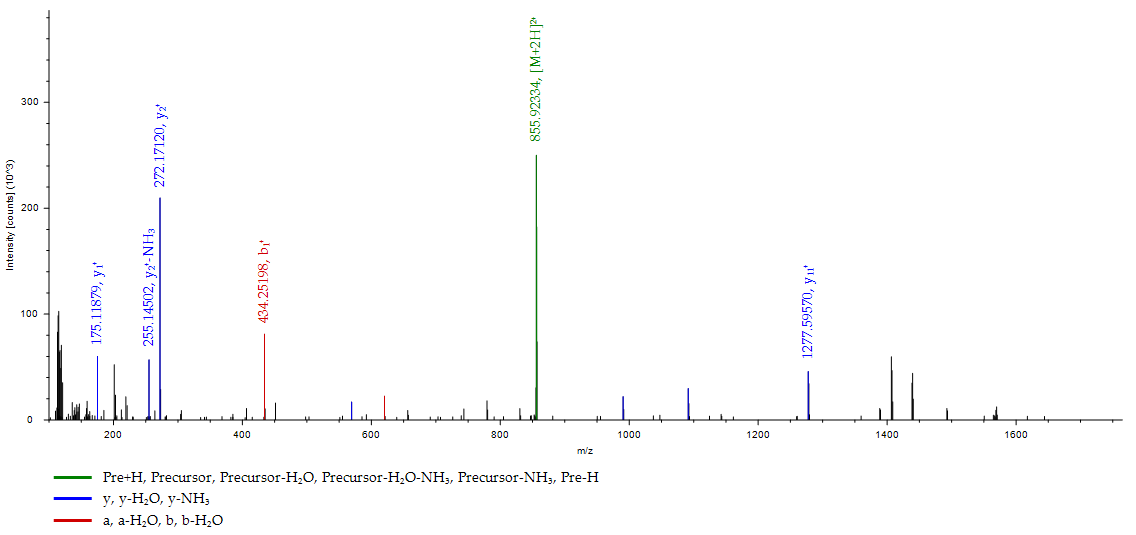
**

**Fig S9D. Annotated spectrum for 20377087 - intestinal lactoferrin receptor [Homo sapiens]**

**
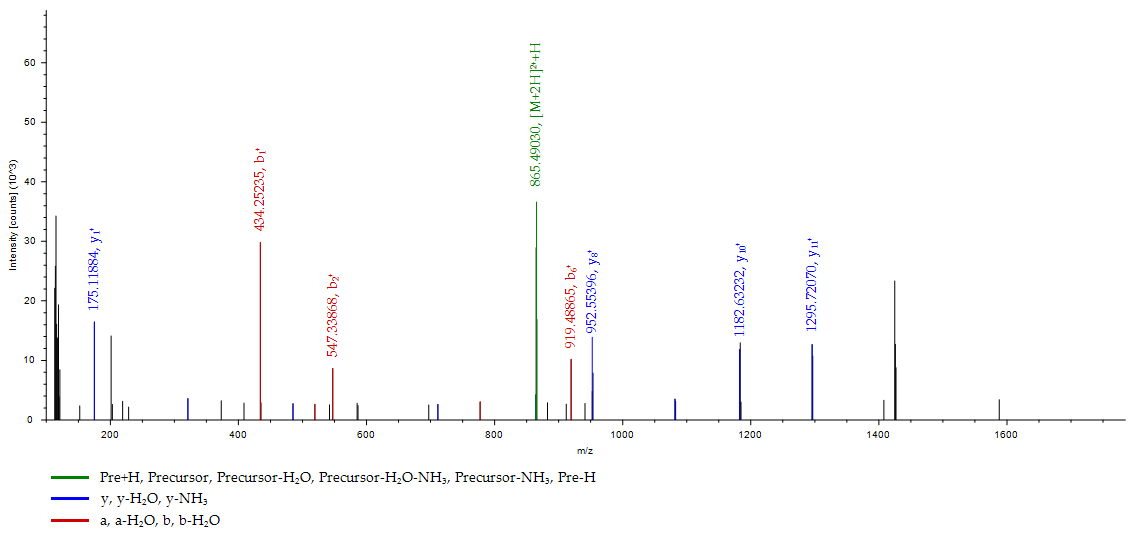
**

**Fig S9E. Annotated spectrum for 20377087 - intestinal lactoferrin receptor [Homo sapiens]**

**
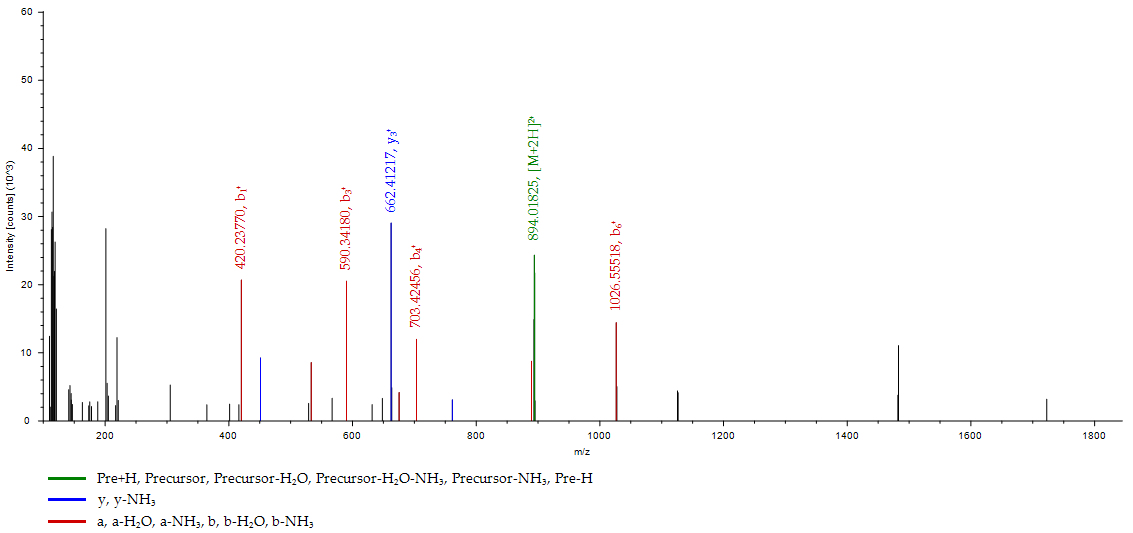
**

**Fig S9F. Annotated spectrum for 20377087 - intestinal lactoferrin receptor [Homo sapiens]**

**
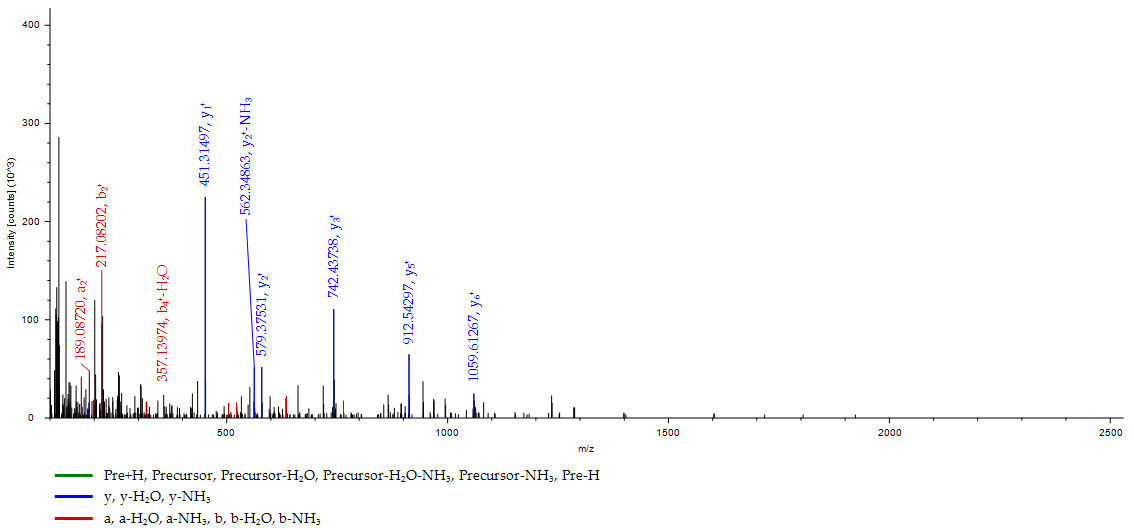
**

**Fig S9G. Annotated spectrum for 20377087 - intestinal lactoferrin receptor [Homo sapiens]**

**
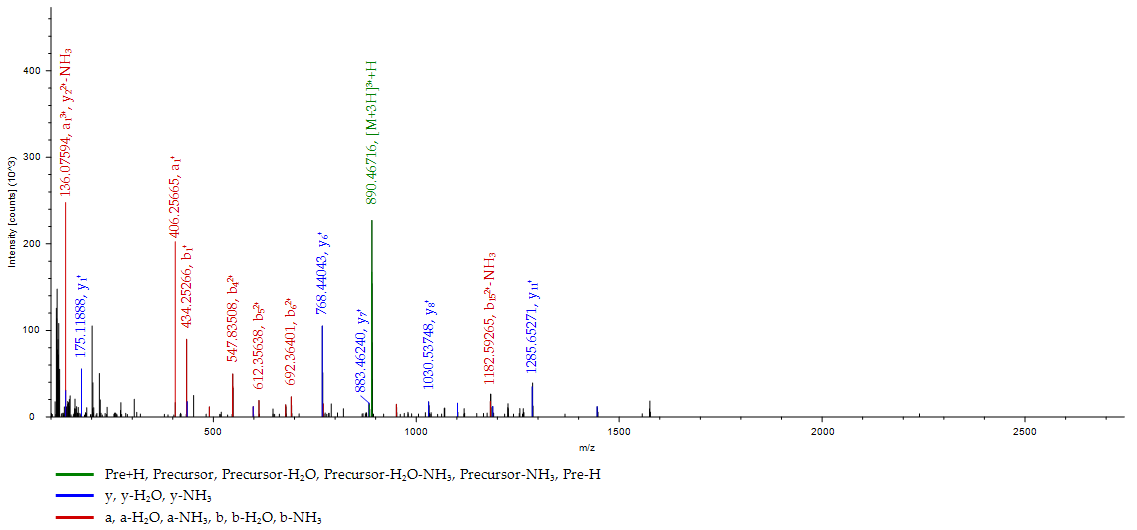
**

**Fig S10A. Annotated spectrum for 74355107 - BRF1 protein [Homo sapiens]**

**
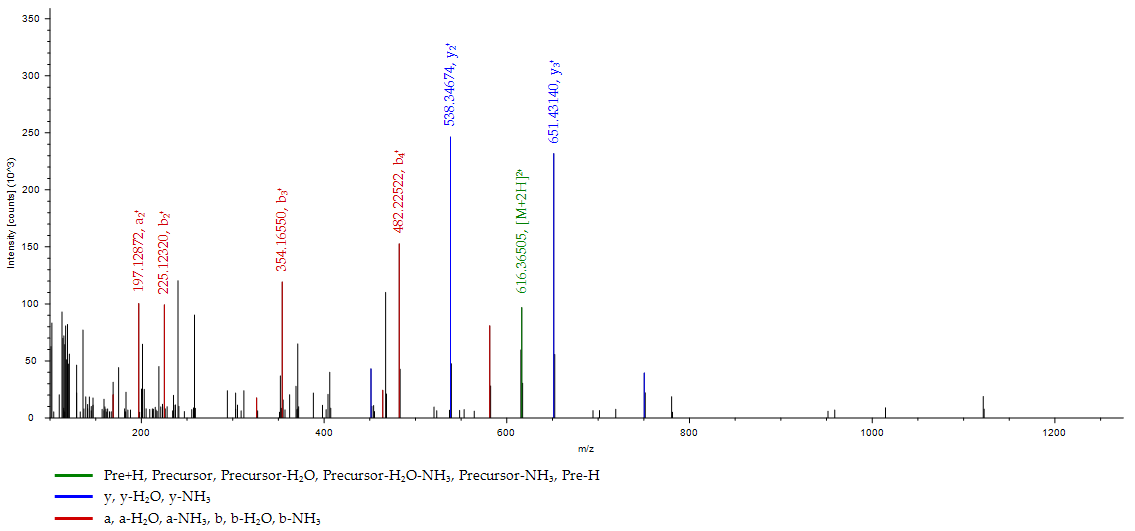
**

**Fig S10B. Annotated spectrum for 74355107 - BRF1 protein [Homo sapiens]**

**
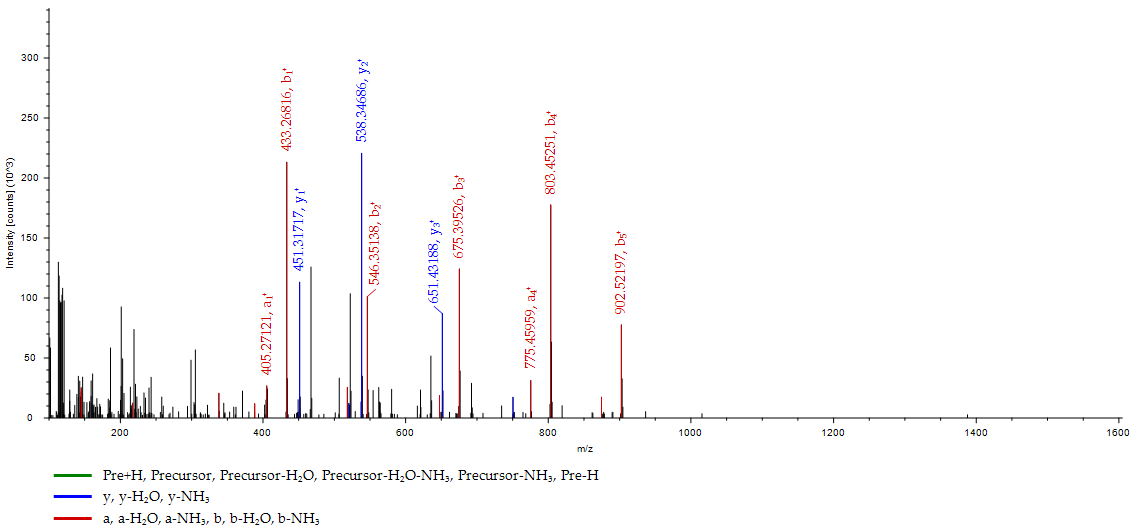
**

**Figure S11. ECM-receptor interaction pathway involved in the CHD-PAH.**

**
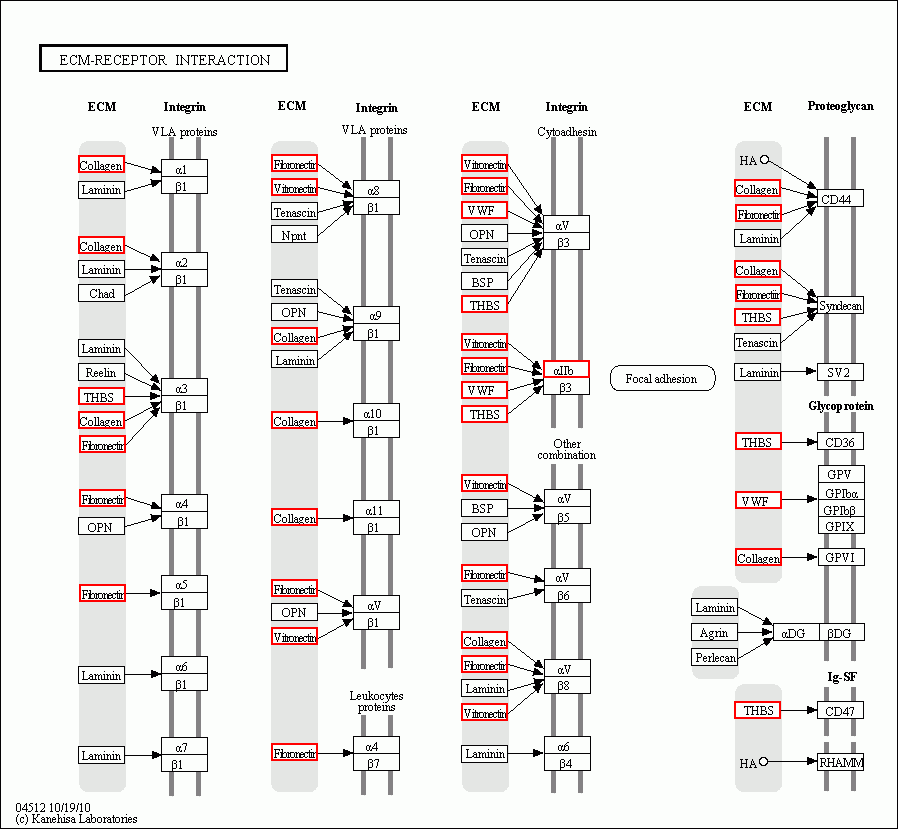
**
