# Supplementary material for: Plasma Proteomic Study in Pulmonary Arterial Hypertension Associated with Congenital Heart Diseases
Source: Sci Rep. 2016 Nov 25;6:36541. doi: 10.1038/srep36541 (PMC5122864; doi:10.1038/srep36541)
Supplement: Supplementary File S1 [file srep36541-s2.doc]

**Plasma Proteomic Study in Pulmonary Arterial Hypertension Associated with Congenital Heart Diseases**

**Xi Zhang,** *PhD***, Hai-Tao Hou,** *MD,* **Jun Wang,** *MD*, **Xiao-Cheng Liu,** *MD*, **Qin Yang,** *PhD*, **and Guo-Wei He*,** *PhD, DSc*

**Abbreviations**

| **TI** | **tricuspid incompetence** |
| --- | --- |
| **DORV** | **double outlet right ventricular** |
| **DCRV** | **double chambered right ventricular** |
| **DS** | **Down syndrome** |
| **PAPVC** | **partial anomalous pulmonary venous connection** |
| **CAVCD** | **complete atrioventricular canal defect** |
| **CoA** | **aortic coarctation** |
| **IAA** | **interruption of aortic arch** |
| **ECD** | **endocardial cushion defect** |

VSD-PAH

| **iTRAQ：** | Patient 1 | VSD，PH | **ELISA：** | Patient 1 | VSD，PH，PFO |
| --- | --- | --- | --- | --- | --- |
|  | Patient 2 | VSD，PH |  | Patient 2 | VSD，PH |
|  | Patient 3 | VSD，PH |  | Patient 3 | VSD，PH |
|  | Patient 4 | VSD，PH |  | Patient 4 | VSD，PH, PFO |
|  | Patient 5 | VSD，PH |  | Patient 5 | VSD，PH, PFO |
|  | Patient 6 | VSD，PH |  | Patient 6 | VSD，PH |
|  | Patient 7 | VSD，PH, |  | Patient 7 | VSD，PH |
|  | Patient 8 | VSD，PH |  | Patient 8 | VSD，PH, TI |
|  | Patient 9 | VSD，PH |  | Patient 9 | VSD，PH |
|  | Patient 10 | VSD，PH, PFO |  | Patient 10 | VSD，PH, PFO |
|  | Patient 11 | VSD，PH, PFO |  | Patient 11 | VSD，PH, TI |
|  | Patient 12 | VSD，PH |  | Patient 12 | VSD，PH |
|  | Patient 13 | VSD，PH, TI |  | Patient 13 | VSD，PH |
|  | Patient 14 | VSD，PH |  | Patient 14 | VSD，PH，PLSVC |
|  | Patient 15 | VSD，PH |  | Patient 15 | VSD，PH |
|  | Patient 16 | VSD，PH, PFO |  | Patient 16 | VSD，PH |
|  | Patient 17 | VSD，PH, PFO |  | Patient 17 | VSD，PH |
|  | Patient 18 | VSD，PH, PFO |  | Patient 18 | VSD，PH, PFO |
|  | Patient 19 | VSD，PH, MI |  | Patient 19 | VSD，PH |
|  | Patient 20 | VSD，PH |  | Patient 20 | VSD，PH, PFO |
|  |  |  |  | Patient 21 | VSD，PH |
|  |  |  |  | Patient 22 | VSD，PH, PFO |
|  |  |  |  | Patient 23 | VSD，PH |
|  |  |  |  | Patient 24 | VSD，PH |
|  |  |  |  | Patient 25 | VSD，PH, PLSVC |
|  |  |  |  | Patient 26 | VSD，PH |
|  |  |  |  | Patient 27 | VSD，PH |
|  |  |  |  | Patient 28 | VSD，PH |
|  |  |  |  | Patient 29 | VSD，PH |
|  |  |  |  | Patient 30 | VSD，PH, PFO |
|  |  |  |  | Patient 31 | VSD，PH |
|  |  |  |  | Patient 32 | VSD，PH, PFO |
|  |  |  |  | Patient 33 | VSD，PH, PFO |
|  |  |  |  | Patient 34 | VSD，PH |
|  |  |  |  | Patient 35 | VSD，PH |
|  |  |  |  | Patient 36 | VSD，PH, TI |
|  |  |  |  | Patient 37 | VSD，PH |
|  |  |  |  | Patient 38 | VSD，PH |
|  |  |  |  | Patient 39 | VSD，PH |
|  |  |  |  | Patient 40 | VSD，PH |

VSD

| **iTRAQ：** | Patient 1 | VSD | **ELISA：** | Patient 1 | VSD |
| --- | --- | --- | --- | --- | --- |
|  | Patient 2 | VSD |  | Patient 2 | VSD |
|  | Patient 3 | VSD，PFO |  | Patient 3 | VSD |
|  | Patient 4 | VSD |  | Patient 4 | VSD |
|  | Patient 5 | VSD |  | Patient 5 | VSD，PFO |
|  | Patient 6 | VSD |  | Patient 6 | VSD |
|  | Patient 7 | VSD |  | Patient 7 | VSD |
|  | Patient 8 | VSD |  | Patient 8 | VSD |
|  | Patient 9 | VSD |  | Patient 9 | VSD，PFO |
|  | Patient 10 | VSD |  | Patient 10 | VSD |
|  | Patient 11 | VSD |  | Patient 11 | VSD，TI |
|  | Patient 12 | VSD，PFO |  | Patient 12 | VSD |
|  | Patient 13 | VSD |  | Patient 13 | VSD，PFO |
|  | Patient 14 | VSD，PFO |  | Patient 14 | VSD，PFO |
|  | Patient 15 | VSD，PFO |  | Patient 15 | VSD，TI |
|  | Patient 16 | VSD，PFO |  | Patient 16 | VSD |
|  | Patient 17 | VSD |  | Patient 17 | VSD |
|  | Patient 18 | VSD |  | Patient 18 | VSD |
|  | Patient 19 | VSD |  | Patient 19 | VSD |
|  | Patient 20 | VSD |  | Patient 20 | VSD |
|  |  |  |  | Patient 21 | VSD |

ASD-PAH

| **iTRAQ：** | Patient 1 | ASD，PH | **ELISA：** | Patient 1 | ASD，PH |
| --- | --- | --- | --- | --- | --- |
|  | Patient 2 | ASD，PH |  | Patient 2 | ASD，PH |
|  | Patient 3 | ASD，PH |  | Patient 3 | ASD，PH, TI |
|  | Patient 4 | ASD，PH, TI |  | Patient 4 | ASD，PH, TI |
|  | Patient 5 | ASD，PH |  | Patient 5 | ASD，PH |
|  | Patient 6 | ASD，PH |  | Patient 6 | ASD，PH, TI |
|  | Patient 7 | ASD，PH |  | Patient 7 | ASD，PH, TI |
|  | Patient 8 | ASD，PH |  | Patient 8 | ASD，PH，PAPVC |
|  | Patient 9 | ASD，PH |  | Patient 9 | ASD，PH, TI |
|  | Patient 10 | ASD，PH |  | Patient 10 | ASD，PH |
|  | Patient 11 | ASD，PH |  | Patient 11 | ASD，PH |
|  | Patient 12 | ASD，PH |  | Patient 12 | ASD，PH |
|  | Patient 13 | ASD，PH |  | Patient 13 | ASD，PH |
|  | Patient 14 | ASD，PH |  | Patient 14 | ASD，PH |
|  | Patient 15 | ASD，PH |  | Patient 15 | ASD，PH |
|  | Patient 16 | ASD，PH |  | Patient 16 | ASD，PH, TI |
|  | Patient 17 | ASD，PH |  | Patient 17 | ASD，PH |
|  | Patient 18 | ASD，PH |  | Patient 18 | ASD，PH, TI |
|  | Patient 19 | ASD，PH |  | Patient 19 | ASD，PH |
|  | Patient 20 | ASD，PH |  | Patient 20 | ASD，PH, TI |
|  |  |  |  | Patient 21 | ASD，PH, TI |

ASD

| **iTRAQ：** | Patient 1 | ASD | **ELISA：** | Patient 1 | ASD |
| --- | --- | --- | --- | --- | --- |
|  | Patient 2 | ASD |  | Patient 2 | ASD |
|  | Patient 3 | ASD |  | Patient 3 | ASD |
|  | Patient 4 | ASD |  | Patient 4 | ASD，PFO |
|  | Patient 5 | ASD |  | Patient 5 | ASD，PFO |
|  | Patient 6 | ASD |  | Patient 6 | ASD |
|  | Patient 7 | ASD |  | Patient 7 | ASD |
|  | Patient 8 | ASD |  | Patient 8 | ASD |
|  | Patient 9 | ASD |  | Patient 9 | ASD |
|  | Patient 10 | ASD |  | Patient 10 | ASD |
|  | Patient 11 | ASD |  | Patient 11 | ASD |
|  | Patient 12 | ASD |  | Patient 12 | ASD |
|  | Patient 13 | ASD |  | Patient 13 | ASD |
|  | Patient 14 | ASD |  | Patient 14 | ASD |
|  | Patient 15 | ASD |  | Patient 15 | ASD |
|  | Patient 16 | ASD |  | Patient 16 | ASD |
|  | Patient 17 | ASD |  | Patient 17 | ASD |
|  | Patient 18 | ASD |  | Patient 18 | ASD，TI |
|  | Patient 19 | ASD |  | Patient 19 | ASD |
|  | Patient 20 | ASD |  | Patient 20 | ASD，TI |
|  |  |  |  | Patient 21 | ASD |

Mix-PAH

| **iTRAQ：** | Patient 1 | VSD，ASD，MI，PH | **ELISA：** | Patient 1 | CAVCD，ASD，PH |
| --- | --- | --- | --- | --- | --- |
|  | Patient 2 | VSD，RVOT，MI，PDA，PH |  | Patient 2 | VSD，ASD，PLSVC，PH |
|  | Patient 3 | VSD，ASD，PH |  | Patient 3 | VSD，PDA，DROV，PH |
|  | Patient 4 | ASD，PDA，PH |  | Patient 4 | ASD，PDA，PH |
|  | Patient 5 | VSD，PDA，ASD，PH |  | Patient 5 | ASD，PDA，PH，DROV |
|  | Patient 6 | VSD，ASD，PH |  | Patient 6 | VSD，ASD，PH |
|  | Patient 7 | VSD，PDA，PH |  | Patient 7 | VSD，PDA，MI，TI，PI，PH |
|  | Patient 8 | VSD，PFO，ASD，PH |  | Patient 8 | VSD，ASD，TI，PH |
|  | Patient 9 | ASD，PDA，PH |  | Patient 9 | VSD，PDA，ASD |
|  | Patient 10 | VSD，PDA，TI，PH |  | Patient 10 | VSD，ASD，PH |
|  | Patient 11 | VSD，ASD，RVOT，PH |  | Patient 11 | PDA，ASD，PS，PH |
|  | Patient 12 | VSD，ASD，TI，PDA，PH |  | Patient 12 | ASD，VSD，PH |
|  | Patient 13 | VSD，ASD，RVOTS，PH |  | Patient 13 | VSD，ASD，PH |
|  | Patient 14 | VSD，PDA，TI，PH |  | Patient 14 | VSD，ASD，PH |
|  | Patient 15 | IAA，VSD，PDA，TI，PH |  | Patient 15 | ASD，PDA，PH |
|  | Patient 16 | VSD、ASD、PH |  | Patient 16 | VSD，ASD，PH |
|  | Patient 17 | VSD、ASD、PH |  | Patient 17 | VSD，PDA，CoA，RVOT，PFO，PH，MI |
|  | Patient 18 | VSD、ASD、PH |  | Patient 18 | VSD，PDA，PH |
|  | Patient 19 | ASD、PDA、PH |  | Patient 19 | VSD，PDA，PH，TI |
|  | Patient 20 | VSD、PDA、PH |  | Patient 20 | ASD，PDA，TI，PH |
|  |  |  |  | Patient 21 | IAA，VSD，PDA，PH，RVOT |

Mix

| **iTRAQ：** | Patient 1 | VSD，PDA，ASD | **ELISA：** | Patient 1 | VSD，ASD |
| --- | --- | --- | --- | --- | --- |
|  | Patient 2 | VSD，PDA |  | Patient 2 | ASD，PDA |
|  | Patient 3 | VSD，ASD |  | Patient 3 | VSD，PDA，PFO |
|  | Patient 4 | ASD，PDA |  | Patient 4 | VSD，ASD |
|  | Patient 5 | VSD，PDA |  | Patient 5 | VSD，PDA |
|  | Patient 6 | ASD，PDA |  | Patient 6 | ASD，VSD |
|  | Patient 7 | VSD，PDA |  | Patient 7 | ASD，PDA |
|  | Patient 8 | ASD，VSD |  | Patient 8 | ASD，VSD |
|  | Patient 9 | ASD，TI |  | Patient 9 | VSD，PDA，TI |
|  | Patient 10 | VSD，ASD |  | Patient 10 | ASD，PDA |
|  | Patient 11 | VSD，PDA |  | Patient 11 | VSD，ASD，PLSVC |
|  | Patient 12 | ASD，PDA |  | Patient 12 | PDA，ASD |
|  | Patient 13 | COA，PDA，ASD |  | Patient 13 | ASD，PDA |
|  | Patient 14 | VSD，ASD |  | Patient 14 | VSD，ASD |
|  | Patient 15 | VSD，ASD |  | Patient 15 | VSD，PDA |
|  | Patient 16 | VSD，ASD |  | Patient 16 | ASD，VSD |
|  | Patient 17 | VSD，ASD，PDA |  | Patient 17 | ASD，PDA |
|  | Patient 18 | ASD，VSD |  | Patient 18 | VSD，PDA，DCRV |
|  | Patient 19 | VSD，ASD |  | Patient 19 | VSD，ASD |
|  | Patient 20 | VSD，ASD |  | Patient 20 | ASD，PDA，TI |
|  |  |  |  | Patient 21 | VSD，ASD，PI |
