# Supplementary material for: Plasma Proteomic Study in Pulmonary Arterial Hypertension Associated with Congenital Heart Diseases
Source: Sci Rep. 2016 Nov 25;6:36541. doi: 10.1038/srep36541 (PMC5122864; doi:10.1038/srep36541)
Supplement: Supplementary File S2 [file srep36541-s3.doc]

**Plasma Proteomic Study in Pulmonary Arterial Hypertension Associated with Congenital Heart Diseases**

**Xi Zhang,** *PhD***, Hai-Tao Hou,** *MD,* **Jun Wang,** *MD*, **Xiao-Cheng Liu,** *MD*, **Qin Yang,** *PhD*, **and Guo-Wei He*,** *PhD, DSc*

VSD-PH vs. Control

| Accession | Description | Score | Coverage | # Proteins | # Unique Peptides | # Peptides | # PSMs | # AAs | MW [kDa] | calc. pI | VSD-PH/Control |
| --- | --- | --- | --- | --- | --- | --- | --- | --- | --- | --- | --- |
| 13195586 | hemoglobin alpha 1 globin chain [Homo sapiens] | 103.91 | 24.00% | 13 | 2 | 2 | 25 | 100 | 10.7 | 7.72 | 0.148 |
| 18418633 | mutant beta-globin [Homo sapiens] | 289.03 | 71.43% | 76 | 7 | 8 | 59 | 147 | 16 | 7.69 | 0.228 |
| 124504316 | HIST2H4B protein [Homo sapiens] | 46.54 | 9.80% | 6 | 1 | 1 | 1 | 102 | 11.4 | 11.36 | 0.266 |
| 21669937 | immunoglobulin heavy chain VHDJ region [Homo sapiens] | 114.57 | 18.55% | 423 | 1 | 2 | 11 | 124 | 13.5 | 9.36 | 0.268 |
| 194375974 | unnamed protein product [Homo sapiens] | 60.43 | 9.84% | 5 | 2 | 2 | 3 | 183 | 20.1 | 8.78 | 0.277 |
| 36321 | SAA precursor polypeptide (119 AA) [Homo sapiens] | 245.07 | 52.10% | 10 | 2 | 5 | 45 | 119 | 13.1 | 8.18 | 0.318 |
| 76252669 | immunoglobulin lambda light chain variable region [Homo sapiens] | 33.83 | 17.65% | 2 | 1 | 1 | 4 | 102 | 10.9 | 7.12 | 0.343 |
| 13937839 | SAA1 protein [Homo sapiens] >gi|123983058|gb|ABM83270.1| serum amyloid A1 [synthetic construct] >gi|123983248|gb|ABM83365.1| serum amyloid A1 [synthetic construct] >gi|123997747|gb|ABM86475.1| serum amyloid A1 [synthetic construct] >gi|157928044|gb|ABW03318.1| serum amyloid A1 [synthetic construct] | 249.56 | 54.10% | 6 | 2 | 5 | 58 | 122 | 13.5 | 6.32 | 0.346 |
| 98956272 | immunoglobulin kappa light chain variable region [Homo sapiens] | 114.64 | 46.79% | 107 | 1 | 3 | 5 | 109 | 11.8 | 9.29 | 0.348 |
| 183851 | G-gamma-hemoglobin [Homo sapiens] | 64.89 | 22.77% | 15 | 1 | 2 | 21 | 101 | 11 | 6.68 | 0.374 |
| 139641 | RecName: Full=Vitamin D-binding protein; Short=DBP; Short=VDB; AltName: Full=Group-specific component; AltName: Full=Gc-globulin; Flags: Precursor >gi|31676|emb|CAA26938.1| unnamed protein product [Homo sapiens] | 812.8 | 58.44% | 1 | 1 | 22 | 73 | 474 | 52.9 | 5.54 | 0.385 |
| 55958543 | heterogeneous nuclear ribonucleoprotein K [Homo sapiens] | 39.26 | 5.23% | 12 | 1 | 1 | 1 | 306 | 34 | 5.78 | 0.392 |
| 189066534 | unnamed protein product [Homo sapiens] | 76.02 | 20.00% | 4 | 2 | 2 | 3 | 175 | 20 | 9.01 | 0.439 |
| 186083 | immunoglobulin lambda-chain [Homo sapiens] | 82.87 | 21.17% | 350 | 1 | 3 | 29 | 137 | 14.3 | 7.24 | 0.444 |
| 32483410 | vitamin D-binding protein precursor [Homo sapiens] | 803.24 | 58.86% | 3 | 1 | 22 | 75 | 474 | 52.9 | 5.45 | 0.482 |
| 1064908 | complement Factor H-related Protein 2 [Homo sapiens] | 222.48 | 31.28% | 3 | 3 | 6 | 38 | 243 | 27.9 | 6.92 | 0.497 |
| 3152372 | anti-FactorVIII scFv [Homo sapiens] | 537.51 | 64.29% | 3758 | 1 | 10 | 90 | 238 | 25 | 8.41 | 0.499 |
| 56378229 | carbamoylphosphate synthetase I [Homo sapiens] | 22.22 | 4.09% | 12 | 1 | 1 | 1 | 513 | 55.7 | 8.94 | 0.503 |
| 31873302 | hypothetical protein [Homo sapiens] >gi|117646030|emb|CAL38482.1| hypothetical protein [synthetic construct] | 241.85 | 24.19% | 27 | 7 | 7 | 15 | 434 | 47.1 | 7.69 | 0.522 |
| 47124510 | APCS protein [Homo sapiens] | 30.38 | 23.91% | 3 | 1 | 1 | 2 | 46 | 5.3 | 4.46 | 0.532 |
| 119588814 | serum amyloid A1, isoform CRA_a [Homo sapiens] >gi|119588815|gb|EAW68409.1| serum amyloid A1, isoform CRA_a [Homo sapiens] >gi|119588817|gb|EAW68411.1| serum amyloid A1, isoform CRA_a [Homo sapiens] | 350.08 | 54.10% | 9 | 3 | 6 | 71 | 122 | 13.6 | 6.79 | 0.538 |
| 119608546 | ficolin (collagen/fibrinogen domain containing) 1, isoform CRA_d [Homo sapiens] | 57.63 | 5.10% | 7 | 1 | 2 | 2 | 314 | 33.8 | 6.64 | 0.538 |
| 71773201 | adenine phosphoribosyltransferase isoform b [Homo sapiens] >gi|114664113|ref|XP_001137924.1| PREDICTED: adenine phosphoribosyltransferase isoform 1 [Pan troglodytes] >gi|119587165|gb|EAW66761.1| adenine phosphoribosyltransferase, isoform CRA_b [Homo sapiens] | 26.87 | 14.93% | 2 | 1 | 1 | 1 | 134 | 14.5 | 7.34 | 0.539 |
| 119608880 | hCG1979429, isoform CRA_a [Homo sapiens] | 16.53 | 0.69% | 1 | 1 | 1 | 1 | 873 | 98.1 | 9.29 | 0.542 |
| 178812 | apolipoprotein B-100 precursor [Homo sapiens] | 1998.27 | 17.34% | 21 | 1 | 59 | 189 | 4563 | 515.1 | 7.11 | 0.544 |
| 4505733 | platelet factor 4 precursor [Homo sapiens] >gi|130304|sp|P02776.2|PLF4_HUMAN RecName: Full=Platelet factor 4; Short=PF-4; AltName: Full=C-X-C motif chemokine 4; AltName: Full=Oncostatin-A; AltName: Full=Iroplact; Contains: RecName: Full=Platelet factor 4, short form; Flags: Precursor >gi|13549118|gb|AAK29643.1|AF349466_3 platelet factor 4 [Homo sapiens] >gi|189851|gb|AAA60066.1| platelet factor 4 [Homo sapiens] >gi|47115291|emb|CAG28605.1| PF4 [Homo sapiens] >gi|62739642|gb|AAH93965.1| Platelet factor 4 [Homo sapiens] >gi|63994325|gb|AAY41003.1| unknown [Homo sapiens] >gi|85567544|gb|AAI12094.1| Platelet factor 4 [Homo sapiens] >gi|119626099|gb|EAX05694.1| platelet factor 4 (chemokine (C-X-C motif) ligand 4) [Homo sapiens] >gi|261859268|dbj|BAI46156.1| platelet factor 4 [synthetic construct] | 125.57 | 34.65% | 1 | 1 | 3 | 22 | 101 | 10.8 | 8.62 | 0.545 |
| 169672524 | transforming growth factor beta 1 precursor [Homo sapiens] | 51.7 | 16.10% | 7 | 1 | 1 | 1 | 118 | 12.9 | 7.2 | 0.546 |
| 119626442 | multimerin 1, isoform CRA_a [Homo sapiens] | 62.55 | 3.58% | 6 | 1 | 1 | 5 | 531 | 58.1 | 8.72 | 0.547 |
| 119594857 | cofilin 1 (non-muscle), isoform CRA_c [Homo sapiens] | 91.11 | 40.88% | 6 | 4 | 4 | 5 | 137 | 15.6 | 8.35 | 0.555 |
| 183763 | factor H homologue [Homo sapiens] >gi|158255096|dbj|BAF83519.1| unnamed protein product [Homo sapiens] | 511 | 37.58% | 4 | 1 | 12 | 142 | 330 | 37.6 | 7.56 | 0.571 |
| 3337390 | haptoglobin [Homo sapiens] | 399.49 | 39.42% | 15 | 5 | 12 | 56 | 345 | 38.2 | 6.6 | 0.574 |
| 34532317 | unnamed protein product [Homo sapiens] | 19.57 | 0.89% | 9 | 1 | 1 | 1 | 1014 | 113.4 | 9.03 | 0.576 |
| 1769552 | von Willebrand factor [Homo sapiens] | 27.48 | 3.73% | 10 | 1 | 1 | 2 | 241 | 27.1 | 6.93 | 0.582 |
| 74355107 | BRF1 protein [Homo sapiens] | 30.09 | 4.97% | 11 | 1 | 1 | 11 | 161 | 18.3 | 8.62 | 0.606 |
| 6707433 | apolipoprotein A5 [Homo sapiens] >gi|6707435|gb|AAF25662.1|AF202890_1 apolipoprotein A5 [Homo sapiens] >gi|167887490|gb|ACA05939.1| apolipoprotein A-V precursor variant 3 [Homo sapiens] >gi|189054886|dbj|BAG37658.1| unnamed protein product [Homo sapiens] | 30.29 | 6.34% | 4 | 1 | 1 | 1 | 363 | 40.9 | 6.43 | 0.607 |
| 146424184 | apolipoprotein C-IV [Homo sapiens] | 73.46 | 15.75% | 3 | 2 | 2 | 5 | 127 | 14.6 | 9.13 | 0.611 |
| 105990532 | apolipoprotein B-100 precursor [Homo sapiens] >gi|260158878|gb|ACX32319.1| apolipoprotein B precursor [synthetic construct] | 2158.39 | 18.58% | 36 | 5 | 63 | 209 | 4563 | 515.2 | 7.05 | 0.627 |
| 119570830 | hCG1741471 [Homo sapiens] | 23 | 3.11% | 7 | 1 | 1 | 2 | 322 | 37.2 | 7.99 | 0.631 |
| 194375299 | unnamed protein product [Homo sapiens] | 265.15 | 33.33% | 58 | 8 | 8 | 22 | 333 | 37.3 | 5.71 | 0.64 |
| 49354849 | immunoglobulin E variable region [Homo sapiens] | 135.35 | 38.02% | 272 | 1 | 3 | 19 | 121 | 13.2 | 8.43 | 0.642 |
| 896272 | This CDS feature is included to show the translation of the corresponding V_region. Presently translation qualifiers on V_region features are illegal [Homo sapiens] | 285.25 | 64.57% | 2946 | 2 | 9 | 63 | 127 | 13.8 | 8.79 | 0.647 |
| 114062 | RecName: Full=Apolipoprotein(a); Short=Apo(a); Short=Lp(a); Flags: Precursor >gi|28620|emb|CAA29618.1| unnamed protein product [Homo sapiens] | 78.87 | 8.60% | 4 | 2 | 2 | 7 | 4548 | 501 | 5.88 | 0.651 |
| 46254055 | immunoglobulin heavy chain [Homo sapiens] | 151.53 | 28.74% | 208 | 1 | 3 | 50 | 167 | 18 | 8.9 | 0.657 |
| 54304028 | glyceraldehyde-3-phosphate dehydrogenase [Homo sapiens] | 45.44 | 17.44% | 7 | 1 | 1 | 3 | 86 | 9.2 | 9.72 | 0.679 |
| 284434903 | thrombocidin-2 antimicrobial variant [Homo sapiens] | 56.55 | 19.84% | 4 | 2 | 2 | 3 | 126 | 13.7 | 9 | 0.685 |
| 119625314 | fibrinogen gamma chain, isoform CRA_e [Homo sapiens] | 1193.9 | 62.53% | 5 | 6 | 27 | 1576 | 419 | 47.4 | 5.95 | 0.687 |
| 119590943 | fibronectin 1, isoform CRA_h [Homo sapiens] | 2674.5 | 37.42% | 82 | 36 | 60 | 502 | 2330 | 256.3 | 5.8 | 0.689 |
| 119598593 | alpha-2-HS-glycoprotein, isoform CRA_a [Homo sapiens] | 532.05 | 46.99% | 2 | 1 | 11 | 318 | 366 | 39.2 | 5.72 | 0.689 |
| 158256710 | unnamed protein product [Homo sapiens] | 235.85 | 9.06% | 11 | 8 | 8 | 18 | 1170 | 129.3 | 4.94 | 0.695 |
| 126273569 | carboxypeptidase B2 isoform a preproprotein [Homo sapiens] >gi|62899885|sp|Q96IY4.1|CBPB2_HUMAN RecName: Full=Carboxypeptidase B2; AltName: Full=Carboxypeptidase U; Short=CPU; AltName: Full=Thrombin-activable fibrinolysis inhibitor; Short=TAFI; AltName: Full=Plasma carboxypeptidase B; Short=pCPB; Flags: Precursor >gi|13937897|gb|AAH07057.1| Carboxypeptidase B2 (plasma) [Homo sapiens] >gi|30582711|gb|AAP35582.1| carboxypeptidase B2 (plasma, carboxypeptidase U) [Homo sapiens] >gi|51234145|gb|AAT97987.1| carboxypeptidase B2 (plasma, carboxypeptidase U) [Homo sapiens] >gi|60656513|gb|AAX32820.1| carboxypeptidase B2 [synthetic construct] >gi|60656515|gb|AAX32821.1| carboxypeptidase B2 [synthetic construct] >gi|119629160|gb|EAX08755.1| carboxypeptidase B2 (plasma, carboxypeptidase U), isoform CRA_b [Homo sapiens] | 34.26 | 1.89% | 3 | 1 | 1 | 3 | 423 | 48.4 | 7.71 | 0.701 |
| 119625310 | fibrinogen gamma chain, isoform CRA_a [Homo sapiens] >gi|119625317|gb|EAX04912.1| fibrinogen gamma chain, isoform CRA_a [Homo sapiens] >gi|119625323|gb|EAX04918.1| fibrinogen gamma chain, isoform CRA_a [Homo sapiens] >gi|119625328|gb|EAX04923.1| fibrinogen gamma chain, isoform CRA_a [Homo sapiens] | 1199.64 | 68.56% | 25 | 2 | 28 | 1123 | 334 | 37.7 | 6.29 | 0.705 |
| 18025670 | immunoglobulin light chain variable region [Homo sapiens] | 66.85 | 25.23% | 17 | 1 | 2 | 4 | 107 | 11.8 | 9.01 | 0.709 |
| 158255874 | unnamed protein product [Homo sapiens] | 487.27 | 17.28% | 9 | 12 | 12 | 53 | 1065 | 122.1 | 5.74 | 0.71 |
| 115298678 | complement C3 precursor [Homo sapiens] >gi|119370332|sp|P01024.2|CO3_HUMAN RecName: Full=Complement C3; AltName: Full=C3 and PZP-like alpha-2-macroglobulin domain-containing protein 1; Contains: RecName: Full=Complement C3 beta chain; Contains: RecName: Full=Complement C3 alpha chain; Contains: RecName: Full=C3a anaphylatoxin; Contains: RecName: Full=Complement C3b alpha' chain; Contains: RecName: Full=Complement C3c alpha' chain fragment 1; Contains: RecName: Full=Complement C3dg fragment; Contains: RecName: Full=Complement C3g fragment; Contains: RecName: Full=Complement C3d fragment; Contains: RecName: Full=Complement C3f fragment; Contains: RecName: Full=Complement C3c alpha' chain fragment 2; Flags: Precursor >gi|40786791|gb|AAR89906.1| complement component 3 [Homo sapiens] >gi|119589477|gb|EAW69071.1| complement component 3, isoform CRA_b [Homo sapiens] >gi|152012494|gb|AAI50201.1| Complement component 3 [Homo sapiens] >gi|152012784|gb|AAI50180.1| Complement component 3 [Homo sapiens] | 3018.01 | 58.15% | 7 | 76 | 76 | 444 | 1663 | 187 | 6.4 | 0.711 |
| 10835095 | serum amyloid A-4 protein precursor [Homo sapiens] >gi|259352|gb|AAB24060.1| serum amyloid A [Homo sapiens] >gi|337750|gb|AAA60298.1| serum amyloid A protein [Homo sapiens] >gi|13937846|gb|AAH07026.1| Serum amyloid A4, constitutive [Homo sapiens] >gi|49456475|emb|CAG46558.1| SAA4 [Homo sapiens] >gi|119588821|gb|EAW68415.1| serum amyloid A4, constitutive [Homo sapiens] | 94.08 | 23.08% | 2 | 3 | 3 | 18 | 130 | 14.8 | 9.23 | 0.713 |
| 189067450 | unnamed protein product [Homo sapiens] | 26.83 | 2.55% | 1 | 1 | 1 | 4 | 275 | 32.3 | 8.37 | 0.713 |
| 221042312 | unnamed protein product [Homo sapiens] | 233.94 | 15.07% | 15 | 4 | 4 | 12 | 564 | 60 | 5.74 | 0.714 |
| 587273 | Immunoglobulin heavy chain variable region [Homo sapiens] | 96.48 | 27.27% | 59 | 1 | 2 | 27 | 132 | 14 | 8.46 | 0.716 |
| 21669315 | immunoglobulin kappa light chain VLJ region [Homo sapiens] | 492.07 | 37.93% | 58 | 1 | 8 | 576 | 261 | 28.2 | 7.3 | 0.722 |
| 118442839 | complement factor H-related protein 1 precursor [Homo sapiens] >gi|239758113|ref|XP_002346300.1| PREDICTED: similar to complement factor H-related 1 isoform 1 [Homo sapiens] >gi|218512041|sp|Q03591.2|FHR1_HUMAN RecName: Full=Complement factor H-related protein 1; Short=FHR-1; AltName: Full=H factor-like protein 1; Short=H-factor-like 1; AltName: Full=H36; Flags: Precursor >gi|183765|gb|AAA35947.1| factor H homologue [Homo sapiens] >gi|5748573|emb|CAB53063.1| complement factor H-related 1 [Homo sapiens] >gi|16876961|gb|AAH16755.1| Complement factor H-related 1 [Homo sapiens] >gi|78070360|gb|AAI07772.1| Complement factor H-related 1 [Homo sapiens] >gi|123984685|gb|ABM83688.1| complement factor H-related 1 [synthetic construct] >gi|123998684|gb|ABM86991.1| complement factor H-related 1 [synthetic construct] | 510.55 | 37.58% | 3 | 1 | 12 | 147 | 330 | 37.6 | 7.39 | 0.724 |
| 553293 | fibronectin [Homo sapiens] | 54.43 | 36.73% | 1 | 1 | 1 | 3 | 49 | 5 | 11.11 | 0.726 |
| 119573007 | apolipoprotein A-II, isoform CRA_d [Homo sapiens] | 478.54 | 75.00% | 4 | 2 | 9 | 695 | 92 | 10.6 | 9.04 | 0.726 |
| 182442 | gamma fibrinogen type A (AA at 202) [Homo sapiens] | 226.09 | 38.56% | 3 | 1 | 5 | 129 | 153 | 17.1 | 5.77 | 0.73 |
| 119570453 | retinol binding protein 4, plasma, isoform CRA_b [Homo sapiens] | 132.32 | 15.08% | 10 | 3 | 3 | 14 | 199 | 23 | 6.09 | 0.731 |
| 112910 | RecName: Full=Alpha-2-HS-glycoprotein; AltName: Full=Ba-alpha-2-glycoprotein; AltName: Full=Alpha-2-Z-globulin; AltName: Full=Fetuin-A; Contains: RecName: Full=Alpha-2-HS-glycoprotein chain A; Contains: RecName: Full=Alpha-2-HS-glycoprotein chain B; Flags: Precursor >gi|178284|gb|AAA51683.1| alpha-2-HS-glycoprotein [Homo sapiens] >gi|499139|gb|AAB29984.1| alpha 2-HS-glycoprotein, alpha 2HSG=insulin receptor inhibitor [human, liver, Peptide, 367 aa] >gi|7106502|dbj|BAA92189.1| alpha2-HS glycoprotein [Homo sapiens] >gi|29387000|gb|AAH48198.1| Alpha-2-HS-glycoprotein [Homo sapiens] >gi|30851645|gb|AAH52590.1| Alpha-2-HS-glycoprotein [Homo sapiens] >gi|119598595|gb|EAW78189.1| alpha-2-HS-glycoprotein, isoform CRA_c [Homo sapiens] >gi|189066558|dbj|BAG35808.1| unnamed protein product [Homo sapiens] | 544.15 | 47.14% | 8 | 1 | 11 | 343 | 367 | 39.3 | 5.72 | 0.731 |
| 189231244 | anti-fluorescein immunoglobulin heavy chain variable region [Homo sapiens] | 128.41 | 33.61% | 369 | 1 | 3 | 12 | 119 | 12.9 | 8.46 | 0.732 |
| 3169770 | immunoglobulin kappa light chain [Homo sapiens] | 456.88 | 40.28% | 201 | 1 | 8 | 707 | 211 | 23 | 8.05 | 0.733 |
| 37789448 | immunoglobulin lambda light chain variable region [Homo sapiens] | 21.04 | 9.64% | 4 | 1 | 1 | 1 | 83 | 8.9 | 7.12 | 0.739 |
| 82734214 | beta-defensin 110 isoform a [Homo sapiens] >gi|84028871|sp|Q30KQ9.1|DB110_HUMAN RecName: Full=Beta-defensin 110; AltName: Full=Defensin, beta 110; AltName: Full=Beta-defensin 10; Short=DEFB-10; AltName: Full=Beta-defensin 111; AltName: Full=Defensin, beta 111; AltName: Full=Beta-defensin 11; Short=DEFB-11; Flags: Precursor >gi|66968904|gb|AAY59751.1| beta-defensin 111 [Homo sapiens] >gi|151555475|gb|AAI48542.1| Defensin, beta 111 [synthetic construct] >gi|162319076|gb|AAI56745.1| Defensin, beta 111 [synthetic construct] | 23.5 | 14.93% | 1 | 1 | 1 | 11 | 67 | 8 | 8.73 | 0.75 |
| 247424364 | immunoglobulin heavy chain variable region [Homo sapiens] | 165.61 | 50.81% | 1078 | 1 | 5 | 28 | 124 | 13.4 | 8.91 | 0.75 |
| 207028494 | L-lactate dehydrogenase A chain isoform 2 [Homo sapiens] >gi|194383812|dbj|BAG59264.1| unnamed protein product [Homo sapiens] | 36.64 | 7.30% | 5 | 1 | 1 | 3 | 274 | 30.2 | 7.15 | 0.751 |
| 632988 | immunoglobulin kappa chain [Homo sapiens] | 70.06 | 28.68% | 184 | 1 | 3 | 7 | 129 | 14 | 8.29 | 0.751 |
| 119625338 | fibrinogen beta chain, isoform CRA_d [Homo sapiens] | 1669.35 | 67.61% | 7 | 24 | 34 | 2036 | 457 | 52.1 | 8.07 | 0.753 |
| 2258128 | complement 9 [Homo sapiens] | 51.17 | 4.50% | 5 | 2 | 2 | 3 | 533 | 60.4 | 5.59 | 0.757 |
| 224979536 | anti-IL-15 immunoglobulin heavy chain variable region 1 [Homo sapiens] | 21.9 | 16.10% | 1 | 1 | 1 | 2 | 118 | 12.7 | 7.12 | 0.76 |
| 63103087 | anti-rabies virus immunoglobulin light chain variable region [Homo sapiens] | 129.26 | 31.78% | 10 | 1 | 2 | 11 | 107 | 11.5 | 6.54 | 0.762 |
| 215982756 | immunoglobulin kappa light chain variable region [Homo sapiens] | 318.72 | 60.40% | 368 | 1 | 7 | 345 | 149 | 16.3 | 7.81 | 0.769 |
| 112701317 | immunoglobulin heavy chain variable region [Homo sapiens] | 63.94 | 32.32% | 36 | 1 | 2 | 3 | 99 | 10.6 | 6.51 | 0.77 |
| 189054579 | unnamed protein product [Homo sapiens] | 40.06 | 7.67% | 2 | 2 | 2 | 3 | 352 | 38.2 | 7.11 | 0.77 |
| 30583505 | catenin (cadherin-associated protein), delta 1 [Homo sapiens] | 42.11 | 3.28% | 25 | 1 | 1 | 1 | 610 | 68 | 8.13 | 0.771 |
| 28931 | beta-subunit (AA 1-312) [Homo sapiens] | 29.34 | 7.69% | 4 | 1 | 1 | 3 | 312 | 34 | 5.03 | 0.773 |
| 180271 | cholesteryl ester transfer protein [Homo sapiens] | 130.3 | 8.00% | 7 | 3 | 3 | 4 | 425 | 47 | 6.01 | 0.773 |
| 31874240 | hypothetical protein [Homo sapiens] | 1112.51 | 62.16% | 23 | 1 | 27 | 1104 | 399 | 45.1 | 6.16 | 0.774 |
| 182839 | factor XIII b subunit precursor [Homo sapiens] | 581.51 | 31.52% | 5 | 16 | 16 | 51 | 660 | 75.3 | 6.37 | 0.774 |
| 13540563 | complement factor H-related protein 5 precursor [Homo sapiens] >gi|23396597|sp|Q9BXR6.1|FHR5_HUMAN RecName: Full=Complement factor H-related protein 5; Short=FHR-5; Flags: Precursor >gi|13195239|gb|AAK15619.1|AF295327_1 complement factor H-related protein 5 [Homo sapiens] >gi|84627551|gb|AAI11774.1| Complement factor H-related 5 [Homo sapiens] >gi|119611674|gb|EAW91268.1| complement factor H-related 5 [Homo sapiens] | 305.8 | 19.86% | 4 | 9 | 10 | 60 | 569 | 64.4 | 7.06 | 0.775 |
| 118406205 | immunoglobulin heavy chain variable region [Homo sapiens] | 58.92 | 22.22% | 231 | 1 | 2 | 7 | 117 | 12.5 | 8.68 | 0.775 |
| 158256226 | unnamed protein product [Homo sapiens] | 1927.17 | 45.90% | 5 | 1 | 46 | 523 | 1231 | 138.9 | 6.71 | 0.777 |
| 184086 | histone H2B.1 [Homo sapiens] | 30.74 | 8.91% | 34 | 1 | 1 | 6 | 101 | 11.3 | 10.14 | 0.783 |
| 62739186 | complement factor H isoform a precursor [Homo sapiens] >gi|56203410|emb|CAI19672.1| complement factor H [Homo sapiens] >gi|119611666|gb|EAW91260.1| hCG40889, isoform CRA_b [Homo sapiens] >gi|119611667|gb|EAW91261.1| hCG40889, isoform CRA_b [Homo sapiens] | 1928.15 | 45.90% | 10 | 1 | 46 | 524 | 1231 | 139 | 6.62 | 0.792 |
| 84798296 | immunoglobulin light chain variable region YV1-4-K4-3 [Homo sapiens] | 58.21 | 23.68% | 4 | 1 | 2 | 10 | 114 | 12.5 | 5.36 | 0.796 |
| 189053338 | unnamed protein product [Homo sapiens] | 25.83 | 4.48% | 5 | 1 | 1 | 3 | 201 | 23.6 | 5.21 | 0.802 |
| 11761629 | fibrinogen alpha chain isoform alpha preproprotein [Homo sapiens] >gi|182426|gb|AAA52427.1| A-alpha fibrinogen [Homo sapiens] >gi|458554|gb|AAA17055.1| common fibrinogen alpha chain [Homo sapiens] >gi|4033511|gb|AAC97143.1| fibrinogen alpha subunit [Homo sapiens] >gi|67514250|gb|AAH98280.1| Fibrinogen alpha chain [Homo sapiens] >gi|71043421|gb|AAH99720.1| Fibrinogen alpha chain [Homo sapiens] >gi|71043479|gb|AAH99706.1| Fibrinogen, alpha chain, isoform alpha preproprotein [Homo sapiens] >gi|74355612|gb|AAI01936.1| Fibrinogen alpha chain [Homo sapiens] >gi|119625332|gb|EAX04927.1| fibrinogen alpha chain, isoform CRA_c [Homo sapiens] >gi|119625333|gb|EAX04928.1| fibrinogen alpha chain, isoform CRA_c [Homo sapiens] | 2376.76 | 58.07% | 12 | 45 | 45 | 2395 | 644 | 69.7 | 8.06 | 0.803 |
| 577055 | gamma-fibrinogen chain fragment [Homo sapiens] | 255.24 | 77.42% | 1 | 1 | 6 | 151 | 62 | 7 | 6.52 | 0.804 |
| 1655598 | lipopolysaccharide binding protein [Homo sapiens] >gi|4530277|gb|AAD21962.1| lipopolysaccharide-binding protein [Homo sapiens] | 464.59 | 23.70% | 7 | 10 | 10 | 75 | 481 | 53.3 | 6.7 | 0.804 |
| 158258641 | unnamed protein product [Homo sapiens] | 22.67 | 5.86% | 14 | 1 | 1 | 1 | 222 | 25.7 | 9 | 0.807 |
| 33319096 | Ig heavy chain variable region, VH3 family [Homo sapiens] | 38.9 | 20.97% | 231 | 1 | 2 | 7 | 124 | 13.6 | 7.94 | 0.809 |
| 77379566 | immunoglobulin kappa chain variable region [Homo sapiens] | 76.5 | 22.22% | 418 | 1 | 2 | 25 | 108 | 12 | 8.81 | 0.811 |
| 2809025 | Ig heavy chain variable region [Homo sapiens] | 41.55 | 42.25% | 134 | 1 | 2 | 4 | 71 | 7.9 | 9.31 | 0.814 |
| 4758146 | neutrophil defensin 1 preproprotein [Homo sapiens] >gi|124248516|ref|NP_001035965.1| alpha-defensin 1 [Homo sapiens] >gi|30316322|sp|P59665.1|DEF1_HUMAN RecName: Full=Neutrophil defensin 1; AltName: Full=HNP-1; Short=HP-1; Short=HP1; AltName: Full=Defensin, alpha 1; Contains: RecName: Full=HP 1-56; Contains: RecName: Full=Neutrophil defensin 2; AltName: Full=HNP-2; Short=HP-2; Short=HP2; Flags: Precursor >gi|32402|emb|CAA36280.1| unnamed protein product [Homo sapiens] >gi|181527|gb|AAA52302.1| neutrophil peptide 1 precursor [Homo sapiens] >gi|181529|gb|AAA52303.1| defensin 1 [Homo sapiens] >gi|292363|gb|AAA36382.1| neutrophil peptide-1 [Homo sapiens] >gi|46854592|gb|AAH69423.1| Defensin, alpha 1 [Homo sapiens] >gi|50057839|gb|AAT68875.1| novel protein, similar to DEFA1 [Homo sapiens] >gi|50057842|gb|AAT68878.1| defensin, alpha 1, myeloid-related sequence [Homo sapiens] >gi|50057843|gb|AAT68879.1| novel protein, similar to DEFA1 [Homo sapiens] >gi|50057844|gb|AAT68880.1| novel protein, similar to DEFA1 [Homo sapiens] >gi|50057847|gb|AAT68883.1| defensin, alpha 1, myeloid-related sequence [Homo sapiens] >gi|50057848|gb|AAT68884.1| defensin, alpha 1, myeloid-related sequence [Homo sapiens] >gi|62739977|gb|AAH93791.1| Defensin, alpha 1 [Homo sapiens] >gi|85567619|gb|AAI12189.1| Defensin, alpha 1, preproprotein [Homo sapiens] >gi|446635|prf||1912193A defensin >gi|1098031|prf||2115200A neutrophil peptide | 43.25 | 32.98% | 2 | 1 | 1 | 2 | 94 | 10.2 | 6.99 | 0.815 |
| 40737478 | C4A3 [Homo sapiens] >gi|40737480|gb|AAR89158.1| C4A [Homo sapiens] >gi|40737484|gb|AAR89160.1| C4A3 [Homo sapiens] | 933.23 | 54.49% | 13 | 1 | 21 | 260 | 534 | 58.4 | 5.99 | 0.819 |
| 194385488 | unnamed protein product [Homo sapiens] | 67.07 | 4.23% | 18 | 2 | 2 | 2 | 780 | 82.5 | 6.55 | 0.82 |
| 1620018 | heat shock protein 90 [Homo sapiens] | 69.98 | 17.88% | 10 | 1 | 1 | 4 | 151 | 16.8 | 4.79 | 0.822 |
| 4379074 | IgM rheumatoid factor immunoglobulin heavy chain [Homo sapiens] | 104.17 | 39.32% | 775 | 1 | 3 | 23 | 117 | 12.8 | 8.4 | 0.826 |
| 14030460 | mannan-binding lectin MBL precursor [Homo sapiens] | 176.08 | 21.37% | 5 | 6 | 6 | 13 | 248 | 26.1 | 5.49 | 0.826 |
| 21669387 | immunoglobulin kappa light chain VLJ region [Homo sapiens] | 483.16 | 34.62% | 28 | 1 | 8 | 553 | 286 | 30.5 | 7.36 | 0.831 |
| 262231791 | complement factor H-related protein 3 isoform 2 precursor [Homo sapiens] >gi|194391108|dbj|BAG60672.1| unnamed protein product [Homo sapiens] | 53.08 | 7.43% | 12 | 2 | 2 | 7 | 269 | 30.7 | 7.78 | 0.832 |
| 4557389 | complement component C8 alpha chain precursor [Homo sapiens] >gi|729167|sp|P07357.2|CO8A_HUMAN RecName: Full=Complement component C8 alpha chain; AltName: Full=Complement component 8 subunit alpha; Flags: Precursor >gi|179718|gb|AAA52200.1| complement protein C8 alpha subunit precursor [Homo sapiens] >gi|56203387|emb|CAI19172.1| complement component 8, alpha polypeptide [Homo sapiens] >gi|124376216|gb|AAI32914.1| Complement component 8, alpha polypeptide [Homo sapiens] | 83.59 | 7.71% | 7 | 3 | 3 | 4 | 584 | 65.1 | 6.47 | 1.2 |
| 33235614 | immunoglobulin kappa light chain [Homo sapiens] | 93.69 | 31.48% | 464 | 1 | 2 | 17 | 108 | 11.7 | 9.17 | 1.208 |
| 62999490 | immunoglobulin lambda light chain variable region [Homo sapiens] | 34.39 | 7.21% | 2 | 1 | 1 | 2 | 111 | 11.5 | 5.25 | 1.208 |
| 86438992 | immunoglobulin heavy chain [Homo sapiens] | 128.3 | 28.45% | 838 | 1 | 3 | 26 | 116 | 12.8 | 7.97 | 1.211 |
| 86438980 | immunoglobulin heavy chain [Homo sapiens] | 193.56 | 22.37% | 31 | 1 | 4 | 51 | 219 | 23.5 | 8.91 | 1.213 |
| 194390048 | unnamed protein product [Homo sapiens] | 349.81 | 47.06% | 17 | 9 | 9 | 31 | 272 | 30.8 | 6.6 | 1.213 |
| 4557325 | apolipoprotein E precursor [Homo sapiens] >gi|114039|sp|P02649.1|APOE_HUMAN RecName: Full=Apolipoprotein E; Short=Apo-E; Flags: Precursor >gi|178851|gb|AAB59546.1| preapolipoprotein E [Homo sapiens] >gi|4105704|gb|AAD02505.1| apolipoprotein E [Homo sapiens] >gi|11034801|gb|AAG27089.1| apolipoprotein-E [Homo sapiens] >gi|13097699|gb|AAH03557.1| Apolipoprotein E [Homo sapiens] >gi|123983676|gb|ABM83474.1| apolipoprotein E [synthetic construct] >gi|123998167|gb|ABM86685.1| apolipoprotein E [synthetic construct] >gi|189054611|dbj|BAG37412.1| unnamed protein product [Homo sapiens] >gi|208965826|dbj|BAG72927.1| apolipoprotein E [synthetic construct] >gi|225131031|gb|ACN81314.1| apolipoprotein E [Homo sapiens] | 1065.04 | 58.36% | 9 | 20 | 20 | 272 | 317 | 36.1 | 5.73 | 1.217 |
| 54780200 | immunoglobulin mu heavy chain [Homo sapiens] | 270.49 | 33.18% | 525 | 1 | 5 | 66 | 211 | 22.7 | 8.29 | 1.217 |
| 189053554 | unnamed protein product [Homo sapiens] | 125.23 | 20.08% | 2 | 3 | 3 | 23 | 244 | 26.4 | 5.74 | 1.218 |
| 1082521 | Ig kappa chain V region - human (fragment) >gi|809555|emb|CAA58108.1| immunoglobulin kappa light chain [Homo sapiens] | 87.06 | 36.84% | 16 | 1 | 3 | 3 | 114 | 12.3 | 8.94 | 1.219 |
| 221042196 | unnamed protein product [Homo sapiens] | 99.51 | 6.36% | 8 | 3 | 3 | 14 | 645 | 72.1 | 7.68 | 1.222 |
| 189069199 | unnamed protein product [Homo sapiens] | 652.37 | 45.91% | 15 | 18 | 18 | 56 | 464 | 52.6 | 6.71 | 1.228 |
| 221044808 | unnamed protein product [Homo sapiens] | 419.54 | 29.03% | 4 | 11 | 11 | 74 | 534 | 59.8 | 6.43 | 1.234 |
| 12733966 | immunoglobulin gamma heavy chain variable region [Homo sapiens] | 167.4 | 39.32% | 1183 | 1 | 4 | 41 | 117 | 13 | 6.04 | 1.239 |
| 33319650 | Ig heavy chain variable region, VH3 family [Homo sapiens] | 100.27 | 34.17% | 151 | 1 | 3 | 5 | 120 | 13.1 | 6.52 | 1.242 |
| 55669575 | Chain A, A Covalent Dimer Of Transthyretin That Affects The Amyloid Pathway >gi|55669576|pdb|1QWH|B Chain B, A Covalent Dimer Of Transthyretin That Affects The Amyloid Pathway >gi|339685|gb|AAA61181.1| transthyretin [Homo sapiens] | 291.36 | 66.67% | 11 | 7 | 7 | 86 | 117 | 12.8 | 5.45 | 1.244 |
| 4960066 | apolipoprotein A1 [Homo sapiens] | 352.13 | 79.10% | 83 | 1 | 9 | 252 | 67 | 7.4 | 6.95 | 1.253 |
| 755742 | unnamed protein product [Homo sapiens] | 103.66 | 51.02% | 10 | 2 | 2 | 11 | 49 | 5.4 | 8.21 | 1.258 |
| 194376318 | unnamed protein product [Homo sapiens] | 29.81 | 3.70% | 3 | 1 | 1 | 1 | 243 | 27 | 9.61 | 1.27 |
| 111917949 | immunoglobulin heavy chain variable region [Homo sapiens] | 76.7 | 18.18% | 77 | 1 | 2 | 8 | 154 | 16.7 | 7.84 | 1.278 |
| 21669819 | immunoglobulin heavy chain VHDJ region [Homo sapiens] | 90.22 | 25.00% | 417 | 1 | 2 | 7 | 120 | 13.1 | 5.85 | 1.284 |
| 156765940 | collectin kidney K1 [Homo sapiens] | 260.61 | 27.41% | 26 | 7 | 7 | 33 | 197 | 21.6 | 4.75 | 1.294 |
| 13661193 | fibulin-1 isoform D precursor [Homo sapiens] >gi|1621019|gb|AAB17099.1| fibulin-1D [Homo sapiens] | 158.68 | 9.25% | 23 | 5 | 5 | 24 | 703 | 77.2 | 5.26 | 1.295 |
| 4467842 | immunoglobulin M heavy chain [Homo sapiens] | 1329.03 | 66.45% | 17 | 1 | 25 | 1256 | 453 | 49.4 | 6.77 | 1.301 |
| 194385976 | unnamed protein product [Homo sapiens] | 42.78 | 8.86% | 5 | 1 | 1 | 3 | 158 | 17.6 | 7.15 | 1.302 |
| 17226634 | immunoglobulin heavy chain variable region [Homo sapiens] | 99.44 | 24.39% | 225 | 1 | 3 | 8 | 123 | 13.4 | 7.83 | 1.302 |
| 1369904 | serum lectin P35 [Homo sapiens] >gi|1669349|dbj|BAA09636.1| lectin P35 [Homo sapiens] | 342.33 | 30.99% | 6 | 8 | 9 | 95 | 313 | 34 | 6.54 | 1.31 |
| 114147277 | immunoglobulin heavy chain variable region [Homo sapiens] | 74.17 | 22.73% | 41 | 1 | 2 | 12 | 110 | 12.3 | 8.29 | 1.311 |
| 4557871 | serotransferrin precursor [Homo sapiens] >gi|136191|sp|P02787.2|TRFE_HUMAN RecName: Full=Serotransferrin; Short=Transferrin; AltName: Full=Siderophilin; AltName: Full=Beta-1 metal-binding globulin; Flags: Precursor >gi|248648|gb|AAB22049.1| transferrin [Homo sapiens] >gi|339453|gb|AAA61140.1| transferrin precursor [Homo sapiens] >gi|15021381|gb|AAK77664.1| transferin [Homo sapiens] >gi|31415705|gb|AAP45055.1| transferrin [Homo sapiens] >gi|94717618|gb|ABF47110.1| transferrin [Homo sapiens] >gi|119599573|gb|EAW79167.1| transferrin, isoform CRA_d [Homo sapiens] | 635.11 | 29.94% | 18 | 15 | 15 | 60 | 698 | 77 | 7.12 | 1.312 |
| 189217853 | 72 kDa type IV collagenase isoform b [Homo sapiens] | 158.27 | 16.56% | 11 | 6 | 6 | 9 | 610 | 68.8 | 5.34 | 1.316 |
| 18041890 | immunoglobulin lambda light chain variable region [Homo sapiens] | 35.59 | 9.09% | 3 | 1 | 1 | 7 | 88 | 9.2 | 5.24 | 1.326 |
| 221044282 | unnamed protein product [Homo sapiens] | 229.04 | 9.77% | 4 | 5 | 5 | 15 | 870 | 95.9 | 4.64 | 1.334 |
| 21628665 | immunoglobulin light chain constant region [Homo sapiens] | 116.72 | 91.43% | 1 | 1 | 3 | 82 | 35 | 3.9 | 6.48 | 1.35 |
| 12054080 | immunoglobulin heavy chain constant region mu [Homo sapiens] | 1191.77 | 62.03% | 8 | 1 | 22 | 1031 | 453 | 49.4 | 6.83 | 1.353 |
| 21518719 | hyaluronidase 1 variant 5 [Homo sapiens] >gi|119585494|gb|EAW65090.1| hyaluronoglucosaminidase 1, isoform CRA_f [Homo sapiens] | 46.2 | 25.00% | 12 | 1 | 1 | 2 | 96 | 10.4 | 8.53 | 1.362 |
| 37777898 | immunoglobulin heavy chain variable region [Homo sapiens] | 196.03 | 32.03% | 213 | 1 | 4 | 61 | 153 | 16.5 | 8.81 | 1.368 |
| 114665902 | PREDICTED: similar to Profilin-1 (Profilin I) [Pan troglodytes] >gi|119610788|gb|EAW90382.1| profilin 1, isoform CRA_b [Homo sapiens] | 41.59 | 7.69% | 2 | 1 | 1 | 1 | 104 | 11.4 | 9.17 | 1.37 |
| 219566101 | immunoglobulin heavy chain [Homo sapiens] | 75.42 | 22.39% | 9 | 1 | 2 | 3 | 134 | 14.7 | 5.48 | 1.383 |
| 87299014 | immunoglobulin light chain variable region [Homo sapiens] | 454.36 | 71.05% | 348 | 1 | 9 | 514 | 152 | 16.1 | 4.93 | 1.383 |
| 219566389 | immunoglobulin kappa light chain [Homo sapiens] | 132.14 | 39.29% | 540 | 1 | 3 | 31 | 112 | 12.4 | 8.94 | 1.393 |
| 21668722 | immunoglobulin heavy chain VHDJ region [Homo sapiens] | 99.89 | 18.11% | 424 | 1 | 2 | 8 | 127 | 13.7 | 8.85 | 1.399 |
| 55957679 | cartilage acidic protein 1 [Homo sapiens] >gi|55958790|emb|CAI14275.1| cartilage acidic protein 1 [Homo sapiens] | 68.51 | 5.34% | 10 | 3 | 3 | 5 | 524 | 56.5 | 5.55 | 1.401 |
| 27369048 | immunoglobulin lambda light chain variable region [Homo sapiens] | 10.42 | 9.29% | 1 | 1 | 1 | 1 | 140 | 14.5 | 7.06 | 1.403 |
| 194383694 | unnamed protein product [Homo sapiens] | 112.48 | 6.53% | 10 | 3 | 3 | 10 | 704 | 77.2 | 4.53 | 1.414 |
| 3004786 | Ig heavy chain variable region [Homo sapiens] | 109.28 | 29.66% | 145 | 1 | 3 | 19 | 118 | 13.1 | 7.97 | 1.418 |
| 4321593 | immunoglobulin G kappa chain [Homo sapiens] | 361.42 | 38.25% | 71 | 1 | 5 | 126 | 217 | 24 | 6.55 | 1.427 |
| 119573006 | apolipoprotein A-II, isoform CRA_c [Homo sapiens] | 399.98 | 67.50% | 1 | 1 | 8 | 484 | 80 | 9 | 6.57 | 1.43 |
| 33319680 | Ig heavy chain variable region, VH3 family [Homo sapiens] | 172.21 | 39.34% | 1007 | 1 | 4 | 29 | 122 | 13.1 | 8.85 | 1.434 |
| 681900 | This CDS feature is included to show the translation of the corresponding V_region. Presently translation qualifiers on V_region features are illegal [Homo sapiens] | 138.54 | 44.86% | 293 | 1 | 3 | 9 | 107 | 11.6 | 8.51 | 1.449 |
| 21669521 | immunoglobulin lambda light chain VLJ region [Homo sapiens] | 493.89 | 40.52% | 420 | 1 | 11 | 747 | 269 | 27.9 | 7.91 | 1.45 |
| 157778872 | immunoglobulin heavy chain variable region [Homo sapiens] | 45.74 | 17.39% | 231 | 1 | 2 | 7 | 115 | 12.8 | 9.01 | 1.452 |
| 21669509 | immunoglobulin lambda light chain VLJ region [Homo sapiens] | 302.26 | 34.43% | 250 | 1 | 7 | 501 | 273 | 28.2 | 7.14 | 1.51 |
| 1864143 | Ig lambda chain V-region [Homo sapiens] | 141.47 | 32.37% | 317 | 1 | 4 | 232 | 139 | 14.4 | 8.43 | 1.54 |
| 33319656 | Ig heavy chain variable region, VH3 family [Homo sapiens] | 122.03 | 30.83% | 763 | 1 | 3 | 26 | 120 | 13.1 | 7.14 | 1.547 |
| 553490 | immunoglobulin lambda-chain [Homo sapiens] | 64.73 | 12.50% | 89 | 1 | 2 | 7 | 128 | 13.4 | 7.81 | 1.55 |
| 109240688 | immunoglobulin kappa chain variable region [Homo sapiens] | 41.25 | 15.09% | 2 | 1 | 1 | 3 | 106 | 11.7 | 7.99 | 1.558 |
| 15886930 | immunoglobulin heavy chain variable region [Homo sapiens] | 122.46 | 46.61% | 493 | 1 | 4 | 11 | 118 | 12.9 | 8.41 | 1.573 |
| 34534492 | unnamed protein product [Homo sapiens] | 13.39 | 8.66% | 1 | 1 | 1 | 1 | 277 | 30.5 | 8.56 | 1.608 |
| 194390508 | unnamed protein product [Homo sapiens] | 1308.75 | 40.93% | 24 | 1 | 25 | 226 | 1014 | 111.2 | 6.21 | 1.67 |
| 11122875 | glycosylphosphatidylinositol phospholipase D [Homo sapiens] | 137.2 | 8.32% | 8 | 3 | 3 | 4 | 457 | 48.5 | 6.2 | 1.685 |
| 20377087 | intestinal lactoferrin receptor [Homo sapiens] >gi|7019846|dbj|BAA90893.1| unnamed protein product [Homo sapiens] >gi|8096221|dbj|BAA96094.1| intelectin [Homo sapiens] >gi|18091783|gb|AAL58073.1| endothelial lectin HL-1 [Homo sapiens] >gi|37181843|gb|AAQ88725.1| ITLN [Homo sapiens] >gi|37724012|gb|AAO17801.1| intelectin [Homo sapiens] >gi|44976129|gb|AAS49907.1| omentin [Homo sapiens] >gi|48146565|emb|CAG33505.1| ITLN1 [Homo sapiens] >gi|52843233|gb|AAU88047.1| intelectin 1 [Homo sapiens] >gi|62896625|dbj|BAD96253.1| intelectin variant [Homo sapiens] | 171.75 | 29.07% | 7 | 7 | 7 | 13 | 313 | 35 | 5.82 | 1.694 |
| 77379428 | immunoglobulin kappa chain variable region [Homo sapiens] | 38.02 | 21.49% | 1 | 1 | 1 | 2 | 121 | 13.1 | 6 | 1.707 |
| 18307322 | immunoglobulin light chain lambda variable region [Homo sapiens] | 27.87 | 12.62% | 1 | 1 | 1 | 1 | 103 | 10.8 | 5.25 | 1.716 |
| 119600101 | phosphatidylinositol-specific phospholipase C, X domain containing 2 [Homo sapiens] | 34.44 | 2.42% | 3 | 1 | 1 | 1 | 289 | 33 | 9.44 | 1.725 |
| 27373753 | apolipoprotein J [Homo sapiens] | 220.42 | 55.26% | 1 | 1 | 5 | 87 | 76 | 9.1 | 8.15 | 1.815 |
| 1620396 | haptoglobin [Homo sapiens] | 370.62 | 34.77% | 7 | 4 | 11 | 42 | 348 | 39 | 6.89 | 1.876 |
| 37694587 | immunoglobulin heavy chain variable region [Homo sapiens] | 81.8 | 25.64% | 12 | 1 | 2 | 5 | 117 | 12.9 | 7.39 | 1.895 |
| 58222449 | anti-tetanus toxoid immunoglobulin heavy chain variable region [Homo sapiens] | 102.65 | 21.49% | 133 | 1 | 3 | 20 | 121 | 13.2 | 8.46 | 2.035 |
| 58222839 | anti-tetanus toxoid immunoglobulin light chain variable region [Homo sapiens] | 60.6 | 30.84% | 125 | 1 | 2 | 9 | 107 | 11.7 | 6.54 | 2.137 |
| 587350 | immunoglobulin kappa light chain variable region [Homo sapiens] | 67.14 | 29.91% | 10 | 1 | 3 | 5 | 117 | 12.6 | 7.12 | 2.203 |
| 247425006 | immunoglobulin heavy chain variable region [Homo sapiens] | 179.48 | 39.34% | 421 | 1 | 4 | 50 | 122 | 13.1 | 8.81 | 2.216 |
| 94469923 | anti-West Nile virus immunoglobulin light chain variable region [Homo sapiens] | 38.87 | 21.82% | 1 | 1 | 1 | 1 | 110 | 12 | 7.96 | 2.234 |
| 21669285 | immunoglobulin lambda light chain VLJ region [Homo sapiens] | 43.55 | 32.43% | 4 | 1 | 2 | 3 | 111 | 11.7 | 8.91 | 2.238 |
| 8777875 | immunoglobulin light chain variable region [Homo sapiens] | 174.72 | 37.61% | 423 | 1 | 3 | 26 | 109 | 12 | 8.48 | 2.253 |
| 18092610 | anti-cardiolipin immunoglobulin light chain [Homo sapiens] | 115.07 | 26.85% | 23 | 1 | 2 | 27 | 108 | 12 | 8.63 | 2.289 |
| 247424903 | immunoglobulin heavy chain variable region [Homo sapiens] | 286.5 | 51.22% | 1252 | 1 | 5 | 69 | 123 | 13.4 | 7.96 | 2.462 |
| 221042228 | unnamed protein product [Homo sapiens] | 15.94 | 2.78% | 1 | 1 | 1 | 1 | 539 | 61.3 | 7.74 | 2.86 |
| 346196 | Ig lambda chain V region - human >gi|452933|gb|AAB28790.1| anti-cardiolipin/beta 2 glycoprotein I immunoglobulin light chain variable region [Homo sapiens] | 102.42 | 21.67% | 284 | 1 | 3 | 22 | 120 | 12.4 | 6.48 | 2.959 |
| 47271320 | immunoglobulin lambda light chain variable region [Homo sapiens] | 59.98 | 21.05% | 104 | 1 | 2 | 5 | 114 | 12.1 | 5.01 | 3.343 |
| 194273292 | immunoglobulin heavy chain variable region [Homo sapiens] | 227.62 | 70.30% | 896 | 1 | 6 | 13 | 101 | 11 | 9.72 | 3.478 |
| 33319586 | Ig heavy chain variable region, VH3 family [Homo sapiens] | 125.55 | 25.00% | 742 | 1 | 2 | 23 | 120 | 13 | 4.77 | 4.879 |
| 226958414 | isovaleryl-CoA dehydrogenase, mitochondrial isoform 2 precursor [Homo sapiens] >gi|119612820|gb|EAW92414.1| isovaleryl Coenzyme A dehydrogenase, isoform CRA_b [Homo sapiens] >gi|193783817|dbj|BAG53799.1| unnamed protein product [Homo sapiens] | 25.55 | 2.27% | 4 | 1 | 1 | 1 | 396 | 43 | 7.66 | 5.884 |
| 2253348 | immunoglobulin heavy chain variable region [Homo sapiens] | 69.15 | 22.13% | 31 | 1 | 2 | 3 | 122 | 13.4 | 9.07 | 8.591 |
| 57162363 | KIAA0515 [Homo sapiens] | 20.36 | 1.53% | 8 | 1 | 1 | 1 | 587 | 63.2 | 6.73 | 11.119 |

**VSD vs. control**

| Coverage | # Proteins | # Unique Peptides | # Peptides | # PSMs | # AAs | MW [kDa] | calc. pI | VSDdanchun/  control |
| --- | --- | --- | --- | --- | --- | --- | --- | --- |
| 24.00% | 13 | 2 | 2 | 25 | 100 | 10.7 | 7.72 | 0.147 |
| 3.73% | 10 | 1 | 1 | 2 | 241 | 27.1 | 6.93 | 0.201 |
| 71.43% | 76 | 7 | 8 | 59 | 147 | 16 | 7.69 | 0.241 |
| 3.58% | 6 | 1 | 1 | 5 | 531 | 58.1 | 8.72 | 0.244 |
| 9.80% | 6 | 1 | 1 | 1 | 102 | 11.4 | 11.36 | 0.252 |
| 9.84% | 5 | 2 | 2 | 3 | 183 | 20.1 | 8.78 | 0.253 |
| 34.65% | 1 | 1 | 3 | 22 | 101 | 10.8 | 8.62 | 0.348 |
| 22.77% | 15 | 1 | 2 | 21 | 101 | 11 | 6.68 | 0.393 |
| 46.79% | 107 | 1 | 3 | 5 | 109 | 11.8 | 9.29 | 0.407 |
| 9.06% | 11 | 8 | 8 | 18 | 1170 | 129.3 | 4.94 | 0.43 |
| 52.10% | 10 | 2 | 5 | 45 | 119 | 13.1 | 8.18 | 0.452 |
| 75.00% | 4 | 2 | 9 | 695 | 92 | 10.6 | 9.04 | 0.518 |
| 58.86% | 3 | 1 | 22 | 75 | 474 | 52.9 | 5.45 | 0.529 |
| 19.84% | 4 | 2 | 2 | 3 | 126 | 13.7 | 9 | 0.553 |
| 15.07% | 15 | 4 | 4 | 12 | 564 | 60 | 5.74 | 0.561 |
| 54.10% | 6 | 2 | 5 | 58 | 122 | 13.5 | 6.32 | 0.566 |
| 58.44% | 1 | 1 | 22 | 73 | 474 | 52.9 | 5.54 | 0.568 |
| 34.43% | 250 | 1 | 7 | 501 | 273 | 28.2 | 7.14 | 0.585 |
| 16.10% | 7 | 1 | 1 | 1 | 118 | 12.9 | 7.2 | 0.589 |
| 38.02% | 272 | 1 | 3 | 19 | 121 | 13.2 | 8.43 | 0.594 |
| 5.10% | 7 | 1 | 2 | 2 | 314 | 33.8 | 6.64 | 0.601 |
| 5.23% | 12 | 1 | 1 | 1 | 306 | 34 | 5.78 | 0.605 |
| 31.28% | 3 | 3 | 6 | 38 | 243 | 27.9 | 6.92 | 0.612 |
| 23.91% | 3 | 1 | 1 | 2 | 46 | 5.3 | 4.46 | 0.618 |
| 1.89% | 3 | 1 | 1 | 3 | 423 | 48.4 | 7.71 | 0.618 |
| 24.19% | 27 | 7 | 7 | 15 | 434 | 47.1 | 7.69 | 0.624 |
| 32.98% | 2 | 1 | 1 | 2 | 94 | 10.2 | 6.99 | 0.625 |
| 9.64% | 4 | 1 | 1 | 1 | 83 | 8.9 | 7.12 | 0.626 |
| 54.10% | 9 | 3 | 6 | 71 | 122 | 13.6 | 6.79 | 0.628 |
| 3.42% | 8 | 1 | 1 | 1 | 234 | 25.5 | 5.06 | 0.63 |
| 32.29% | 193 | 1 | 2 | 2 | 96 | 10.3 | 7.97 | 0.632 |
| 28.74% | 208 | 1 | 3 | 50 | 167 | 18 | 8.9 | 0.635 |
| 7.30% | 5 | 1 | 1 | 3 | 274 | 30.2 | 7.15 | 0.65 |
| 40.93% | 24 | 1 | 25 | 226 | 1014 | 111.2 | 6.21 | 0.658 |
| 18.55% | 423 | 1 | 2 | 11 | 124 | 13.5 | 9.36 | 0.658 |
| 18.97% | 627 | 1 | 2 | 22 | 116 | 12.9 | 7.85 | 0.667 |
| 79.10% | 83 | 1 | 9 | 252 | 67 | 7.4 | 6.95 | 0.667 |
| 26.17% | 419 | 1 | 2 | 26 | 107 | 11.7 | 7.97 | 0.671 |
| 17.44% | 7 | 1 | 1 | 3 | 86 | 9.2 | 9.72 | 0.675 |
| 40.88% | 6 | 4 | 4 | 5 | 137 | 15.6 | 8.35 | 0.686 |
| 62.53% | 5 | 6 | 27 | 1576 | 419 | 47.4 | 5.95 | 0.688 |
| 46.99% | 2 | 1 | 11 | 318 | 366 | 39.2 | 5.72 | 0.697 |
| 43.77% | 309 | 1 | 9 | 529 | 265 | 28.5 | 7.97 | 0.698 |
| 17.65% | 2 | 1 | 1 | 4 | 102 | 10.9 | 7.12 | 0.703 |
| 17.88% | 10 | 1 | 1 | 4 | 151 | 16.8 | 4.79 | 0.705 |
| 0.89% | 9 | 1 | 1 | 1 | 1014 | 113.4 | 9.03 | 0.709 |
| 48.08% | 1 | 2 | 4 | 9 | 104 | 11.5 | 9.1 | 0.715 |
| 6.34% | 4 | 1 | 1 | 1 | 363 | 40.9 | 6.43 | 0.72 |
| 0.69% | 1 | 1 | 1 | 1 | 873 | 98.1 | 9.29 | 0.72 |
| 7.43% | 12 | 2 | 2 | 7 | 269 | 30.7 | 7.78 | 0.724 |
| 17.28% | 9 | 12 | 12 | 53 | 1065 | 122.1 | 5.74 | 0.732 |
| 6.06% | 1 | 1 | 1 | 2 | 132 | 13.7 | 8.94 | 0.735 |
| 33.33% | 58 | 8 | 8 | 22 | 333 | 37.3 | 5.71 | 0.738 |
| 16.00% | 4 | 8 | 8 | 42 | 525 | 59.5 | 7.44 | 0.742 |
| 4.09% | 12 | 1 | 1 | 1 | 513 | 55.7 | 8.94 | 0.742 |
| 32.32% | 36 | 1 | 2 | 3 | 99 | 10.6 | 6.51 | 0.742 |
| 37.42% | 82 | 36 | 60 | 502 | 2330 | 256.3 | 5.8 | 0.746 |
| 30.61% | 136 | 1 | 2 | 41 | 98 | 10.8 | 7.08 | 0.747 |
| 2.55% | 1 | 1 | 1 | 4 | 275 | 32.3 | 8.37 | 0.748 |
| 64.29% | 3758 | 1 | 10 | 90 | 238 | 25 | 8.41 | 0.751 |
| 52.17% | 497 | 1 | 3 | 31 | 92 | 10.1 | 5.94 | 0.754 |
| 52.99% | 692 | 1 | 4 | 31 | 134 | 14.5 | 7.96 | 0.754 |
| 43.52% | 461 | 1 | 3 | 17 | 108 | 11.3 | 7.96 | 0.754 |
| 37.58% | 3 | 1 | 12 | 147 | 330 | 37.6 | 7.39 | 0.758 |
| 8.11% | 5 | 3 | 3 | 7 | 444 | 50.7 | 8.28 | 0.761 |
| 25.93% | 193 | 1 | 2 | 2 | 108 | 11.5 | 5.96 | 0.761 |
| 67.61% | 7 | 24 | 34 | 2036 | 457 | 52.1 | 8.07 | 0.764 |
| 53.04% | 3 | 4 | 14 | 328 | 247 | 27.7 | 7.99 | 0.766 |
| 18.68% | 2 | 3 | 3 | 9 | 273 | 30.7 | 6.84 | 0.77 |
| 14.93% | 2 | 1 | 1 | 1 | 134 | 14.5 | 7.34 | 0.772 |
| 25.00% | 17 | 1 | 2 | 6 | 108 | 11.4 | 4.92 | 0.772 |
| 58.15% | 7 | 76 | 76 | 444 | 1663 | 187 | 6.4 | 0.775 |
| 68.56% | 25 | 2 | 28 | 1123 | 334 | 37.7 | 6.29 | 0.775 |
| 17.34% | 21 | 1 | 59 | 189 | 4563 | 515.1 | 7.11 | 0.779 |
| 47.85% | 7 | 1 | 21 | 248 | 650 | 72 | 6.49 | 0.781 |
| 30.07% | 5 | 3 | 3 | 7 | 143 | 16.1 | 6.3 | 0.788 |
| 3.14% | 5 | 2 | 2 | 5 | 764 | 85.5 | 6.96 | 0.79 |
| 22.22% | 418 | 1 | 2 | 25 | 108 | 12 | 8.81 | 0.796 |
| 28.68% | 184 | 1 | 3 | 7 | 129 | 14 | 8.29 | 0.798 |
| 8.91% | 34 | 1 | 1 | 6 | 101 | 11.3 | 10.14 | 0.8 |
| 93.57% | 87 | 32 | 40 | 1407 | 249 | 28.9 | 5.6 | 0.8 |
| 2.56% | 13 | 5 | 5 | 12 | 2224 | 251.5 | 6.05 | 0.809 |
| 3.11% | 7 | 1 | 1 | 2 | 322 | 37.2 | 7.99 | 0.809 |
| 7.67% | 2 | 2 | 2 | 3 | 352 | 38.2 | 7.11 | 0.809 |
| 39.42% | 15 | 5 | 12 | 56 | 345 | 38.2 | 6.6 | 0.812 |
| 23.68% | 4 | 1 | 2 | 10 | 114 | 12.5 | 5.36 | 0.815 |
| 58.07% | 12 | 45 | 45 | 2395 | 644 | 69.7 | 8.06 | 0.824 |
| 21.37% | 5 | 6 | 6 | 13 | 248 | 26.1 | 5.49 | 0.824 |
| 7.69% | 4 | 1 | 1 | 3 | 312 | 34 | 5.03 | 0.825 |
| 27.27% | 244 | 1 | 2 | 16 | 121 | 12.7 | 5.31 | 0.825 |
| 1.31% | 4 | 1 | 1 | 1 | 609 | 68.9 | 6.77 | 0.825 |
| 26.61% | 3 | 1 | 6 | 71 | 327 | 36.1 | 7.34 | 0.827 |
| 22.22% | 231 | 1 | 2 | 7 | 117 | 12.5 | 8.68 | 0.827 |
| 27.27% | 59 | 1 | 2 | 27 | 132 | 14 | 8.46 | 0.83 |
| 39.29% | 540 | 1 | 3 | 31 | 112 | 12.4 | 8.94 | 1.201 |
| 36.89% | 113 | 1 | 2 | 7 | 122 | 12.6 | 8.5 | 1.207 |
| 9.69% | 10 | 3 | 3 | 6 | 423 | 47.6 | 6.11 | 1.208 |
| 30.30% | 16 | 1 | 2 | 2 | 99 | 10.8 | 8.48 | 1.208 |
| 4.23% | 18 | 2 | 2 | 2 | 780 | 82.5 | 6.55 | 1.212 |
| 76.35% | 36 | 24 | 45 | 799 | 609 | 69.3 | 6.28 | 1.213 |
| 28.45% | 838 | 1 | 3 | 26 | 116 | 12.8 | 7.97 | 1.213 |
| 62.03% | 8 | 1 | 22 | 1031 | 453 | 49.4 | 6.83 | 1.224 |
| 34.26% | 93 | 2 | 3 | 34 | 108 | 11.7 | 8.47 | 1.224 |
| 58.36% | 9 | 20 | 20 | 272 | 317 | 36.1 | 5.73 | 1.231 |
| 82.93% | 692 | 1 | 4 | 33 | 82 | 9 | 7.96 | 1.235 |
| 45.70% | 15 | 1 | 10 | 71 | 337 | 37.8 | 5.08 | 1.235 |
| 37.78% | 190 | 1 | 2 | 14 | 90 | 9.7 | 7.96 | 1.241 |
| 7.46% | 9 | 1 | 1 | 3 | 362 | 41.6 | 5.01 | 1.252 |
| 29.67% | 735 | 1 | 5 | 64 | 209 | 22.6 | 7.68 | 1.253 |
| 82.41% | 704 | 1 | 5 | 33 | 108 | 11.5 | 7.96 | 1.259 |
| 25.24% | 97 | 1 | 2 | 2 | 103 | 11.1 | 8.88 | 1.262 |
| 66.67% | 11 | 7 | 7 | 86 | 117 | 12.8 | 5.45 | 1.264 |
| 33.33% | 15 | 1 | 1 | 1 | 24 | 2.7 | 4.23 | 1.266 |
| 55.26% | 1 | 1 | 5 | 87 | 76 | 9.1 | 8.15 | 1.267 |
| 45.26% | 3 | 3 | 3 | 21 | 137 | 15.6 | 4.73 | 1.272 |
| 33.87% | 80 | 2 | 5 | 14 | 124 | 12.9 | 8.5 | 1.272 |
| 5.34% | 10 | 3 | 3 | 5 | 524 | 56.5 | 5.55 | 1.28 |
| 8.00% | 7 | 3 | 3 | 4 | 425 | 47 | 6.01 | 1.282 |
| 10.06% | 7 | 5 | 11 | 39 | 1481 | 163.6 | 6.42 | 1.284 |
| 32.43% | 4 | 1 | 2 | 3 | 111 | 11.7 | 8.91 | 1.294 |
| 27.66% | 13 | 1 | 3 | 214 | 141 | 15.1 | 5.45 | 1.295 |
| 78.47% | 5 | 1 | 22 | 318 | 288 | 32.6 | 6.54 | 1.299 |
| 29.94% | 18 | 15 | 15 | 60 | 698 | 77 | 7.12 | 1.306 |
| 48.37% | 58 | 1 | 8 | 578 | 215 | 23.4 | 6.54 | 1.333 |
| 6.53% | 10 | 3 | 3 | 10 | 704 | 77.2 | 4.53 | 1.354 |
| 7.69% | 2 | 1 | 1 | 1 | 104 | 11.4 | 9.17 | 1.357 |
| 29.77% | 418 | 1 | 2 | 25 | 131 | 14.2 | 6.51 | 1.359 |
| 9.77% | 4 | 5 | 5 | 15 | 870 | 95.9 | 4.64 | 1.378 |
| 29.07% | 7 | 7 | 7 | 13 | 313 | 35 | 5.82 | 1.393 |
| 9.09% | 3 | 1 | 1 | 7 | 88 | 9.2 | 5.24 | 1.406 |
| 21.05% | 104 | 1 | 2 | 5 | 114 | 12.1 | 5.01 | 1.411 |
| 3.61% | 2 | 1 | 1 | 1 | 527 | 59.7 | 7.24 | 1.412 |
| 91.43% | 1 | 1 | 3 | 82 | 35 | 3.9 | 6.48 | 1.415 |
| 20.64% | 2 | 6 | 6 | 8 | 470 | 51.7 | 8.81 | 1.432 |
| 39.34% | 1007 | 1 | 4 | 29 | 122 | 13.1 | 8.85 | 1.435 |
| 36.84% | 16 | 1 | 3 | 3 | 114 | 12.3 | 8.94 | 1.438 |
| 18.18% | 77 | 1 | 2 | 8 | 154 | 16.7 | 7.84 | 1.444 |
| 7.71% | 7 | 3 | 3 | 4 | 584 | 65.1 | 6.47 | 1.445 |
| 39.63% | 106 | 1 | 10 | 568 | 270 | 28.1 | 7.14 | 1.454 |
| 31.48% | 464 | 1 | 2 | 17 | 108 | 11.7 | 9.17 | 1.467 |
| 14.58% | 39 | 1 | 3 | 36 | 144 | 15.6 | 9.13 | 1.481 |
| 21.88% | 47 | 1 | 2 | 18 | 128 | 14.4 | 5.1 | 1.501 |
| 26.13% | 23 | 1 | 2 | 26 | 111 | 12.3 | 8.85 | 1.508 |
| 18.75% | 2 | 1 | 1 | 4 | 96 | 10.4 | 8.5 | 1.532 |
| 8.66% | 1 | 1 | 1 | 1 | 277 | 30.5 | 8.56 | 1.538 |
| 25.00% | 417 | 1 | 2 | 7 | 120 | 13.1 | 5.85 | 1.564 |
| 29.66% | 145 | 1 | 3 | 19 | 118 | 13.1 | 7.97 | 1.581 |
| 4.80% | 8 | 3 | 3 | 4 | 1041 | 112.4 | 7.31 | 1.585 |
| 37.72% | 120 | 1 | 4 | 9 | 114 | 12.1 | 8.02 | 1.586 |
| 44.86% | 293 | 1 | 3 | 9 | 107 | 11.6 | 8.51 | 1.597 |
| 32.03% | 213 | 1 | 4 | 61 | 153 | 16.5 | 8.81 | 1.635 |
| 2.42% | 3 | 1 | 1 | 1 | 289 | 33 | 9.44 | 1.728 |
| 51.22% | 1252 | 1 | 5 | 69 | 123 | 13.4 | 7.96 | 1.762 |
| 30.83% | 763 | 1 | 3 | 26 | 120 | 13.1 | 7.14 | 1.766 |
| 9.09% | 4 | 1 | 1 | 3 | 88 | 9.5 | 5.17 | 1.809 |
| 12.62% | 1 | 1 | 1 | 1 | 103 | 10.8 | 5.25 | 1.878 |
| 70.30% | 896 | 1 | 6 | 13 | 101 | 11 | 9.72 | 1.89 |
| 24.59% | 743 | 1 | 2 | 24 | 122 | 13.1 | 6.57 | 1.948 |
| 26.85% | 23 | 1 | 2 | 27 | 108 | 12 | 8.63 | 1.962 |
| 53.42% | 425 | 1 | 3 | 25 | 73 | 8 | 8.53 | 2.015 |
| 21.49% | 1 | 1 | 1 | 2 | 121 | 13.1 | 6 | 2.024 |
| 43.65% | 494 | 1 | 4 | 12 | 126 | 14 | 7.91 | 2.036 |
| 37.61% | 423 | 1 | 3 | 26 | 109 | 12 | 8.48 | 2.039 |
| 8.32% | 8 | 3 | 3 | 4 | 457 | 48.5 | 6.2 | 2.066 |
| 25.64% | 12 | 1 | 2 | 5 | 117 | 12.9 | 7.39 | 2.113 |
| 66.45% | 17 | 1 | 25 | 1256 | 453 | 49.4 | 6.77 | 2.115 |
| 21.49% | 133 | 1 | 3 | 20 | 121 | 13.2 | 8.46 | 2.129 |
| 46.61% | 493 | 1 | 4 | 11 | 118 | 12.9 | 8.41 | 2.14 |
| 12.50% | 89 | 1 | 2 | 7 | 128 | 13.4 | 7.81 | 2.171 |
| 34.17% | 151 | 1 | 3 | 5 | 120 | 13.1 | 6.52 | 2.18 |
| 21.82% | 1 | 1 | 1 | 1 | 110 | 12 | 7.96 | 2.255 |
| 15.09% | 2 | 1 | 1 | 3 | 106 | 11.7 | 7.99 | 2.298 |
| 39.32% | 1183 | 1 | 4 | 41 | 117 | 13 | 6.04 | 2.343 |
| 29.91% | 10 | 1 | 3 | 5 | 117 | 12.6 | 7.12 | 2.37 |
| 34.77% | 7 | 4 | 11 | 42 | 348 | 39 | 6.89 | 2.403 |
| 39.34% | 421 | 1 | 4 | 50 | 122 | 13.1 | 8.81 | 2.447 |
| 2.78% | 1 | 1 | 1 | 1 | 539 | 61.3 | 7.74 | 2.462 |
| 25.00% | 742 | 1 | 2 | 23 | 120 | 13 | 4.77 | 2.63 |
| 44.95% | 242 | 1 | 3 | 27 | 109 | 11.7 | 7.28 | 2.68 |
| 21.67% | 284 | 1 | 3 | 22 | 120 | 12.4 | 6.48 | 4.139 |
| 2.27% | 4 | 1 | 1 | 1 | 396 | 43 | 7.66 | 8.047 |
| 1.53% | 8 | 1 | 1 | 1 | 587 | 63.2 | 6.73 | 15.068 |
| 22.13% | 31 | 1 | 2 | 3 | 122 | 13.4 | 9.07 | 31.93 |

**ASD-PH vs. Control**

| Accession | Description | Score | Coverage | # Proteins | # Unique Peptides | # Peptides | # PSMs | # AAs | MW [kDa] | calc. pI | ASD-PH/Control |
| --- | --- | --- | --- | --- | --- | --- | --- | --- | --- | --- | --- |
| 13195586 | hemoglobin alpha 1 globin chain [Homo sapiens] | 103.91 | 24.00% | 13 | 2 | 2 | 25 | 100 | 10.7 | 7.72 | 0.181 |
| 18418633 | mutant beta-globin [Homo sapiens] | 289.03 | 71.43% | 76 | 7 | 8 | 59 | 147 | 16 | 7.69 | 0.231 |
| 183851 | G-gamma-hemoglobin [Homo sapiens] | 64.89 | 22.77% | 15 | 1 | 2 | 21 | 101 | 11 | 6.68 | 0.29 |
| 194375974 | unnamed protein product [Homo sapiens] | 60.43 | 9.84% | 5 | 2 | 2 | 3 | 183 | 20.1 | 8.78 | 0.321 |
| 186083 | immunoglobulin lambda-chain [Homo sapiens] | 82.87 | 21.17% | 350 | 1 | 3 | 29 | 137 | 14.3 | 7.24 | 0.398 |
| 55958543 | heterogeneous nuclear ribonucleoprotein K [Homo sapiens] | 39.26 | 5.23% | 12 | 1 | 1 | 1 | 306 | 34 | 5.78 | 0.416 |
| 13937839 | SAA1 protein [Homo sapiens] >gi|123983058|gb|ABM83270.1| serum amyloid A1 [synthetic construct] >gi|123983248|gb|ABM83365.1| serum amyloid A1 [synthetic construct] >gi|123997747|gb|ABM86475.1| serum amyloid A1 [synthetic construct] >gi|157928044|gb|ABW03318.1| serum amyloid A1 [synthetic construct] | 249.56 | 54.10% | 6 | 2 | 5 | 58 | 122 | 13.5 | 6.32 | 0.47 |
| 56378229 | carbamoylphosphate synthetase I [Homo sapiens] | 22.22 | 4.09% | 12 | 1 | 1 | 1 | 513 | 55.7 | 8.94 | 0.471 |
| 21669937 | immunoglobulin heavy chain VHDJ region [Homo sapiens] | 114.57 | 18.55% | 423 | 1 | 2 | 11 | 124 | 13.5 | 9.36 | 0.471 |
| 3152372 | anti-FactorVIII scFv [Homo sapiens] | 537.51 | 64.29% | 3758 | 1 | 10 | 90 | 238 | 25 | 8.41 | 0.5 |
| 207028494 | L-lactate dehydrogenase A chain isoform 2 [Homo sapiens] >gi|194383812|dbj|BAG59264.1| unnamed protein product [Homo sapiens] | 36.64 | 7.30% | 5 | 1 | 1 | 3 | 274 | 30.2 | 7.15 | 0.549 |
| 46254055 | immunoglobulin heavy chain [Homo sapiens] | 151.53 | 28.74% | 208 | 1 | 3 | 50 | 167 | 18 | 8.9 | 0.567 |
| 36321 | SAA precursor polypeptide (119 AA) [Homo sapiens] | 245.07 | 52.10% | 10 | 2 | 5 | 45 | 119 | 13.1 | 8.18 | 0.569 |
| 587406 | immunoglobulin lambda chain variable region [Homo sapiens] | 78.84 | 27.27% | 244 | 1 | 2 | 16 | 121 | 12.7 | 5.31 | 0.577 |
| 112700542 | immunoglobulin heavy chain variable region [Homo sapiens] | 154.72 | 43.56% | 1441 | 1 | 4 | 24 | 101 | 10.9 | 8.29 | 0.58 |
| 146424184 | apolipoprotein C-IV [Homo sapiens] | 73.46 | 15.75% | 3 | 2 | 2 | 5 | 127 | 14.6 | 9.13 | 0.593 |
| 1064908 | complement Factor H-related Protein 2 [Homo sapiens] | 222.48 | 31.28% | 3 | 3 | 6 | 38 | 243 | 27.9 | 6.92 | 0.601 |
| 18041906 | immunoglobulin lambda light chain variable region [Homo sapiens] | 44.07 | 32.29% | 193 | 1 | 2 | 2 | 96 | 10.3 | 7.97 | 0.611 |
| 10835095 | serum amyloid A-4 protein precursor [Homo sapiens] >gi|259352|gb|AAB24060.1| serum amyloid A [Homo sapiens] >gi|337750|gb|AAA60298.1| serum amyloid A protein [Homo sapiens] >gi|13937846|gb|AAH07026.1| Serum amyloid A4, constitutive [Homo sapiens] >gi|49456475|emb|CAG46558.1| SAA4 [Homo sapiens] >gi|119588821|gb|EAW68415.1| serum amyloid A4, constitutive [Homo sapiens] | 94.08 | 23.08% | 2 | 3 | 3 | 18 | 130 | 14.8 | 9.23 | 0.614 |
| 34532317 | unnamed protein product [Homo sapiens] | 19.57 | 0.89% | 9 | 1 | 1 | 1 | 1014 | 113.4 | 9.03 | 0.629 |
| 76252669 | immunoglobulin lambda light chain variable region [Homo sapiens] | 33.83 | 17.65% | 2 | 1 | 1 | 4 | 102 | 10.9 | 7.12 | 0.633 |
| 51103589 | immunoglobulin variable region VL lambda domain [Homo sapiens] | 92.76 | 25.00% | 17 | 1 | 2 | 6 | 108 | 11.4 | 4.92 | 0.635 |
| 169672524 | transforming growth factor beta 1 precursor [Homo sapiens] | 51.7 | 16.10% | 7 | 1 | 1 | 1 | 118 | 12.9 | 7.2 | 0.638 |
| 2809025 | Ig heavy chain variable region [Homo sapiens] | 41.55 | 42.25% | 134 | 1 | 2 | 4 | 71 | 7.9 | 9.31 | 0.641 |
| 31873302 | hypothetical protein [Homo sapiens] >gi|117646030|emb|CAL38482.1| hypothetical protein [synthetic construct] | 241.85 | 24.19% | 27 | 7 | 7 | 15 | 434 | 47.1 | 7.69 | 0.653 |
| 54304028 | glyceraldehyde-3-phosphate dehydrogenase [Homo sapiens] | 45.44 | 17.44% | 7 | 1 | 1 | 3 | 86 | 9.2 | 9.72 | 0.657 |
| 4505733 | platelet factor 4 precursor [Homo sapiens] >gi|130304|sp|P02776.2|PLF4_HUMAN RecName: Full=Platelet factor 4; Short=PF-4; AltName: Full=C-X-C motif chemokine 4; AltName: Full=Oncostatin-A; AltName: Full=Iroplact; Contains: RecName: Full=Platelet factor 4, short form; Flags: Precursor >gi|13549118|gb|AAK29643.1|AF349466_3 platelet factor 4 [Homo sapiens] >gi|189851|gb|AAA60066.1| platelet factor 4 [Homo sapiens] >gi|47115291|emb|CAG28605.1| PF4 [Homo sapiens] >gi|62739642|gb|AAH93965.1| Platelet factor 4 [Homo sapiens] >gi|63994325|gb|AAY41003.1| unknown [Homo sapiens] >gi|85567544|gb|AAI12094.1| Platelet factor 4 [Homo sapiens] >gi|119626099|gb|EAX05694.1| platelet factor 4 (chemokine (C-X-C motif) ligand 4) [Homo sapiens] >gi|261859268|dbj|BAI46156.1| platelet factor 4 [synthetic construct] | 125.57 | 34.65% | 1 | 1 | 3 | 22 | 101 | 10.8 | 8.62 | 0.657 |
| 30583505 | catenin (cadherin-associated protein), delta 1 [Homo sapiens] | 42.11 | 3.28% | 25 | 1 | 1 | 1 | 610 | 68 | 8.13 | 0.66 |
| 28931 | beta-subunit (AA 1-312) [Homo sapiens] | 29.34 | 7.69% | 4 | 1 | 1 | 3 | 312 | 34 | 5.03 | 0.66 |
| 82734214 | beta-defensin 110 isoform a [Homo sapiens] >gi|84028871|sp|Q30KQ9.1|DB110_HUMAN RecName: Full=Beta-defensin 110; AltName: Full=Defensin, beta 110; AltName: Full=Beta-defensin 10; Short=DEFB-10; AltName: Full=Beta-defensin 111; AltName: Full=Defensin, beta 111; AltName: Full=Beta-defensin 11; Short=DEFB-11; Flags: Precursor >gi|66968904|gb|AAY59751.1| beta-defensin 111 [Homo sapiens] >gi|151555475|gb|AAI48542.1| Defensin, beta 111 [synthetic construct] >gi|162319076|gb|AAI56745.1| Defensin, beta 111 [synthetic construct] | 23.5 | 14.93% | 1 | 1 | 1 | 11 | 67 | 8 | 8.73 | 0.664 |
| 10636616 | immunoglobulin heavy chain variable region [Homo sapiens] | 89.37 | 18.97% | 627 | 1 | 2 | 22 | 116 | 12.9 | 7.85 | 0.664 |
| 247424364 | immunoglobulin heavy chain variable region [Homo sapiens] | 165.61 | 50.81% | 1078 | 1 | 5 | 28 | 124 | 13.4 | 8.91 | 0.664 |
| 112701317 | immunoglobulin heavy chain variable region [Homo sapiens] | 63.94 | 32.32% | 36 | 1 | 2 | 3 | 99 | 10.6 | 6.51 | 0.665 |
| 119573007 | apolipoprotein A-II, isoform CRA_d [Homo sapiens] | 478.54 | 75.00% | 4 | 2 | 9 | 695 | 92 | 10.6 | 9.04 | 0.67 |
| 156616294 | N-acetylmuramoyl-L-alanine amidase precursor [Homo sapiens] >gi|38258222|sp|Q96PD5.1|PGRP2_HUMAN RecName: Full=N-acetylmuramoyl-L-alanine amidase; AltName: Full=Peptidoglycan recognition protein long; Short=PGRP-L; AltName: Full=Peptidoglycan recognition protein 2; Flags: Precursor >gi|15705411|gb|AAL05629.1|AF384856_1 peptidoglycan recognition protein L precursor [Homo sapiens] >gi|119604888|gb|EAW84482.1| peptidoglycan recognition protein 2, isoform CRA_a [Homo sapiens] >gi|158258016|dbj|BAF84981.1| unnamed protein product [Homo sapiens] | 148.7 | 18.92% | 6 | 6 | 6 | 10 | 576 | 62.2 | 7.55 | 0.672 |
| 37789448 | immunoglobulin lambda light chain variable region [Homo sapiens] | 21.04 | 9.64% | 4 | 1 | 1 | 1 | 83 | 8.9 | 7.12 | 0.674 |
| 139641 | RecName: Full=Vitamin D-binding protein; Short=DBP; Short=VDB; AltName: Full=Group-specific component; AltName: Full=Gc-globulin; Flags: Precursor >gi|31676|emb|CAA26938.1| unnamed protein product [Homo sapiens] | 812.8 | 58.44% | 1 | 1 | 22 | 73 | 474 | 52.9 | 5.54 | 0.675 |
| 587273 | Immunoglobulin heavy chain variable region [Homo sapiens] | 96.48 | 27.27% | 59 | 1 | 2 | 27 | 132 | 14 | 8.46 | 0.677 |
| 3337390 | haptoglobin [Homo sapiens] | 399.49 | 39.42% | 15 | 5 | 12 | 56 | 345 | 38.2 | 6.6 | 0.678 |
| 124504316 | HIST2H4B protein [Homo sapiens] | 46.54 | 9.80% | 6 | 1 | 1 | 1 | 102 | 11.4 | 11.36 | 0.686 |
| 221042312 | unnamed protein product [Homo sapiens] | 233.94 | 15.07% | 15 | 4 | 4 | 12 | 564 | 60 | 5.74 | 0.688 |
| 896272 | This CDS feature is included to show the translation of the corresponding V_region. Presently translation qualifiers on V_region features are illegal [Homo sapiens] | 285.25 | 64.57% | 2946 | 2 | 9 | 63 | 127 | 13.8 | 8.79 | 0.69 |
| 4379074 | IgM rheumatoid factor immunoglobulin heavy chain [Homo sapiens] | 104.17 | 39.32% | 775 | 1 | 3 | 23 | 117 | 12.8 | 8.4 | 0.692 |
| 183763 | factor H homologue [Homo sapiens] >gi|158255096|dbj|BAF83519.1| unnamed protein product [Homo sapiens] | 511 | 37.58% | 4 | 1 | 12 | 142 | 330 | 37.6 | 7.56 | 0.708 |
| 158258641 | unnamed protein product [Homo sapiens] | 22.67 | 5.86% | 14 | 1 | 1 | 1 | 222 | 25.7 | 9 | 0.711 |
| 119608546 | ficolin (collagen/fibrinogen domain containing) 1, isoform CRA_d [Homo sapiens] | 57.63 | 5.10% | 7 | 1 | 2 | 2 | 314 | 33.8 | 6.64 | 0.712 |
| 49354849 | immunoglobulin E variable region [Homo sapiens] | 135.35 | 38.02% | 272 | 1 | 3 | 19 | 121 | 13.2 | 8.43 | 0.714 |
| 3169770 | immunoglobulin kappa light chain [Homo sapiens] | 456.88 | 40.28% | 201 | 1 | 8 | 707 | 211 | 23 | 8.05 | 0.722 |
| 5419725 | immunoglobulin light chain variable region [Homo sapiens] | 150.43 | 31.48% | 258 | 1 | 3 | 10 | 108 | 11.5 | 7.12 | 0.722 |
| 189067487 | unnamed protein product [Homo sapiens] | 34.33 | 3.61% | 2 | 1 | 1 | 1 | 527 | 59.7 | 7.24 | 0.723 |
| 1145214 | anti-c-erbB-2 immunoglobulin light chain V region [Homo sapiens] | 113.85 | 41.07% | 298 | 1 | 3 | 24 | 112 | 11.7 | 5.94 | 0.725 |
| 178812 | apolipoprotein B-100 precursor [Homo sapiens] | 1998.27 | 17.34% | 21 | 1 | 59 | 189 | 4563 | 515.1 | 7.11 | 0.733 |
| 189053554 | unnamed protein product [Homo sapiens] | 125.23 | 20.08% | 2 | 3 | 3 | 23 | 244 | 26.4 | 5.74 | 0.735 |
| 119570453 | retinol binding protein 4, plasma, isoform CRA_b [Homo sapiens] | 132.32 | 15.08% | 10 | 3 | 3 | 14 | 199 | 23 | 6.09 | 0.744 |
| 4505735 | platelet factor 4 variant [Homo sapiens] >gi|130306|sp|P10720.1|PF4V_HUMAN RecName: Full=Platelet factor 4 variant; AltName: Full=PF4var1; AltName: Full=PF4alt; AltName: Full=C-X-C motif chemokine 4 variant; AltName: Full=CXCL4L1; Contains: RecName: Full=Platelet factor 4 variant(4-74); Contains: RecName: Full=Platelet factor 4 variant(5-74); Contains: RecName: Full=Platelet factor 4 variant(6-74); Flags: Precursor >gi|292390|gb|AAA60067.1| platelet factor 4 [Homo sapiens] >gi|119626097|gb|EAX05692.1| platelet factor 4 variant 1 [Homo sapiens] >gi|120659980|gb|AAI30654.1| Platelet factor 4 variant 1 [Homo sapiens] >gi|120660120|gb|AAI30658.1| Platelet factor 4 variant 1 [Homo sapiens] | 168.33 | 48.08% | 1 | 2 | 4 | 9 | 104 | 11.5 | 9.1 | 0.753 |
| 119626442 | multimerin 1, isoform CRA_a [Homo sapiens] | 62.55 | 3.58% | 6 | 1 | 1 | 5 | 531 | 58.1 | 8.72 | 0.759 |
| 268374656 | immunoglobulin E heavy chain variable region [Homo sapiens] | 117.96 | 42.57% | 956 | 1 | 3 | 24 | 101 | 11.6 | 6.58 | 0.761 |
| 54779633 | immunoglobulin mu heavy chain [Homo sapiens] | 349.62 | 36.45% | 2295 | 1 | 7 | 185 | 214 | 23 | 7.02 | 0.763 |
| 178834 | apolipoprotein CI [Homo sapiens] | 57.49 | 26.09% | 2 | 2 | 2 | 16 | 69 | 7.7 | 6.73 | 0.763 |
| 110589608 | anti-streptococcal/anti-lysoganglioside immunoglobulin heavy chain variable region [Homo sapiens] | 13.91 | 17.76% | 6 | 1 | 1 | 1 | 107 | 11.5 | 10.2 | 0.764 |
| 105990532 | apolipoprotein B-100 precursor [Homo sapiens] >gi|260158878|gb|ACX32319.1| apolipoprotein B precursor [synthetic construct] | 2158.39 | 18.58% | 36 | 5 | 63 | 209 | 4563 | 515.2 | 7.05 | 0.766 |
| 178741 | apolipoprotein C-II [Homo sapiens] >gi|357629|prf||1303321A apolipoprotein CII | 353.8 | 65.93% | 6 | 6 | 6 | 187 | 91 | 10.2 | 4.56 | 0.77 |
| 14030460 | mannan-binding lectin MBL precursor [Homo sapiens] | 176.08 | 21.37% | 5 | 6 | 6 | 13 | 248 | 26.1 | 5.49 | 0.77 |
| 15637419 | anti-pneumococcal capsular polysaccharide immunoglobulin heavy chain variable region [Homo sapiens] | 121.54 | 30.61% | 136 | 1 | 2 | 41 | 98 | 10.8 | 7.08 | 0.772 |
| 33319096 | Ig heavy chain variable region, VH3 family [Homo sapiens] | 38.9 | 20.97% | 231 | 1 | 2 | 7 | 124 | 13.6 | 7.94 | 0.773 |
| 119588814 | serum amyloid A1, isoform CRA_a [Homo sapiens] >gi|119588815|gb|EAW68409.1| serum amyloid A1, isoform CRA_a [Homo sapiens] >gi|119588817|gb|EAW68411.1| serum amyloid A1, isoform CRA_a [Homo sapiens] | 350.08 | 54.10% | 9 | 3 | 6 | 71 | 122 | 13.6 | 6.79 | 0.773 |
| 58223278 | anti-tetanus toxoid immunoglobulin light chain variable region [Homo sapiens] | 269.33 | 82.41% | 704 | 1 | 5 | 33 | 108 | 11.5 | 7.96 | 0.775 |
| 40795877 | anti-Toxoplasma gondii SAG1 immunoglobulin light chain variable region [Homo sapiens] | 156.94 | 34.26% | 93 | 2 | 3 | 34 | 108 | 11.7 | 8.47 | 0.776 |
| 112910 | RecName: Full=Alpha-2-HS-glycoprotein; AltName: Full=Ba-alpha-2-glycoprotein; AltName: Full=Alpha-2-Z-globulin; AltName: Full=Fetuin-A; Contains: RecName: Full=Alpha-2-HS-glycoprotein chain A; Contains: RecName: Full=Alpha-2-HS-glycoprotein chain B; Flags: Precursor >gi|178284|gb|AAA51683.1| alpha-2-HS-glycoprotein [Homo sapiens] >gi|499139|gb|AAB29984.1| alpha 2-HS-glycoprotein, alpha 2HSG=insulin receptor inhibitor [human, liver, Peptide, 367 aa] >gi|7106502|dbj|BAA92189.1| alpha2-HS glycoprotein [Homo sapiens] >gi|29387000|gb|AAH48198.1| Alpha-2-HS-glycoprotein [Homo sapiens] >gi|30851645|gb|AAH52590.1| Alpha-2-HS-glycoprotein [Homo sapiens] >gi|119598595|gb|EAW78189.1| alpha-2-HS-glycoprotein, isoform CRA_c [Homo sapiens] >gi|189066558|dbj|BAG35808.1| unnamed protein product [Homo sapiens] | 544.15 | 47.14% | 8 | 1 | 11 | 343 | 367 | 39.3 | 5.72 | 0.777 |
| 119598593 | alpha-2-HS-glycoprotein, isoform CRA_a [Homo sapiens] | 532.05 | 46.99% | 2 | 1 | 11 | 318 | 366 | 39.2 | 5.72 | 0.78 |
| 194383496 | unnamed protein product [Homo sapiens] | 925.68 | 45.45% | 12 | 1 | 21 | 152 | 605 | 68.3 | 5.82 | 0.782 |
| 70798731 | immunoglobulin kappa light chain variable region [Homo sapiens] | 154.27 | 53.42% | 425 | 1 | 3 | 25 | 73 | 8 | 8.53 | 0.782 |
| 33325 | unnamed protein product [Homo sapiens] | 232.03 | 52.99% | 692 | 1 | 4 | 31 | 134 | 14.5 | 7.96 | 0.787 |
| 189066534 | unnamed protein product [Homo sapiens] | 76.02 | 20.00% | 4 | 2 | 2 | 3 | 175 | 20 | 9.01 | 0.788 |
| 158255874 | unnamed protein product [Homo sapiens] | 487.27 | 17.28% | 9 | 12 | 12 | 53 | 1065 | 122.1 | 5.74 | 0.796 |
| 74355107 | BRF1 protein [Homo sapiens] | 30.09 | 4.97% | 11 | 1 | 1 | 11 | 161 | 18.3 | 8.62 | 0.796 |
| 194273292 | immunoglobulin heavy chain variable region [Homo sapiens] | 227.62 | 70.30% | 896 | 1 | 6 | 13 | 101 | 11 | 9.72 | 0.8 |
| 62739186 | complement factor H isoform a precursor [Homo sapiens] >gi|56203410|emb|CAI19672.1| complement factor H [Homo sapiens] >gi|119611666|gb|EAW91260.1| hCG40889, isoform CRA_b [Homo sapiens] >gi|119611667|gb|EAW91261.1| hCG40889, isoform CRA_b [Homo sapiens] | 1928.15 | 45.90% | 10 | 1 | 46 | 524 | 1231 | 139 | 6.62 | 0.802 |
| 118406205 | immunoglobulin heavy chain variable region [Homo sapiens] | 58.92 | 22.22% | 231 | 1 | 2 | 7 | 117 | 12.5 | 8.68 | 0.803 |
| 1655598 | lipopolysaccharide binding protein [Homo sapiens] >gi|4530277|gb|AAD21962.1| lipopolysaccharide-binding protein [Homo sapiens] | 464.59 | 23.70% | 7 | 10 | 10 | 75 | 481 | 53.3 | 6.7 | 0.803 |
| 195452250 | immunoglobulin lambda light chain variable region [Homo sapiens] | 32.98 | 19.75% | 4 | 1 | 1 | 10 | 81 | 8.4 | 4.44 | 0.803 |
| 148733226 | serpin peptidase inhibitor, clade A (alpha-1 antiproteinase, antitrypsin), member 10 [Homo sapiens] | 73.38 | 8.11% | 5 | 3 | 3 | 7 | 444 | 50.7 | 8.28 | 0.806 |
| 189067450 | unnamed protein product [Homo sapiens] | 26.83 | 2.55% | 1 | 1 | 1 | 4 | 275 | 32.3 | 8.37 | 0.807 |
| 178775 | proapolipoprotein [Homo sapiens] | 1732.48 | 93.57% | 87 | 32 | 40 | 1407 | 249 | 28.9 | 5.6 | 0.816 |
| 5459317 | mannose binding lectin-associated serine protease-2 related protein, MAp19 (19kDa) [Homo sapiens] | 134.2 | 28.00% | 11 | 4 | 4 | 8 | 175 | 19.5 | 5.73 | 0.823 |
| 158256710 | unnamed protein product [Homo sapiens] | 235.85 | 9.06% | 11 | 8 | 8 | 18 | 1170 | 129.3 | 4.94 | 0.828 |
| 184086 | histone H2B.1 [Homo sapiens] | 30.74 | 8.91% | 34 | 1 | 1 | 6 | 101 | 11.3 | 10.14 | 0.828 |
| 126273569 | carboxypeptidase B2 isoform a preproprotein [Homo sapiens] >gi|62899885|sp|Q96IY4.1|CBPB2_HUMAN RecName: Full=Carboxypeptidase B2; AltName: Full=Carboxypeptidase U; Short=CPU; AltName: Full=Thrombin-activable fibrinolysis inhibitor; Short=TAFI; AltName: Full=Plasma carboxypeptidase B; Short=pCPB; Flags: Precursor >gi|13937897|gb|AAH07057.1| Carboxypeptidase B2 (plasma) [Homo sapiens] >gi|30582711|gb|AAP35582.1| carboxypeptidase B2 (plasma, carboxypeptidase U) [Homo sapiens] >gi|51234145|gb|AAT97987.1| carboxypeptidase B2 (plasma, carboxypeptidase U) [Homo sapiens] >gi|60656513|gb|AAX32820.1| carboxypeptidase B2 [synthetic construct] >gi|60656515|gb|AAX32821.1| carboxypeptidase B2 [synthetic construct] >gi|119629160|gb|EAX08755.1| carboxypeptidase B2 (plasma, carboxypeptidase U), isoform CRA_b [Homo sapiens] | 34.26 | 1.89% | 3 | 1 | 1 | 3 | 423 | 48.4 | 7.71 | 0.833 |
| 111917949 | immunoglobulin heavy chain variable region [Homo sapiens] | 76.7 | 18.18% | 77 | 1 | 2 | 8 | 154 | 16.7 | 7.84 | 1.201 |
| 194385976 | unnamed protein product [Homo sapiens] | 42.78 | 8.86% | 5 | 1 | 1 | 3 | 158 | 17.6 | 7.15 | 1.204 |
| 4557871 | serotransferrin precursor [Homo sapiens] >gi|136191|sp|P02787.2|TRFE_HUMAN RecName: Full=Serotransferrin; Short=Transferrin; AltName: Full=Siderophilin; AltName: Full=Beta-1 metal-binding globulin; Flags: Precursor >gi|248648|gb|AAB22049.1| transferrin [Homo sapiens] >gi|339453|gb|AAA61140.1| transferrin precursor [Homo sapiens] >gi|15021381|gb|AAK77664.1| transferin [Homo sapiens] >gi|31415705|gb|AAP45055.1| transferrin [Homo sapiens] >gi|94717618|gb|ABF47110.1| transferrin [Homo sapiens] >gi|119599573|gb|EAW79167.1| transferrin, isoform CRA_d [Homo sapiens] | 635.11 | 29.94% | 18 | 15 | 15 | 60 | 698 | 77 | 7.12 | 1.21 |
| 567112 | OMM protein (Ig gamma3) heavy chain [Homo sapiens] | 307.84 | 28.75% | 3 | 1 | 7 | 91 | 313 | 34.9 | 7.49 | 1.211 |
| 4960066 | apolipoprotein A1 [Homo sapiens] | 352.13 | 79.10% | 83 | 1 | 9 | 252 | 67 | 7.4 | 6.95 | 1.212 |
| 9295301 | immunoglobulin light chain variable region [Homo sapiens] | 109.65 | 26.13% | 23 | 1 | 2 | 26 | 111 | 12.3 | 8.85 | 1.214 |
| 532598 | Ig J-chain [Homo sapiens] | 192.26 | 45.26% | 3 | 3 | 3 | 21 | 137 | 15.6 | 4.73 | 1.215 |
| 20377087 | intestinal lactoferrin receptor [Homo sapiens] >gi|7019846|dbj|BAA90893.1| unnamed protein product [Homo sapiens] >gi|8096221|dbj|BAA96094.1| intelectin [Homo sapiens] >gi|18091783|gb|AAL58073.1| endothelial lectin HL-1 [Homo sapiens] >gi|37181843|gb|AAQ88725.1| ITLN [Homo sapiens] >gi|37724012|gb|AAO17801.1| intelectin [Homo sapiens] >gi|44976129|gb|AAS49907.1| omentin [Homo sapiens] >gi|48146565|emb|CAG33505.1| ITLN1 [Homo sapiens] >gi|52843233|gb|AAU88047.1| intelectin 1 [Homo sapiens] >gi|62896625|dbj|BAD96253.1| intelectin variant [Homo sapiens] | 171.75 | 29.07% | 7 | 7 | 7 | 13 | 313 | 35 | 5.82 | 1.218 |
| 1769552 | von Willebrand factor [Homo sapiens] | 27.48 | 3.73% | 10 | 1 | 1 | 2 | 241 | 27.1 | 6.93 | 1.223 |
| 70798883 | immunoglobulin kappa light chain variable region [Homo sapiens] | 93.38 | 25.24% | 97 | 1 | 2 | 2 | 103 | 11.1 | 8.88 | 1.227 |
| 189181722 | proteoglycan 4 isoform C [Homo sapiens] | 244.6 | 6.41% | 12 | 8 | 8 | 19 | 1311 | 141 | 9.39 | 1.228 |
| 194385488 | unnamed protein product [Homo sapiens] | 67.07 | 4.23% | 18 | 2 | 2 | 2 | 780 | 82.5 | 6.55 | 1.231 |
| 1369904 | serum lectin P35 [Homo sapiens] >gi|1669349|dbj|BAA09636.1| lectin P35 [Homo sapiens] | 342.33 | 30.99% | 6 | 8 | 9 | 95 | 313 | 34 | 6.54 | 1.239 |
| 157778872 | immunoglobulin heavy chain variable region [Homo sapiens] | 45.74 | 17.39% | 231 | 1 | 2 | 7 | 115 | 12.8 | 9.01 | 1.241 |
| 194390048 | unnamed protein product [Homo sapiens] | 349.81 | 47.06% | 17 | 9 | 9 | 31 | 272 | 30.8 | 6.6 | 1.243 |
| 119573006 | apolipoprotein A-II, isoform CRA_c [Homo sapiens] | 399.98 | 67.50% | 1 | 1 | 8 | 484 | 80 | 9 | 6.57 | 1.251 |
| 189066554 | unnamed protein product [Homo sapiens] | 924.02 | 44.21% | 5 | 1 | 21 | 151 | 622 | 70 | 5.9 | 1.269 |
| 21669819 | immunoglobulin heavy chain VHDJ region [Homo sapiens] | 90.22 | 25.00% | 417 | 1 | 2 | 7 | 120 | 13.1 | 5.85 | 1.27 |
| 4502027 | serum albumin preproprotein [Homo sapiens] >gi|197098046|ref|NP_001127106.1| serum albumin precursor [Pongo abelii] >gi|113576|sp|P02768.2|ALBU_HUMAN RecName: Full=Serum albumin; Flags: Precursor >gi|75054626|sp|Q5NVH5.1|ALBU_PONAB RecName: Full=Serum albumin; Flags: Precursor >gi|7770117|gb|AAF69594.1|AF119917_2 PRO0903 [Homo sapiens] >gi|178344|gb|AAA98797.1| albumin [Homo sapiens] >gi|21706456|gb|AAH34023.1| Albumin [Homo sapiens] >gi|23243418|gb|AAH36003.1| Albumin [Homo sapiens] >gi|49176517|gb|AAT52213.1| cell growth inhibiting protein 42 [Homo sapiens] >gi|52001697|gb|AAU21642.1| serum albumin precursor [Homo sapiens] >gi|56403804|emb|CAI29688.1| hypothetical protein [Pongo abelii] >gi|115607207|gb|ABJ16448.1| serum albumin [Homo sapiens] >gi|119626081|gb|EAX05676.1| albumin, isoform CRA_r [Homo sapiens] >gi|123981022|gb|ABM82340.1| albumin [synthetic construct] >gi|123995825|gb|ABM85514.1| albumin [synthetic construct] >gi|152112964|gb|ABS29264.1| albumin [Homo sapiens] | 1969.11 | 76.35% | 36 | 24 | 45 | 799 | 609 | 69.3 | 6.28 | 1.273 |
| 553181 | angiotensinogen [Homo sapiens] | 86.02 | 7.40% | 13 | 2 | 2 | 4 | 338 | 36.5 | 7.05 | 1.273 |
| 221044808 | unnamed protein product [Homo sapiens] | 419.54 | 29.03% | 4 | 11 | 11 | 74 | 534 | 59.8 | 6.43 | 1.275 |
| 21669523 | immunoglobulin lambda light chain VLJ region [Homo sapiens] | 427.3 | 39.63% | 106 | 1 | 10 | 568 | 270 | 28.1 | 7.14 | 1.287 |
| 81295708 | immunoglobulin light chain variable region [Homo sapiens] | 151.35 | 37.72% | 120 | 1 | 4 | 9 | 114 | 12.1 | 8.02 | 1.288 |
| 190194 | serum paraoxonase [Homo sapiens] | 414.48 | 45.70% | 15 | 1 | 10 | 71 | 337 | 37.8 | 5.08 | 1.293 |
| 21668722 | immunoglobulin heavy chain VHDJ region [Homo sapiens] | 99.89 | 18.11% | 424 | 1 | 2 | 8 | 127 | 13.7 | 8.85 | 1.298 |
| 86438980 | immunoglobulin heavy chain [Homo sapiens] | 193.56 | 22.37% | 31 | 1 | 4 | 51 | 219 | 23.5 | 8.91 | 1.298 |
| 37287526 | paraneoplastic pemphigus associated Castleman's disease immunoglobulin light chain variable region [Homo sapiens] | 24.41 | 15.93% | 1 | 1 | 1 | 1 | 113 | 12.6 | 7.12 | 1.313 |
| 119608995 | pregnancy-zone protein, isoform CRA_b [Homo sapiens] | 382.81 | 10.06% | 7 | 5 | 11 | 39 | 1481 | 163.6 | 6.42 | 1.317 |
| 12803959 | Tropomyosin 4 [Homo sapiens] | 101.51 | 16.13% | 10 | 4 | 4 | 7 | 248 | 28.6 | 4.69 | 1.318 |
| 33319680 | Ig heavy chain variable region, VH3 family [Homo sapiens] | 172.21 | 39.34% | 1007 | 1 | 4 | 29 | 122 | 13.1 | 8.85 | 1.32 |
| 681900 | This CDS feature is included to show the translation of the corresponding V_region. Presently translation qualifiers on V_region features are illegal [Homo sapiens] | 138.54 | 44.86% | 293 | 1 | 3 | 9 | 107 | 11.6 | 8.51 | 1.323 |
| 55957679 | cartilage acidic protein 1 [Homo sapiens] >gi|55958790|emb|CAI14275.1| cartilage acidic protein 1 [Homo sapiens] | 68.51 | 5.34% | 10 | 3 | 3 | 5 | 524 | 56.5 | 5.55 | 1.334 |
| 21628665 | immunoglobulin light chain constant region [Homo sapiens] | 116.72 | 91.43% | 1 | 1 | 3 | 82 | 35 | 3.9 | 6.48 | 1.335 |
| 156765940 | collectin kidney K1 [Homo sapiens] | 260.61 | 27.41% | 26 | 7 | 7 | 33 | 197 | 21.6 | 4.75 | 1.354 |
| 553426 | immunoglobulin heavy chain VDJC region [Homo sapiens] | 103.19 | 14.58% | 39 | 1 | 3 | 36 | 144 | 15.6 | 9.13 | 1.361 |
| 13661193 | fibulin-1 isoform D precursor [Homo sapiens] >gi|1621019|gb|AAB17099.1| fibulin-1D [Homo sapiens] | 158.68 | 9.25% | 23 | 5 | 5 | 24 | 703 | 77.2 | 5.26 | 1.365 |
| 110626504 | anti-SARS-CoV S protein immunoglobulin kappa light chain [Homo sapiens] | 505.36 | 43.93% | 58 | 1 | 8 | 576 | 214 | 23.6 | 7.71 | 1.374 |
| 4323944 | immunoglobulin kappa light chain variable region [Homo sapiens] | 230.86 | 71.72% | 692 | 1 | 4 | 31 | 99 | 10.9 | 9.57 | 1.38 |
| 54780200 | immunoglobulin mu heavy chain [Homo sapiens] | 270.49 | 33.18% | 525 | 1 | 5 | 66 | 211 | 22.7 | 8.29 | 1.382 |
| 194383694 | unnamed protein product [Homo sapiens] | 112.48 | 6.53% | 10 | 3 | 3 | 10 | 704 | 77.2 | 4.53 | 1.391 |
| 4557389 | complement component C8 alpha chain precursor [Homo sapiens] >gi|729167|sp|P07357.2|CO8A_HUMAN RecName: Full=Complement component C8 alpha chain; AltName: Full=Complement component 8 subunit alpha; Flags: Precursor >gi|179718|gb|AAA52200.1| complement protein C8 alpha subunit precursor [Homo sapiens] >gi|56203387|emb|CAI19172.1| complement component 8, alpha polypeptide [Homo sapiens] >gi|124376216|gb|AAI32914.1| Complement component 8, alpha polypeptide [Homo sapiens] | 83.59 | 7.71% | 7 | 3 | 3 | 4 | 584 | 65.1 | 6.47 | 1.391 |
| 15886930 | immunoglobulin heavy chain variable region [Homo sapiens] | 122.46 | 46.61% | 493 | 1 | 4 | 11 | 118 | 12.9 | 8.41 | 1.392 |
| 1620018 | heat shock protein 90 [Homo sapiens] | 69.98 | 17.88% | 10 | 1 | 1 | 4 | 151 | 16.8 | 4.79 | 1.395 |
| 5174411 | CD5 antigen-like precursor [Homo sapiens] >gi|20177834|sp|O43866.1|CD5L_HUMAN RecName: Full=CD5 antigen-like; AltName: Full=SP-alpha; AltName: Full=CT-2; AltName: Full=IgM-associated peptide; Flags: Precursor >gi|2702314|gb|AAB91989.1| Sp alpha [Homo sapiens] >gi|4102235|gb|AAD01446.1| AIM [Homo sapiens] >gi|11967471|emb|CAC19458.1| CD5 molecule-like [Homo sapiens] >gi|21707924|gb|AAH33586.1| CD5 molecule-like [Homo sapiens] >gi|119573244|gb|EAW52859.1| CD5 antigen-like (scavenger receptor cysteine rich family) [Homo sapiens] >gi|123993879|gb|ABM84541.1| CD5 molecule-like [synthetic construct] >gi|123997245|gb|ABM86224.1| CD5 molecule-like [synthetic construct] >gi|158257512|dbj|BAF84729.1| unnamed protein product [Homo sapiens] | 466.86 | 47.84% | 2 | 14 | 14 | 57 | 347 | 38.1 | 5.47 | 1.399 |
| 4321593 | immunoglobulin G kappa chain [Homo sapiens] | 361.42 | 38.25% | 71 | 1 | 5 | 126 | 217 | 24 | 6.55 | 1.4 |
| 21518719 | hyaluronidase 1 variant 5 [Homo sapiens] >gi|119585494|gb|EAW65090.1| hyaluronoglucosaminidase 1, isoform CRA_f [Homo sapiens] | 46.2 | 25.00% | 12 | 1 | 1 | 2 | 96 | 10.4 | 8.53 | 1.41 |
| 189069199 | unnamed protein product [Homo sapiens] | 652.37 | 45.91% | 15 | 18 | 18 | 56 | 464 | 52.6 | 6.71 | 1.412 |
| 62088684 | Integrin alpha-IIb precursor variant [Homo sapiens] | 35.75 | 4.17% | 13 | 2 | 2 | 2 | 551 | 59.2 | 6.05 | 1.414 |
| 33319650 | Ig heavy chain variable region, VH3 family [Homo sapiens] | 100.27 | 34.17% | 151 | 1 | 3 | 5 | 120 | 13.1 | 6.52 | 1.415 |
| 27369048 | immunoglobulin lambda light chain variable region [Homo sapiens] | 10.42 | 9.29% | 1 | 1 | 1 | 1 | 140 | 14.5 | 7.06 | 1.42 |
| 77379428 | immunoglobulin kappa chain variable region [Homo sapiens] | 38.02 | 21.49% | 1 | 1 | 1 | 2 | 121 | 13.1 | 6 | 1.434 |
| 587144 | anti-Desmosome antibody light chain variable region mRNA [Homo sapiens] | 69.21 | 39.47% | 16 | 1 | 3 | 5 | 114 | 12.5 | 7.96 | 1.436 |
| 37789717 | immunoglobulin lambda light chain variable region [Homo sapiens] | 37.12 | 9.09% | 4 | 1 | 1 | 3 | 88 | 9.5 | 5.17 | 1.437 |
| 21669285 | immunoglobulin lambda light chain VLJ region [Homo sapiens] | 43.55 | 32.43% | 4 | 1 | 2 | 3 | 111 | 11.7 | 8.91 | 1.442 |
| 55669575 | Chain A, A Covalent Dimer Of Transthyretin That Affects The Amyloid Pathway >gi|55669576|pdb|1QWH|B Chain B, A Covalent Dimer Of Transthyretin That Affects The Amyloid Pathway >gi|339685|gb|AAA61181.1| transthyretin [Homo sapiens] | 291.36 | 66.67% | 11 | 7 | 7 | 86 | 117 | 12.8 | 5.45 | 1.45 |
| 54779153 | immunoglobulin mu heavy chain [Homo sapiens] | 192.36 | 29.67% | 735 | 1 | 5 | 64 | 209 | 22.6 | 7.68 | 1.453 |
| 18307322 | immunoglobulin light chain lambda variable region [Homo sapiens] | 27.87 | 12.62% | 1 | 1 | 1 | 1 | 103 | 10.8 | 5.25 | 1.464 |
| 87299014 | immunoglobulin light chain variable region [Homo sapiens] | 454.36 | 71.05% | 348 | 1 | 9 | 514 | 152 | 16.1 | 4.93 | 1.467 |
| 7012705 | immunoglobulin light chain variable region [Homo sapiens] | 212.91 | 68.22% | 425 | 1 | 4 | 27 | 107 | 11.5 | 7.96 | 1.467 |
| 221044282 | unnamed protein product [Homo sapiens] | 229.04 | 9.77% | 4 | 5 | 5 | 15 | 870 | 95.9 | 4.64 | 1.49 |
| 114147277 | immunoglobulin heavy chain variable region [Homo sapiens] | 74.17 | 22.73% | 41 | 1 | 2 | 12 | 110 | 12.3 | 8.29 | 1.495 |
| 3004786 | Ig heavy chain variable region [Homo sapiens] | 109.28 | 29.66% | 145 | 1 | 3 | 19 | 118 | 13.1 | 7.97 | 1.514 |
| 221042196 | unnamed protein product [Homo sapiens] | 99.51 | 6.36% | 8 | 3 | 3 | 14 | 645 | 72.1 | 7.68 | 1.517 |
| 215982760 | immunoglobulin lambda light chain variable region [Homo sapiens] | 150.34 | 27.66% | 13 | 1 | 3 | 214 | 141 | 15.1 | 5.45 | 1.529 |
| 7770217 | PRO2675 [Homo sapiens] >gi|119626082|gb|EAX05677.1| albumin, isoform CRA_s [Homo sapiens] | 939.35 | 78.47% | 5 | 1 | 22 | 318 | 288 | 32.6 | 6.54 | 1.557 |
| 119600101 | phosphatidylinositol-specific phospholipase C, X domain containing 2 [Homo sapiens] | 34.44 | 2.42% | 3 | 1 | 1 | 1 | 289 | 33 | 9.44 | 1.586 |
| 247425451 | immunoglobulin heavy chain variable region [Homo sapiens] | 125.32 | 24.59% | 743 | 1 | 2 | 24 | 122 | 13.1 | 6.57 | 1.588 |
| 109240688 | immunoglobulin kappa chain variable region [Homo sapiens] | 41.25 | 15.09% | 2 | 1 | 1 | 3 | 106 | 11.7 | 7.99 | 1.599 |
| 6707433 | apolipoprotein A5 [Homo sapiens] >gi|6707435|gb|AAF25662.1|AF202890_1 apolipoprotein A5 [Homo sapiens] >gi|167887490|gb|ACA05939.1| apolipoprotein A-V precursor variant 3 [Homo sapiens] >gi|189054886|dbj|BAG37658.1| unnamed protein product [Homo sapiens] | 30.29 | 6.34% | 4 | 1 | 1 | 1 | 363 | 40.9 | 6.43 | 1.6 |
| 15080499 | Serpin peptidase inhibitor, clade A (alpha-1 antiproteinase, antitrypsin), member 1 [Homo sapiens] | 343.07 | 34.93% | 29 | 11 | 11 | 63 | 418 | 46.7 | 5.59 | 1.615 |
| 247424903 | immunoglobulin heavy chain variable region [Homo sapiens] | 286.5 | 51.22% | 1252 | 1 | 5 | 69 | 123 | 13.4 | 7.96 | 1.616 |
| 4467842 | immunoglobulin M heavy chain [Homo sapiens] | 1329.03 | 66.45% | 17 | 1 | 25 | 1256 | 453 | 49.4 | 6.77 | 1.621 |
| 58222449 | anti-tetanus toxoid immunoglobulin heavy chain variable region [Homo sapiens] | 102.65 | 21.49% | 133 | 1 | 3 | 20 | 121 | 13.2 | 8.46 | 1.627 |
| 34534492 | unnamed protein product [Homo sapiens] | 13.39 | 8.66% | 1 | 1 | 1 | 1 | 277 | 30.5 | 8.56 | 1.636 |
| 37694587 | immunoglobulin heavy chain variable region [Homo sapiens] | 81.8 | 25.64% | 12 | 1 | 2 | 5 | 117 | 12.9 | 7.39 | 1.645 |
| 12733966 | immunoglobulin gamma heavy chain variable region [Homo sapiens] | 167.4 | 39.32% | 1183 | 1 | 4 | 41 | 117 | 13 | 6.04 | 1.689 |
| 33235626 | immunoglobulin kappa light chain [Homo sapiens] | 146.86 | 31.48% | 467 | 1 | 2 | 22 | 108 | 11.7 | 9.17 | 1.694 |
| 219566389 | immunoglobulin kappa light chain [Homo sapiens] | 132.14 | 39.29% | 540 | 1 | 3 | 31 | 112 | 12.4 | 8.94 | 1.732 |
| 2253348 | immunoglobulin heavy chain variable region [Homo sapiens] | 69.15 | 22.13% | 31 | 1 | 2 | 3 | 122 | 13.4 | 9.07 | 1.751 |
| 11122875 | glycosylphosphatidylinositol phospholipase D [Homo sapiens] | 137.2 | 8.32% | 8 | 3 | 3 | 4 | 457 | 48.5 | 6.2 | 1.765 |
| 18041890 | immunoglobulin lambda light chain variable region [Homo sapiens] | 35.59 | 9.09% | 3 | 1 | 1 | 7 | 88 | 9.2 | 5.24 | 1.789 |
| 114665902 | PREDICTED: similar to Profilin-1 (Profilin I) [Pan troglodytes] >gi|119610788|gb|EAW90382.1| profilin 1, isoform CRA_b [Homo sapiens] | 41.59 | 7.69% | 2 | 1 | 1 | 1 | 104 | 11.4 | 9.17 | 1.791 |
| 553490 | immunoglobulin lambda-chain [Homo sapiens] | 64.73 | 12.50% | 89 | 1 | 2 | 7 | 128 | 13.4 | 7.81 | 1.906 |
| 11558186 | immunoglobulin light chain variable region [Homo sapiens] | 33.94 | 6.06% | 1 | 1 | 1 | 2 | 132 | 13.7 | 8.94 | 1.906 |
| 1620396 | haptoglobin [Homo sapiens] | 370.62 | 34.77% | 7 | 4 | 11 | 42 | 348 | 39 | 6.89 | 1.989 |
| 18092610 | anti-cardiolipin immunoglobulin light chain [Homo sapiens] | 115.07 | 26.85% | 23 | 1 | 2 | 27 | 108 | 12 | 8.63 | 2.014 |
| 194390508 | unnamed protein product [Homo sapiens] | 1308.75 | 40.93% | 24 | 1 | 25 | 226 | 1014 | 111.2 | 6.21 | 2.021 |
| 33319656 | Ig heavy chain variable region, VH3 family [Homo sapiens] | 122.03 | 30.83% | 763 | 1 | 3 | 26 | 120 | 13.1 | 7.14 | 2.069 |
| 8777875 | immunoglobulin light chain variable region [Homo sapiens] | 174.72 | 37.61% | 423 | 1 | 3 | 26 | 109 | 12 | 8.48 | 2.19 |
| 587350 | immunoglobulin kappa light chain variable region [Homo sapiens] | 67.14 | 29.91% | 10 | 1 | 3 | 5 | 117 | 12.6 | 7.12 | 2.222 |
| 6643569 | immunoglobulin lambda light chain variable region [Homo sapiens] >gi|6643571|gb|AAF20644.1| immunoglobulin lambda light chain variable region [Homo sapiens] | 46.99 | 25.93% | 193 | 1 | 2 | 2 | 108 | 11.5 | 5.96 | 2.266 |
| 247425006 | immunoglobulin heavy chain variable region [Homo sapiens] | 179.48 | 39.34% | 421 | 1 | 4 | 50 | 122 | 13.1 | 8.81 | 2.4 |
| 33319586 | Ig heavy chain variable region, VH3 family [Homo sapiens] | 125.55 | 25.00% | 742 | 1 | 2 | 23 | 120 | 13 | 4.77 | 2.44 |
| 1082521 | Ig kappa chain V region - human (fragment) >gi|809555|emb|CAA58108.1| immunoglobulin kappa light chain [Homo sapiens] | 87.06 | 36.84% | 16 | 1 | 3 | 3 | 114 | 12.3 | 8.94 | 2.469 |
| 346196 | Ig lambda chain V region - human >gi|452933|gb|AAB28790.1| anti-cardiolipin/beta 2 glycoprotein I immunoglobulin light chain variable region [Homo sapiens] | 102.42 | 21.67% | 284 | 1 | 3 | 22 | 120 | 12.4 | 6.48 | 2.742 |
| 221042228 | unnamed protein product [Homo sapiens] | 15.94 | 2.78% | 1 | 1 | 1 | 1 | 539 | 61.3 | 7.74 | 3.382 |
| 94469923 | anti-West Nile virus immunoglobulin light chain variable region [Homo sapiens] | 38.87 | 21.82% | 1 | 1 | 1 | 1 | 110 | 12 | 7.96 | 6.163 |

**ASD vs. Control**

| Accession | Description | Score | Coverage | # Proteins | # Unique Peptides | # Peptides | # PSMs | # AAs | MW [kDa] | calc. pI | ASDdanchun/  Control |
| --- | --- | --- | --- | --- | --- | --- | --- | --- | --- | --- | --- |
| 13195586 | hemoglobin alpha 1 globin chain [Homo sapiens] | 103.91 | 24.00% | 13 | 2 | 2 | 25 | 100 | 10.7 | 7.72 | 0.18 |
| 18418633 | mutant beta-globin [Homo sapiens] | 289.03 | 71.43% | 76 | 7 | 8 | 59 | 147 | 16 | 7.69 | 0.216 |
| 124504316 | HIST2H4B protein [Homo sapiens] | 46.54 | 9.80% | 6 | 1 | 1 | 1 | 102 | 11.4 | 11.36 | 0.294 |
| 194375974 | unnamed protein product [Homo sapiens] | 60.43 | 9.84% | 5 | 2 | 2 | 3 | 183 | 20.1 | 8.78 | 0.354 |
| 18041906 | immunoglobulin lambda light chain variable region [Homo sapiens] | 44.07 | 32.29% | 193 | 1 | 2 | 2 | 96 | 10.3 | 7.97 | 0.4 |
| 32483410 | vitamin D-binding protein precursor [Homo sapiens] | 803.24 | 58.86% | 3 | 1 | 22 | 75 | 474 | 52.9 | 5.45 | 0.435 |
| 21669937 | immunoglobulin heavy chain VHDJ region [Homo sapiens] | 114.57 | 18.55% | 423 | 1 | 2 | 11 | 124 | 13.5 | 9.36 | 0.456 |
| 119626442 | multimerin 1, isoform CRA_a [Homo sapiens] | 62.55 | 3.58% | 6 | 1 | 1 | 5 | 531 | 58.1 | 8.72 | 0.502 |
| 183851 | G-gamma-hemoglobin [Homo sapiens] | 64.89 | 22.77% | 15 | 1 | 2 | 21 | 101 | 11 | 6.68 | 0.507 |
| 4505733 | platelet factor 4 precursor [Homo sapiens] >gi|130304|sp|P02776.2|PLF4_HUMAN RecName: Full=Platelet factor 4; Short=PF-4; AltName: Full=C-X-C motif chemokine 4; AltName: Full=Oncostatin-A; AltName: Full=Iroplact; Contains: RecName: Full=Platelet factor 4, short form; Flags: Precursor >gi|13549118|gb|AAK29643.1|AF349466_3 platelet factor 4 [Homo sapiens] >gi|189851|gb|AAA60066.1| platelet factor 4 [Homo sapiens] >gi|47115291|emb|CAG28605.1| PF4 [Homo sapiens] >gi|62739642|gb|AAH93965.1| Platelet factor 4 [Homo sapiens] >gi|63994325|gb|AAY41003.1| unknown [Homo sapiens] >gi|85567544|gb|AAI12094.1| Platelet factor 4 [Homo sapiens] >gi|119626099|gb|EAX05694.1| platelet factor 4 (chemokine (C-X-C motif) ligand 4) [Homo sapiens] >gi|261859268|dbj|BAI46156.1| platelet factor 4 [synthetic construct] | 125.57 | 34.65% | 1 | 1 | 3 | 22 | 101 | 10.8 | 8.62 | 0.525 |
| 76252669 | immunoglobulin lambda light chain variable region [Homo sapiens] | 33.83 | 17.65% | 2 | 1 | 1 | 4 | 102 | 10.9 | 7.12 | 0.545 |
| 169672524 | transforming growth factor beta 1 precursor [Homo sapiens] | 51.7 | 16.10% | 7 | 1 | 1 | 1 | 118 | 12.9 | 7.2 | 0.569 |
| 2809025 | Ig heavy chain variable region [Homo sapiens] | 41.55 | 42.25% | 134 | 1 | 2 | 4 | 71 | 7.9 | 9.31 | 0.57 |
| 34532317 | unnamed protein product [Homo sapiens] | 19.57 | 0.89% | 9 | 1 | 1 | 1 | 1014 | 113.4 | 9.03 | 0.574 |
| 10636616 | immunoglobulin heavy chain variable region [Homo sapiens] | 89.37 | 18.97% | 627 | 1 | 2 | 22 | 116 | 12.9 | 7.85 | 0.576 |
| 31873302 | hypothetical protein [Homo sapiens] >gi|117646030|emb|CAL38482.1| hypothetical protein [synthetic construct] | 241.85 | 24.19% | 27 | 7 | 7 | 15 | 434 | 47.1 | 7.69 | 0.577 |
| 112700542 | immunoglobulin heavy chain variable region [Homo sapiens] | 154.72 | 43.56% | 1441 | 1 | 4 | 24 | 101 | 10.9 | 8.29 | 0.584 |
| 27650476 | immunoglobulin heavy chain variable region [Homo sapiens] | 57.64 | 46.48% | 1 | 1 | 2 | 2 | 71 | 8 | 8.72 | 0.588 |
| 1769552 | von Willebrand factor [Homo sapiens] | 27.48 | 3.73% | 10 | 1 | 1 | 2 | 241 | 27.1 | 6.93 | 0.6 |
| 54304028 | glyceraldehyde-3-phosphate dehydrogenase [Homo sapiens] | 45.44 | 17.44% | 7 | 1 | 1 | 3 | 86 | 9.2 | 9.72 | 0.611 |
| 30583505 | catenin (cadherin-associated protein), delta 1 [Homo sapiens] | 42.11 | 3.28% | 25 | 1 | 1 | 1 | 610 | 68 | 8.13 | 0.617 |
| 207028494 | L-lactate dehydrogenase A chain isoform 2 [Homo sapiens] >gi|194383812|dbj|BAG59264.1| unnamed protein product [Homo sapiens] | 36.64 | 7.30% | 5 | 1 | 1 | 3 | 274 | 30.2 | 7.15 | 0.629 |
| 55958543 | heterogeneous nuclear ribonucleoprotein K [Homo sapiens] | 39.26 | 5.23% | 12 | 1 | 1 | 1 | 306 | 34 | 5.78 | 0.631 |
| 3152372 | anti-FactorVIII scFv [Homo sapiens] | 537.51 | 64.29% | 3758 | 1 | 10 | 90 | 238 | 25 | 8.41 | 0.637 |
| 146424184 | apolipoprotein C-IV [Homo sapiens] | 73.46 | 15.75% | 3 | 2 | 2 | 5 | 127 | 14.6 | 9.13 | 0.642 |
| 46254055 | immunoglobulin heavy chain [Homo sapiens] | 151.53 | 28.74% | 208 | 1 | 3 | 50 | 167 | 18 | 8.9 | 0.644 |
| 158256710 | unnamed protein product [Homo sapiens] | 235.85 | 9.06% | 11 | 8 | 8 | 18 | 1170 | 129.3 | 4.94 | 0.665 |
| 587273 | Immunoglobulin heavy chain variable region [Homo sapiens] | 96.48 | 27.27% | 59 | 1 | 2 | 27 | 132 | 14 | 8.46 | 0.674 |
| 71773201 | adenine phosphoribosyltransferase isoform b [Homo sapiens] >gi|114664113|ref|XP_001137924.1| PREDICTED: adenine phosphoribosyltransferase isoform 1 [Pan troglodytes] >gi|119587165|gb|EAW66761.1| adenine phosphoribosyltransferase, isoform CRA_b [Homo sapiens] | 26.87 | 14.93% | 2 | 1 | 1 | 1 | 134 | 14.5 | 7.34 | 0.677 |
| 56378229 | carbamoylphosphate synthetase I [Homo sapiens] | 22.22 | 4.09% | 12 | 1 | 1 | 1 | 513 | 55.7 | 8.94 | 0.681 |
| 112701317 | immunoglobulin heavy chain variable region [Homo sapiens] | 63.94 | 32.32% | 36 | 1 | 2 | 3 | 99 | 10.6 | 6.51 | 0.681 |
| 21668722 | immunoglobulin heavy chain VHDJ region [Homo sapiens] | 99.89 | 18.11% | 424 | 1 | 2 | 8 | 127 | 13.7 | 8.85 | 0.684 |
| 194385488 | unnamed protein product [Homo sapiens] | 67.07 | 4.23% | 18 | 2 | 2 | 2 | 780 | 82.5 | 6.55 | 0.689 |
| 4379074 | IgM rheumatoid factor immunoglobulin heavy chain [Homo sapiens] | 104.17 | 39.32% | 775 | 1 | 3 | 23 | 117 | 12.8 | 8.4 | 0.689 |
| 194389616 | unnamed protein product [Homo sapiens] | 23.6 | 2.10% | 28 | 1 | 1 | 1 | 525 | 59.7 | 7.12 | 0.691 |
| 119608546 | ficolin (collagen/fibrinogen domain containing) 1, isoform CRA_d [Homo sapiens] | 57.63 | 5.10% | 7 | 1 | 2 | 2 | 314 | 33.8 | 6.64 | 0.691 |
| 896272 | This CDS feature is included to show the translation of the corresponding V_region. Presently translation qualifiers on V_region features are illegal [Homo sapiens] | 285.25 | 64.57% | 2946 | 2 | 9 | 63 | 127 | 13.8 | 8.79 | 0.695 |
| 3337390 | haptoglobin [Homo sapiens] | 399.49 | 39.42% | 15 | 5 | 12 | 56 | 345 | 38.2 | 6.6 | 0.696 |
| 219566101 | immunoglobulin heavy chain [Homo sapiens] | 75.42 | 22.39% | 9 | 1 | 2 | 3 | 134 | 14.7 | 5.48 | 0.697 |
| 114062 | RecName: Full=Apolipoprotein(a); Short=Apo(a); Short=Lp(a); Flags: Precursor >gi|28620|emb|CAA29618.1| unnamed protein product [Homo sapiens] | 78.87 | 8.60% | 4 | 2 | 2 | 7 | 4548 | 501 | 5.88 | 0.698 |
| 416178 | desmoglein 2 [Homo sapiens] | 105.68 | 5.64% | 2 | 4 | 4 | 5 | 1117 | 122.3 | 5.27 | 0.703 |
| 181482 | serum vitamin D-binding protein precursor [Homo sapiens] | 825.06 | 59.28% | 2 | 2 | 23 | 76 | 474 | 53 | 5.54 | 0.704 |
| 94469923 | anti-West Nile virus immunoglobulin light chain variable region [Homo sapiens] | 38.87 | 21.82% | 1 | 1 | 1 | 1 | 110 | 12 | 7.96 | 0.706 |
| 156616294 | N-acetylmuramoyl-L-alanine amidase precursor [Homo sapiens] >gi|38258222|sp|Q96PD5.1|PGRP2_HUMAN RecName: Full=N-acetylmuramoyl-L-alanine amidase; AltName: Full=Peptidoglycan recognition protein long; Short=PGRP-L; AltName: Full=Peptidoglycan recognition protein 2; Flags: Precursor >gi|15705411|gb|AAL05629.1|AF384856_1 peptidoglycan recognition protein L precursor [Homo sapiens] >gi|119604888|gb|EAW84482.1| peptidoglycan recognition protein 2, isoform CRA_a [Homo sapiens] >gi|158258016|dbj|BAF84981.1| unnamed protein product [Homo sapiens] | 148.7 | 18.92% | 6 | 6 | 6 | 10 | 576 | 62.2 | 7.55 | 0.707 |
| 74355107 | BRF1 protein [Homo sapiens] | 30.09 | 4.97% | 11 | 1 | 1 | 11 | 161 | 18.3 | 8.62 | 0.714 |
| 119573007 | apolipoprotein A-II, isoform CRA_d [Homo sapiens] | 478.54 | 75.00% | 4 | 2 | 9 | 695 | 92 | 10.6 | 9.04 | 0.715 |
| 119598593 | alpha-2-HS-glycoprotein, isoform CRA_a [Homo sapiens] | 532.05 | 46.99% | 2 | 1 | 11 | 318 | 366 | 39.2 | 5.72 | 0.717 |
| 4505735 | platelet factor 4 variant [Homo sapiens] >gi|130306|sp|P10720.1|PF4V_HUMAN RecName: Full=Platelet factor 4 variant; AltName: Full=PF4var1; AltName: Full=PF4alt; AltName: Full=C-X-C motif chemokine 4 variant; AltName: Full=CXCL4L1; Contains: RecName: Full=Platelet factor 4 variant(4-74); Contains: RecName: Full=Platelet factor 4 variant(5-74); Contains: RecName: Full=Platelet factor 4 variant(6-74); Flags: Precursor >gi|292390|gb|AAA60067.1| platelet factor 4 [Homo sapiens] >gi|119626097|gb|EAX05692.1| platelet factor 4 variant 1 [Homo sapiens] >gi|120659980|gb|AAI30654.1| Platelet factor 4 variant 1 [Homo sapiens] >gi|120660120|gb|AAI30658.1| Platelet factor 4 variant 1 [Homo sapiens] | 168.33 | 48.08% | 1 | 2 | 4 | 9 | 104 | 11.5 | 9.1 | 0.72 |
| 19744552 | immunoglobulin light chain variable region [Homo sapiens] | 65.39 | 35.14% | 30 | 1 | 2 | 5 | 111 | 12.2 | 8.48 | 0.72 |
| 47271320 | immunoglobulin lambda light chain variable region [Homo sapiens] | 59.98 | 21.05% | 104 | 1 | 2 | 5 | 114 | 12.1 | 5.01 | 0.722 |
| 118406205 | immunoglobulin heavy chain variable region [Homo sapiens] | 58.92 | 22.22% | 231 | 1 | 2 | 7 | 117 | 12.5 | 8.68 | 0.722 |
| 2258128 | complement 9 [Homo sapiens] | 51.17 | 4.50% | 5 | 2 | 2 | 3 | 533 | 60.4 | 5.59 | 0.727 |
| 284434903 | thrombocidin-2 antimicrobial variant [Homo sapiens] | 56.55 | 19.84% | 4 | 2 | 2 | 3 | 126 | 13.7 | 9 | 0.729 |
| 178812 | apolipoprotein B-100 precursor [Homo sapiens] | 1998.27 | 17.34% | 21 | 1 | 59 | 189 | 4563 | 515.1 | 7.11 | 0.731 |
| 5419725 | immunoglobulin light chain variable region [Homo sapiens] | 150.43 | 31.48% | 258 | 1 | 3 | 10 | 108 | 11.5 | 7.12 | 0.732 |
| 119594857 | cofilin 1 (non-muscle), isoform CRA_c [Homo sapiens] | 91.11 | 40.88% | 6 | 4 | 4 | 5 | 137 | 15.6 | 8.35 | 0.737 |
| 158258641 | unnamed protein product [Homo sapiens] | 22.67 | 5.86% | 14 | 1 | 1 | 1 | 222 | 25.7 | 9 | 0.738 |
| 119590943 | fibronectin 1, isoform CRA_h [Homo sapiens] | 2674.5 | 37.42% | 82 | 36 | 60 | 502 | 2330 | 256.3 | 5.8 | 0.739 |
| 119570830 | hCG1741471 [Homo sapiens] | 23 | 3.11% | 7 | 1 | 1 | 2 | 322 | 37.2 | 7.99 | 0.739 |
| 18025670 | immunoglobulin light chain variable region [Homo sapiens] | 66.85 | 25.23% | 17 | 1 | 2 | 4 | 107 | 11.8 | 9.01 | 0.742 |
| 54779633 | immunoglobulin mu heavy chain [Homo sapiens] | 349.62 | 36.45% | 2295 | 1 | 7 | 185 | 214 | 23 | 7.02 | 0.748 |
| 28931 | beta-subunit (AA 1-312) [Homo sapiens] | 29.34 | 7.69% | 4 | 1 | 1 | 3 | 312 | 34 | 5.03 | 0.75 |
| 189231244 | anti-fluorescein immunoglobulin heavy chain variable region [Homo sapiens] | 128.41 | 33.61% | 369 | 1 | 3 | 12 | 119 | 12.9 | 8.46 | 0.751 |
| 10835095 | serum amyloid A-4 protein precursor [Homo sapiens] >gi|259352|gb|AAB24060.1| serum amyloid A [Homo sapiens] >gi|337750|gb|AAA60298.1| serum amyloid A protein [Homo sapiens] >gi|13937846|gb|AAH07026.1| Serum amyloid A4, constitutive [Homo sapiens] >gi|49456475|emb|CAG46558.1| SAA4 [Homo sapiens] >gi|119588821|gb|EAW68415.1| serum amyloid A4, constitutive [Homo sapiens] | 94.08 | 23.08% | 2 | 3 | 3 | 18 | 130 | 14.8 | 9.23 | 0.751 |
| 58223278 | anti-tetanus toxoid immunoglobulin light chain variable region [Homo sapiens] | 269.33 | 82.41% | 704 | 1 | 5 | 33 | 108 | 11.5 | 7.96 | 0.752 |
| 553293 | fibronectin [Homo sapiens] | 54.43 | 36.73% | 1 | 1 | 1 | 3 | 49 | 5 | 11.11 | 0.753 |
| 189067450 | unnamed protein product [Homo sapiens] | 26.83 | 2.55% | 1 | 1 | 1 | 4 | 275 | 32.3 | 8.37 | 0.758 |
| 51103589 | immunoglobulin variable region VL lambda domain [Homo sapiens] | 92.76 | 25.00% | 17 | 1 | 2 | 6 | 108 | 11.4 | 4.92 | 0.76 |
| 13937839 | SAA1 protein [Homo sapiens] >gi|123983058|gb|ABM83270.1| serum amyloid A1 [synthetic construct] >gi|123983248|gb|ABM83365.1| serum amyloid A1 [synthetic construct] >gi|123997747|gb|ABM86475.1| serum amyloid A1 [synthetic construct] >gi|157928044|gb|ABW03318.1| serum amyloid A1 [synthetic construct] | 249.56 | 54.10% | 6 | 2 | 5 | 58 | 122 | 13.5 | 6.32 | 0.761 |
| 184086 | histone H2B.1 [Homo sapiens] | 30.74 | 8.91% | 34 | 1 | 1 | 6 | 101 | 11.3 | 10.14 | 0.762 |
| 82734214 | beta-defensin 110 isoform a [Homo sapiens] >gi|84028871|sp|Q30KQ9.1|DB110_HUMAN RecName: Full=Beta-defensin 110; AltName: Full=Defensin, beta 110; AltName: Full=Beta-defensin 10; Short=DEFB-10; AltName: Full=Beta-defensin 111; AltName: Full=Defensin, beta 111; AltName: Full=Beta-defensin 11; Short=DEFB-11; Flags: Precursor >gi|66968904|gb|AAY59751.1| beta-defensin 111 [Homo sapiens] >gi|151555475|gb|AAI48542.1| Defensin, beta 111 [synthetic construct] >gi|162319076|gb|AAI56745.1| Defensin, beta 111 [synthetic construct] | 23.5 | 14.93% | 1 | 1 | 1 | 11 | 67 | 8 | 8.73 | 0.762 |
| 186083 | immunoglobulin lambda-chain [Homo sapiens] | 82.87 | 21.17% | 350 | 1 | 3 | 29 | 137 | 14.3 | 7.24 | 0.762 |
| 247424364 | immunoglobulin heavy chain variable region [Homo sapiens] | 165.61 | 50.81% | 1078 | 1 | 5 | 28 | 124 | 13.4 | 8.91 | 0.762 |
| 194378020 | unnamed protein product [Homo sapiens] | 86.18 | 16.61% | 7 | 3 | 3 | 7 | 289 | 32.1 | 5.85 | 0.764 |
| 587406 | immunoglobulin lambda chain variable region [Homo sapiens] | 78.84 | 27.27% | 244 | 1 | 2 | 16 | 121 | 12.7 | 5.31 | 0.766 |
| 126273569 | carboxypeptidase B2 isoform a preproprotein [Homo sapiens] >gi|62899885|sp|Q96IY4.1|CBPB2_HUMAN RecName: Full=Carboxypeptidase B2; AltName: Full=Carboxypeptidase U; Short=CPU; AltName: Full=Thrombin-activable fibrinolysis inhibitor; Short=TAFI; AltName: Full=Plasma carboxypeptidase B; Short=pCPB; Flags: Precursor >gi|13937897|gb|AAH07057.1| Carboxypeptidase B2 (plasma) [Homo sapiens] >gi|30582711|gb|AAP35582.1| carboxypeptidase B2 (plasma, carboxypeptidase U) [Homo sapiens] >gi|51234145|gb|AAT97987.1| carboxypeptidase B2 (plasma, carboxypeptidase U) [Homo sapiens] >gi|60656513|gb|AAX32820.1| carboxypeptidase B2 [synthetic construct] >gi|60656515|gb|AAX32821.1| carboxypeptidase B2 [synthetic construct] >gi|119629160|gb|EAX08755.1| carboxypeptidase B2 (plasma, carboxypeptidase U), isoform CRA_b [Homo sapiens] | 34.26 | 1.89% | 3 | 1 | 1 | 3 | 423 | 48.4 | 7.71 | 0.769 |
| 194375299 | unnamed protein product [Homo sapiens] | 265.15 | 33.33% | 58 | 8 | 8 | 22 | 333 | 37.3 | 5.71 | 0.771 |
| 158255874 | unnamed protein product [Homo sapiens] | 487.27 | 17.28% | 9 | 12 | 12 | 53 | 1065 | 122.1 | 5.74 | 0.771 |
| 33319096 | Ig heavy chain variable region, VH3 family [Homo sapiens] | 38.9 | 20.97% | 231 | 1 | 2 | 7 | 124 | 13.6 | 7.94 | 0.772 |
| 119610555 | sex hormone-binding globulin, isoform CRA_a [Homo sapiens] | 444.5 | 54.65% | 16 | 11 | 11 | 40 | 344 | 37.5 | 6.23 | 0.773 |
| 40795877 | anti-Toxoplasma gondii SAG1 immunoglobulin light chain variable region [Homo sapiens] | 156.94 | 34.26% | 93 | 2 | 3 | 34 | 108 | 11.7 | 8.47 | 0.78 |
| 70798731 | immunoglobulin kappa light chain variable region [Homo sapiens] | 154.27 | 53.42% | 425 | 1 | 3 | 25 | 73 | 8 | 8.53 | 0.78 |
| 63103087 | anti-rabies virus immunoglobulin light chain variable region [Homo sapiens] | 129.26 | 31.78% | 10 | 1 | 2 | 11 | 107 | 11.5 | 6.54 | 0.782 |
| 180271 | cholesteryl ester transfer protein [Homo sapiens] | 130.3 | 8.00% | 7 | 3 | 3 | 4 | 425 | 47 | 6.01 | 0.782 |
| 118442839 | complement factor H-related protein 1 precursor [Homo sapiens] >gi|239758113|ref|XP_002346300.1| PREDICTED: similar to complement factor H-related 1 isoform 1 [Homo sapiens] >gi|218512041|sp|Q03591.2|FHR1_HUMAN RecName: Full=Complement factor H-related protein 1; Short=FHR-1; AltName: Full=H factor-like protein 1; Short=H-factor-like 1; AltName: Full=H36; Flags: Precursor >gi|183765|gb|AAA35947.1| factor H homologue [Homo sapiens] >gi|5748573|emb|CAB53063.1| complement factor H-related 1 [Homo sapiens] >gi|16876961|gb|AAH16755.1| Complement factor H-related 1 [Homo sapiens] >gi|78070360|gb|AAI07772.1| Complement factor H-related 1 [Homo sapiens] >gi|123984685|gb|ABM83688.1| complement factor H-related 1 [synthetic construct] >gi|123998684|gb|ABM86991.1| complement factor H-related 1 [synthetic construct] | 510.55 | 37.58% | 3 | 1 | 12 | 147 | 330 | 37.6 | 7.39 | 0.785 |
| 1064908 | complement Factor H-related Protein 2 [Homo sapiens] | 222.48 | 31.28% | 3 | 3 | 6 | 38 | 243 | 27.9 | 6.92 | 0.788 |
| 189067487 | unnamed protein product [Homo sapiens] | 34.33 | 3.61% | 2 | 1 | 1 | 1 | 527 | 59.7 | 7.24 | 0.789 |
| 119625338 | fibrinogen beta chain, isoform CRA_d [Homo sapiens] | 1669.35 | 67.61% | 7 | 24 | 34 | 2036 | 457 | 52.1 | 8.07 | 0.791 |
| 119625314 | fibrinogen gamma chain, isoform CRA_e [Homo sapiens] | 1193.9 | 62.53% | 5 | 6 | 27 | 1576 | 419 | 47.4 | 5.95 | 0.798 |
| 105990532 | apolipoprotein B-100 precursor [Homo sapiens] >gi|260158878|gb|ACX32319.1| apolipoprotein B precursor [synthetic construct] | 2158.39 | 18.58% | 36 | 5 | 63 | 209 | 4563 | 515.2 | 7.05 | 0.799 |
| 34530477 | unnamed protein product [Homo sapiens] | 82.31 | 15.31% | 6 | 2 | 2 | 2 | 196 | 20.8 | 8.4 | 0.799 |
| 193784716 | unnamed protein product [Homo sapiens] | 58.37 | 6.86% | 1 | 3 | 3 | 3 | 423 | 47.7 | 5.52 | 0.804 |
| 119609949 | lectin, galactoside-binding, soluble, 3 binding protein, isoform CRA_a [Homo sapiens] | 513.78 | 31.50% | 9 | 12 | 12 | 43 | 581 | 64.8 | 5.34 | 0.809 |
| 178741 | apolipoprotein C-II [Homo sapiens] >gi|357629|prf||1303321A apolipoprotein CII | 353.8 | 65.93% | 6 | 6 | 6 | 187 | 91 | 10.2 | 4.56 | 0.81 |
| 221042312 | unnamed protein product [Homo sapiens] | 233.94 | 15.07% | 15 | 4 | 4 | 12 | 564 | 60 | 5.74 | 0.811 |
| 119588814 | serum amyloid A1, isoform CRA_a [Homo sapiens] >gi|119588815|gb|EAW68409.1| serum amyloid A1, isoform CRA_a [Homo sapiens] >gi|119588817|gb|EAW68411.1| serum amyloid A1, isoform CRA_a [Homo sapiens] | 350.08 | 54.10% | 9 | 3 | 6 | 71 | 122 | 13.6 | 6.79 | 0.813 |
| 84798296 | immunoglobulin light chain variable region YV1-4-K4-3 [Homo sapiens] | 58.21 | 23.68% | 4 | 1 | 2 | 10 | 114 | 12.5 | 5.36 | 0.816 |
| 183763 | factor H homologue [Homo sapiens] >gi|158255096|dbj|BAF83519.1| unnamed protein product [Homo sapiens] | 511 | 37.58% | 4 | 1 | 12 | 142 | 330 | 37.6 | 7.56 | 0.824 |
| 226958414 | isovaleryl-CoA dehydrogenase, mitochondrial isoform 2 precursor [Homo sapiens] >gi|119612820|gb|EAW92414.1| isovaleryl Coenzyme A dehydrogenase, isoform CRA_b [Homo sapiens] >gi|193783817|dbj|BAG53799.1| unnamed protein product [Homo sapiens] | 25.55 | 2.27% | 4 | 1 | 1 | 1 | 396 | 43 | 7.66 | 0.825 |
| 115298678 | complement C3 precursor [Homo sapiens] >gi|119370332|sp|P01024.2|CO3_HUMAN RecName: Full=Complement C3; AltName: Full=C3 and PZP-like alpha-2-macroglobulin domain-containing protein 1; Contains: RecName: Full=Complement C3 beta chain; Contains: RecName: Full=Complement C3 alpha chain; Contains: RecName: Full=C3a anaphylatoxin; Contains: RecName: Full=Complement C3b alpha' chain; Contains: RecName: Full=Complement C3c alpha' chain fragment 1; Contains: RecName: Full=Complement C3dg fragment; Contains: RecName: Full=Complement C3g fragment; Contains: RecName: Full=Complement C3d fragment; Contains: RecName: Full=Complement C3f fragment; Contains: RecName: Full=Complement C3c alpha' chain fragment 2; Flags: Precursor >gi|40786791|gb|AAR89906.1| complement component 3 [Homo sapiens] >gi|119589477|gb|EAW69071.1| complement component 3, isoform CRA_b [Homo sapiens] >gi|152012494|gb|AAI50201.1| Complement component 3 [Homo sapiens] >gi|152012784|gb|AAI50180.1| Complement component 3 [Homo sapiens] | 3018.01 | 58.15% | 7 | 76 | 76 | 444 | 1663 | 187 | 6.4 | 0.829 |
| 189053554 | unnamed protein product [Homo sapiens] | 125.23 | 20.08% | 2 | 3 | 3 | 23 | 244 | 26.4 | 5.74 | 0.829 |
| 31874240 | hypothetical protein [Homo sapiens] | 1112.51 | 62.16% | 23 | 1 | 27 | 1104 | 399 | 45.1 | 6.16 | 0.832 |
| 33319680 | Ig heavy chain variable region, VH3 family [Homo sapiens] | 172.21 | 39.34% | 1007 | 1 | 4 | 29 | 122 | 13.1 | 8.85 | 1.202 |
| 1620018 | heat shock protein 90 [Homo sapiens] | 69.98 | 17.88% | 10 | 1 | 1 | 4 | 151 | 16.8 | 4.79 | 1.205 |
| 221042196 | unnamed protein product [Homo sapiens] | 99.51 | 6.36% | 8 | 3 | 3 | 14 | 645 | 72.1 | 7.68 | 1.205 |
| 119579722 | complement factor properdin, isoform CRA_b [Homo sapiens] | 25.72 | 3.82% | 6 | 1 | 1 | 2 | 340 | 37.3 | 8.02 | 1.206 |
| 20377087 | intestinal lactoferrin receptor [Homo sapiens] >gi|7019846|dbj|BAA90893.1| unnamed protein product [Homo sapiens] >gi|8096221|dbj|BAA96094.1| intelectin [Homo sapiens] >gi|18091783|gb|AAL58073.1| endothelial lectin HL-1 [Homo sapiens] >gi|37181843|gb|AAQ88725.1| ITLN [Homo sapiens] >gi|37724012|gb|AAO17801.1| intelectin [Homo sapiens] >gi|44976129|gb|AAS49907.1| omentin [Homo sapiens] >gi|48146565|emb|CAG33505.1| ITLN1 [Homo sapiens] >gi|52843233|gb|AAU88047.1| intelectin 1 [Homo sapiens] >gi|62896625|dbj|BAD96253.1| intelectin variant [Homo sapiens] | 171.75 | 29.07% | 7 | 7 | 7 | 13 | 313 | 35 | 5.82 | 1.212 |
| 224979536 | anti-IL-15 immunoglobulin heavy chain variable region 1 [Homo sapiens] | 21.9 | 16.10% | 1 | 1 | 1 | 2 | 118 | 12.7 | 7.12 | 1.216 |
| 21669331 | immunoglobulin kappa light chain VLJ region [Homo sapiens] | 445.13 | 40.00% | 420 | 2 | 7 | 238 | 270 | 28.8 | 7.69 | 1.217 |
| 156765940 | collectin kidney K1 [Homo sapiens] | 260.61 | 27.41% | 26 | 7 | 7 | 33 | 197 | 21.6 | 4.75 | 1.218 |
| 5174411 | CD5 antigen-like precursor [Homo sapiens] >gi|20177834|sp|O43866.1|CD5L_HUMAN RecName: Full=CD5 antigen-like; AltName: Full=SP-alpha; AltName: Full=CT-2; AltName: Full=IgM-associated peptide; Flags: Precursor >gi|2702314|gb|AAB91989.1| Sp alpha [Homo sapiens] >gi|4102235|gb|AAD01446.1| AIM [Homo sapiens] >gi|11967471|emb|CAC19458.1| CD5 molecule-like [Homo sapiens] >gi|21707924|gb|AAH33586.1| CD5 molecule-like [Homo sapiens] >gi|119573244|gb|EAW52859.1| CD5 antigen-like (scavenger receptor cysteine rich family) [Homo sapiens] >gi|123993879|gb|ABM84541.1| CD5 molecule-like [synthetic construct] >gi|123997245|gb|ABM86224.1| CD5 molecule-like [synthetic construct] >gi|158257512|dbj|BAF84729.1| unnamed protein product [Homo sapiens] | 466.86 | 47.84% | 2 | 14 | 14 | 57 | 347 | 38.1 | 5.47 | 1.229 |
| 21669521 | immunoglobulin lambda light chain VLJ region [Homo sapiens] | 493.89 | 40.52% | 420 | 1 | 11 | 747 | 269 | 27.9 | 7.91 | 1.232 |
| 70888104 | immunoglobulin lambda light chain variable region [Homo sapiens] | 183.7 | 55.56% | 47 | 1 | 4 | 39 | 108 | 11.6 | 4.65 | 1.232 |
| 54780728 | immunoglobulin mu heavy chain [Homo sapiens] | 318.62 | 23.75% | 2295 | 1 | 7 | 196 | 240 | 26.4 | 8.32 | 1.25 |
| 34534492 | unnamed protein product [Homo sapiens] | 13.39 | 8.66% | 1 | 1 | 1 | 1 | 277 | 30.5 | 8.56 | 1.254 |
| 194273292 | immunoglobulin heavy chain variable region [Homo sapiens] | 227.62 | 70.30% | 896 | 1 | 6 | 13 | 101 | 11 | 9.72 | 1.26 |
| 4758146 | neutrophil defensin 1 preproprotein [Homo sapiens] >gi|124248516|ref|NP_001035965.1| alpha-defensin 1 [Homo sapiens] >gi|30316322|sp|P59665.1|DEF1_HUMAN RecName: Full=Neutrophil defensin 1; AltName: Full=HNP-1; Short=HP-1; Short=HP1; AltName: Full=Defensin, alpha 1; Contains: RecName: Full=HP 1-56; Contains: RecName: Full=Neutrophil defensin 2; AltName: Full=HNP-2; Short=HP-2; Short=HP2; Flags: Precursor >gi|32402|emb|CAA36280.1| unnamed protein product [Homo sapiens] >gi|181527|gb|AAA52302.1| neutrophil peptide 1 precursor [Homo sapiens] >gi|181529|gb|AAA52303.1| defensin 1 [Homo sapiens] >gi|292363|gb|AAA36382.1| neutrophil peptide-1 [Homo sapiens] >gi|46854592|gb|AAH69423.1| Defensin, alpha 1 [Homo sapiens] >gi|50057839|gb|AAT68875.1| novel protein, similar to DEFA1 [Homo sapiens] >gi|50057842|gb|AAT68878.1| defensin, alpha 1, myeloid-related sequence [Homo sapiens] >gi|50057843|gb|AAT68879.1| novel protein, similar to DEFA1 [Homo sapiens] >gi|50057844|gb|AAT68880.1| novel protein, similar to DEFA1 [Homo sapiens] >gi|50057847|gb|AAT68883.1| defensin, alpha 1, myeloid-related sequence [Homo sapiens] >gi|50057848|gb|AAT68884.1| defensin, alpha 1, myeloid-related sequence [Homo sapiens] >gi|62739977|gb|AAH93791.1| Defensin, alpha 1 [Homo sapiens] >gi|85567619|gb|AAI12189.1| Defensin, alpha 1, preproprotein [Homo sapiens] >gi|446635|prf||1912193A defensin >gi|1098031|prf||2115200A neutrophil peptide | 43.25 | 32.98% | 2 | 1 | 1 | 2 | 94 | 10.2 | 6.99 | 1.263 |
| 27373753 | apolipoprotein J [Homo sapiens] | 220.42 | 55.26% | 1 | 1 | 5 | 87 | 76 | 9.1 | 8.15 | 1.275 |
| 194391084 | unnamed protein product [Homo sapiens] | 617.63 | 41.93% | 9 | 18 | 18 | 100 | 415 | 46.5 | 6.43 | 1.278 |
| 40737478 | C4A3 [Homo sapiens] >gi|40737480|gb|AAR89158.1| C4A [Homo sapiens] >gi|40737484|gb|AAR89160.1| C4A3 [Homo sapiens] | 933.23 | 54.49% | 13 | 1 | 21 | 260 | 534 | 58.4 | 5.99 | 1.282 |
| 4557871 | serotransferrin precursor [Homo sapiens] >gi|136191|sp|P02787.2|TRFE_HUMAN RecName: Full=Serotransferrin; Short=Transferrin; AltName: Full=Siderophilin; AltName: Full=Beta-1 metal-binding globulin; Flags: Precursor >gi|248648|gb|AAB22049.1| transferrin [Homo sapiens] >gi|339453|gb|AAA61140.1| transferrin precursor [Homo sapiens] >gi|15021381|gb|AAK77664.1| transferin [Homo sapiens] >gi|31415705|gb|AAP45055.1| transferrin [Homo sapiens] >gi|94717618|gb|ABF47110.1| transferrin [Homo sapiens] >gi|119599573|gb|EAW79167.1| transferrin, isoform CRA_d [Homo sapiens] | 635.11 | 29.94% | 18 | 15 | 15 | 60 | 698 | 77 | 7.12 | 1.284 |
| 1082521 | Ig kappa chain V region - human (fragment) >gi|809555|emb|CAA58108.1| immunoglobulin kappa light chain [Homo sapiens] | 87.06 | 36.84% | 16 | 1 | 3 | 3 | 114 | 12.3 | 8.94 | 1.286 |
| 681900 | This CDS feature is included to show the translation of the corresponding V_region. Presently translation qualifiers on V_region features are illegal [Homo sapiens] | 138.54 | 44.86% | 293 | 1 | 3 | 9 | 107 | 11.6 | 8.51 | 1.289 |
| 54779153 | immunoglobulin mu heavy chain [Homo sapiens] | 192.36 | 29.67% | 735 | 1 | 5 | 64 | 209 | 22.6 | 7.68 | 1.294 |
| 6643569 | immunoglobulin lambda light chain variable region [Homo sapiens] >gi|6643571|gb|AAF20644.1| immunoglobulin lambda light chain variable region [Homo sapiens] | 46.99 | 25.93% | 193 | 1 | 2 | 2 | 108 | 11.5 | 5.96 | 1.301 |
| 77379428 | immunoglobulin kappa chain variable region [Homo sapiens] | 38.02 | 21.49% | 1 | 1 | 1 | 2 | 121 | 13.1 | 6 | 1.301 |
| 110626504 | anti-SARS-CoV S protein immunoglobulin kappa light chain [Homo sapiens] | 505.36 | 43.93% | 58 | 1 | 8 | 576 | 214 | 23.6 | 7.71 | 1.304 |
| 7770217 | PRO2675 [Homo sapiens] >gi|119626082|gb|EAX05677.1| albumin, isoform CRA_s [Homo sapiens] | 939.35 | 78.47% | 5 | 1 | 22 | 318 | 288 | 32.6 | 6.54 | 1.311 |
| 86438992 | immunoglobulin heavy chain [Homo sapiens] | 128.3 | 28.45% | 838 | 1 | 3 | 26 | 116 | 12.8 | 7.97 | 1.312 |
| 21619848 | IGL@ protein [Homo sapiens] | 440.88 | 46.81% | 109 | 1 | 11 | 570 | 235 | 24.8 | 7.71 | 1.32 |
| 21669523 | immunoglobulin lambda light chain VLJ region [Homo sapiens] | 427.3 | 39.63% | 106 | 1 | 10 | 568 | 270 | 28.1 | 7.14 | 1.33 |
| 194383694 | unnamed protein product [Homo sapiens] | 112.48 | 6.53% | 10 | 3 | 3 | 10 | 704 | 77.2 | 4.53 | 1.331 |
| 28559048 | Ig rearranged L-chain V-region [Homo sapiens] | 51.37 | 14.37% | 243 | 1 | 2 | 14 | 167 | 17.3 | 5.35 | 1.331 |
| 21628665 | immunoglobulin light chain constant region [Homo sapiens] | 116.72 | 91.43% | 1 | 1 | 3 | 82 | 35 | 3.9 | 6.48 | 1.334 |
| 109240688 | immunoglobulin kappa chain variable region [Homo sapiens] | 41.25 | 15.09% | 2 | 1 | 1 | 3 | 106 | 11.7 | 7.99 | 1.335 |
| 170684604 | immunoglobulin lambda 3 light chain [Homo sapiens] | 327.61 | 37.80% | 10 | 1 | 7 | 650 | 209 | 22.3 | 5.95 | 1.338 |
| 1369904 | serum lectin P35 [Homo sapiens] >gi|1669349|dbj|BAA09636.1| lectin P35 [Homo sapiens] | 342.33 | 30.99% | 6 | 8 | 9 | 95 | 313 | 34 | 6.54 | 1.338 |
| 12733966 | immunoglobulin gamma heavy chain variable region [Homo sapiens] | 167.4 | 39.32% | 1183 | 1 | 4 | 41 | 117 | 13 | 6.04 | 1.361 |
| 567112 | OMM protein (Ig gamma3) heavy chain [Homo sapiens] | 307.84 | 28.75% | 3 | 1 | 7 | 91 | 313 | 34.9 | 7.49 | 1.361 |
| 31337558 | immunoglobulin gamma heavy chain variable region [Homo sapiens] | 125.51 | 29.37% | 842 | 1 | 3 | 26 | 126 | 14.2 | 6.62 | 1.361 |
| 497334 | immunoglobulin lambda chain variable region [Homo sapiens] | 17.71 | 16.84% | 2 | 1 | 1 | 1 | 95 | 10 | 4.88 | 1.367 |
| 190194 | serum paraoxonase [Homo sapiens] | 414.48 | 45.70% | 15 | 1 | 10 | 71 | 337 | 37.8 | 5.08 | 1.372 |
| 77379876 | immunoglobulin lambda chain variable region [Homo sapiens] | 75.33 | 12.70% | 83 | 1 | 2 | 10 | 126 | 13.1 | 8.44 | 1.378 |
| 37789717 | immunoglobulin lambda light chain variable region [Homo sapiens] | 37.12 | 9.09% | 4 | 1 | 1 | 3 | 88 | 9.5 | 5.17 | 1.383 |
| 194390508 | unnamed protein product [Homo sapiens] | 1308.75 | 40.93% | 24 | 1 | 25 | 226 | 1014 | 111.2 | 6.21 | 1.387 |
| 27369048 | immunoglobulin lambda light chain variable region [Homo sapiens] | 10.42 | 9.29% | 1 | 1 | 1 | 1 | 140 | 14.5 | 7.06 | 1.388 |
| 221044282 | unnamed protein product [Homo sapiens] | 229.04 | 9.77% | 4 | 5 | 5 | 15 | 870 | 95.9 | 4.64 | 1.39 |
| 532598 | Ig J-chain [Homo sapiens] | 192.26 | 45.26% | 3 | 3 | 3 | 21 | 137 | 15.6 | 4.73 | 1.405 |
| 33319656 | Ig heavy chain variable region, VH3 family [Homo sapiens] | 122.03 | 30.83% | 763 | 1 | 3 | 26 | 120 | 13.1 | 7.14 | 1.407 |
| 54780200 | immunoglobulin mu heavy chain [Homo sapiens] | 270.49 | 33.18% | 525 | 1 | 5 | 66 | 211 | 22.7 | 8.29 | 1.415 |
| 21669509 | immunoglobulin lambda light chain VLJ region [Homo sapiens] | 302.26 | 34.43% | 250 | 1 | 7 | 501 | 273 | 28.2 | 7.14 | 1.416 |
| 87299014 | immunoglobulin light chain variable region [Homo sapiens] | 454.36 | 71.05% | 348 | 1 | 9 | 514 | 152 | 16.1 | 4.93 | 1.42 |
| 55957679 | cartilage acidic protein 1 [Homo sapiens] >gi|55958790|emb|CAI14275.1| cartilage acidic protein 1 [Homo sapiens] | 68.51 | 5.34% | 10 | 3 | 3 | 5 | 524 | 56.5 | 5.55 | 1.42 |
| 215982760 | immunoglobulin lambda light chain variable region [Homo sapiens] | 150.34 | 27.66% | 13 | 1 | 3 | 214 | 141 | 15.1 | 5.45 | 1.43 |
| 54780230 | immunoglobulin mu heavy chain [Homo sapiens] | 161.81 | 19.43% | 633 | 1 | 4 | 89 | 211 | 22.7 | 8.31 | 1.45 |
| 17226634 | immunoglobulin heavy chain variable region [Homo sapiens] | 99.44 | 24.39% | 225 | 1 | 3 | 8 | 123 | 13.4 | 7.83 | 1.455 |
| 33319650 | Ig heavy chain variable region, VH3 family [Homo sapiens] | 100.27 | 34.17% | 151 | 1 | 3 | 5 | 120 | 13.1 | 6.52 | 1.479 |
| 4321593 | immunoglobulin G kappa chain [Homo sapiens] | 361.42 | 38.25% | 71 | 1 | 5 | 126 | 217 | 24 | 6.55 | 1.486 |
| 7705753 | complement C1q subcomponent subunit A precursor [Homo sapiens] >gi|399138|sp|P02745.2|C1QA_HUMAN RecName: Full=Complement C1q subcomponent subunit A; Flags: Precursor >gi|4894854|gb|AAD32626.1|AF135157_1 complement C1q A chain precursor [Homo sapiens] >gi|20988805|gb|AAH30153.1| Complement component 1, q subcomponent, A chain [Homo sapiens] >gi|48734785|gb|AAH71986.1| Complement component 1, q subcomponent, A chain [Homo sapiens] >gi|54781353|gb|AAV40828.1| complement component 1, q subcomponent, alpha polypeptide [Homo sapiens] >gi|56205031|emb|CAI22893.1| complement component 1, q subcomponent, A chain [Homo sapiens] >gi|119615420|gb|EAW95014.1| complement component 1, q subcomponent, A chain, isoform CRA_a [Homo sapiens] >gi|119615421|gb|EAW95015.1| complement component 1, q subcomponent, A chain, isoform CRA_a [Homo sapiens] >gi|189065196|dbj|BAG34919.1| unnamed protein product [Homo sapiens] >gi|190692105|gb|ACE87827.1| complement component 1, q subcomponent, A chain protein [synthetic construct] >gi|254071643|gb|ACT64581.1| complement component 1, q subcomponent, A chain protein [synthetic construct] | 59.13 | 20.41% | 1 | 2 | 2 | 5 | 245 | 26 | 9.11 | 1.492 |
| 114147277 | immunoglobulin heavy chain variable region [Homo sapiens] | 74.17 | 22.73% | 41 | 1 | 2 | 12 | 110 | 12.3 | 8.29 | 1.518 |
| 18041890 | immunoglobulin lambda light chain variable region [Homo sapiens] | 35.59 | 9.09% | 3 | 1 | 1 | 7 | 88 | 9.2 | 5.24 | 1.525 |
| 55669575 | Chain A, A Covalent Dimer Of Transthyretin That Affects The Amyloid Pathway >gi|55669576|pdb|1QWH|B Chain B, A Covalent Dimer Of Transthyretin That Affects The Amyloid Pathway >gi|339685|gb|AAA61181.1| transthyretin [Homo sapiens] | 291.36 | 66.67% | 11 | 7 | 7 | 86 | 117 | 12.8 | 5.45 | 1.543 |
| 553490 | immunoglobulin lambda-chain [Homo sapiens] | 64.73 | 12.50% | 89 | 1 | 2 | 7 | 128 | 13.4 | 7.81 | 1.55 |
| 11558186 | immunoglobulin light chain variable region [Homo sapiens] | 33.94 | 6.06% | 1 | 1 | 1 | 2 | 132 | 13.7 | 8.94 | 1.569 |
| 157778872 | immunoglobulin heavy chain variable region [Homo sapiens] | 45.74 | 17.39% | 231 | 1 | 2 | 7 | 115 | 12.8 | 9.01 | 1.58 |
| 3004786 | Ig heavy chain variable region [Homo sapiens] | 109.28 | 29.66% | 145 | 1 | 3 | 19 | 118 | 13.1 | 7.97 | 1.656 |
| 4960066 | apolipoprotein A1 [Homo sapiens] | 352.13 | 79.10% | 83 | 1 | 9 | 252 | 67 | 7.4 | 6.95 | 1.689 |
| 1620396 | haptoglobin [Homo sapiens] | 370.62 | 34.77% | 7 | 4 | 11 | 42 | 348 | 39 | 6.89 | 1.7 |
| 1905799 | immunogloblin light chain [Homo sapiens] | 197.3 | 44.95% | 242 | 1 | 3 | 27 | 109 | 11.7 | 7.28 | 1.712 |
| 37694587 | immunoglobulin heavy chain variable region [Homo sapiens] | 81.8 | 25.64% | 12 | 1 | 2 | 5 | 117 | 12.9 | 7.39 | 1.733 |
| 247424903 | immunoglobulin heavy chain variable region [Homo sapiens] | 286.5 | 51.22% | 1252 | 1 | 5 | 69 | 123 | 13.4 | 7.96 | 1.737 |
| 58222449 | anti-tetanus toxoid immunoglobulin heavy chain variable region [Homo sapiens] | 102.65 | 21.49% | 133 | 1 | 3 | 20 | 121 | 13.2 | 8.46 | 1.783 |
| 587350 | immunoglobulin kappa light chain variable region [Homo sapiens] | 67.14 | 29.91% | 10 | 1 | 3 | 5 | 117 | 12.6 | 7.12 | 1.786 |
| 11122875 | glycosylphosphatidylinositol phospholipase D [Homo sapiens] | 137.2 | 8.32% | 8 | 3 | 3 | 4 | 457 | 48.5 | 6.2 | 1.804 |
| 247425451 | immunoglobulin heavy chain variable region [Homo sapiens] | 125.32 | 24.59% | 743 | 1 | 2 | 24 | 122 | 13.1 | 6.57 | 1.836 |
| 8777875 | immunoglobulin light chain variable region [Homo sapiens] | 174.72 | 37.61% | 423 | 1 | 3 | 26 | 109 | 12 | 8.48 | 1.924 |
| 7012705 | immunoglobulin light chain variable region [Homo sapiens] | 212.91 | 68.22% | 425 | 1 | 4 | 27 | 107 | 11.5 | 7.96 | 2.029 |
| 33319586 | Ig heavy chain variable region, VH3 family [Homo sapiens] | 125.55 | 25.00% | 742 | 1 | 2 | 23 | 120 | 13 | 4.77 | 2.156 |
| 2253348 | immunoglobulin heavy chain variable region [Homo sapiens] | 69.15 | 22.13% | 31 | 1 | 2 | 3 | 122 | 13.4 | 9.07 | 2.16 |
| 4467842 | immunoglobulin M heavy chain [Homo sapiens] | 1329.03 | 66.45% | 17 | 1 | 25 | 1256 | 453 | 49.4 | 6.77 | 2.2 |
| 119600101 | phosphatidylinositol-specific phospholipase C, X domain containing 2 [Homo sapiens] | 34.44 | 2.42% | 3 | 1 | 1 | 1 | 289 | 33 | 9.44 | 2.256 |
| 247425006 | immunoglobulin heavy chain variable region [Homo sapiens] | 179.48 | 39.34% | 421 | 1 | 4 | 50 | 122 | 13.1 | 8.81 | 2.331 |
| 346196 | Ig lambda chain V region - human >gi|452933|gb|AAB28790.1| anti-cardiolipin/beta 2 glycoprotein I immunoglobulin light chain variable region [Homo sapiens] | 102.42 | 21.67% | 284 | 1 | 3 | 22 | 120 | 12.4 | 6.48 | 2.575 |
| 18092610 | anti-cardiolipin immunoglobulin light chain [Homo sapiens] | 115.07 | 26.85% | 23 | 1 | 2 | 27 | 108 | 12 | 8.63 | 2.653 |
| 221042228 | unnamed protein product [Homo sapiens] | 15.94 | 2.78% | 1 | 1 | 1 | 1 | 539 | 61.3 | 7.74 | 5.108 |

**Mix-PH vs. control**

| Accession | Description | Score | Coverage | # Proteins | # Unique Peptides | # Peptides | # PSMs | # AAs | MW [kDa] | calc. pI | Mix-PH/Control |
| --- | --- | --- | --- | --- | --- | --- | --- | --- | --- | --- | --- |
| 21669937 | immunoglobulin heavy chain VHDJ region [Homo sapiens] | 114.57 | 18.55% | 423 | 1 | 2 | 11 | 124 | 13.5 | 9.36 | 0.254 |
| 13195586 | hemoglobin alpha 1 globin chain [Homo sapiens] | 103.91 | 24.00% | 13 | 2 | 2 | 25 | 100 | 10.7 | 7.72 | 0.295 |
| 36321 | SAA precursor polypeptide (119 AA) [Homo sapiens] | 245.07 | 52.10% | 10 | 2 | 5 | 45 | 119 | 13.1 | 8.18 | 0.304 |
| 124504316 | HIST2H4B protein [Homo sapiens] | 46.54 | 9.80% | 6 | 1 | 1 | 1 | 102 | 11.4 | 11.36 | 0.312 |
| 194375974 | unnamed protein product [Homo sapiens] | 60.43 | 9.84% | 5 | 2 | 2 | 3 | 183 | 20.1 | 8.78 | 0.351 |
| 13937839 | SAA1 protein [Homo sapiens] >gi|123983058|gb|ABM83270.1| serum amyloid A1 [synthetic construct] >gi|123983248|gb|ABM83365.1| serum amyloid A1 [synthetic construct] >gi|123997747|gb|ABM86475.1| serum amyloid A1 [synthetic construct] >gi|157928044|gb|ABW03318.1| serum amyloid A1 [synthetic construct] | 249.56 | 54.10% | 6 | 2 | 5 | 58 | 122 | 13.5 | 6.32 | 0.375 |
| 56378229 | carbamoylphosphate synthetase I [Homo sapiens] | 22.22 | 4.09% | 12 | 1 | 1 | 1 | 513 | 55.7 | 8.94 | 0.399 |
| 146424184 | apolipoprotein C-IV [Homo sapiens] | 73.46 | 15.75% | 3 | 2 | 2 | 5 | 127 | 14.6 | 9.13 | 0.403 |
| 186083 | immunoglobulin lambda-chain [Homo sapiens] | 82.87 | 21.17% | 350 | 1 | 3 | 29 | 137 | 14.3 | 7.24 | 0.427 |
| 10835095 | serum amyloid A-4 protein precursor [Homo sapiens] >gi|259352|gb|AAB24060.1| serum amyloid A [Homo sapiens] >gi|337750|gb|AAA60298.1| serum amyloid A protein [Homo sapiens] >gi|13937846|gb|AAH07026.1| Serum amyloid A4, constitutive [Homo sapiens] >gi|49456475|emb|CAG46558.1| SAA4 [Homo sapiens] >gi|119588821|gb|EAW68415.1| serum amyloid A4, constitutive [Homo sapiens] | 94.08 | 23.08% | 2 | 3 | 3 | 18 | 130 | 14.8 | 9.23 | 0.439 |
| 189231244 | anti-fluorescein immunoglobulin heavy chain variable region [Homo sapiens] | 128.41 | 33.61% | 369 | 1 | 3 | 12 | 119 | 12.9 | 8.46 | 0.462 |
| 118406205 | immunoglobulin heavy chain variable region [Homo sapiens] | 58.92 | 22.22% | 231 | 1 | 2 | 7 | 117 | 12.5 | 8.68 | 0.474 |
| 55958543 | heterogeneous nuclear ribonucleoprotein K [Homo sapiens] | 39.26 | 5.23% | 12 | 1 | 1 | 1 | 306 | 34 | 5.78 | 0.508 |
| 119588814 | serum amyloid A1, isoform CRA_a [Homo sapiens] >gi|119588815|gb|EAW68409.1| serum amyloid A1, isoform CRA_a [Homo sapiens] >gi|119588817|gb|EAW68411.1| serum amyloid A1, isoform CRA_a [Homo sapiens] | 350.08 | 54.10% | 9 | 3 | 6 | 71 | 122 | 13.6 | 6.79 | 0.53 |
| 3152372 | anti-FactorVIII scFv [Homo sapiens] | 537.51 | 64.29% | 3758 | 1 | 10 | 90 | 238 | 25 | 8.41 | 0.538 |
| 4505733 | platelet factor 4 precursor [Homo sapiens] >gi|130304|sp|P02776.2|PLF4_HUMAN RecName: Full=Platelet factor 4; Short=PF-4; AltName: Full=C-X-C motif chemokine 4; AltName: Full=Oncostatin-A; AltName: Full=Iroplact; Contains: RecName: Full=Platelet factor 4, short form; Flags: Precursor >gi|13549118|gb|AAK29643.1|AF349466_3 platelet factor 4 [Homo sapiens] >gi|189851|gb|AAA60066.1| platelet factor 4 [Homo sapiens] >gi|47115291|emb|CAG28605.1| PF4 [Homo sapiens] >gi|62739642|gb|AAH93965.1| Platelet factor 4 [Homo sapiens] >gi|63994325|gb|AAY41003.1| unknown [Homo sapiens] >gi|85567544|gb|AAI12094.1| Platelet factor 4 [Homo sapiens] >gi|119626099|gb|EAX05694.1| platelet factor 4 (chemokine (C-X-C motif) ligand 4) [Homo sapiens] >gi|261859268|dbj|BAI46156.1| platelet factor 4 [synthetic construct] | 125.57 | 34.65% | 1 | 1 | 3 | 22 | 101 | 10.8 | 8.62 | 0.548 |
| 180271 | cholesteryl ester transfer protein [Homo sapiens] | 130.3 | 8.00% | 7 | 3 | 3 | 4 | 425 | 47 | 6.01 | 0.552 |
| 226958414 | isovaleryl-CoA dehydrogenase, mitochondrial isoform 2 precursor [Homo sapiens] >gi|119612820|gb|EAW92414.1| isovaleryl Coenzyme A dehydrogenase, isoform CRA_b [Homo sapiens] >gi|193783817|dbj|BAG53799.1| unnamed protein product [Homo sapiens] | 25.55 | 2.27% | 4 | 1 | 1 | 1 | 396 | 43 | 7.66 | 0.556 |
| 119573007 | apolipoprotein A-II, isoform CRA_d [Homo sapiens] | 478.54 | 75.00% | 4 | 2 | 9 | 695 | 92 | 10.6 | 9.04 | 0.571 |
| 32483410 | vitamin D-binding protein precursor [Homo sapiens] | 803.24 | 58.86% | 3 | 1 | 22 | 75 | 474 | 52.9 | 5.45 | 0.577 |
| 178812 | apolipoprotein B-100 precursor [Homo sapiens] | 1998.27 | 17.34% | 21 | 1 | 59 | 189 | 4563 | 515.1 | 7.11 | 0.579 |
| 76252669 | immunoglobulin lambda light chain variable region [Homo sapiens] | 33.83 | 17.65% | 2 | 1 | 1 | 4 | 102 | 10.9 | 7.12 | 0.579 |
| 54780362 | immunoglobulin mu heavy chain [Homo sapiens] | 241.84 | 26.44% | 2119 | 1 | 5 | 94 | 208 | 22.6 | 7.77 | 0.58 |
| 119594857 | cofilin 1 (non-muscle), isoform CRA_c [Homo sapiens] | 91.11 | 40.88% | 6 | 4 | 4 | 5 | 137 | 15.6 | 8.35 | 0.588 |
| 54304028 | glyceraldehyde-3-phosphate dehydrogenase [Homo sapiens] | 45.44 | 17.44% | 7 | 1 | 1 | 3 | 86 | 9.2 | 9.72 | 0.603 |
| 169672524 | transforming growth factor beta 1 precursor [Homo sapiens] | 51.7 | 16.10% | 7 | 1 | 1 | 1 | 118 | 12.9 | 7.2 | 0.605 |
| 268374656 | immunoglobulin E heavy chain variable region [Homo sapiens] | 117.96 | 42.57% | 956 | 1 | 3 | 24 | 101 | 11.6 | 6.58 | 0.62 |
| 34532317 | unnamed protein product [Homo sapiens] | 19.57 | 0.89% | 9 | 1 | 1 | 1 | 1014 | 113.4 | 9.03 | 0.627 |
| 18418633 | mutant beta-globin [Homo sapiens] | 289.03 | 71.43% | 76 | 7 | 8 | 59 | 147 | 16 | 7.69 | 0.628 |
| 105990532 | apolipoprotein B-100 precursor [Homo sapiens] >gi|260158878|gb|ACX32319.1| apolipoprotein B precursor [synthetic construct] | 2158.39 | 18.58% | 36 | 5 | 63 | 209 | 4563 | 515.2 | 7.05 | 0.628 |
| 247425006 | immunoglobulin heavy chain variable region [Homo sapiens] | 179.48 | 39.34% | 421 | 1 | 4 | 50 | 122 | 13.1 | 8.81 | 0.629 |
| 221042312 | unnamed protein product [Homo sapiens] | 233.94 | 15.07% | 15 | 4 | 4 | 12 | 564 | 60 | 5.74 | 0.632 |
| 2809025 | Ig heavy chain variable region [Homo sapiens] | 41.55 | 42.25% | 134 | 1 | 2 | 4 | 71 | 7.9 | 9.31 | 0.633 |
| 119626442 | multimerin 1, isoform CRA_a [Homo sapiens] | 62.55 | 3.58% | 6 | 1 | 1 | 5 | 531 | 58.1 | 8.72 | 0.639 |
| 31873302 | hypothetical protein [Homo sapiens] >gi|117646030|emb|CAL38482.1| hypothetical protein [synthetic construct] | 241.85 | 24.19% | 27 | 7 | 7 | 15 | 434 | 47.1 | 7.69 | 0.64 |
| 1064908 | complement Factor H-related Protein 2 [Homo sapiens] | 222.48 | 31.28% | 3 | 3 | 6 | 38 | 243 | 27.9 | 6.92 | 0.641 |
| 31874240 | hypothetical protein [Homo sapiens] | 1112.51 | 62.16% | 23 | 1 | 27 | 1104 | 399 | 45.1 | 6.16 | 0.643 |
| 71773201 | adenine phosphoribosyltransferase isoform b [Homo sapiens] >gi|114664113|ref|XP_001137924.1| PREDICTED: adenine phosphoribosyltransferase isoform 1 [Pan troglodytes] >gi|119587165|gb|EAW66761.1| adenine phosphoribosyltransferase, isoform CRA_b [Homo sapiens] | 26.87 | 14.93% | 2 | 1 | 1 | 1 | 134 | 14.5 | 7.34 | 0.644 |
| 114062 | RecName: Full=Apolipoprotein(a); Short=Apo(a); Short=Lp(a); Flags: Precursor >gi|28620|emb|CAA29618.1| unnamed protein product [Homo sapiens] | 78.87 | 8.60% | 4 | 2 | 2 | 7 | 4548 | 501 | 5.88 | 0.645 |
| 119584204 | fibrinogen-like 1, isoform CRA_b [Homo sapiens] | 65.43 | 12.36% | 9 | 2 | 2 | 6 | 178 | 21 | 5.97 | 0.66 |
| 4758146 | neutrophil defensin 1 preproprotein [Homo sapiens] >gi|124248516|ref|NP_001035965.1| alpha-defensin 1 [Homo sapiens] >gi|30316322|sp|P59665.1|DEF1_HUMAN RecName: Full=Neutrophil defensin 1; AltName: Full=HNP-1; Short=HP-1; Short=HP1; AltName: Full=Defensin, alpha 1; Contains: RecName: Full=HP 1-56; Contains: RecName: Full=Neutrophil defensin 2; AltName: Full=HNP-2; Short=HP-2; Short=HP2; Flags: Precursor >gi|32402|emb|CAA36280.1| unnamed protein product [Homo sapiens] >gi|181527|gb|AAA52302.1| neutrophil peptide 1 precursor [Homo sapiens] >gi|181529|gb|AAA52303.1| defensin 1 [Homo sapiens] >gi|292363|gb|AAA36382.1| neutrophil peptide-1 [Homo sapiens] >gi|46854592|gb|AAH69423.1| Defensin, alpha 1 [Homo sapiens] >gi|50057839|gb|AAT68875.1| novel protein, similar to DEFA1 [Homo sapiens] >gi|50057842|gb|AAT68878.1| defensin, alpha 1, myeloid-related sequence [Homo sapiens] >gi|50057843|gb|AAT68879.1| novel protein, similar to DEFA1 [Homo sapiens] >gi|50057844|gb|AAT68880.1| novel protein, similar to DEFA1 [Homo sapiens] >gi|50057847|gb|AAT68883.1| defensin, alpha 1, myeloid-related sequence [Homo sapiens] >gi|50057848|gb|AAT68884.1| defensin, alpha 1, myeloid-related sequence [Homo sapiens] >gi|62739977|gb|AAH93791.1| Defensin, alpha 1 [Homo sapiens] >gi|85567619|gb|AAI12189.1| Defensin, alpha 1, preproprotein [Homo sapiens] >gi|446635|prf||1912193A defensin >gi|1098031|prf||2115200A neutrophil peptide | 43.25 | 32.98% | 2 | 1 | 1 | 2 | 94 | 10.2 | 6.99 | 0.662 |
| 119570830 | hCG1741471 [Homo sapiens] | 23 | 3.11% | 7 | 1 | 1 | 2 | 322 | 37.2 | 7.99 | 0.667 |
| 7012705 | immunoglobulin light chain variable region [Homo sapiens] | 212.91 | 68.22% | 425 | 1 | 4 | 27 | 107 | 11.5 | 7.96 | 0.668 |
| 70798883 | immunoglobulin kappa light chain variable region [Homo sapiens] | 93.38 | 25.24% | 97 | 1 | 2 | 2 | 103 | 11.1 | 8.88 | 0.67 |
| 189054579 | unnamed protein product [Homo sapiens] | 40.06 | 7.67% | 2 | 2 | 2 | 3 | 352 | 38.2 | 7.11 | 0.671 |
| 119625314 | fibrinogen gamma chain, isoform CRA_e [Homo sapiens] | 1193.9 | 62.53% | 5 | 6 | 27 | 1576 | 419 | 47.4 | 5.95 | 0.675 |
| 46254055 | immunoglobulin heavy chain [Homo sapiens] | 151.53 | 28.74% | 208 | 1 | 3 | 50 | 167 | 18 | 8.9 | 0.678 |
| 189067450 | unnamed protein product [Homo sapiens] | 26.83 | 2.55% | 1 | 1 | 1 | 4 | 275 | 32.3 | 8.37 | 0.683 |
| 114385630 | immunoglobulin light chain variable region [Homo sapiens] | 76.22 | 16.82% | 2 | 1 | 1 | 19 | 107 | 11.7 | 7.97 | 0.688 |
| 148733226 | serpin peptidase inhibitor, clade A (alpha-1 antiproteinase, antitrypsin), member 10 [Homo sapiens] | 73.38 | 8.11% | 5 | 3 | 3 | 7 | 444 | 50.7 | 8.28 | 0.69 |
| 194375299 | unnamed protein product [Homo sapiens] | 265.15 | 33.33% | 58 | 8 | 8 | 22 | 333 | 37.3 | 5.71 | 0.691 |
| 896272 | This CDS feature is included to show the translation of the corresponding V_region. Presently translation qualifiers on V_region features are illegal [Homo sapiens] | 285.25 | 64.57% | 2946 | 2 | 9 | 63 | 127 | 13.8 | 8.79 | 0.707 |
| 119625336 | fibrinogen beta chain, isoform CRA_b [Homo sapiens] | 570.94 | 53.04% | 3 | 4 | 14 | 328 | 247 | 27.7 | 7.99 | 0.708 |
| 194385488 | unnamed protein product [Homo sapiens] | 67.07 | 4.23% | 18 | 2 | 2 | 2 | 780 | 82.5 | 6.55 | 0.71 |
| 82734214 | beta-defensin 110 isoform a [Homo sapiens] >gi|84028871|sp|Q30KQ9.1|DB110_HUMAN RecName: Full=Beta-defensin 110; AltName: Full=Defensin, beta 110; AltName: Full=Beta-defensin 10; Short=DEFB-10; AltName: Full=Beta-defensin 111; AltName: Full=Defensin, beta 111; AltName: Full=Beta-defensin 11; Short=DEFB-11; Flags: Precursor >gi|66968904|gb|AAY59751.1| beta-defensin 111 [Homo sapiens] >gi|151555475|gb|AAI48542.1| Defensin, beta 111 [synthetic construct] >gi|162319076|gb|AAI56745.1| Defensin, beta 111 [synthetic construct] | 23.5 | 14.93% | 1 | 1 | 1 | 11 | 67 | 8 | 8.73 | 0.712 |
| 247424364 | immunoglobulin heavy chain variable region [Homo sapiens] | 165.61 | 50.81% | 1078 | 1 | 5 | 28 | 124 | 13.4 | 8.91 | 0.712 |
| 115298678 | complement C3 precursor [Homo sapiens] >gi|119370332|sp|P01024.2|CO3_HUMAN RecName: Full=Complement C3; AltName: Full=C3 and PZP-like alpha-2-macroglobulin domain-containing protein 1; Contains: RecName: Full=Complement C3 beta chain; Contains: RecName: Full=Complement C3 alpha chain; Contains: RecName: Full=C3a anaphylatoxin; Contains: RecName: Full=Complement C3b alpha' chain; Contains: RecName: Full=Complement C3c alpha' chain fragment 1; Contains: RecName: Full=Complement C3dg fragment; Contains: RecName: Full=Complement C3g fragment; Contains: RecName: Full=Complement C3d fragment; Contains: RecName: Full=Complement C3f fragment; Contains: RecName: Full=Complement C3c alpha' chain fragment 2; Flags: Precursor >gi|40786791|gb|AAR89906.1| complement component 3 [Homo sapiens] >gi|119589477|gb|EAW69071.1| complement component 3, isoform CRA_b [Homo sapiens] >gi|152012494|gb|AAI50201.1| Complement component 3 [Homo sapiens] >gi|152012784|gb|AAI50180.1| Complement component 3 [Homo sapiens] | 3018.01 | 58.15% | 7 | 76 | 76 | 444 | 1663 | 187 | 6.4 | 0.716 |
| 18025670 | immunoglobulin light chain variable region [Homo sapiens] | 66.85 | 25.23% | 17 | 1 | 2 | 4 | 107 | 11.8 | 9.01 | 0.716 |
| 49354849 | immunoglobulin E variable region [Homo sapiens] | 135.35 | 38.02% | 272 | 1 | 3 | 19 | 121 | 13.2 | 8.43 | 0.719 |
| 189066534 | unnamed protein product [Homo sapiens] | 76.02 | 20.00% | 4 | 2 | 2 | 3 | 175 | 20 | 9.01 | 0.719 |
| 58222839 | anti-tetanus toxoid immunoglobulin light chain variable region [Homo sapiens] | 60.6 | 30.84% | 125 | 1 | 2 | 9 | 107 | 11.7 | 6.54 | 0.721 |
| 119625338 | fibrinogen beta chain, isoform CRA_d [Homo sapiens] | 1669.35 | 67.61% | 7 | 24 | 34 | 2036 | 457 | 52.1 | 8.07 | 0.729 |
| 194383496 | unnamed protein product [Homo sapiens] | 925.68 | 45.45% | 12 | 1 | 21 | 152 | 605 | 68.3 | 5.82 | 0.742 |
| 3337390 | haptoglobin [Homo sapiens] | 399.49 | 39.42% | 15 | 5 | 12 | 56 | 345 | 38.2 | 6.6 | 0.745 |
| 178741 | apolipoprotein C-II [Homo sapiens] >gi|357629|prf||1303321A apolipoprotein CII | 353.8 | 65.93% | 6 | 6 | 6 | 187 | 91 | 10.2 | 4.56 | 0.747 |
| 15637419 | anti-pneumococcal capsular polysaccharide immunoglobulin heavy chain variable region [Homo sapiens] | 121.54 | 30.61% | 136 | 1 | 2 | 41 | 98 | 10.8 | 7.08 | 0.748 |
| 1655598 | lipopolysaccharide binding protein [Homo sapiens] >gi|4530277|gb|AAD21962.1| lipopolysaccharide-binding protein [Homo sapiens] | 464.59 | 23.70% | 7 | 10 | 10 | 75 | 481 | 53.3 | 6.7 | 0.752 |
| 27650476 | immunoglobulin heavy chain variable region [Homo sapiens] | 57.64 | 46.48% | 1 | 1 | 2 | 2 | 71 | 8 | 8.72 | 0.753 |
| 262231791 | complement factor H-related protein 3 isoform 2 precursor [Homo sapiens] >gi|194391108|dbj|BAG60672.1| unnamed protein product [Homo sapiens] | 53.08 | 7.43% | 12 | 2 | 2 | 7 | 269 | 30.7 | 7.78 | 0.755 |
| 184086 | histone H2B.1 [Homo sapiens] | 30.74 | 8.91% | 34 | 1 | 1 | 6 | 101 | 11.3 | 10.14 | 0.758 |
| 11761629 | fibrinogen alpha chain isoform alpha preproprotein [Homo sapiens] >gi|182426|gb|AAA52427.1| A-alpha fibrinogen [Homo sapiens] >gi|458554|gb|AAA17055.1| common fibrinogen alpha chain [Homo sapiens] >gi|4033511|gb|AAC97143.1| fibrinogen alpha subunit [Homo sapiens] >gi|67514250|gb|AAH98280.1| Fibrinogen alpha chain [Homo sapiens] >gi|71043421|gb|AAH99720.1| Fibrinogen alpha chain [Homo sapiens] >gi|71043479|gb|AAH99706.1| Fibrinogen, alpha chain, isoform alpha preproprotein [Homo sapiens] >gi|74355612|gb|AAI01936.1| Fibrinogen alpha chain [Homo sapiens] >gi|119625332|gb|EAX04927.1| fibrinogen alpha chain, isoform CRA_c [Homo sapiens] >gi|119625333|gb|EAX04928.1| fibrinogen alpha chain, isoform CRA_c [Homo sapiens] | 2376.76 | 58.07% | 12 | 45 | 45 | 2395 | 644 | 69.7 | 8.06 | 0.761 |
| 5360675 | anti-Entamoeba histolytica immunoglobulin kappa light chain [Homo sapiens] | 534.71 | 48.37% | 58 | 1 | 8 | 578 | 215 | 23.4 | 6.54 | 0.766 |
| 12054080 | immunoglobulin heavy chain constant region mu [Homo sapiens] | 1191.77 | 62.03% | 8 | 1 | 22 | 1031 | 453 | 49.4 | 6.83 | 0.772 |
| 13540563 | complement factor H-related protein 5 precursor [Homo sapiens] >gi|23396597|sp|Q9BXR6.1|FHR5_HUMAN RecName: Full=Complement factor H-related protein 5; Short=FHR-5; Flags: Precursor >gi|13195239|gb|AAK15619.1|AF295327_1 complement factor H-related protein 5 [Homo sapiens] >gi|84627551|gb|AAI11774.1| Complement factor H-related 5 [Homo sapiens] >gi|119611674|gb|EAW91268.1| complement factor H-related 5 [Homo sapiens] | 305.8 | 19.86% | 4 | 9 | 10 | 60 | 569 | 64.4 | 7.06 | 0.775 |
| 34530477 | unnamed protein product [Homo sapiens] | 82.31 | 15.31% | 6 | 2 | 2 | 2 | 196 | 20.8 | 8.4 | 0.775 |
| 119608546 | ficolin (collagen/fibrinogen domain containing) 1, isoform CRA_d [Homo sapiens] | 57.63 | 5.10% | 7 | 1 | 2 | 2 | 314 | 33.8 | 6.64 | 0.775 |
| 47124510 | APCS protein [Homo sapiens] | 30.38 | 23.91% | 3 | 1 | 1 | 2 | 46 | 5.3 | 4.46 | 0.779 |
| 224979536 | anti-IL-15 immunoglobulin heavy chain variable region 1 [Homo sapiens] | 21.9 | 16.10% | 1 | 1 | 1 | 2 | 118 | 12.7 | 7.12 | 0.781 |
| 1769552 | von Willebrand factor [Homo sapiens] | 27.48 | 3.73% | 10 | 1 | 1 | 2 | 241 | 27.1 | 6.93 | 0.785 |
| 553426 | immunoglobulin heavy chain VDJC region [Homo sapiens] | 103.19 | 14.58% | 39 | 1 | 3 | 36 | 144 | 15.6 | 9.13 | 0.786 |
| 16878304 | ABCB9 protein [Homo sapiens] >gi|119618763|gb|EAW98357.1| ATP-binding cassette, sub-family B (MDR/TAP), member 9, isoform CRA_e [Homo sapiens] | 39.56 | 1.17% | 11 | 1 | 1 | 8 | 596 | 66.2 | 8.5 | 0.789 |
| 160858157 | collagen type VI alpha 5 [Homo sapiens] | 23.55 | 1.31% | 4 | 1 | 1 | 1 | 609 | 68.9 | 6.77 | 0.795 |
| 30425438 | out at first protein homolog precursor [Homo sapiens] >gi|74727479|sp|Q86UD1.1|OAF_HUMAN RecName: Full=Out at first protein homolog; AltName: Full=HCV NS5A-transactivated protein 13 target protein 2; Flags: Precursor >gi|28838376|gb|AAH47726.1| OAF homolog (Drosophila) [Homo sapiens] >gi|38505473|gb|AAR23238.1| NS5ATP13TP2 [Homo sapiens] | 110.71 | 18.68% | 2 | 3 | 3 | 9 | 273 | 30.7 | 6.84 | 0.797 |
| 4505735 | platelet factor 4 variant [Homo sapiens] >gi|130306|sp|P10720.1|PF4V_HUMAN RecName: Full=Platelet factor 4 variant; AltName: Full=PF4var1; AltName: Full=PF4alt; AltName: Full=C-X-C motif chemokine 4 variant; AltName: Full=CXCL4L1; Contains: RecName: Full=Platelet factor 4 variant(4-74); Contains: RecName: Full=Platelet factor 4 variant(5-74); Contains: RecName: Full=Platelet factor 4 variant(6-74); Flags: Precursor >gi|292390|gb|AAA60067.1| platelet factor 4 [Homo sapiens] >gi|119626097|gb|EAX05692.1| platelet factor 4 variant 1 [Homo sapiens] >gi|120659980|gb|AAI30654.1| Platelet factor 4 variant 1 [Homo sapiens] >gi|120660120|gb|AAI30658.1| Platelet factor 4 variant 1 [Homo sapiens] | 168.33 | 48.08% | 1 | 2 | 4 | 9 | 104 | 11.5 | 9.1 | 0.798 |
| 14030460 | mannan-binding lectin MBL precursor [Homo sapiens] | 176.08 | 21.37% | 5 | 6 | 6 | 13 | 248 | 26.1 | 5.49 | 0.799 |
| 77379566 | immunoglobulin kappa chain variable region [Homo sapiens] | 76.5 | 22.22% | 418 | 1 | 2 | 25 | 108 | 12 | 8.81 | 0.8 |
| 87299000 | immunoglobulin light chain variable region [Homo sapiens] | 120.82 | 37.19% | 40 | 1 | 3 | 9 | 121 | 13.2 | 8.91 | 0.804 |
| 4323960 | immunoglobulin kappa light chain variable region [Homo sapiens] | 94.68 | 26.17% | 419 | 1 | 2 | 26 | 107 | 11.7 | 7.97 | 0.806 |
| 194388758 | unnamed protein product [Homo sapiens] | 70.32 | 11.19% | 18 | 3 | 3 | 5 | 277 | 30.8 | 8.66 | 0.807 |
| 181482 | serum vitamin D-binding protein precursor [Homo sapiens] | 825.06 | 59.28% | 2 | 2 | 23 | 76 | 474 | 53 | 5.54 | 0.808 |
| 178834 | apolipoprotein CI [Homo sapiens] | 57.49 | 26.09% | 2 | 2 | 2 | 16 | 69 | 7.7 | 6.73 | 0.812 |
| 74355107 | BRF1 protein [Homo sapiens] | 30.09 | 4.97% | 11 | 1 | 1 | 11 | 161 | 18.3 | 8.62 | 0.815 |
| 37789448 | immunoglobulin lambda light chain variable region [Homo sapiens] | 21.04 | 9.64% | 4 | 1 | 1 | 1 | 83 | 8.9 | 7.12 | 0.815 |
| 221039462 | unnamed protein product [Homo sapiens] | 835.73 | 59.63% | 2 | 2 | 17 | 198 | 270 | 30.4 | 7.99 | 0.819 |
| 158256710 | unnamed protein product [Homo sapiens] | 235.85 | 9.06% | 11 | 8 | 8 | 18 | 1170 | 129.3 | 4.94 | 0.821 |
| 189066632 | unnamed protein product [Homo sapiens] | 253.44 | 16.00% | 4 | 8 | 8 | 42 | 525 | 59.5 | 7.44 | 0.824 |
| 31337558 | immunoglobulin gamma heavy chain variable region [Homo sapiens] | 125.51 | 29.37% | 842 | 1 | 3 | 26 | 126 | 14.2 | 6.62 | 0.825 |
| 119579722 | complement factor properdin, isoform CRA_b [Homo sapiens] | 25.72 | 3.82% | 6 | 1 | 1 | 2 | 340 | 37.3 | 8.02 | 0.826 |
| 2258128 | complement 9 [Homo sapiens] | 51.17 | 4.50% | 5 | 2 | 2 | 3 | 533 | 60.4 | 5.59 | 0.827 |
| 119598593 | alpha-2-HS-glycoprotein, isoform CRA_a [Homo sapiens] | 532.05 | 46.99% | 2 | 1 | 11 | 318 | 366 | 39.2 | 5.72 | 0.827 |
| 119590943 | fibronectin 1, isoform CRA_h [Homo sapiens] | 2674.5 | 37.42% | 82 | 36 | 60 | 502 | 2330 | 256.3 | 5.8 | 0.828 |
| 21669449 | immunoglobulin kappa light chain VLJ region [Homo sapiens] | 511.38 | 43.77% | 309 | 1 | 9 | 529 | 265 | 28.5 | 7.97 | 0.828 |
| 28931 | beta-subunit (AA 1-312) [Homo sapiens] | 29.34 | 7.69% | 4 | 1 | 1 | 3 | 312 | 34 | 5.03 | 0.831 |
| 158255874 | unnamed protein product [Homo sapiens] | 487.27 | 17.28% | 9 | 12 | 12 | 53 | 1065 | 122.1 | 5.74 | 0.831 |
| 119625310 | fibrinogen gamma chain, isoform CRA_a [Homo sapiens] >gi|119625317|gb|EAX04912.1| fibrinogen gamma chain, isoform CRA_a [Homo sapiens] >gi|119625323|gb|EAX04918.1| fibrinogen gamma chain, isoform CRA_a [Homo sapiens] >gi|119625328|gb|EAX04923.1| fibrinogen gamma chain, isoform CRA_a [Homo sapiens] | 1199.64 | 68.56% | 25 | 2 | 28 | 1123 | 334 | 37.7 | 6.29 | 0.832 |
| 542891 | Ig kappa chain V-J region - human >gi|441433|emb|CAA51150.1| Ig kappa light chain (VJ) [Homo sapiens] | 84.79 | 29.77% | 418 | 1 | 2 | 25 | 131 | 14.2 | 6.51 | 0.833 |
| 1335098 | unnamed protein product [Homo sapiens] | 320.81 | 23.92% | 9 | 8 | 8 | 50 | 439 | 49.3 | 6.92 | 1.202 |
| 11558186 | immunoglobulin light chain variable region [Homo sapiens] | 33.94 | 6.06% | 1 | 1 | 1 | 2 | 132 | 13.7 | 8.94 | 1.203 |
| 1082521 | Ig kappa chain V region - human (fragment) >gi|809555|emb|CAA58108.1| immunoglobulin kappa light chain [Homo sapiens] | 87.06 | 36.84% | 16 | 1 | 3 | 3 | 114 | 12.3 | 8.94 | 1.204 |
| 13661193 | fibulin-1 isoform D precursor [Homo sapiens] >gi|1621019|gb|AAB17099.1| fibulin-1D [Homo sapiens] | 158.68 | 9.25% | 23 | 5 | 5 | 24 | 703 | 77.2 | 5.26 | 1.205 |
| 755742 | unnamed protein product [Homo sapiens] | 103.66 | 51.02% | 10 | 2 | 2 | 11 | 49 | 5.4 | 8.21 | 1.21 |
| 57162363 | KIAA0515 [Homo sapiens] | 20.36 | 1.53% | 8 | 1 | 1 | 1 | 587 | 63.2 | 6.73 | 1.216 |
| 27369048 | immunoglobulin lambda light chain variable region [Homo sapiens] | 10.42 | 9.29% | 1 | 1 | 1 | 1 | 140 | 14.5 | 7.06 | 1.222 |
| 158256226 | unnamed protein product [Homo sapiens] | 1927.17 | 45.90% | 5 | 1 | 46 | 523 | 1231 | 138.9 | 6.71 | 1.223 |
| 219566389 | immunoglobulin kappa light chain [Homo sapiens] | 132.14 | 39.29% | 540 | 1 | 3 | 31 | 112 | 12.4 | 8.94 | 1.224 |
| 119609947 | TIMP metallopeptidase inhibitor 2, isoform CRA_c [Homo sapiens] >gi|194376854|dbj|BAG57573.1| unnamed protein product [Homo sapiens] | 78.1 | 30.07% | 5 | 3 | 3 | 7 | 143 | 16.1 | 6.3 | 1.224 |
| 247424903 | immunoglobulin heavy chain variable region [Homo sapiens] | 286.5 | 51.22% | 1252 | 1 | 5 | 69 | 123 | 13.4 | 7.96 | 1.231 |
| 70888104 | immunoglobulin lambda light chain variable region [Homo sapiens] | 183.7 | 55.56% | 47 | 1 | 4 | 39 | 108 | 11.6 | 4.65 | 1.231 |
| 183851 | G-gamma-hemoglobin [Homo sapiens] | 64.89 | 22.77% | 15 | 1 | 2 | 21 | 101 | 11 | 6.68 | 1.232 |
| 15080499 | Serpin peptidase inhibitor, clade A (alpha-1 antiproteinase, antitrypsin), member 1 [Homo sapiens] | 343.07 | 34.93% | 29 | 11 | 11 | 63 | 418 | 46.7 | 5.59 | 1.244 |
| 4505047 | lumican precursor [Homo sapiens] >gi|20141464|sp|P51884.2|LUM_HUMAN RecName: Full=Lumican; AltName: Full=Keratan sulfate proteoglycan lumican; Short=KSPG lumican; Flags: Precursor >gi|699577|gb|AAA91639.1| lumican [Homo sapiens] >gi|13937865|gb|AAH07038.1| Lumican [Homo sapiens] >gi|23271183|gb|AAH35997.1| Lumican [Homo sapiens] >gi|30582253|gb|AAP35353.1| lumican [Homo sapiens] >gi|60656531|gb|AAX32829.1| lumican [synthetic construct] >gi|60656533|gb|AAX32830.1| lumican [synthetic construct] >gi|119617855|gb|EAW97449.1| lumican [Homo sapiens] >gi|123983755|gb|ABM83480.1| lumican [synthetic construct] >gi|123998179|gb|ABM86691.1| lumican [synthetic construct] >gi|189053396|dbj|BAG35562.1| unnamed protein product [Homo sapiens] | 271.3 | 31.36% | 3 | 10 | 10 | 21 | 338 | 38.4 | 6.61 | 1.245 |
| 51103589 | immunoglobulin variable region VL lambda domain [Homo sapiens] | 92.76 | 25.00% | 17 | 1 | 2 | 6 | 108 | 11.4 | 4.92 | 1.247 |
| 21669509 | immunoglobulin lambda light chain VLJ region [Homo sapiens] | 302.26 | 34.43% | 250 | 1 | 7 | 501 | 273 | 28.2 | 7.14 | 1.252 |
| 194385604 | unnamed protein product [Homo sapiens] | 424.74 | 28.78% | 11 | 9 | 13 | 35 | 615 | 68.9 | 5.34 | 1.259 |
| 77157824 | immunoglobulin lambda light chain variable region [Homo sapiens] >gi|77157826|gb|ABA61886.1| immunoglobulin lambda light chain variable region [Homo sapiens] | 99.36 | 43.52% | 461 | 1 | 3 | 17 | 108 | 11.3 | 7.96 | 1.271 |
| 47132620 | keratin, type II cytoskeletal 2 epidermal [Homo sapiens] >gi|239938650|sp|P35908.2|K22E_HUMAN RecName: Full=Keratin, type II cytoskeletal 2 epidermal; AltName: Full=Cytokeratin-2e; Short=CK-2e; AltName: Full=Keratin-2e; Short=K2e; AltName: Full=Type-II keratin Kb2; AltName: Full=Keratin-2 epidermis; AltName: Full=Epithelial keratin-2e >gi|64654451|gb|AAH96294.1| Keratin 2 [Homo sapiens] >gi|68563334|gb|AAH99644.1| Keratin 2 [Homo sapiens] >gi|68563400|gb|AAH99643.1| Keratin 2 [Homo sapiens] >gi|119617047|gb|EAW96641.1| keratin 2A (epidermal ichthyosis bullosa of Siemens) [Homo sapiens] | 343.89 | 23.00% | 45 | 8 | 10 | 19 | 639 | 65.4 | 8 | 1.272 |
| 28559048 | Ig rearranged L-chain V-region [Homo sapiens] | 51.37 | 14.37% | 243 | 1 | 2 | 14 | 167 | 17.3 | 5.35 | 1.272 |
| 153266841 | beta-2-glycoprotein 1 precursor [Homo sapiens] >gi|543826|sp|P02749.3|APOH_HUMAN RecName: Full=Beta-2-glycoprotein 1; AltName: Full=Beta-2-glycoprotein I; Short=Beta(2)GPI; Short=B2GPI; AltName: Full=Apolipoprotein H; Short=Apo-H; AltName: Full=Activated protein C-binding protein; AltName: Full=APC inhibitor; AltName: Full=Anticardiolipin cofactor; Flags: Precursor >gi|28814|emb|CAA40977.1| Apolipoprotein H (beta2-glycoprotein I) [Homo sapiens] >gi|178857|gb|AAA51766.1| apolipoprotein H [Homo sapiens] >gi|244678|gb|AAB21330.1| beta 2-glycoprotein I [Homo sapiens] >gi|2765106|emb|CAA72279.1| beta-2-glycoprotein I [Homo sapiens] >gi|18089104|gb|AAH20703.1| Apolipoprotein H (beta-2-glycoprotein I) [Homo sapiens] >gi|20072226|gb|AAH26283.1| Apolipoprotein H (beta-2-glycoprotein I) [Homo sapiens] >gi|32165624|gb|AAP72014.1| apolipoprotein H (beta-2-glycoprotein I) [Homo sapiens] >gi|119609418|gb|EAW89012.1| apolipoprotein H (beta-2-glycoprotein I), isoform CRA_a [Homo sapiens] >gi|123980918|gb|ABM82288.1| apolipoprotein H (beta-2-glycoprotein I) [synthetic construct] >gi|123995733|gb|ABM85468.1| apolipoprotein H (beta-2-glycoprotein I) [synthetic construct] | 228.1 | 33.91% | 5 | 8 | 8 | 21 | 345 | 38.3 | 7.97 | 1.273 |
| 86438992 | immunoglobulin heavy chain [Homo sapiens] | 128.3 | 28.45% | 838 | 1 | 3 | 26 | 116 | 12.8 | 7.97 | 1.273 |
| 5419725 | immunoglobulin light chain variable region [Homo sapiens] | 150.43 | 31.48% | 258 | 1 | 3 | 10 | 108 | 11.5 | 7.12 | 1.274 |
| 58223278 | anti-tetanus toxoid immunoglobulin light chain variable region [Homo sapiens] | 269.33 | 82.41% | 704 | 1 | 5 | 33 | 108 | 11.5 | 7.96 | 1.277 |
| 247425451 | immunoglobulin heavy chain variable region [Homo sapiens] | 125.32 | 24.59% | 743 | 1 | 2 | 24 | 122 | 13.1 | 6.57 | 1.279 |
| 13278732 | Complement factor B [Homo sapiens] >gi|14124934|gb|AAH07990.1| Complement factor B [Homo sapiens] >gi|62898361|dbj|BAD97120.1| complement factor B preproprotein variant [Homo sapiens] >gi|119623955|gb|EAX03550.1| complement factor B [Homo sapiens] >gi|123982996|gb|ABM83239.1| complement factor B [synthetic construct] >gi|123997681|gb|ABM86442.1| complement factor B [synthetic construct] | 47.16 | 3.14% | 5 | 2 | 2 | 5 | 764 | 85.5 | 6.96 | 1.287 |
| 567112 | OMM protein (Ig gamma3) heavy chain [Homo sapiens] | 307.84 | 28.75% | 3 | 1 | 7 | 91 | 313 | 34.9 | 7.49 | 1.289 |
| 119608489 | ADAM metallopeptidase with thrombospondin type 1 motif, 13, isoform CRA_a [Homo sapiens] >gi|119608490|gb|EAW88084.1| ADAM metallopeptidase with thrombospondin type 1 motif, 13, isoform CRA_a [Homo sapiens] >gi|119608491|gb|EAW88085.1| ADAM metallopeptidase with thrombospondin type 1 motif, 13, isoform CRA_a [Homo sapiens] | 106.05 | 4.80% | 8 | 3 | 3 | 4 | 1041 | 112.4 | 7.31 | 1.291 |
| 272982598 | anti-HIV-1 gp120 immunoglobulin heavy chain [Homo sapiens] | 550.51 | 27.22% | 208 | 1 | 10 | 252 | 474 | 51.8 | 7.91 | 1.295 |
| 156765940 | collectin kidney K1 [Homo sapiens] | 260.61 | 27.41% | 26 | 7 | 7 | 33 | 197 | 21.6 | 4.75 | 1.309 |
| 112700542 | immunoglobulin heavy chain variable region [Homo sapiens] | 154.72 | 43.56% | 1441 | 1 | 4 | 24 | 101 | 10.9 | 8.29 | 1.309 |
| 111917949 | immunoglobulin heavy chain variable region [Homo sapiens] | 76.7 | 18.18% | 77 | 1 | 2 | 8 | 154 | 16.7 | 7.84 | 1.331 |
| 21669523 | immunoglobulin lambda light chain VLJ region [Homo sapiens] | 427.3 | 39.63% | 106 | 1 | 10 | 568 | 270 | 28.1 | 7.14 | 1.344 |
| 119573006 | apolipoprotein A-II, isoform CRA_c [Homo sapiens] | 399.98 | 67.50% | 1 | 1 | 8 | 484 | 80 | 9 | 6.57 | 1.349 |
| 55957679 | cartilage acidic protein 1 [Homo sapiens] >gi|55958790|emb|CAI14275.1| cartilage acidic protein 1 [Homo sapiens] | 68.51 | 5.34% | 10 | 3 | 3 | 5 | 524 | 56.5 | 5.55 | 1.35 |
| 553490 | immunoglobulin lambda-chain [Homo sapiens] | 64.73 | 12.50% | 89 | 1 | 2 | 7 | 128 | 13.4 | 7.81 | 1.351 |
| 194383694 | unnamed protein product [Homo sapiens] | 112.48 | 6.53% | 10 | 3 | 3 | 10 | 704 | 77.2 | 4.53 | 1.365 |
| 189217853 | 72 kDa type IV collagenase isoform b [Homo sapiens] | 158.27 | 16.56% | 11 | 6 | 6 | 9 | 610 | 68.8 | 5.34 | 1.368 |
| 190609605 | immunoglobulin kappa light chain virable region [Homo sapiens] | 48.36 | 18.75% | 2 | 1 | 1 | 4 | 96 | 10.4 | 8.5 | 1.399 |
| 194390508 | unnamed protein product [Homo sapiens] | 1308.75 | 40.93% | 24 | 1 | 25 | 226 | 1014 | 111.2 | 6.21 | 1.411 |
| 119600101 | phosphatidylinositol-specific phospholipase C, X domain containing 2 [Homo sapiens] | 34.44 | 2.42% | 3 | 1 | 1 | 1 | 289 | 33 | 9.44 | 1.414 |
| 18307322 | immunoglobulin light chain lambda variable region [Homo sapiens] | 27.87 | 12.62% | 1 | 1 | 1 | 1 | 103 | 10.8 | 5.25 | 1.419 |
| 193784716 | unnamed protein product [Homo sapiens] | 58.37 | 6.86% | 1 | 3 | 3 | 3 | 423 | 47.7 | 5.52 | 1.42 |
| 177870 | alpha-2-macroglobulin precursor [Homo sapiens] >gi|25303946|gb|AAH40071.1| Alpha-2-macroglobulin [Homo sapiens] | 1686.87 | 42.61% | 11 | 38 | 44 | 243 | 1474 | 163.2 | 6.42 | 1.429 |
| 71773110 | apolipoprotein A-IV precursor [Homo sapiens] | 1731.94 | 81.31% | 2 | 4 | 39 | 786 | 396 | 45.3 | 5.38 | 1.435 |
| 6643569 | immunoglobulin lambda light chain variable region [Homo sapiens] >gi|6643571|gb|AAF20644.1| immunoglobulin lambda light chain variable region [Homo sapiens] | 46.99 | 25.93% | 193 | 1 | 2 | 2 | 108 | 11.5 | 5.96 | 1.446 |
| 587350 | immunoglobulin kappa light chain variable region [Homo sapiens] | 67.14 | 29.91% | 10 | 1 | 3 | 5 | 117 | 12.6 | 7.12 | 1.448 |
| 21669285 | immunoglobulin lambda light chain VLJ region [Homo sapiens] | 43.55 | 32.43% | 4 | 1 | 2 | 3 | 111 | 11.7 | 8.91 | 1.456 |
| 194273292 | immunoglobulin heavy chain variable region [Homo sapiens] | 227.62 | 70.30% | 896 | 1 | 6 | 13 | 101 | 11 | 9.72 | 1.484 |
| 3970966 | spa-1-like; similar to AF026504 (PID:g2555183) [Homo sapiens] | 23.31 | 3.00% | 6 | 1 | 1 | 2 | 699 | 77.9 | 8.02 | 1.486 |
| 77379428 | immunoglobulin kappa chain variable region [Homo sapiens] | 38.02 | 21.49% | 1 | 1 | 1 | 2 | 121 | 13.1 | 6 | 1.489 |
| 20377087 | intestinal lactoferrin receptor [Homo sapiens] >gi|7019846|dbj|BAA90893.1| unnamed protein product [Homo sapiens] >gi|8096221|dbj|BAA96094.1| intelectin [Homo sapiens] >gi|18091783|gb|AAL58073.1| endothelial lectin HL-1 [Homo sapiens] >gi|37181843|gb|AAQ88725.1| ITLN [Homo sapiens] >gi|37724012|gb|AAO17801.1| intelectin [Homo sapiens] >gi|44976129|gb|AAS49907.1| omentin [Homo sapiens] >gi|48146565|emb|CAG33505.1| ITLN1 [Homo sapiens] >gi|52843233|gb|AAU88047.1| intelectin 1 [Homo sapiens] >gi|62896625|dbj|BAD96253.1| intelectin variant [Homo sapiens] | 171.75 | 29.07% | 7 | 7 | 7 | 13 | 313 | 35 | 5.82 | 1.508 |
| 77378386 | immunoglobulin lambda light chain variable region [Homo sapiens] | 57.61 | 36.89% | 113 | 1 | 2 | 7 | 122 | 12.6 | 8.5 | 1.509 |
| 21518719 | hyaluronidase 1 variant 5 [Homo sapiens] >gi|119585494|gb|EAW65090.1| hyaluronoglucosaminidase 1, isoform CRA_f [Homo sapiens] | 46.2 | 25.00% | 12 | 1 | 1 | 2 | 96 | 10.4 | 8.53 | 1.524 |
| 87299014 | immunoglobulin light chain variable region [Homo sapiens] | 454.36 | 71.05% | 348 | 1 | 9 | 514 | 152 | 16.1 | 4.93 | 1.531 |
| 21669521 | immunoglobulin lambda light chain VLJ region [Homo sapiens] | 493.89 | 40.52% | 420 | 1 | 11 | 747 | 269 | 27.9 | 7.91 | 1.536 |
| 3004726 | IgM heavy chain variable region [Homo sapiens] | 146.27 | 43.65% | 494 | 1 | 4 | 12 | 126 | 14 | 7.91 | 1.547 |
| 3153306 | immunoglobulin light chain variable region [Homo sapiens] | 130.73 | 33.87% | 80 | 2 | 5 | 14 | 124 | 12.9 | 8.5 | 1.562 |
| 21628665 | immunoglobulin light chain constant region [Homo sapiens] | 116.72 | 91.43% | 1 | 1 | 3 | 82 | 35 | 3.9 | 6.48 | 1.567 |
| 34534492 | unnamed protein product [Homo sapiens] | 13.39 | 8.66% | 1 | 1 | 1 | 1 | 277 | 30.5 | 8.56 | 1.577 |
| 37694587 | immunoglobulin heavy chain variable region [Homo sapiens] | 81.8 | 25.64% | 12 | 1 | 2 | 5 | 117 | 12.9 | 7.39 | 1.581 |
| 195452250 | immunoglobulin lambda light chain variable region [Homo sapiens] | 32.98 | 19.75% | 4 | 1 | 1 | 10 | 81 | 8.4 | 4.44 | 1.587 |
| 47271320 | immunoglobulin lambda light chain variable region [Homo sapiens] | 59.98 | 21.05% | 104 | 1 | 2 | 5 | 114 | 12.1 | 5.01 | 1.599 |
| 109240688 | immunoglobulin kappa chain variable region [Homo sapiens] | 41.25 | 15.09% | 2 | 1 | 1 | 3 | 106 | 11.7 | 7.99 | 1.599 |
| 4321593 | immunoglobulin G kappa chain [Homo sapiens] | 361.42 | 38.25% | 71 | 1 | 5 | 126 | 217 | 24 | 6.55 | 1.607 |
| 1620396 | haptoglobin [Homo sapiens] | 370.62 | 34.77% | 7 | 4 | 11 | 42 | 348 | 39 | 6.89 | 1.617 |
| 158259857 | unnamed protein product [Homo sapiens] | 262.47 | 21.62% | 8 | 8 | 8 | 22 | 495 | 54.2 | 5.97 | 1.65 |
| 15886930 | immunoglobulin heavy chain variable region [Homo sapiens] | 122.46 | 46.61% | 493 | 1 | 4 | 11 | 118 | 12.9 | 8.41 | 1.65 |
| 11122875 | glycosylphosphatidylinositol phospholipase D [Homo sapiens] | 137.2 | 8.32% | 8 | 3 | 3 | 4 | 457 | 48.5 | 6.2 | 1.674 |
| 7770217 | PRO2675 [Homo sapiens] >gi|119626082|gb|EAX05677.1| albumin, isoform CRA_s [Homo sapiens] | 939.35 | 78.47% | 5 | 1 | 22 | 318 | 288 | 32.6 | 6.54 | 1.942 |
| 21668722 | immunoglobulin heavy chain VHDJ region [Homo sapiens] | 99.89 | 18.11% | 424 | 1 | 2 | 8 | 127 | 13.7 | 8.85 | 1.954 |
| 221044282 | unnamed protein product [Homo sapiens] | 229.04 | 9.77% | 4 | 5 | 5 | 15 | 870 | 95.9 | 4.64 | 2.015 |
| 170684488 | immunoglobulin lambda 1 light chain [Homo sapiens] | 195.72 | 32.87% | 43 | 1 | 4 | 366 | 216 | 22.6 | 7.33 | 2.027 |
| 58222449 | anti-tetanus toxoid immunoglobulin heavy chain variable region [Homo sapiens] | 102.65 | 21.49% | 133 | 1 | 3 | 20 | 121 | 13.2 | 8.46 | 2.081 |
| 8777875 | immunoglobulin light chain variable region [Homo sapiens] | 174.72 | 37.61% | 423 | 1 | 3 | 26 | 109 | 12 | 8.48 | 2.191 |
| 221042228 | unnamed protein product [Homo sapiens] | 15.94 | 2.78% | 1 | 1 | 1 | 1 | 539 | 61.3 | 7.74 | 2.315 |
| 33235626 | immunoglobulin kappa light chain [Homo sapiens] | 146.86 | 31.48% | 467 | 1 | 2 | 22 | 108 | 11.7 | 9.17 | 2.552 |
| 4502027 | serum albumin preproprotein [Homo sapiens] >gi|197098046|ref|NP_001127106.1| serum albumin precursor [Pongo abelii] >gi|113576|sp|P02768.2|ALBU_HUMAN RecName: Full=Serum albumin; Flags: Precursor >gi|75054626|sp|Q5NVH5.1|ALBU_PONAB RecName: Full=Serum albumin; Flags: Precursor >gi|7770117|gb|AAF69594.1|AF119917_2 PRO0903 [Homo sapiens] >gi|178344|gb|AAA98797.1| albumin [Homo sapiens] >gi|21706456|gb|AAH34023.1| Albumin [Homo sapiens] >gi|23243418|gb|AAH36003.1| Albumin [Homo sapiens] >gi|49176517|gb|AAT52213.1| cell growth inhibiting protein 42 [Homo sapiens] >gi|52001697|gb|AAU21642.1| serum albumin precursor [Homo sapiens] >gi|56403804|emb|CAI29688.1| hypothetical protein [Pongo abelii] >gi|115607207|gb|ABJ16448.1| serum albumin [Homo sapiens] >gi|119626081|gb|EAX05676.1| albumin, isoform CRA_r [Homo sapiens] >gi|123981022|gb|ABM82340.1| albumin [synthetic construct] >gi|123995825|gb|ABM85514.1| albumin [synthetic construct] >gi|152112964|gb|ABS29264.1| albumin [Homo sapiens] | 1969.11 | 76.35% | 36 | 24 | 45 | 799 | 609 | 69.3 | 6.28 | 2.635 |
| 4557871 | serotransferrin precursor [Homo sapiens] >gi|136191|sp|P02787.2|TRFE_HUMAN RecName: Full=Serotransferrin; Short=Transferrin; AltName: Full=Siderophilin; AltName: Full=Beta-1 metal-binding globulin; Flags: Precursor >gi|248648|gb|AAB22049.1| transferrin [Homo sapiens] >gi|339453|gb|AAA61140.1| transferrin precursor [Homo sapiens] >gi|15021381|gb|AAK77664.1| transferin [Homo sapiens] >gi|31415705|gb|AAP45055.1| transferrin [Homo sapiens] >gi|94717618|gb|ABF47110.1| transferrin [Homo sapiens] >gi|119599573|gb|EAW79167.1| transferrin, isoform CRA_d [Homo sapiens] | 635.11 | 29.94% | 18 | 15 | 15 | 60 | 698 | 77 | 7.12 | 3.262 |
| 346196 | Ig lambda chain V region - human >gi|452933|gb|AAB28790.1| anti-cardiolipin/beta 2 glycoprotein I immunoglobulin light chain variable region [Homo sapiens] | 102.42 | 21.67% | 284 | 1 | 3 | 22 | 120 | 12.4 | 6.48 | 3.578 |
| 33319586 | Ig heavy chain variable region, VH3 family [Homo sapiens] | 125.55 | 25.00% | 742 | 1 | 2 | 23 | 120 | 13 | 4.77 | 5.884 |
| 94469923 | anti-West Nile virus immunoglobulin light chain variable region [Homo sapiens] | 38.87 | 21.82% | 1 | 1 | 1 | 1 | 110 | 12 | 7.96 | 5.898 |
| 2253348 | immunoglobulin heavy chain variable region [Homo sapiens] | 69.15 | 22.13% | 31 | 1 | 2 | 3 | 122 | 13.4 | 9.07 | 9.322 |

**Mix vs. control**

| Accession | Description | Score | Coverage | # Proteins | # Unique Peptides | # Peptides | # PSMs | # AAs | MW [kDa] | calc. pI | Mix/Control |
| --- | --- | --- | --- | --- | --- | --- | --- | --- | --- | --- | --- |
| 13195586 | hemoglobin alpha 1 globin chain [Homo sapiens] | 103.91 | 24.00% | 13 | 2 | 2 | 25 | 100 | 10.7 | 7.72 | 0.167 |
| 36321 | SAA precursor polypeptide (119 AA) [Homo sapiens] | 245.07 | 52.10% | 10 | 2 | 5 | 45 | 119 | 13.1 | 8.18 | 0.197 |
| 18418633 | mutant beta-globin [Homo sapiens] | 289.03 | 71.43% | 76 | 7 | 8 | 59 | 147 | 16 | 7.69 | 0.243 |
| 194375974 | unnamed protein product [Homo sapiens] | 60.43 | 9.84% | 5 | 2 | 2 | 3 | 183 | 20.1 | 8.78 | 0.25 |
| 124504316 | HIST2H4B protein [Homo sapiens] | 46.54 | 9.80% | 6 | 1 | 1 | 1 | 102 | 11.4 | 11.36 | 0.261 |
| 13937839 | SAA1 protein [Homo sapiens] >gi|123983058|gb|ABM83270.1| serum amyloid A1 [synthetic construct] >gi|123983248|gb|ABM83365.1| serum amyloid A1 [synthetic construct] >gi|123997747|gb|ABM86475.1| serum amyloid A1 [synthetic construct] >gi|157928044|gb|ABW03318.1| serum amyloid A1 [synthetic construct] | 249.56 | 54.10% | 6 | 2 | 5 | 58 | 122 | 13.5 | 6.32 | 0.306 |
| 119626442 | multimerin 1, isoform CRA_a [Homo sapiens] | 62.55 | 3.58% | 6 | 1 | 1 | 5 | 531 | 58.1 | 8.72 | 0.312 |
| 17226634 | immunoglobulin heavy chain variable region [Homo sapiens] | 99.44 | 24.39% | 225 | 1 | 3 | 8 | 123 | 13.4 | 7.83 | 0.324 |
| 4505733 | platelet factor 4 precursor [Homo sapiens] >gi|130304|sp|P02776.2|PLF4_HUMAN RecName: Full=Platelet factor 4; Short=PF-4; AltName: Full=C-X-C motif chemokine 4; AltName: Full=Oncostatin-A; AltName: Full=Iroplact; Contains: RecName: Full=Platelet factor 4, short form; Flags: Precursor >gi|13549118|gb|AAK29643.1|AF349466_3 platelet factor 4 [Homo sapiens] >gi|189851|gb|AAA60066.1| platelet factor 4 [Homo sapiens] >gi|47115291|emb|CAG28605.1| PF4 [Homo sapiens] >gi|62739642|gb|AAH93965.1| Platelet factor 4 [Homo sapiens] >gi|63994325|gb|AAY41003.1| unknown [Homo sapiens] >gi|85567544|gb|AAI12094.1| Platelet factor 4 [Homo sapiens] >gi|119626099|gb|EAX05694.1| platelet factor 4 (chemokine (C-X-C motif) ligand 4) [Homo sapiens] >gi|261859268|dbj|BAI46156.1| platelet factor 4 [synthetic construct] | 125.57 | 34.65% | 1 | 1 | 3 | 22 | 101 | 10.8 | 8.62 | 0.35 |
| 158256710 | unnamed protein product [Homo sapiens] | 235.85 | 9.06% | 11 | 8 | 8 | 18 | 1170 | 129.3 | 4.94 | 0.401 |
| 183763 | factor H homologue [Homo sapiens] >gi|158255096|dbj|BAF83519.1| unnamed protein product [Homo sapiens] | 511 | 37.58% | 4 | 1 | 12 | 142 | 330 | 37.6 | 7.56 | 0.411 |
| 21669937 | immunoglobulin heavy chain VHDJ region [Homo sapiens] | 114.57 | 18.55% | 423 | 1 | 2 | 11 | 124 | 13.5 | 9.36 | 0.426 |
| 32483410 | vitamin D-binding protein precursor [Homo sapiens] | 803.24 | 58.86% | 3 | 1 | 22 | 75 | 474 | 52.9 | 5.45 | 0.445 |
| 221044784 | unnamed protein product [Homo sapiens] | 22.71 | 3.42% | 8 | 1 | 1 | 1 | 234 | 25.5 | 5.06 | 0.476 |
| 189066534 | unnamed protein product [Homo sapiens] | 76.02 | 20.00% | 4 | 2 | 2 | 3 | 175 | 20 | 9.01 | 0.485 |
| 183851 | G-gamma-hemoglobin [Homo sapiens] | 64.89 | 22.77% | 15 | 1 | 2 | 21 | 101 | 11 | 6.68 | 0.494 |
| 4505735 | platelet factor 4 variant [Homo sapiens] >gi|130306|sp|P10720.1|PF4V_HUMAN RecName: Full=Platelet factor 4 variant; AltName: Full=PF4var1; AltName: Full=PF4alt; AltName: Full=C-X-C motif chemokine 4 variant; AltName: Full=CXCL4L1; Contains: RecName: Full=Platelet factor 4 variant(4-74); Contains: RecName: Full=Platelet factor 4 variant(5-74); Contains: RecName: Full=Platelet factor 4 variant(6-74); Flags: Precursor >gi|292390|gb|AAA60067.1| platelet factor 4 [Homo sapiens] >gi|119626097|gb|EAX05692.1| platelet factor 4 variant 1 [Homo sapiens] >gi|120659980|gb|AAI30654.1| Platelet factor 4 variant 1 [Homo sapiens] >gi|120660120|gb|AAI30658.1| Platelet factor 4 variant 1 [Homo sapiens] | 168.33 | 48.08% | 1 | 2 | 4 | 9 | 104 | 11.5 | 9.1 | 0.502 |
| 34532317 | unnamed protein product [Homo sapiens] | 19.57 | 0.89% | 9 | 1 | 1 | 1 | 1014 | 113.4 | 9.03 | 0.507 |
| 587406 | immunoglobulin lambda chain variable region [Homo sapiens] | 78.84 | 27.27% | 244 | 1 | 2 | 16 | 121 | 12.7 | 5.31 | 0.538 |
| 31873302 | hypothetical protein [Homo sapiens] >gi|117646030|emb|CAL38482.1| hypothetical protein [synthetic construct] | 241.85 | 24.19% | 27 | 7 | 7 | 15 | 434 | 47.1 | 7.69 | 0.541 |
| 56378229 | carbamoylphosphate synthetase I [Homo sapiens] | 22.22 | 4.09% | 12 | 1 | 1 | 1 | 513 | 55.7 | 8.94 | 0.544 |
| 98956272 | immunoglobulin kappa light chain variable region [Homo sapiens] | 114.64 | 46.79% | 107 | 1 | 3 | 5 | 109 | 11.8 | 9.29 | 0.547 |
| 139641 | RecName: Full=Vitamin D-binding protein; Short=DBP; Short=VDB; AltName: Full=Group-specific component; AltName: Full=Gc-globulin; Flags: Precursor >gi|31676|emb|CAA26938.1| unnamed protein product [Homo sapiens] | 812.8 | 58.44% | 1 | 1 | 22 | 73 | 474 | 52.9 | 5.54 | 0.559 |
| 1064908 | complement Factor H-related Protein 2 [Homo sapiens] | 222.48 | 31.28% | 3 | 3 | 6 | 38 | 243 | 27.9 | 6.92 | 0.575 |
| 119594857 | cofilin 1 (non-muscle), isoform CRA_c [Homo sapiens] | 91.11 | 40.88% | 6 | 4 | 4 | 5 | 137 | 15.6 | 8.35 | 0.579 |
| 119608546 | ficolin (collagen/fibrinogen domain containing) 1, isoform CRA_d [Homo sapiens] | 57.63 | 5.10% | 7 | 1 | 2 | 2 | 314 | 33.8 | 6.64 | 0.583 |
| 3337390 | haptoglobin [Homo sapiens] | 399.49 | 39.42% | 15 | 5 | 12 | 56 | 345 | 38.2 | 6.6 | 0.599 |
| 1769552 | von Willebrand factor [Homo sapiens] | 27.48 | 3.73% | 10 | 1 | 1 | 2 | 241 | 27.1 | 6.93 | 0.606 |
| 54304028 | glyceraldehyde-3-phosphate dehydrogenase [Homo sapiens] | 45.44 | 17.44% | 7 | 1 | 1 | 3 | 86 | 9.2 | 9.72 | 0.629 |
| 119588814 | serum amyloid A1, isoform CRA_a [Homo sapiens] >gi|119588815|gb|EAW68409.1| serum amyloid A1, isoform CRA_a [Homo sapiens] >gi|119588817|gb|EAW68411.1| serum amyloid A1, isoform CRA_a [Homo sapiens] | 350.08 | 54.10% | 9 | 3 | 6 | 71 | 122 | 13.6 | 6.79 | 0.63 |
| 10636616 | immunoglobulin heavy chain variable region [Homo sapiens] | 89.37 | 18.97% | 627 | 1 | 2 | 22 | 116 | 12.9 | 7.85 | 0.639 |
| 4758146 | neutrophil defensin 1 preproprotein [Homo sapiens] >gi|124248516|ref|NP_001035965.1| alpha-defensin 1 [Homo sapiens] >gi|30316322|sp|P59665.1|DEF1_HUMAN RecName: Full=Neutrophil defensin 1; AltName: Full=HNP-1; Short=HP-1; Short=HP1; AltName: Full=Defensin, alpha 1; Contains: RecName: Full=HP 1-56; Contains: RecName: Full=Neutrophil defensin 2; AltName: Full=HNP-2; Short=HP-2; Short=HP2; Flags: Precursor >gi|32402|emb|CAA36280.1| unnamed protein product [Homo sapiens] >gi|181527|gb|AAA52302.1| neutrophil peptide 1 precursor [Homo sapiens] >gi|181529|gb|AAA52303.1| defensin 1 [Homo sapiens] >gi|292363|gb|AAA36382.1| neutrophil peptide-1 [Homo sapiens] >gi|46854592|gb|AAH69423.1| Defensin, alpha 1 [Homo sapiens] >gi|50057839|gb|AAT68875.1| novel protein, similar to DEFA1 [Homo sapiens] >gi|50057842|gb|AAT68878.1| defensin, alpha 1, myeloid-related sequence [Homo sapiens] >gi|50057843|gb|AAT68879.1| novel protein, similar to DEFA1 [Homo sapiens] >gi|50057844|gb|AAT68880.1| novel protein, similar to DEFA1 [Homo sapiens] >gi|50057847|gb|AAT68883.1| defensin, alpha 1, myeloid-related sequence [Homo sapiens] >gi|50057848|gb|AAT68884.1| defensin, alpha 1, myeloid-related sequence [Homo sapiens] >gi|62739977|gb|AAH93791.1| Defensin, alpha 1 [Homo sapiens] >gi|85567619|gb|AAI12189.1| Defensin, alpha 1, preproprotein [Homo sapiens] >gi|446635|prf||1912193A defensin >gi|1098031|prf||2115200A neutrophil peptide | 43.25 | 32.98% | 2 | 1 | 1 | 2 | 94 | 10.2 | 6.99 | 0.642 |
| 148733226 | serpin peptidase inhibitor, clade A (alpha-1 antiproteinase, antitrypsin), member 10 [Homo sapiens] | 73.38 | 8.11% | 5 | 3 | 3 | 7 | 444 | 50.7 | 8.28 | 0.647 |
| 247425006 | immunoglobulin heavy chain variable region [Homo sapiens] | 179.48 | 39.34% | 421 | 1 | 4 | 50 | 122 | 13.1 | 8.81 | 0.649 |
| 71773201 | adenine phosphoribosyltransferase isoform b [Homo sapiens] >gi|114664113|ref|XP_001137924.1| PREDICTED: adenine phosphoribosyltransferase isoform 1 [Pan troglodytes] >gi|119587165|gb|EAW66761.1| adenine phosphoribosyltransferase, isoform CRA_b [Homo sapiens] | 26.87 | 14.93% | 2 | 1 | 1 | 1 | 134 | 14.5 | 7.34 | 0.659 |
| 2258128 | complement 9 [Homo sapiens] | 51.17 | 4.50% | 5 | 2 | 2 | 3 | 533 | 60.4 | 5.59 | 0.661 |
| 47124510 | APCS protein [Homo sapiens] | 30.38 | 23.91% | 3 | 1 | 1 | 2 | 46 | 5.3 | 4.46 | 0.676 |
| 119584204 | fibrinogen-like 1, isoform CRA_b [Homo sapiens] | 65.43 | 12.36% | 9 | 2 | 2 | 6 | 178 | 21 | 5.97 | 0.687 |
| 284434903 | thrombocidin-2 antimicrobial variant [Homo sapiens] | 56.55 | 19.84% | 4 | 2 | 2 | 3 | 126 | 13.7 | 9 | 0.689 |
| 126273569 | carboxypeptidase B2 isoform a preproprotein [Homo sapiens] >gi|62899885|sp|Q96IY4.1|CBPB2_HUMAN RecName: Full=Carboxypeptidase B2; AltName: Full=Carboxypeptidase U; Short=CPU; AltName: Full=Thrombin-activable fibrinolysis inhibitor; Short=TAFI; AltName: Full=Plasma carboxypeptidase B; Short=pCPB; Flags: Precursor >gi|13937897|gb|AAH07057.1| Carboxypeptidase B2 (plasma) [Homo sapiens] >gi|30582711|gb|AAP35582.1| carboxypeptidase B2 (plasma, carboxypeptidase U) [Homo sapiens] >gi|51234145|gb|AAT97987.1| carboxypeptidase B2 (plasma, carboxypeptidase U) [Homo sapiens] >gi|60656513|gb|AAX32820.1| carboxypeptidase B2 [synthetic construct] >gi|60656515|gb|AAX32821.1| carboxypeptidase B2 [synthetic construct] >gi|119629160|gb|EAX08755.1| carboxypeptidase B2 (plasma, carboxypeptidase U), isoform CRA_b [Homo sapiens] | 34.26 | 1.89% | 3 | 1 | 1 | 3 | 423 | 48.4 | 7.71 | 0.689 |
| 146424184 | apolipoprotein C-IV [Homo sapiens] | 73.46 | 15.75% | 3 | 2 | 2 | 5 | 127 | 14.6 | 9.13 | 0.691 |
| 119570830 | hCG1741471 [Homo sapiens] | 23 | 3.11% | 7 | 1 | 1 | 2 | 322 | 37.2 | 7.99 | 0.692 |
| 194273292 | immunoglobulin heavy chain variable region [Homo sapiens] | 227.62 | 70.30% | 896 | 1 | 6 | 13 | 101 | 11 | 9.72 | 0.693 |
| 49354849 | immunoglobulin E variable region [Homo sapiens] | 135.35 | 38.02% | 272 | 1 | 3 | 19 | 121 | 13.2 | 8.43 | 0.695 |
| 37789448 | immunoglobulin lambda light chain variable region [Homo sapiens] | 21.04 | 9.64% | 4 | 1 | 1 | 1 | 83 | 8.9 | 7.12 | 0.695 |
| 184086 | histone H2B.1 [Homo sapiens] | 30.74 | 8.91% | 34 | 1 | 1 | 6 | 101 | 11.3 | 10.14 | 0.697 |
| 21669509 | immunoglobulin lambda light chain VLJ region [Homo sapiens] | 302.26 | 34.43% | 250 | 1 | 7 | 501 | 273 | 28.2 | 7.14 | 0.698 |
| 194375299 | unnamed protein product [Homo sapiens] | 265.15 | 33.33% | 58 | 8 | 8 | 22 | 333 | 37.3 | 5.71 | 0.704 |
| 1655598 | lipopolysaccharide binding protein [Homo sapiens] >gi|4530277|gb|AAD21962.1| lipopolysaccharide-binding protein [Homo sapiens] | 464.59 | 23.70% | 7 | 10 | 10 | 75 | 481 | 53.3 | 6.7 | 0.708 |
| 54780230 | immunoglobulin mu heavy chain [Homo sapiens] | 161.81 | 19.43% | 633 | 1 | 4 | 89 | 211 | 22.7 | 8.31 | 0.713 |
| 10835095 | serum amyloid A-4 protein precursor [Homo sapiens] >gi|259352|gb|AAB24060.1| serum amyloid A [Homo sapiens] >gi|337750|gb|AAA60298.1| serum amyloid A protein [Homo sapiens] >gi|13937846|gb|AAH07026.1| Serum amyloid A4, constitutive [Homo sapiens] >gi|49456475|emb|CAG46558.1| SAA4 [Homo sapiens] >gi|119588821|gb|EAW68415.1| serum amyloid A4, constitutive [Homo sapiens] | 94.08 | 23.08% | 2 | 3 | 3 | 18 | 130 | 14.8 | 9.23 | 0.72 |
| 158255874 | unnamed protein product [Homo sapiens] | 487.27 | 17.28% | 9 | 12 | 12 | 53 | 1065 | 122.1 | 5.74 | 0.721 |
| 3152372 | anti-FactorVIII scFv [Homo sapiens] | 537.51 | 64.29% | 3758 | 1 | 10 | 90 | 238 | 25 | 8.41 | 0.726 |
| 77379566 | immunoglobulin kappa chain variable region [Homo sapiens] | 76.5 | 22.22% | 418 | 1 | 2 | 25 | 108 | 12 | 8.81 | 0.727 |
| 119598593 | alpha-2-HS-glycoprotein, isoform CRA_a [Homo sapiens] | 532.05 | 46.99% | 2 | 1 | 11 | 318 | 366 | 39.2 | 5.72 | 0.727 |
| 7770217 | PRO2675 [Homo sapiens] >gi|119626082|gb|EAX05677.1| albumin, isoform CRA_s [Homo sapiens] | 939.35 | 78.47% | 5 | 1 | 22 | 318 | 288 | 32.6 | 6.54 | 0.73 |
| 105990532 | apolipoprotein B-100 precursor [Homo sapiens] >gi|260158878|gb|ACX32319.1| apolipoprotein B precursor [synthetic construct] | 2158.39 | 18.58% | 36 | 5 | 63 | 209 | 4563 | 515.2 | 7.05 | 0.73 |
| 194385488 | unnamed protein product [Homo sapiens] | 67.07 | 4.23% | 18 | 2 | 2 | 2 | 780 | 82.5 | 6.55 | 0.733 |
| 5459317 | mannose binding lectin-associated serine protease-2 related protein, MAp19 (19kDa) [Homo sapiens] | 134.2 | 28.00% | 11 | 4 | 4 | 8 | 175 | 19.5 | 5.73 | 0.74 |
| 98956202 | immunoglobulin kappa light chain variable region [Homo sapiens] | 132.02 | 52.17% | 497 | 1 | 3 | 31 | 92 | 10.1 | 5.94 | 0.743 |
| 115298678 | complement C3 precursor [Homo sapiens] >gi|119370332|sp|P01024.2|CO3_HUMAN RecName: Full=Complement C3; AltName: Full=C3 and PZP-like alpha-2-macroglobulin domain-containing protein 1; Contains: RecName: Full=Complement C3 beta chain; Contains: RecName: Full=Complement C3 alpha chain; Contains: RecName: Full=C3a anaphylatoxin; Contains: RecName: Full=Complement C3b alpha' chain; Contains: RecName: Full=Complement C3c alpha' chain fragment 1; Contains: RecName: Full=Complement C3dg fragment; Contains: RecName: Full=Complement C3g fragment; Contains: RecName: Full=Complement C3d fragment; Contains: RecName: Full=Complement C3f fragment; Contains: RecName: Full=Complement C3c alpha' chain fragment 2; Flags: Precursor >gi|40786791|gb|AAR89906.1| complement component 3 [Homo sapiens] >gi|119589477|gb|EAW69071.1| complement component 3, isoform CRA_b [Homo sapiens] >gi|152012494|gb|AAI50201.1| Complement component 3 [Homo sapiens] >gi|152012784|gb|AAI50180.1| Complement component 3 [Homo sapiens] | 3018.01 | 58.15% | 7 | 76 | 76 | 444 | 1663 | 187 | 6.4 | 0.747 |
| 194385606 | unnamed protein product [Homo sapiens] | 92.97 | 6.39% | 9 | 3 | 3 | 5 | 626 | 70.3 | 5.34 | 0.75 |
| 189054579 | unnamed protein product [Homo sapiens] | 40.06 | 7.67% | 2 | 2 | 2 | 3 | 352 | 38.2 | 7.11 | 0.753 |
| 178751 | alpha-2-antiplasmin precursor [Homo sapiens] | 359.91 | 25.82% | 9 | 9 | 9 | 24 | 488 | 54.2 | 6.07 | 0.754 |
| 119573007 | apolipoprotein A-II, isoform CRA_d [Homo sapiens] | 478.54 | 75.00% | 4 | 2 | 9 | 695 | 92 | 10.6 | 9.04 | 0.759 |
| 30583505 | catenin (cadherin-associated protein), delta 1 [Homo sapiens] | 42.11 | 3.28% | 25 | 1 | 1 | 1 | 610 | 68 | 8.13 | 0.761 |
| 170684488 | immunoglobulin lambda 1 light chain [Homo sapiens] | 195.72 | 32.87% | 43 | 1 | 4 | 366 | 216 | 22.6 | 7.33 | 0.762 |
| 896272 | This CDS feature is included to show the translation of the corresponding V_region. Presently translation qualifiers on V_region features are illegal [Homo sapiens] | 285.25 | 64.57% | 2946 | 2 | 9 | 63 | 127 | 13.8 | 8.79 | 0.765 |
| 2809025 | Ig heavy chain variable region [Homo sapiens] | 41.55 | 42.25% | 134 | 1 | 2 | 4 | 71 | 7.9 | 9.31 | 0.774 |
| 10437873 | unnamed protein product [Homo sapiens] | 33.85 | 7.46% | 9 | 1 | 1 | 3 | 362 | 41.6 | 5.01 | 0.776 |
| 194383496 | unnamed protein product [Homo sapiens] | 925.68 | 45.45% | 12 | 1 | 21 | 152 | 605 | 68.3 | 5.82 | 0.784 |
| 13477169 | Vitronectin [Homo sapiens] >gi|119571467|gb|EAW51082.1| vitronectin [Homo sapiens] >gi|123993437|gb|ABM84320.1| vitronectin [synthetic construct] >gi|124000413|gb|ABM87715.1| vitronectin [synthetic construct] | 553.88 | 30.33% | 5 | 11 | 11 | 265 | 478 | 54.3 | 5.8 | 0.785 |
| 119625336 | fibrinogen beta chain, isoform CRA_b [Homo sapiens] | 570.94 | 53.04% | 3 | 4 | 14 | 328 | 247 | 27.7 | 7.99 | 0.789 |
| 119608880 | hCG1979429, isoform CRA_a [Homo sapiens] | 16.53 | 0.69% | 1 | 1 | 1 | 1 | 873 | 98.1 | 9.29 | 0.792 |
| 112700542 | immunoglobulin heavy chain variable region [Homo sapiens] | 154.72 | 43.56% | 1441 | 1 | 4 | 24 | 101 | 10.9 | 8.29 | 0.796 |
| 221042312 | unnamed protein product [Homo sapiens] | 233.94 | 15.07% | 15 | 4 | 4 | 12 | 564 | 60 | 5.74 | 0.8 |
| 87299000 | immunoglobulin light chain variable region [Homo sapiens] | 120.82 | 37.19% | 40 | 1 | 3 | 9 | 121 | 13.2 | 8.91 | 0.8 |
| 40737478 | C4A3 [Homo sapiens] >gi|40737480|gb|AAR89158.1| C4A [Homo sapiens] >gi|40737484|gb|AAR89160.1| C4A3 [Homo sapiens] | 933.23 | 54.49% | 13 | 1 | 21 | 260 | 534 | 58.4 | 5.99 | 0.805 |
| 119625314 | fibrinogen gamma chain, isoform CRA_e [Homo sapiens] | 1193.9 | 62.53% | 5 | 6 | 27 | 1576 | 419 | 47.4 | 5.95 | 0.807 |
| 189053338 | unnamed protein product [Homo sapiens] | 25.83 | 4.48% | 5 | 1 | 1 | 3 | 201 | 23.6 | 5.21 | 0.807 |
| 119590943 | fibronectin 1, isoform CRA_h [Homo sapiens] | 2674.5 | 37.42% | 82 | 36 | 60 | 502 | 2330 | 256.3 | 5.8 | 0.81 |
| 5174411 | CD5 antigen-like precursor [Homo sapiens] >gi|20177834|sp|O43866.1|CD5L_HUMAN RecName: Full=CD5 antigen-like; AltName: Full=SP-alpha; AltName: Full=CT-2; AltName: Full=IgM-associated peptide; Flags: Precursor >gi|2702314|gb|AAB91989.1| Sp alpha [Homo sapiens] >gi|4102235|gb|AAD01446.1| AIM [Homo sapiens] >gi|11967471|emb|CAC19458.1| CD5 molecule-like [Homo sapiens] >gi|21707924|gb|AAH33586.1| CD5 molecule-like [Homo sapiens] >gi|119573244|gb|EAW52859.1| CD5 antigen-like (scavenger receptor cysteine rich family) [Homo sapiens] >gi|123993879|gb|ABM84541.1| CD5 molecule-like [synthetic construct] >gi|123997245|gb|ABM86224.1| CD5 molecule-like [synthetic construct] >gi|158257512|dbj|BAF84729.1| unnamed protein product [Homo sapiens] | 466.86 | 47.84% | 2 | 14 | 14 | 57 | 347 | 38.1 | 5.47 | 0.81 |
| 207028494 | L-lactate dehydrogenase A chain isoform 2 [Homo sapiens] >gi|194383812|dbj|BAG59264.1| unnamed protein product [Homo sapiens] | 36.64 | 7.30% | 5 | 1 | 1 | 3 | 274 | 30.2 | 7.15 | 0.812 |
| 194388758 | unnamed protein product [Homo sapiens] | 70.32 | 11.19% | 18 | 3 | 3 | 5 | 277 | 30.8 | 8.66 | 0.814 |
| 178812 | apolipoprotein B-100 precursor [Homo sapiens] | 1998.27 | 17.34% | 21 | 1 | 59 | 189 | 4563 | 515.1 | 7.11 | 0.815 |
| 118406205 | immunoglobulin heavy chain variable region [Homo sapiens] | 58.92 | 22.22% | 231 | 1 | 2 | 7 | 117 | 12.5 | 8.68 | 0.817 |
| 553293 | fibronectin [Homo sapiens] | 54.43 | 36.73% | 1 | 1 | 1 | 3 | 49 | 5 | 11.11 | 0.821 |
| 180271 | cholesteryl ester transfer protein [Homo sapiens] | 130.3 | 8.00% | 7 | 3 | 3 | 4 | 425 | 47 | 6.01 | 0.826 |
| 47132620 | keratin, type II cytoskeletal 2 epidermal [Homo sapiens] >gi|239938650|sp|P35908.2|K22E_HUMAN RecName: Full=Keratin, type II cytoskeletal 2 epidermal; AltName: Full=Cytokeratin-2e; Short=CK-2e; AltName: Full=Keratin-2e; Short=K2e; AltName: Full=Type-II keratin Kb2; AltName: Full=Keratin-2 epidermis; AltName: Full=Epithelial keratin-2e >gi|64654451|gb|AAH96294.1| Keratin 2 [Homo sapiens] >gi|68563334|gb|AAH99644.1| Keratin 2 [Homo sapiens] >gi|68563400|gb|AAH99643.1| Keratin 2 [Homo sapiens] >gi|119617047|gb|EAW96641.1| keratin 2A (epidermal ichthyosis bullosa of Siemens) [Homo sapiens] | 343.89 | 23.00% | 45 | 8 | 10 | 19 | 639 | 65.4 | 8 | 0.828 |
| 224979536 | anti-IL-15 immunoglobulin heavy chain variable region 1 [Homo sapiens] | 21.9 | 16.10% | 1 | 1 | 1 | 2 | 118 | 12.7 | 7.12 | 0.828 |
| 74355107 | BRF1 protein [Homo sapiens] | 30.09 | 4.97% | 11 | 1 | 1 | 11 | 161 | 18.3 | 8.62 | 0.828 |
| 110589608 | anti-streptococcal/anti-lysoganglioside immunoglobulin heavy chain variable region [Homo sapiens] | 13.91 | 17.76% | 6 | 1 | 1 | 1 | 107 | 11.5 | 10.2 | 0.829 |
| 76252669 | immunoglobulin lambda light chain variable region [Homo sapiens] | 33.83 | 17.65% | 2 | 1 | 1 | 4 | 102 | 10.9 | 7.12 | 0.831 |
| 182442 | gamma fibrinogen type A (AA at 202) [Homo sapiens] | 226.09 | 38.56% | 3 | 1 | 5 | 129 | 153 | 17.1 | 5.77 | 0.831 |
| 247424903 | immunoglobulin heavy chain variable region [Homo sapiens] | 286.5 | 51.22% | 1252 | 1 | 5 | 69 | 123 | 13.4 | 7.96 | 1.2 |
| 37694587 | immunoglobulin heavy chain variable region [Homo sapiens] | 81.8 | 25.64% | 12 | 1 | 2 | 5 | 117 | 12.9 | 7.39 | 1.2 |
| 170684552 | immunoglobulin lambda 1 light chain [Homo sapiens] | 473.86 | 54.63% | 358 | 1 | 11 | 749 | 216 | 22.9 | 6.3 | 1.2 |
| 77379581 | immunoglobulin kappa chain variable region [Homo sapiens] | 50.81 | 32.04% | 106 | 1 | 2 | 2 | 103 | 11.2 | 8.91 | 1.201 |
| 55669575 | Chain A, A Covalent Dimer Of Transthyretin That Affects The Amyloid Pathway >gi|55669576|pdb|1QWH|B Chain B, A Covalent Dimer Of Transthyretin That Affects The Amyloid Pathway >gi|339685|gb|AAA61181.1| transthyretin [Homo sapiens] | 291.36 | 66.67% | 11 | 7 | 7 | 86 | 117 | 12.8 | 5.45 | 1.207 |
| 1369904 | serum lectin P35 [Homo sapiens] >gi|1669349|dbj|BAA09636.1| lectin P35 [Homo sapiens] | 342.33 | 30.99% | 6 | 8 | 9 | 95 | 313 | 34 | 6.54 | 1.209 |
| 57162363 | KIAA0515 [Homo sapiens] | 20.36 | 1.53% | 8 | 1 | 1 | 1 | 587 | 63.2 | 6.73 | 1.216 |
| 190609605 | immunoglobulin kappa light chain virable region [Homo sapiens] | 48.36 | 18.75% | 2 | 1 | 1 | 4 | 96 | 10.4 | 8.5 | 1.221 |
| 81295708 | immunoglobulin light chain variable region [Homo sapiens] | 151.35 | 37.72% | 120 | 1 | 4 | 9 | 114 | 12.1 | 8.02 | 1.226 |
| 3153306 | immunoglobulin light chain variable region [Homo sapiens] | 130.73 | 33.87% | 80 | 2 | 5 | 14 | 124 | 12.9 | 8.5 | 1.231 |
| 189066554 | unnamed protein product [Homo sapiens] | 924.02 | 44.21% | 5 | 1 | 21 | 151 | 622 | 70 | 5.9 | 1.239 |
| 18041890 | immunoglobulin lambda light chain variable region [Homo sapiens] | 35.59 | 9.09% | 3 | 1 | 1 | 7 | 88 | 9.2 | 5.24 | 1.243 |
| 12733966 | immunoglobulin gamma heavy chain variable region [Homo sapiens] | 167.4 | 39.32% | 1183 | 1 | 4 | 41 | 117 | 13 | 6.04 | 1.247 |
| 4502067 | protein AMBP preproprotein [Homo sapiens] >gi|122801|sp|P02760.1|AMBP_HUMAN RecName: Full=Protein AMBP; Contains: RecName: Full=Alpha-1-microglobulin; Short=Protein HC; AltName: Full=Complex-forming glycoprotein heterogeneous in charge; AltName: Full=Alpha-1 microglycoprotein; Contains: RecName: Full=Inter-alpha-trypsin inhibitor light chain; Short=ITI-LC; AltName: Full=Bikunin; AltName: Full=HI-30; AltName: Full=Uronic-acid-rich protein; AltName: Full=EDC1; Contains: RecName: Full=Trypstatin; Flags: Precursor >gi|24479|emb|CAA28182.1| unnamed protein product [Homo sapiens] >gi|32047|emb|CAA27803.1| HC polypeptide [Homo sapiens] >gi|186600|gb|AAA59196.1| inter-alpha-trypsin inhibitor light chain [Homo sapiens] >gi|825614|emb|CAA38585.1| alpha1-microglobulin; bikunin [Homo sapiens] >gi|27371332|gb|AAH41593.1| Alpha-1-microglobulin/bikunin precursor [Homo sapiens] >gi|47118013|gb|AAT11154.1| growth-inhibiting protein 19 [Homo sapiens] >gi|55957384|emb|CAI15899.1| alpha-1-microglobulin/bikunin precursor [Homo sapiens] >gi|119607810|gb|EAW87404.1| alpha-1-microglobulin/bikunin precursor [Homo sapiens] >gi|158255110|dbj|BAF83526.1| unnamed protein product [Homo sapiens] >gi|208965810|dbj|BAG72919.1| alpha-1-microglobulin/bikunin precursor [synthetic construct] | 476.46 | 33.52% | 4 | 11 | 11 | 51 | 352 | 39 | 6.25 | 1.25 |
| 219566101 | immunoglobulin heavy chain [Homo sapiens] | 75.42 | 22.39% | 9 | 1 | 2 | 3 | 134 | 14.7 | 5.48 | 1.251 |
| 9295301 | immunoglobulin light chain variable region [Homo sapiens] | 109.65 | 26.13% | 23 | 1 | 2 | 26 | 111 | 12.3 | 8.85 | 1.252 |
| 18025670 | immunoglobulin light chain variable region [Homo sapiens] | 66.85 | 25.23% | 17 | 1 | 2 | 4 | 107 | 11.8 | 9.01 | 1.255 |
| 27369048 | immunoglobulin lambda light chain variable region [Homo sapiens] | 10.42 | 9.29% | 1 | 1 | 1 | 1 | 140 | 14.5 | 7.06 | 1.266 |
| 37287526 | paraneoplastic pemphigus associated Castleman's disease immunoglobulin light chain variable region [Homo sapiens] | 24.41 | 15.93% | 1 | 1 | 1 | 1 | 113 | 12.6 | 7.12 | 1.282 |
| 77378386 | immunoglobulin lambda light chain variable region [Homo sapiens] | 57.61 | 36.89% | 113 | 1 | 2 | 7 | 122 | 12.6 | 8.5 | 1.283 |
| 119585666 | inter-alpha (globulin) inhibitor H3, isoform CRA_a [Homo sapiens] | 99.6 | 5.07% | 7 | 3 | 3 | 17 | 670 | 75 | 5.94 | 1.318 |
| 5051025 | immunoglobulin gamma heavy chain variable region [Homo sapiens] | 96.49 | 21.88% | 47 | 1 | 2 | 18 | 128 | 14.4 | 5.1 | 1.322 |
| 215982760 | immunoglobulin lambda light chain variable region [Homo sapiens] | 150.34 | 27.66% | 13 | 1 | 3 | 214 | 141 | 15.1 | 5.45 | 1.327 |
| 7012705 | immunoglobulin light chain variable region [Homo sapiens] | 212.91 | 68.22% | 425 | 1 | 4 | 27 | 107 | 11.5 | 7.96 | 1.329 |
| 158258641 | unnamed protein product [Homo sapiens] | 22.67 | 5.86% | 14 | 1 | 1 | 1 | 222 | 25.7 | 9 | 1.335 |
| 722568 | immunoglobulin kappa chain [Homo sapiens] | 65.57 | 30.30% | 16 | 1 | 2 | 2 | 99 | 10.8 | 8.48 | 1.339 |
| 189067487 | unnamed protein product [Homo sapiens] | 34.33 | 3.61% | 2 | 1 | 1 | 1 | 527 | 59.7 | 7.24 | 1.355 |
| 21669515 | immunoglobulin lambda light chain VLJ region [Homo sapiens] | 437.27 | 40.89% | 491 | 1 | 10 | 746 | 269 | 28 | 6.74 | 1.357 |
| 33319680 | Ig heavy chain variable region, VH3 family [Homo sapiens] | 172.21 | 39.34% | 1007 | 1 | 4 | 29 | 122 | 13.1 | 8.85 | 1.372 |
| 119608489 | ADAM metallopeptidase with thrombospondin type 1 motif, 13, isoform CRA_a [Homo sapiens] >gi|119608490|gb|EAW88084.1| ADAM metallopeptidase with thrombospondin type 1 motif, 13, isoform CRA_a [Homo sapiens] >gi|119608491|gb|EAW88085.1| ADAM metallopeptidase with thrombospondin type 1 motif, 13, isoform CRA_a [Homo sapiens] | 106.05 | 4.80% | 8 | 3 | 3 | 4 | 1041 | 112.4 | 7.31 | 1.372 |
| 114147465 | immunoglobulin heavy chain variable region [Homo sapiens] | 85.07 | 25.00% | 742 | 1 | 2 | 23 | 132 | 14.3 | 8.43 | 1.38 |
| 4321593 | immunoglobulin G kappa chain [Homo sapiens] | 361.42 | 38.25% | 71 | 1 | 5 | 126 | 217 | 24 | 6.55 | 1.394 |
| 4557389 | complement component C8 alpha chain precursor [Homo sapiens] >gi|729167|sp|P07357.2|CO8A_HUMAN RecName: Full=Complement component C8 alpha chain; AltName: Full=Complement component 8 subunit alpha; Flags: Precursor >gi|179718|gb|AAA52200.1| complement protein C8 alpha subunit precursor [Homo sapiens] >gi|56203387|emb|CAI19172.1| complement component 8, alpha polypeptide [Homo sapiens] >gi|124376216|gb|AAI32914.1| Complement component 8, alpha polypeptide [Homo sapiens] | 83.59 | 7.71% | 7 | 3 | 3 | 4 | 584 | 65.1 | 6.47 | 1.416 |
| 194389616 | unnamed protein product [Homo sapiens] | 23.6 | 2.10% | 28 | 1 | 1 | 1 | 525 | 59.7 | 7.12 | 1.445 |
| 157778872 | immunoglobulin heavy chain variable region [Homo sapiens] | 45.74 | 17.39% | 231 | 1 | 2 | 7 | 115 | 12.8 | 9.01 | 1.445 |
| 11122875 | glycosylphosphatidylinositol phospholipase D [Homo sapiens] | 137.2 | 8.32% | 8 | 3 | 3 | 4 | 457 | 48.5 | 6.2 | 1.446 |
| 20377087 | intestinal lactoferrin receptor [Homo sapiens] >gi|7019846|dbj|BAA90893.1| unnamed protein product [Homo sapiens] >gi|8096221|dbj|BAA96094.1| intelectin [Homo sapiens] >gi|18091783|gb|AAL58073.1| endothelial lectin HL-1 [Homo sapiens] >gi|37181843|gb|AAQ88725.1| ITLN [Homo sapiens] >gi|37724012|gb|AAO17801.1| intelectin [Homo sapiens] >gi|44976129|gb|AAS49907.1| omentin [Homo sapiens] >gi|48146565|emb|CAG33505.1| ITLN1 [Homo sapiens] >gi|52843233|gb|AAU88047.1| intelectin 1 [Homo sapiens] >gi|62896625|dbj|BAD96253.1| intelectin variant [Homo sapiens] | 171.75 | 29.07% | 7 | 7 | 7 | 13 | 313 | 35 | 5.82 | 1.446 |
| 114665902 | PREDICTED: similar to Profilin-1 (Profilin I) [Pan troglodytes] >gi|119610788|gb|EAW90382.1| profilin 1, isoform CRA_b [Homo sapiens] | 41.59 | 7.69% | 2 | 1 | 1 | 1 | 104 | 11.4 | 9.17 | 1.448 |
| 1905799 | immunogloblin light chain [Homo sapiens] | 197.3 | 44.95% | 242 | 1 | 3 | 27 | 109 | 11.7 | 7.28 | 1.451 |
| 111917949 | immunoglobulin heavy chain variable region [Homo sapiens] | 76.7 | 18.18% | 77 | 1 | 2 | 8 | 154 | 16.7 | 7.84 | 1.459 |
| 186083 | immunoglobulin lambda-chain [Homo sapiens] | 82.87 | 21.17% | 350 | 1 | 3 | 29 | 137 | 14.3 | 7.24 | 1.49 |
| 21669521 | immunoglobulin lambda light chain VLJ region [Homo sapiens] | 493.89 | 40.52% | 420 | 1 | 11 | 747 | 269 | 27.9 | 7.91 | 1.497 |
| 542891 | Ig kappa chain V-J region - human >gi|441433|emb|CAA51150.1| Ig kappa light chain (VJ) [Homo sapiens] | 84.79 | 29.77% | 418 | 1 | 2 | 25 | 131 | 14.2 | 6.51 | 1.527 |
| 221044282 | unnamed protein product [Homo sapiens] | 229.04 | 9.77% | 4 | 5 | 5 | 15 | 870 | 95.9 | 4.64 | 1.536 |
| 18307322 | immunoglobulin light chain lambda variable region [Homo sapiens] | 27.87 | 12.62% | 1 | 1 | 1 | 1 | 103 | 10.8 | 5.25 | 1.546 |
| 553490 | immunoglobulin lambda-chain [Homo sapiens] | 64.73 | 12.50% | 89 | 1 | 2 | 7 | 128 | 13.4 | 7.81 | 1.562 |
| 21669285 | immunoglobulin lambda light chain VLJ region [Homo sapiens] | 43.55 | 32.43% | 4 | 1 | 2 | 3 | 111 | 11.7 | 8.91 | 1.572 |
| 70798731 | immunoglobulin kappa light chain variable region [Homo sapiens] | 154.27 | 53.42% | 425 | 1 | 3 | 25 | 73 | 8 | 8.53 | 1.654 |
| 8777875 | immunoglobulin light chain variable region [Homo sapiens] | 174.72 | 37.61% | 423 | 1 | 3 | 26 | 109 | 12 | 8.48 | 1.659 |
| 553426 | immunoglobulin heavy chain VDJC region [Homo sapiens] | 103.19 | 14.58% | 39 | 1 | 3 | 36 | 144 | 15.6 | 9.13 | 1.668 |
| 37777898 | immunoglobulin heavy chain variable region [Homo sapiens] | 196.03 | 32.03% | 213 | 1 | 4 | 61 | 153 | 16.5 | 8.81 | 1.732 |
| 1620396 | haptoglobin [Homo sapiens] | 370.62 | 34.77% | 7 | 4 | 11 | 42 | 348 | 39 | 6.89 | 1.753 |
| 3004726 | IgM heavy chain variable region [Homo sapiens] | 146.27 | 43.65% | 494 | 1 | 4 | 12 | 126 | 14 | 7.91 | 1.753 |
| 33319650 | Ig heavy chain variable region, VH3 family [Homo sapiens] | 100.27 | 34.17% | 151 | 1 | 3 | 5 | 120 | 13.1 | 6.52 | 1.825 |
| 119600101 | phosphatidylinositol-specific phospholipase C, X domain containing 2 [Homo sapiens] | 34.44 | 2.42% | 3 | 1 | 1 | 1 | 289 | 33 | 9.44 | 1.841 |
| 58223278 | anti-tetanus toxoid immunoglobulin light chain variable region [Homo sapiens] | 269.33 | 82.41% | 704 | 1 | 5 | 33 | 108 | 11.5 | 7.96 | 1.845 |
| 34534492 | unnamed protein product [Homo sapiens] | 13.39 | 8.66% | 1 | 1 | 1 | 1 | 277 | 30.5 | 8.56 | 1.869 |
| 109240688 | immunoglobulin kappa chain variable region [Homo sapiens] | 41.25 | 15.09% | 2 | 1 | 1 | 3 | 106 | 11.7 | 7.99 | 1.906 |
| 189231244 | anti-fluorescein immunoglobulin heavy chain variable region [Homo sapiens] | 128.41 | 33.61% | 369 | 1 | 3 | 12 | 119 | 12.9 | 8.46 | 1.922 |
| 77379428 | immunoglobulin kappa chain variable region [Homo sapiens] | 38.02 | 21.49% | 1 | 1 | 1 | 2 | 121 | 13.1 | 6 | 1.927 |
| 47271320 | immunoglobulin lambda light chain variable region [Homo sapiens] | 59.98 | 21.05% | 104 | 1 | 2 | 5 | 114 | 12.1 | 5.01 | 1.93 |
| 346196 | Ig lambda chain V region - human >gi|452933|gb|AAB28790.1| anti-cardiolipin/beta 2 glycoprotein I immunoglobulin light chain variable region [Homo sapiens] | 102.42 | 21.67% | 284 | 1 | 3 | 22 | 120 | 12.4 | 6.48 | 1.94 |
| 21628665 | immunoglobulin light chain constant region [Homo sapiens] | 116.72 | 91.43% | 1 | 1 | 3 | 82 | 35 | 3.9 | 6.48 | 1.973 |
| 21668722 | immunoglobulin heavy chain VHDJ region [Homo sapiens] | 99.89 | 18.11% | 424 | 1 | 2 | 8 | 127 | 13.7 | 8.85 | 2.02 |
| 194390508 | unnamed protein product [Homo sapiens] | 1308.75 | 40.93% | 24 | 1 | 25 | 226 | 1014 | 111.2 | 6.21 | 2.063 |
| 221042228 | unnamed protein product [Homo sapiens] | 15.94 | 2.78% | 1 | 1 | 1 | 1 | 539 | 61.3 | 7.74 | 2.185 |
| 58222449 | anti-tetanus toxoid immunoglobulin heavy chain variable region [Homo sapiens] | 102.65 | 21.49% | 133 | 1 | 3 | 20 | 121 | 13.2 | 8.46 | 2.245 |
| 114385630 | immunoglobulin light chain variable region [Homo sapiens] | 76.22 | 16.82% | 2 | 1 | 1 | 19 | 107 | 11.7 | 7.97 | 2.325 |
| 94469923 | anti-West Nile virus immunoglobulin light chain variable region [Homo sapiens] | 38.87 | 21.82% | 1 | 1 | 1 | 1 | 110 | 12 | 7.96 | 4.371 |
| 2253348 | immunoglobulin heavy chain variable region [Homo sapiens] | 69.15 | 22.13% | 31 | 1 | 2 | 3 | 122 | 13.4 | 9.07 | 16.498 |
